# Modular and Selective Arylation of Aryl Germanes (C−GeEt_3_) over C−Bpin, C−SiR_3_ and Halogens Enabled by Light‐Activated Gold Catalysis

**DOI:** 10.1002/anie.202005066

**Published:** 2020-06-12

**Authors:** Grant J. Sherborne, Avetik G. Gevondian, Ignacio Funes‐Ardoiz, Amit Dahiya, Christoph Fricke, Franziska Schoenebeck

**Affiliations:** ^1^ Institute of Organic Chemistry RWTH Aachen University Landoltweg 1 52074 Aachen Germany

**Keywords:** DFT calculations, organogermanium, photoredox catalysis

## Abstract

Selective C${}_{\mathrm{sp}{}^{2}}$ –C${}_{\mathrm{sp}{}^{2}}$ couplings are powerful strategies for the rapid and programmable construction of bi‐ or multiaryls. To this end, the next frontier of synthetic modularity will likely arise from harnessing the coupling space that is orthogonal to the powerful Pd‐catalyzed coupling regime. This report details the realization of this concept and presents the fully selective arylation of aryl germanes (which are inert under Pd^0^/Pd^II^ catalysis) in the presence of the valuable functionalities C−BPin, C−SiMe_3_, C−I, C−Br, C−Cl, which in turn offer versatile opportunities for diversification. The protocol makes use of visible light activation combined with gold catalysis, which facilitates the selective coupling of C−Ge with aryl diazonium salts. Contrary to previous light‐/gold‐catalyzed couplings of Ar–N_2_^+^, which were specialized in Ar–N_2_^+^ scope, we present conditions to efficiently couple electron‐rich, electron‐poor, heterocyclic and sterically hindered aryl diazonium salts. Our computational data suggest that while electron‐poor Ar–N_2_^+^ salts are readily activated by gold under blue‐light irradiation, there is a competing dissociative deactivation pathway for excited electron‐rich Ar–N_2_^+^, which requires an alternative photo‐redox approach to enable productive couplings.

## Graphical abstract

**Light‐activated gold catalysis** facilitates selective arylation of aryl germanes (which are inert under Pd^0/^Pd^II^ catalysis) in the presence of valuable functionalities such as C−BPin, C−SiMe_3_, C−I, C−Br, andC−Cl, which in turn offer versatile opportunities for diversification.

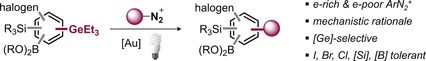

Biaryls are privileged motifs in materials, pharmaceuticals, agrochemicals, and natural products.^1^ Their function is strongly dictated by their substitution pattern, and as such, a direct, programmable and modular approach towards diversely and densely functionalized biaryl motifs is of significant interest. In this context, site‐selective and sequential Pd‐catalyzed cross‐coupling strategies represent the state of the art to build a diversifiable biaryl core in a modular fashion via differentiation at either the oxidative addition step (e.g. C−Br vs. C−Cl vs. C−OTf)^2^, ^3^ or the transmetalation step (e.g. B(OH)_2_ vs. BMIDA or R_3_Sn vs. BPin);^4^ see Figure 1. The extent of modularity is dictated by the ability to control functionalization at one against all competing coupling sites, but is also inherently limited by the number of available (pseudo)halogens and transmetalating agents (these being: ‐SiR_3_, ‐SnR_3_, ‐BR_2_). Recently, our group added organogermanes (‐GeR_3_)^5^ to the selectivity toolbox, which are robust, non‐toxic, easily installable,^6^ and unreactive in standard Pd^0^/Pd^II^‐catalyzed couplings, while privileged coupling partners with aryl iodides under nanoparticle catalysis that outcompete for example, ArBPin or ArBMIDA.^7^ However, the currently available sequential or iterative coupling repertoire with Pd is far from complete, and the increasing demands towards a rapid and automated exploration of chemical space will greatly benefit from new and orthogonal disconnections.^8^ For example, none of these methods allow to assemble richly decorated small molecules in the presence of C−iodine bonds, which owing to their pronounced halogen bonding^9^ are receiving increasing interest in medicinal and materials chemistry.^10^ In this context, oxidative gold catalysis^11^ has emerged as a powerful alternative to construct C${}_{\mathrm{sp}{}^{2}}$ –C${}_{\mathrm{sp}{}^{2}}$ bonds in the presence of aromatic C−halogen, including C−I bonds via the coupling of Ar−FG with ArH (see Figure 1).^12^ However, intramolecular competitions of for example, C−Bpin vs. C−SiMe_3_ or C−GeR_3_ vs. C−SiR_3_ functionalizations are—with the exception of ArH=indole^13^ and ArF_n_ 12h—not yet possible for a wide range of substrates due to incompatibilities with the strong stoichiometric oxidant that is mechanistically necessary to re‐oxidize Au^I^ to Au^III^. A promising alternative approach is the use of aryl diazonium salts^14^ under light activation,^15^ which obviates the need for an additional oxidant, as the ArN_2_^+^ serves as both, the oxidant and coupling partner.^16^, ^17^, ^18^ Hashmi et al. recently showed that this strategy enables the selective arylation of aryl silanes (C−SiMe_3_) in the presence of C−BPin or C−B(MIDA) as well as C−halogens, albeit under the limitation that the aryl diazonium salt had to be electron‐poor.^19^

**Figure 1:**
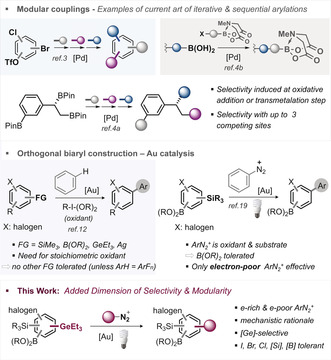
Overview of state‐of‐the‐art of modular synthesis and this work.

In light of our recent studies that identified organogermanes as highly reactive functionality in gold‐catalyzed couplings with ArH,12g we envisioned that we might be able to widen the scope in ArN_2_^+^ electronics as well as add another dimension of modularity in biaryl construction by developing a selective arylation of C−GeEt_3_ under light activation that tolerates all other widely used and typical coupling functionalities, that is, C−BPin, C−SiMe_3_ as well as halogens (C−I, C−Br and C−Cl). Undoubtedly, such a multi‐selective process would enable powerful opportunities for downstream diversification and synthetic modularity.

We initially set out to investigate the feasibility of the arylation of triethyl(4‐fluorophenyl)germane with 4‐nitrobenzenediazonium tetrafluoroborate and examined various commonly employed Au^I^ catalysts under blue LED irradiation in MeCN (see Supporting Information for additional details).^20^ Pleasingly, the relatively cheap, commercially available and air‐stable [(PPh_3_)AuCl] proved to be effective and gave the corresponding coupling product in 96 % yield after 2 h reaction time at room temperature. In the absence of gold catalyst and/or visible light, no reaction took place (see Supporting Information). We subsequently explored the scope of the couplings and investigated especially those aryl germanes that would, as the corresponding aryl boronic acids, be unstable and sluggishly reactive or incompatible in the widely employed Pd‐catalyzed Suzuki cross coupling,^21^ that is, highly fluorinated, heterocyclic, sterically hindered as well as halogenated aryl germanes.^7^ The presence of fluorine in *ortho*‐ or *para‐*positions of the germane (**1**–**3**, see Table 1) as well as electron‐rich heterocycles, such as the sterically demanding isoxazole (**16**), 2‐ and 3‐substituted thiophenes (**15**, **17**), and a 1,3‐benzodioxole (**18**) were well tolerated. Notably, analogous couplings of the latter previously needed metal and base additives to proceed.16c Similarly, the halogens Cl, Br, and I were fully tolerated in the couplings (**5**–**14**), offering powerful handles for follow‐up functionalizations by established Pd catalysis. A frequent challenge in gold‐catalyzed coupling processes is *ortho*‐substitution.21b Pleasingly, *ortho*‐substituted aryl germanes (**19**–**23**) show excellent coupling yields, even for the sterically demanding *i*Pr group (**19** and **20**). Owing to the absence of an external oxidant, the sensitive allyl ether group was found to be tolerated (**22**). The *ortho* tolerance also extends to the aryl diazonium salt, where excellent yields were obtained with the *ortho*‐nitro aryldiazonium salt in combination with a multiply halogenated germane (**24**), and with an *ortho*‐nitrile‐containing substrate, giving the 2,2′‐substituted 1‐1′‐biphenyl motif (**23**).

**Table 1:** Substrate scope of photocatalyst‐free light‐mediated arylation of aryl germanes.

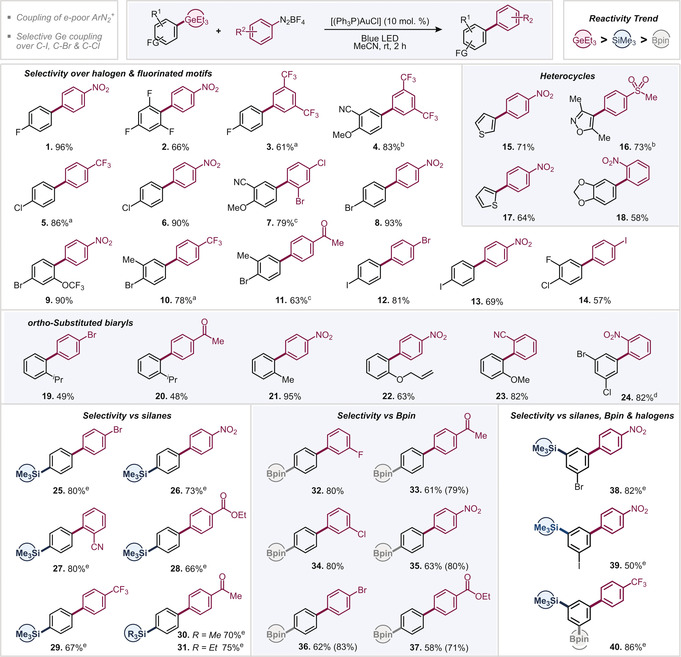

Reaction conditions: ArGeEt_3_ (0.3 mmol), ArN_2_BF_4_ (0.45 mmol), [(PPh_3_)AuCl] (0.03 mmol), MeCN (3 mL). [a] 7 h reaction time. [b] 16 h reaction time. [c] 3 h reaction time. [d] 5 h reaction time. [e] 1.2 equiv. of ArN_2_BF_4_.

In light of the observed relatively short reaction times of ca. 2 h as compared to the reported coupling times of aryl silanes (ca. 10 h^19^), we envisioned that chemoselective couplings under *intra*molecular competitions of aryl germanes versus silanes and boronic esters might be feasible. We observed exclusive arylation of the GeEt_3_ over SiMe_3_ (**25**–**30**), SiEt_3_ (**31**), and Bpin (**32**–**37**) functionalities along with tolerating C−halogen bonds, *ortho* and electron‐withdrawing substituents, such as ester, ketone, nitro, nitrile, and CF_3_ groups. As such, the exceptional high reactivity of germanes allows for the construction of the biaryl motif in the presence of valuable silane, BPin, halogen sites as well as additional functionality.

When employing electron‐rich aryl diazonium salts, however, such as *para*‐methoxyaryl ArN_2_^+^, we neither observed product formation nor consumption of starting materials under these conditions (and also stoichiometric reactivity tests, see page S54 in the Supporting Information). These observations are in line with previous silane functionalizations that were shown with solely electron‐deficient ArN_2_^+^.^19^

In an effort to understand the origins of these divergent reactivities, we undertook mechanistic and computational investigations. Our UV/Vis spectroscopic measurements (see page S56 in the Supporting Information) indicate that the absorption for the electron‐rich diazonium salts is at much shorter wavelength and outside the range of visible light, suggesting that there might not be sufficient absorption and activation by light. However, when we subsequently replaced visible by more powerful UV light, we observed full consumption of the ArN_2_^+^, but only trace amounts (<1 %) of coupling products. These results suggest that there must be another aspect of the reactivity difference.

Our computational studies^22^ revealed the likely cause: mechanistically, the light‐activated (=excited) diazonium–gold complex will undergo *Intersystem Crossing* (ISC) to reach the triplet energy surface (which is a feasible process due to the strong spin‐orbit coupling in heavy atoms like gold^23^). In the triplet state, there are then two competing pathways (see Figure 2): there is either productive addition to Au under nitrogen loss (to **Int3**) or, alternatively, the gold–diazonium complex dissociates, eventually causing decomposition of the aryl diazonium salt via **Int2**. Interestingly, while the productive pathway is calculated to be favored for the electron‐poor Ar–N_2_^+^, the electron‐rich examples will—if excited to their triplet state—preferentially dissociate from gold and release N_2_ to eventually generate undesired species. These findings are in line with the observed unproductive consumption of electron‐rich Ar–N_2_^+^ under more powerful UV‐irradiation.

**Figure 2:**
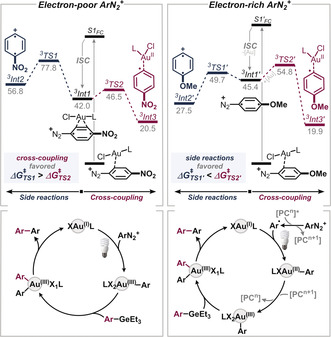
Calculated barriers of competing pathways for cross‐coupling vs. side reaction for electron‐deficient (left) and electron‐rich (right) diazonium salts and proposed catalytic cycles (bottom). Free energies are shown.^22^ For further details see Supporting Information. [“PC”=photocatalyst].

As such, simple variation of wavelength to potentially promote oxidative addition also to electron‐rich diazonium salts will not be sufficient, and a fundamentally different mechanism is needed to circumvent the lack of absorption of Ar–N_2_^+^ and de‐coordination from Au. Inspired by Fouquet's findings that the addition of a photosensitizer significantly enhanced the rate and yields in Au‐catalyzed arylation of aryl boronic acids,^24^ we next set out to explore the effectiveness of an added photocatalyst, [Ru(bpy)_3_][PF_6_]_2_. However, the pronounced redox ability led to premature reductions of the Ar–N_2_^+^, while triggering homocoupling of the aryl germane. In an attempt to favor the productive gold‐catalyzed cross‐coupling, we investigated alternative ligands for Au and found that the bidentate ligand, dppe was optimal (see Supporting Information). The use of [(Me_2_S)AuCl]/dppe in conjunction with the photocatalyst strongly favored the productive coupling for the electron‐rich *para*‐methoxyaryl diazonium salt.

With these conditions in hand, we subsequently explored the wider scope for couplings (see Table 2). To our delight, we found consistently high reactivity when reacting 4‐methoxybenzene‐diazonium tetrafluoroborate with various aryl germanes (**41**–**44**, **47**, **53**). The halogen and Bpin selectivity was found to be retained also in the presence of the photocatalyst. The germanium group proved to be highly reactive under these conditions also, with reactions being complete within 2 h. There was no competitive functionalization at C−SiMe_3_ (**48**). Small quantities of homocoupled aryl germane could not be eliminated in full, however. Alternative electron‐rich diazonium salts, such as alkyl‐ or aryl‐substituted examples coupled efficiently with aryl germanes (**45**–**46**, **48**–**50**, **52**). *Ortho* substitution was also examined and found to be tolerated, in both, the diazonium salt (**51**–**52**) as well as aryl germane (**53**).

**Table 2:** Substrate scope for reactions with electron‐rich diazonium salts.

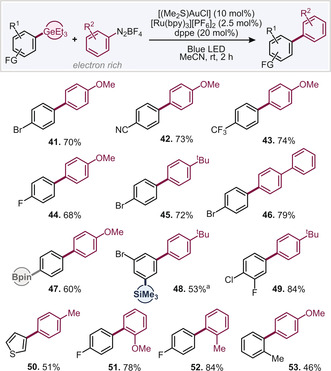

Typical conditions: ArGeEt_3_ (0.3 mmol), ArN_2_BF_4_ (0.6 mmol), [(SMe_2_)AuCl] (0.03 mmol), dppe (0.06 mmol) [Ru(bpy)_3_][PF_6_]_2_ (0.0075 mmol) MeCN (3 mL). [a] 1.2 equiv. of ArN_2_BF_4_.

As such, the blue light/[(Ph_3_P)AuCl] promoted conditions for electron‐deficient and the photocatalyst/visible light/[(Me_2_S)AuCl]/dppe promoted conditions for electron‐rich aryl diazonium salts overall enable the chemoselective arylation of electron‐rich or electron‐poor aryl germanes in the presence of SiMe_3_, BPin, and various halogen sites, even in the presence of challenging *ortho*‐substituents or heterocycles. All electronic combinations of biaryl are therefore accessible, which offers a powerful platform for further diversification to any desired follow‐up motif.

To illustrate this further (Figure 3), we applied our newly developed protocol to poly‐functionalized substrates (**54**, **56**, **58**, and **60**) and performed a fully selective arylation of the germanium group in the presence of C−Br, C−Bpin, and/or C−SiMe_3_. The resulting biaryl scaffolds were further diversified by rapid and air‐tolerant arylation or alkylation of C−Br enabled by Pd^I^–iodo dimer chemistry^3^, ^25^ as well as Chan‐Lam or Suzuki couplings of C−BPin and iodination and acetoxylation of C−SiMe_3_.

**Figure 3:**
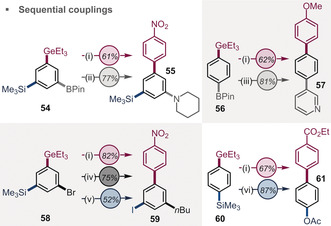
i) ArGeEt_3_ (0.3 mmol), ArN_2_BF_4_ (0.36–0.45 mmol), [(PPh_3_)AuCl] (0.03 mmol), MeCN; ii) ArBpin (0.2 mmol), Cu(OAc)_2_ (0.2 mmol), NEt_3_ (0.4 mmol), piperidine (0.4 mmol), MeCN; iii) ArBpin (0.20 mmol), ArBr (0.22 mmol), K_2_CO_3_ (0.40 mmol), Pd(dppf)Cl_2_⋅CH_2_Cl_2_ (0.01 mmol), 1,4‐dioxane; iv) ArBr (0.20 mmol), AlkZnCl/LiCl (0.46 mmol), Pd^I^‐I‐dimer (0.005 mmol); v) ArSiMe_3_ (0.3 mmol), N‐iodo succinimide (0.6 mmol), *p*TsOH (0.6 mmol), CHCl_3_; vi) ArSiMe_3_ (0.2 mmol), PhI(O_2_CCF_3_) (0.3 mmol), Pd(OAc)_2_ (0.01 mmol), AcOH.

In summary, the fully selective arylation of C−GeEt_3_ groups in the presence of C−BPin, C−SiMe_3_, C−I, C−Br, and C−Cl under light‐activated gold catalysis has been demonstrated herein. The method makes use of readily accessible aryl diazonium salts as coupling partner and is characterized by operational simplicity, mildness, and short reaction times. Our mechanistic studies revealed that while electron‐deficient Ar–N_2_^+^ couple directly under visible light/gold catalysis, electron‐rich analogues suffer from lack of absorption and even if excited with more powerful UV‐light will instead undergo dissociation from the gold catalyst, which causes their unproductive consumption. The mechanistic deviation to a photoredox‐based approach paired with a bidentate phosphine ligand‐coordinated gold catalyst, allowed us to circumvent these mechanistic challenges, enabling efficient and selective couplings also with electron‐rich Ar–N_2_^+^. Given the inertness of C−GeEt_3_ in typical Pd^0^/Pd^II^ catalysis, an orthogonal coupling space to the widely employed Pd cross‐coupling regime has been unleashed herein, which creates an additional dimension for modular, sequential, and iterative syntheses, which are of utmost value to rapidly access richly functionalized molecules, especially in the context of automated synthesis.

## Conflict of interest

The authors declare no conflict of interest.

## Supporting information

As a service to our authors and readers, this journal provides supporting information supplied by the authors. Such materials are peer reviewed and may be re‐organized for online delivery, but are not copy‐edited or typeset. Technical support issues arising from supporting information (other than missing files) should be addressed to the authors.

Supplementary
