# Supplementary material for: Modular and Selective Arylation of Aryl Germanes (C−GeEt3) over C−Bpin, C−SiR3 and Halogens Enabled by Light‐Activated Gold Catalysis
Source: Angew Chem Int Ed Engl. 2020 Jun 12;59(36):15543–8. doi: 10.1002/anie.202005066 (PMC7496160; doi:10.1002/anie.202005066)
Supplement: Supplementary file 1 — Supplementary [file ANIE-59-15543-s001.pdf]

Angewandte Chemie
Eine Zeitschrift der Gesellschaft Deutscher Chemiker

Supporting Information

Modular and Selective Arylation of Aryl Germanes (C−$GeEt_3$) over C−Bpin, C−SiR_3 and Halogens Enabled by Light-Activated Gold Catalysis

*Grant J. Sherborne, Avetik G. Gevondian, Ignacio Funes-Ardoiz, Amit Dahiya, Christoph Fricke, and Franziska Schoenebeck**

anie_202005066_sm_miscellaneous_information.pdf

Contents

1. General Experimental Details S2
2. General Procedures S4
  2.1 Au-catalyzed Cross-coupling of Aryl Germanes with *e*-Poor ArN_2BF_4 .... S4
  2.2 Au-catalyzed Cross-coupling of Aryl Germanes with *e*-Rich ArN_2BF_4 .... S22
  2.3 Synthesis of Diazonium Salts S28
  2.4 Synthesis of Aryl Triethylgermanes S34
  2.5 Synthesis of Bpin-substituted Aryl Germanes S44
  2.6 Synthesis of *p*-F-PhB(MIDA) and *p*-F-$PhSiMe_3$ S45
3. Derivatization of Biaryal Scaffolds S47
4. Reaction Development S52
  4.1 Catalyst Screening for Electron Poor System S52
  4.2 Ligand Screening for Electron Rich System S53
5. Mechanistic Investigations S54
  5.1 Stoichiometric Experiments S54
  5.2 UV/Vis Analysis S56
  5.3 Experiment with UV Light S57
6. Computational Details S58
  6.1 General Computational Details S58
  6.2 Competing Pathways in the Activation of *e*-Rich and *e*-Poor ArN_2BF_4 .... S59
  6.3 Photoredox Reduction of *e*-Rich ArN_2BF_4 .... S60
  6.4 XYZ Coordinates and Energies for Optimized Structures S61
7. NMR Spectra S73
8. References S135

1. General Experimental Details

All the reagents and the starting materials were purchased from Sigma-Aldrich, Alfa Aesar, abcr, TCI, Fluorochem or Acros Organics and used as received. Gold complexes were purchased from ChemPur, Sigma-Aldrich and abcr. Anhydrous THF and toluene were dried using an Innovative Technology PS-MD-5 solvent purification system. DMSO, degassed and anhydrous acetonitrile was purchased from Sigma-Aldrich. Solvents used in work up and purification were distilled prior to use.

Thin layer chromatography (TLC) was performed on Merck Kieselgel 60 F254 aluminium plates with unmodified silica and visualized either under UV light or stained with $KMnO_4$. Flash column chromatography was performed with Merck silica gel 60 (35 – 70 mesh). Preparative HPLC was performed on a Gilson-Abimed HPLC (employing UV detector model 117) using a Merck LiChrosorb Si60 column (porosity 7 μm, 250 x 25 mm).

All the ^{1}H, ^{13}C, ^{19}F and ^{31}P NMR spectra were recorded on Bruker Avance Neo 600, Varian VNMRS 600, Varian VNMRS 400 or Varian VNMRS 300 spectrometers at ambient temperature. Chemical shifts (δ) are reported in parts per million (ppm) and were referenced either to residual solvent peak ($CDCl_3$, CD_3CN, DMSO-d_6 for ^{1}H and ^{13}C spectra) or by the instrument internally after locking and shimming to the deuterated solvent (for ^{19}F and ^{31}P). Coupling constants (*J*) are given in Hertz (Hz). Multiplicities of signals in ^{1}H, ^{19}F, and ^{13}C NMR were designated as s (singlet), d (doublet), dd (doublet of doublets), dt (doublet of triplets), ddd (doublet of doublets of doublets), t (triplet), td (triplet of doublets), q (quartet), quint (quintet), sext (sextet), sept (septet), and m (multiplet).

Gas chromatography coupled with mass spectrometry (GC-MS) was performed on an Agilent Technologies 5975 series MSD mass spectrometer under electron ionization (EI) mode coupled with an Agilent Technologies 7820A gas chromatograph employing an Agilent 19091s-433 HP-5MS column (30 m × 0.250 μm × 0.250 μm) or Agilent CP-Sil8-CB column (30 m × 0.25 μm × 1.00 μm). High-resolution mass spectrometry (HRMS) was performed using a Thermo Scientific LTQ Orbitrap XL spectrometer, Finnigan MAT95 and Bruker Maxis II LC-MS-System. Low-resolution masses of known compounds were extracted from their GC-MS chromatograms. IR spectra were recorded on a Spectrum 100 spectrometer with an UATR Diamond/KRS-5 crystal with attenuated total reflectance (ATR). UV/Vis spectra were recorded on Shimadzu UV-2600 spectrophotometer.

All the reactions were carried out in oven dried glassware under an argon atmosphere either in an argon-filled glovebox or by using *Schlenk* techniques, unless otherwise specified. The reactions with blue light were performed in a setup made of a foiled vessel (135 mm) equipped with a blue LED stripe (1 m, 18.6 W, 465 – 470 nm, λ_{max} = 467 nm), a stirring plate, and two cooling fans providing ambient temperature (Figure S1). During experiments the setup was protected by a foil-covered shield from external sources of light. The blue LED stripe was purchased from ledxon® GmbH.

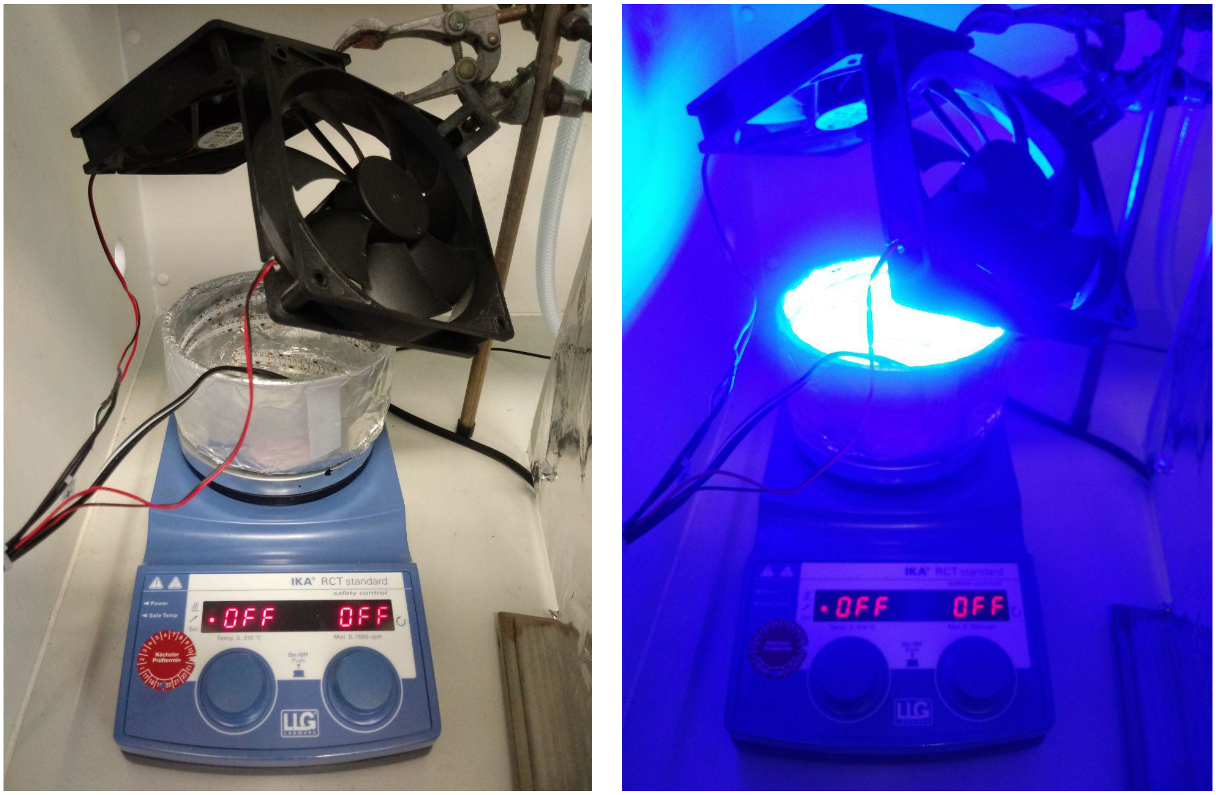

Figure S1. The blue LED setup off (left) and on (right)

2. General Procedures

2.1 Au-catalyzed Cross-coupling of Aryl Germanes with *e*-Poor ArN_2BF_4

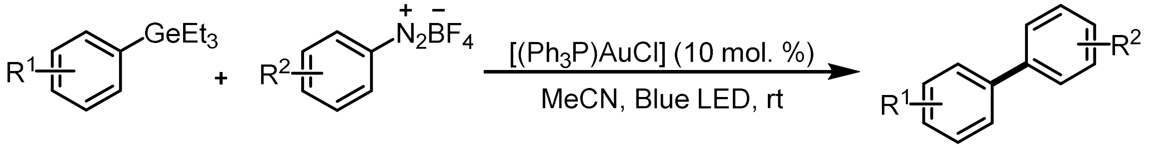

General Procedure 1 (GP 1)

In an argon-filled glovebox, corresponding aryl triethylgermane (0.300 mmol, 1.0 equiv.), diazonium salt (0.450 mmol, 1.5 equiv.), and $[(Ph_3P)AuCl]$ (0.030 mmol, 10 mol. %) were mixed in a screw top vial equipped with magnetic stirring bar and dissolved in anhydrous and degassed MeCN (3 ml). The vial with the solution was placed into the blue LED setup. After full consumption of aryl germane (determined by GC-MS) the crude mixture was concentrated *in vacuo,* and residue was purified by silica gel column chromatography. Reaction time is specified for the individual compounds.

3,5-Dimethyl-4-(4-(methylsulfonyl)phenyl)isoxazole

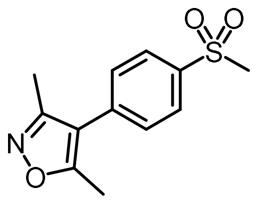

The cross-coupling was performed according to GP 1 by using 3,5-dimethyl-4-(triethylgermyl)isoxazole (76.8 mg, 0.300 mmole, 1.0 equiv.) and 4-(methylsulfonyl)benzenediazonium tetrafluoroborate (121.5 mg, 0.450 mmol, 1.5 equiv.). Reaction time: 16 h. The title product was obtained after purification by column chromatography (1:1 *n*-pentane/EtOAc) as a white solid (55.0 mg, 0.219 mmol, 73%).

$\mathbf{R_f}$ = 0.30 (1:1 *n*-pentane/EtOAc). **^{1}H NMR** (600 MHz, $CDCl_3$) δ/ppm = 8.02 (d, *J* = 8.4 Hz, 2H), 7.47 (d, *J* = 8.4 Hz, 2H), 3.11 (s, 3H), 2.45 (s, 3H), 2.30 (s, 3H). **^{13}C NMR** (151 MHz, $CDCl_3$) δ/ppm = 166.4, 158.3, 139.8, 136.6, 130.0, 128.1, 115.5, 44.7, 11.9, 11.0. **IR** (neat): ν/cm^{-1} = 3013, 2923, 2854, 1712, 1621, 1564, 1487, 1419, 1393, 1303, 1235, 1145, 1088, 1024, 997, 953, 893, 840, 772, 722. **HRMS** (ESI) calculated for $C_{12}H_{14}NO_3S$: 252.0689 $[M+H]^+$, found: 252.0686.

4'-Bromo-2-isopropyl-1,1'-biphenyl

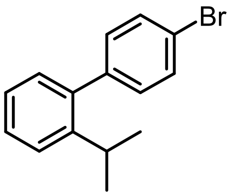

The cross-coupling was performed according to GP 1 by using triethyl(2-isopropylphenyl)germane (83.7 mg, 0.300 mmol, 1.0 equiv.) and 4-bromobenzenediazonium tetrafluoroborate (121.9 mg, 0.450 mmol,

1.5 equiv.). Reaction time: 2 h. The title product was obtained after purification by column chromatography (*n*-hexane) and preparative HPLC (95:5 *n*-hexane/EtOAc) as a colorless oil (40.5 mg, 0.147 mmol, 49%).

R$_f$ = 0.53 (*n*-hexane). **^{1}H NMR** (400 MHz, $CDCl_3$) δ/ppm = 7.55 (d, *J* = 8.2 Hz, 2H), 7.44-7.34 (m, 2H), 7.25-7.13 (m, 4H), 3.02 (sept, *J* = 6.9 Hz, 1H), 1.18 (d, *J* = 6.9 Hz, 6H). **^{13}C NMR** (101 MHz, $CDCl_3$) δ/ppm = 146.4, 141.1, 139.9, 131.3, 131.1, 129.9, 128.2, 125.8, 125.6, 121.1, 29.5, 24.4. **IR** (neat): ν/cm^{-1} = 3058, 3023, 2961, 2927, 2868, 2324, 2113, 1905, 1588, 1473, 1445, 1386, 1363, 1258, 1094, 1071, 1036, 1004, 946, 826, 756, 722. **HRMS** (APCI) calculated for $C_{15}H_{15}Br$: 274.0352 $[M]^+$, found: 274.0352.

4-Fluoro-4'-nitro-1,1'-biphenyl

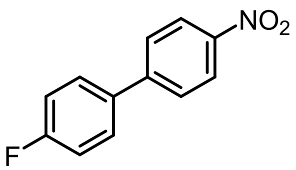

The cross-coupling was performed according to GP 1 by using triethyl(4-fluorophenyl)germane (76.5 mg, 0.300 mmole, 1.0 equiv.) and 4-nitrobenzenediazonium tetrafluoroborate (106.6 mg, 0.450 mmol, 1.5 equiv.). Reaction time: 2 h. The title product was obtained after purification by column chromatography (25:1 *n*-hexane/EtOAc) as a white solid (62.2 mg, 0.286 mmol, 96%).

R$_f$ = 0.50 (20:1 *n*-hexane/EtOAc). **^{1}H NMR** (400 MHz, $CDCl_3$) δ/ppm = 8.28 (d, *J* = 8.7 Hz, 2H), 7.69 (d, *J* = 8.7 Hz, 2H), 7.60 (dd, *J* = 8.7, 5.4 Hz, 2H), 7.23-7.13 (m, 2H). **^{13}C NMR** (101 MHz, $CDCl_3$) δ/ppm = 163.5 (d, *J* = 248.3 Hz), 147.2, 146.7, 135.0 (d, *J* = 3.1 Hz), 129.3 (d, *J* = 8.3 Hz), 127.5, 124.3, 116.3 (d, *J* = 21.6 Hz). **^{19}F NMR** (376 MHz, $CDCl_3$) δ/ppm = -112.67 – -112.82 (m, 1F). **HRMS** (EI) calculated for $C_{12}H_8FNO_2$: 217.0534 $[M]^+$, found: 217.0540. These data are in agreement with those reported previously in the literature.[1]

2-(4'-Bromo-[1,1'-biphenyl]-4-yl)-4,4,5,5-tetramethyl-1,3,2-dioxaborolane

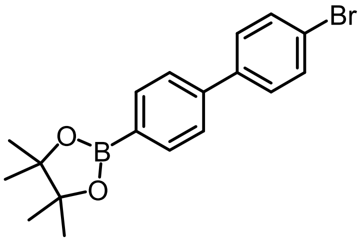

The cross-coupling was performed according to GP 1 by using triethyl(4-(4,4,5,5-tetramethyl-1,3,2-dioxaborolan-2-yl)phenyl)germane (108.9 mg, 0.300 mmol, 1.0 equiv.) and 4-bromobenzenediazonium tetrafluoroborate (121.9 mg, 0.450 mmol, 1.5 equiv.). Reaction time: 2 h. The title product was obtained after purification by column chromatography (DCM) as a white solid (89.0 mg, 0.248 mmol, 62% [83% according to NMR]).

R$_f$ = 0.73 (DCM). **^{1}H NMR** (600 MHz, $CDCl_3$) δ/ppm = 7.90 (d, *J* = 8.4 Hz, 2H), 7.56 (d, *J* = 8.3 Hz, 4H), 7.48 (d, *J* = 8.4 Hz, 2H), 1.38 (s, 12H). **^{13}C NMR** (101 MHz, $CDCl_3$) δ/ppm = 142.8, 140.1,

135.5, 132.0, 129.0, 126.4, 122.0, 84.1, 25.0 (carbon atom attached to boron atom is not observed). **HRMS** (EI) calculated for $C_{18}H_{20}B^{79}BrO_2$: 358.0734 $[M]^+$, found: 358.0737. These data are in agreement with those reported previously in the literature.[2]

4-Bromo-3-methyl-4'-(trifluoromethyl)-1,1'-biphenyl

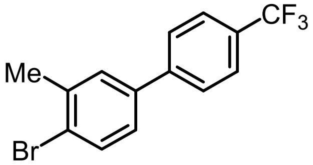

The cross-coupling was performed according to GP 1 by using (4-bromo-3-methylphenyl)triethylgermane (99.0 mg, 0.300 mmol, 1.0 equiv.) and (4-trifluoromethyl)benzenediazonium tetrafluoroborate (117.0 mg, 0.450 mmol, 1.5 equiv.). Reaction time: 7 h. The title product was obtained after purification by column chromatography (*n*-hexane) and preparative HPLC (95:5 *n*-hexane/EtOAc) as a colorless oil (73.7 mg, 0.234 mmol, 78%).

R$_f$ = 0.58 (*n*-hexane). **^{1}H NMR** (600 MHz, $CDCl_3$) δ/ppm = 7.69 (d, *J* = 8.3 Hz, 2H), 7.65 (d, *J* = 8.3 Hz, 2H), 7.62 (d, *J* = 8.2 Hz, 2H), 7.45 (d, *J* = 2.3 Hz, 2H), 7.29-7.25 (m, 1H), 2.48 (s, 3H). **^{13}C NMR** (151 MHz, $CDCl_3$) δ/ppm = 143.9, 139.1, 138.7, 133.1, 129.8 (q, *J* = 32.5 Hz), 129.7, 127.4, 126.3, 125.9 (q, *J* = 3.7 Hz), 125.2, 124.4 (q, *J* = 271.9 Hz), 23.2. **^{19}F NMR** (565 MHz, $CDCl_3$) δ/ppm = -62.47 (s, 3F). **IR** (neat): ν/cm^{-1} = 2952, 2855, 2326, 2102, 1921, 1616, 1562, 1473, 1417, 1381, 1323, 1165, 1119, 1070, 1022, 885, 843, 813, 743, 660. **HRMS** (EI) calculated for $C_{14}H_{10}{}^{79}BrF_3$: 313.9912 $[M]^+$, found: 313.9906.

2-(4-Nitrophenyl)thiophene

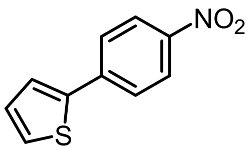

The cross-coupling was performed according to GP 1 by using triethyl(thiophen-2-yl)germane (72.9 mg, 0.300 mmole, 1.0 equiv.) and 4-nitrobenzenediazonium tetrafluoroborate (106.6 mg, 0.450 mmol, 1.5 equiv.). Reaction time: 2 h. The title product was obtained after purification by column chromatography (4:1, 2:1 *n*-hexane/DCM) as a yellow solid (39.7 mg, 0.193 mmol, 64%).

R$_f$ = 0.30 (2:1 *n*-hexane/DCM). **^{1}H NMR** (400 MHz, $CDCl_3$) δ/ppm = 8.24 (d, *J* = 8.9 Hz, 2H), 7.75 (d, *J* = 8.9 Hz, 2H), 7.48 (dd, *J* = 3.6, 1.0 Hz, 1H), 7.44 (dd, *J* = 5.1, 1.0 Hz, 1H), 7.15 (dd, *J* = 5.1, 3.6 Hz, 1H). **^{13}C NMR** (101 MHz, $CDCl_3$) δ/ppm = 146.7, 141.7, 140.7, 128.8, 127.8, 126.1, 125.8, 124.5. **HRMS** (ESI) calculated for $C_{10}H_7NNaO_2S$: 228.0090 $[M+Na]^+$, found: 228.0090. These data are in agreement with those reported previously in the literature.[3]

Trimethyl(4'-nitro-[1,1'-biphenyl]-4-yl)silane

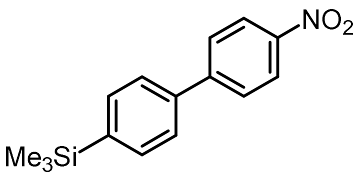

The cross-coupling was performed according to GP 1 by using trimethyl(4-(triethylgermyl)phenyl)silane (92.7 mg, 0.300 mmol, 1.0 equiv.) and 4-nitrobenzenediazonium tetrafluoroborate (85.3 mg, 0.360 mmol, 1.2 equiv.). Reaction time: 2 h. The title product was obtained after purification by column chromatography (6:1 to 2:1 *n*-hexane/DCM) as a pale yellow solid (59.4 mg, 0.219 mmol, 73%).

R_f = 0.12 (8:1 *n*-hexane/DCM). **^{1}H NMR** (400 MHz, $CDCl_3$) δ/ppm = 8.30 (d, *J* = 8.8 Hz, 2H), 7.75 (d, *J* = 8.8 Hz, 2H), 7.67 (d, *J* = 8.1 Hz, 2H), 7.61 (d, *J* = 8.1 Hz, 2H), 0.32 (s, 9H). **^{13}C NMR** (101 MHz, $CDCl_3$) δ/ppm = 147.7, 147.3, 141.8, 139.2, 134.3, 127.9, 126.8, 124.3, -1.0. **IR** (neat): ν/cm^{-1} = 2956, 2926, 2852, 1595, 1544, 1511, 1411, 1334, 1248, 1179, 1106, 1003, 837, 749, 694. **HRMS** (ESI) calculated for $C_{15}H_{17}NNaO_2Si$: 294.0921 [M+Na]$^+$, found: 294.0921.

Trimethyl(4'-(trifluoromethyl)-[1,1'-biphenyl]-4-yl)silane

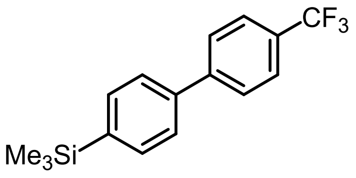

The cross-coupling was performed according to GP 1 by using trimethyl(4-(triethylgermyl)phenyl)silane (92.7 mg, 0.300 mmol, 1.0 equiv.) and (4-trifluoromethyl)benzenediazonium tetrafluoroborate (93.6 mg, 0.360 mmol, 1.2 equiv.). Reaction time: 2 h. The title product was obtained after purification by column chromatography (*n*-hexane) as a white solid (62.9 mg, 0.214 mmol, 67%, purity 96%).

R_f = 0.51 (*n*-hexane). **^{1}H NMR** (600 MHz, $CDCl_3$) δ/ppm = 7.70 (s, 4H), 7.64 (d, *J* = 8.2 Hz, 2H), 7.59 (d, *J* = 8.2 Hz, 2H), 0.32 (s, 9H). **^{13}C NMR** (151 MHz, $CDCl_3$) δ/ppm = 144.8, 140.7, 140.2, 134.2, 129.5 (q, *J* = 32.3 Hz), 127.6, 126.7, 125.9 (q, *J* = 3.8 Hz), 124.4 (q, *J* = 271.8 Hz), -1.0. **^{19}F NMR** (376 MHz, $CDCl_3$) δ/ppm = -62.44 (s, 3F). **IR** (neat): ν/cm^{-1} = 2957, 1696, 1615, 1542, 1413, 1386, 1320, 1252, 1164, 1109, 1067, 1006, 837, 811, 758, 741, 697. **HRMS** (EI) calculated for $C_{16}H_{17}F_3Si$: 294.1046 [M]$^+$, found: 294.1047.

Ethyl 4'-(trimethylsilyl)-[1,1'-biphenyl]-4-carboxylate

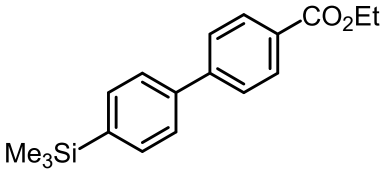

The cross-coupling was performed according to GP 1 by using trimethyl(4-(triethylgermyl)phenyl)silane (92.7 mg, 0.300 mmol, 1.0 equiv.) and 4-(ethoxycarbonyl)benzenediazonium tetrafluoroborate (95.0 mg, 0.360 mmol, 1.2 equiv.). Reaction time: 3 h. The title product was obtained after purification by column chromatography

(*n*-hexane to 25:1 *n*-hexane/EtOAc) and preparative HPLC (9:1 *n*-hexane/EtOAc) as a white solid (59.0 mg, 0.198 mmol, 66%).

R$_f$ = 0.30 (25:1 *n*-hexane/EtOAc). **^{1}H NMR** (600 MHz, $CDCl_3$) δ/ppm = 8.12 (d, *J* = 8.4 Hz, 2H), 7.67 (d, *J* = 8.4 Hz, 2H), 7.64-7.60 (m, 4H), 4.41 (q, *J* = 7.1 Hz, 2H), 1.42 (t, *J* = 7.1 Hz, 3H), 0.31 (s, 9H). **^{13}C NMR** (151 MHz, $CDCl_3$) δ/ppm = 166.7, 145.6, 140.6, 140.5, 134.1, 130.2, 129.5, 127.1, 126.7, 61.1, 14.5, -1.0. **IR** (neat): ν/cm^{-1} = 2955, 1712, 1604, 1476, 1446, 1410, 1371, 1311, 1273, 1205, 1178, 1104, 1023, 1003, 838, 756, 700. **HRMS** (ESI) calculated for $C_{18}H_{22}NaO_2Si$: 321.1281 [M+Na]$^+$, found: 321.1274.

4'-(Trimethylsilyl)-[1,1'-biphenyl]-2-carbonitrile

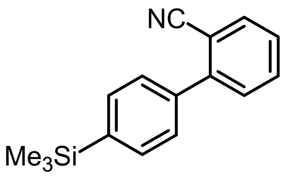

The cross-coupling was performed according to GP 1 by using trimethyl(4-(triethylgermyl)phenyl)silane (92.7 mg, 0.300 mmol, 1.0 equiv.) and 2-cyanobenzenediazonium tetrafluoroborate (78.1 mg, 0.360 mmol, 1.2 equiv.). Reaction time: 2 h. The title product was obtained after purification by column chromatography (15:1 *n*-hexane/EtOAc) as a dark yellow oil (60.3 mg, 0.240 mmol, 80%).

R$_f$ = 0.31 (15:1 *n*-hexane/EtOAc). **^{1}H NMR** (400 MHz, $CDCl_3$) δ/ppm = 7.81-7.75 (m, 1H), 7.70-7.61 (m, 3H), 7.59-7.50 (m, 3H), 7.47-7.41 (m, 1H), 0.32 (s, 9H). **^{13}C NMR** (101 MHz, $CDCl_3$) δ/ppm = 145.6, 141.4, 138.5, 133.9, 133.9, 133.0, 130.2, 128.1, 127.7, 118.9, 111.5, -1.0. **IR** (neat): ν/cm^{-1} = 3065, 2955, 2225, 2092, 1595, 1476, 1442, 1385, 1249, 1115, 1003, 839, 757, 694. **HRMS** (ESI) calculated for $C_{16}H_{17}NNaSi$: 274.1022 [M+Na]$^+$, found: 274.1022.

1-(4'-(Trimethylsilyl)-[1,1'-biphenyl]-4-yl)ethan-1-one

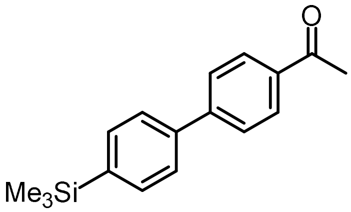

The cross-coupling was performed according to GP 1 by using trimethyl(4-(triethylgermyl)phenyl)silane (92.7 mg, 0.300 mmol, 1.0 equiv.) and 4-acetylbenzenediazonium tetrafluoroborate (84.2 mg, 0.360 mmol, 1.2 equiv.). Reaction time: 2 h. The title product was obtained after purification by column chromatography (15:1 *n*-hexane/EtOAc) as a pale yellow solid (56.6 mg, 0.211 mmol, 70%).

R$_f$ = 0.28 (15:1 *n*-hexane/EtOAc). **^{1}H NMR** (400 MHz, $CDCl_3$) δ/ppm = 8.04 (d, *J* = 8.6 Hz, 2H), 7.70 (d, *J* = 8.6 Hz, 2H), 7.64-7.62 (m, 4H), 2.64 (s, 3H), 0.31 (s, 9H). **^{13}C NMR** (101 MHz, $CDCl_3$) δ/ppm = 197.9, 145.9, 140.8, 140.3, 136.1, 134.1, 129.1, 127.4, 126.7, 26.8, -1.0. **HRMS** (ESI)

calculated for $C_{17}H_{20}NaOSi$: 291.1176 $[M+Na]^+$, found: 291.1185. These data are in agreement with those reported previously in the literature.[4]

5-(2-Nitrophenyl)benzo[*d*][1,3]dioxole

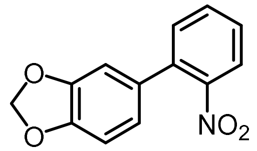

The cross-coupling was performed according to GP 1 by using benzo[*d*][1,3]dioxol-5-yltriethylgermane (84.3 mg, 0.300 mmol, 1.0 equiv.) and 2-nitrobenzenediazonium tetrafluoroborate (106.6 mg, 0.450 mmol, 1.5 equiv.). Reaction time: 2 h. The title product was obtained after purification by column chromatography (12:1 *n*-hexane/EtOAc) as a red oil (40.5 mg, 0.167 mmol, 58%).

R_f = 0.30 (12:1 *n*-hexane/EtOAc). **^{1}H NMR** (600 MHz, $CDCl_3$) δ/ppm = 7.80 (dd, *J* = 8.1, 1.3 Hz, 1H), 7.60-7.56 (m, 1H), 7.47-7.43 (m, 1H), 7.42 (dd, *J* = 7.7, 1.4 Hz, 1H), 6.86 (d, *J* = 7.9 Hz, 1H), 6.80 (d, *J* = 1.8 Hz, 1H), 6.78 (dd, *J* = 7.9, 1.8 Hz, 1H), 6.01 (s, 2H). **^{13}C NMR** (151 MHz, $CDCl_3$) δ/ppm = 149.6, 148.1, 148.0, 136.0, 132.3, 132.0, 131.1, 128.1, 124.2, 121.8, 108.8, 108.6, 101.5. **IR** (neat): *ν*/cm^{-1} = 2895, 1729, 1608, 1571, 1522, 1471, 1352, 1224, 1145, 1109, 1037, 933, 892, 855, 813, 781, 749, 712, 666. **HRMS** (ESI) calculated for $C_{13}H_9NNaO_4$: 266.0424 $[M+Na]^+$, found: 266.0423.

3-(4-Nitrophenyl)thiophene

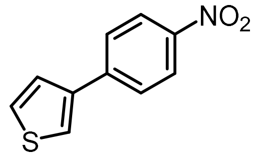

The cross-coupling was performed according to GP 1 by using triethyl(thiophen-3-yl)germane (72.9 mg, 0.300 mmole, 1.0 equiv.) and 4-nitrobenzenediazonium tetrafluoroborate (106.6 mg, 0.450 mmol, 1.5 equiv.). Reaction time: 2 h. The title product was obtained after purification by column chromatography (*n*-pentane to 20:1 *n*-pentane/EtOAc) as a yellow solid (43.5 mg, 0.212 mmol, 71%).

R_f = 0.27 (30:1 *n*-pentane/EtOAc). **^{1}H NMR** (600 MHz, $CDCl_3$) δ/ppm = 8.27 (d, *J* = 8.8 Hz, 2H), 7.74 (d, *J* = 8.8 Hz, 2H), 7.64 (dd, *J* = 2.9, 1.3 Hz, 1H), 7.47 (dd, *J* = 5.0, 2.9 Hz, 1H), 7.44 (dd, *J* = 5.0, 1.3 Hz, 1H). **^{13}C NMR** (151 MHz, $CDCl_3$) δ/ppm = 146.8, 142.1, 140.1, 127.5, 127.0, 126.2, 124.5, 123.3. **HRMS** (ESI) calculated for $C_{10}H_7NNaO_2S$: 228.0090 $[M+Na]^+$, found: 228.0090. These data are in agreement with those reported previously in the literature.[5]

1-(2'-Isopropyl-[1,1'-biphenyl]-4-yl)ethan-1-one

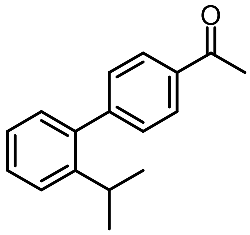

The cross-coupling was performed according to GP 1 by using triethyl(2-isopropylphenyl)germane (83.7 mg, 0.300 mmol, 1.0 equiv.) and 4-acetylbenzenediazonium tetrafluoroborate (105.3 mg, 0.450 mmol, 1.5 equiv.). Reaction time: 2 h. The title product was obtained after purification by column chromatography (20:1 *n*-pentane/EtOAc) as a pale yellow oil (34.1 mg, 0.143 mmol, 48%).

R$_f$ = 0.30 (20:1 *n*-pentane/EtOAc). **^{1}H NMR** (600 MHz, $CDCl_3$) δ/ppm = 8.01 (d, *J* = 8.3 Hz, 2H), 7.43-7.36 (m, 4H), 7.25-7.22 (m, 1H), 7.16 (dd, *J* = 7.6, 1.4 Hz, 1H), 2.99 (sept, *J* = 6.9 Hz, 1H), 2.66 (s, 3H), 1.16 (d, *J* = 6.9 Hz, 6H). **^{13}C NMR** (151 MHz, $CDCl_3$) δ/ppm = 198.0, 147.4, 146.3, 140.1, 135.7, 129.8, 129.7, 128.4, 128.3, 125.9, 125.6, 29.6, 26.8, 24.4. **IR** (neat): ν/cm^{-1} = 3059, 3023, 2962, 2927, 2869, 2324, 2092, 1682, 1603, 1559, 1479, 1443, 1400, 1357, 1262, 1180, 1105, 1035, 1005, 955, 839, 759, 668. **HRMS** (ESI) calculated for $C_{17}H_{18}NaO$: 261.1250 $[M+Na]^+$, found: 261.1250.

3'-Bromo-5'-chloro-2-nitro-1,1'-biphenyl

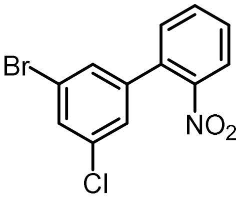

The cross-coupling was performed according to GP 1 by using (3-bromo-5-chlorophenyl)triethylgermane (105.1 mg, 0.300 mmol, 1.0 equiv.) and 2-nitrobenzenediazonium tetrafluoroborate (106.6 mg, 0.450 mmol, 1.5 equiv.). Reaction time: 7 h. The title product was obtained after purification by column chromatography (15:1 *n*-pentane/EtOAc) as a yellow solid (77.2 mg, 0.247 mmol, 82%).

R$_f$ = 0.27 (15:1 *n*-pentane/EtOAc). **^{1}H NMR** (600 MHz, $CDCl_3$) δ/ppm = 7.96 (dd, *J* = 8.2, 1.3 Hz, 1H), 7.68-7.63 (m, 1H), 7.58-7.54 (m, 2H), 7.39 (dd, *J* = 7.6, 1.4 Hz, 1H), 7.36-7.34 (m, 1H), 7.25-7.23 (m, 1H). **^{13}C NMR** (151 MHz, $CDCl_3$) δ/ppm = 148.7, 140.9, 135.4, 134.0, 133.0, 132.0, 131.3, 129.5, 127.1, 124.7, 123.0. **IR** (neat): ν/cm^{-1} = 3069, 2923, 2858, 2342, 2111, 1738, 1586, 1553, 1518, 1441, 1406, 1346, 1309, 1268, 1145, 1098, 1038, 890, 854, 809, 780, 741, 709, 676. **HRMS** (ESI) calculated for $C_{12}H_7{}^{79}Br^{35}ClNNaO_2$: 333.9241 $[M+Na]^+$, found 333.9240.

The resolution of the ^{13}C NMR does not allow for accurate assignment of all the signals.

2'-Methoxy-[1,1'-biphenyl]-2-carbonitrile

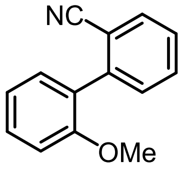

The cross-coupling was performed according to GP 1 by using triethyl(2-methoxyphenyl)germane (80.1 mg, 0.300 mmol, 1.0 equiv.) and 2-cyanobenzenediazonium tetrafluoroborate (97.6 mg, 0.450 mmol, 1.5 equiv.). Reaction time: 5 h. The title product was obtained after purification by column chromatography (20:1 *n*-pentane/EtOAc) as oil (50.8 mg, 0.243 mmol, 82%).

R_f = 0.30 (20:1 *n*-pentane/EtOAc). **^{1}H NMR** (600 MHz, $CDCl_3$) δ/ppm = 7.73 (dd, *J* = 7.8, 1.4 Hz, 1H), 7.64-7.60 (m, 1H), 7.47-7.45 (m, 1H), 7.43-7.40 (m, 2H), 7.26 (dd, *J* = 7.5, 1.7 Hz, 1H), 7.08-7.04 (m, 1H), 7.04-7.02 (m, 1H), 3.85 (s, 3H). **^{13}C NMR** (151 MHz, $CDCl_3$) δ/ppm = 156.6, 142.7, 132.9, 132.5, 131.1, 131.0, 130.5, 127.47, 127.43, 120.9, 118.8, 113.6, 111.5, 55.6. **HRMS** (ESI) calculated for $C_{14}H_{11}NNaO$: 232.0733 $[M+Na]^+$, found: 232.0732. These data are in agreement with those reported previously in the literature.[6]

2'-Bromo-4'-chloro-4-methoxy-[1,1'-biphenyl]-3-carbonitrile

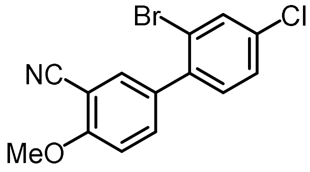

The cross-coupling was performed according to GP 1 by using 2-methoxy-5-(triethylgermyl)benzonitrile (87.6 mg, 0.300 mmol, 1.0 equiv.) and 2-bromo-4-chlorobenzenediazonium tetrafluoroborate (137.4 mg, 0.450 mmole, 1.5 equiv.). Reaction time: 3 h. The title product was obtained after purification by column chromatography (10:1 *n*-pentane/EtOAc) as a pale brown solid (67.9 mg, 0.210 mmol, 79%).

R_f = 0.30 (10:1 *n*-pentane/EtOAc). **^{1}H NMR** (600 MHz, $CDCl_3$) δ/ppm = 7.69 (d, *J* = 2.1 Hz, 1H), 7.59 (d, *J* = 2.2 Hz, 1H), 7.57 (dd, *J* = 8.7, 2.2 Hz, 1H), 7.36 (dd, *J* = 8.2, 2.1 Hz, 1H), 7.22 (d, *J* = 8.2 Hz, 1H), 7.04 (d, *J* = 8.7 Hz, 1H), 3.99 (s, 3H). **^{13}C NMR** (151 MHz, $CDCl_3$) δ/ppm = 161.0, 138.7, 135.5, 134.6, 134.6, 133.1, 132.9, 131.8, 128.1, 123.2, 116.2, 111.2, 101.9, 56.4. **IR** (neat): ν/cm^{-1} = 3085, 2924, 2850, 2308, 2226, 2116, 1908, 1734, 1608, 1581, 1550, 1502, 1464, 1411, 1367, 1283, 1263, 1191, 1130, 1095, 1065, 1012, 954, 920, 869, 815, 769, 741, 662. **HRMS** (ESI) calculated for $C_{14}H_9{}^{79}Br^{35}ClNNaO$: 343.9448 $[M+Na]^+$, found: 343.9443.

1-(4'-Bromo-3'-methyl-[1,1'-biphenyl]-4-yl)ethan-1-one

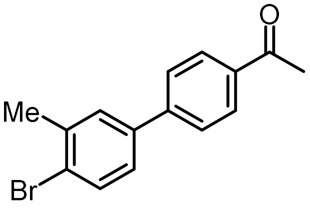

The cross-coupling was performed according to GP 1 by using (4-bromo-3-methylphenyl)triethylgermane (99.0 mg, 0.300 mmol, 1.0 equiv.) and 4-acetylbenzenediazonium tetrafluoroborate (105.3 mg, 0.450 mmol, 1.5 equiv.). Reaction time: 3 h. The title

product was obtained after purification by column chromatography (20:1 *n*-pentane/EtOAc) as a white solid (54.7 mg, 0.189 mmol, 63%).

R$_f$ = 0.20 (20:1 *n*-pentane/EtOAc). **^{1}H NMR** (600 MHz, $CDCl_3$) δ/ppm = 8.02 (d, *J* = 8.4 Hz, 2H), 7.65 (d, *J* = 8.4 Hz, 2H), 7.62 (d, *J* = 8.2 Hz, 1H), 7.48 (d, *J* = 2.2 Hz, 1H), 7.30 (dd, *J* = 8.2, 2.2 Hz, 1H), 2.64 (s, 3H), 2.48 (s, 3H). **^{13}C NMR** (151 MHz, $CDCl_3$) δ/ppm = 197.8, 144.9, 139.2, 138.6, 136.2, 133.0, 129.7, 129.1, 127.2, 126.2, 125.3, 26.8, 23.2. **IR** (neat): ν/cm^{-1} = 3055, 2921, 2853, 2324, 2115, 1740, 1675, 1598, 1512, 1469, 1439, 1415, 1351, 1259, 1190, 1151, 1120, 1078, 1021, 955, 899, 845, 809, 746, 705. **LC-MS** (APCI) calculated for $C_{15}H_{14}{}^{79}BrO$: 289.0223 $[M+H]^+$, found: 289.0225.

4-Methoxy-3',5'-bis(trifluoromethyl)-[1,1'-biphenyl]-3-carbonitrile

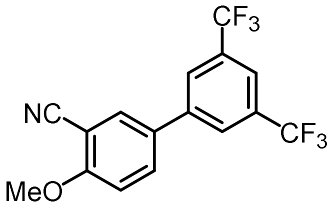

The cross-coupling was performed according to GP 1 by using 2-methoxy-5-(triethylgermyl)benzonitrile (87.6 mg, 0.300 mmol, 1.0 equiv.) and 3,5-bis(trifluoromethyl)benzenediazonium tetrafluoroborate (147.6 mg, 0.450 mmol, 1.5 equiv.). Reaction time: 16 h. The title product was obtained after purification by column chromatography (10:1 *n*-pentane/EtOAc) as an orange solid (85.5 mg, 0.248 mmol, 83%).

R$_f$ = 0.20 (10:1 *n*-pentane/EtOAc). **^{1}H NMR** (600 MHz, $CDCl_3$) δ/ppm = 7.95-7.92 (m, 2H), 7.89-7.86 (m, 1H), 7.82 (d, *J* = 2.4 Hz, 1H), 7.79 (dd, *J* = 8.7, 2.4 Hz, 1H), 7.12 (d, *J* = 8.7 Hz, 1H), 4.02 (s, 3H). **^{13}C NMR** (151 MHz, $CDCl_3$) δ/ppm = 161.7, 140.9, 133.3, 133.0, 132.7 (q, *J* = 33.4 Hz), 132.50, 131.3, 123.2 (q, *J* = 273.0 Hz), 121.6-121.4 (m), 115.9, 112.3, 103.1, 56.6. **^{19}F NMR** (376 MHz, $CDCl_3$) δ/ppm = -62.92 (s, 6F). **IR** (neat): ν/cm^{-1} = 2925, 2852, 2234, 1610, 1509, 1465, 1374, 1272, 1163, 1113, 1063, 1020, 894, 826, 745, 703, 680. **HRMS** (ESI) calculated for $C_{16}H_9F_6NNaO$: 368.0481 $[M+Na]^+$, found: 368.0477.

2,4,6-Trifluoro-4'-nitro-1,1'-biphenyl

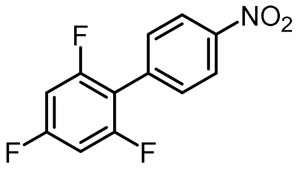

The cross-coupling was performed according to GP 1 by using triethyl(2,4,6-trifluorophenyl)germane (87.3 mg, 0.300 mmol, 1.0 equiv.) and 4-nitrobenzenediazonium tetrafluoroborate (106.6 mg, 0.450 mmol, 1.5 equiv.). Reaction time: 2 h. The title product was obtained after purification by column chromatography (20:1 *n*-hexane/EtOAc) as a white solid (50.1 mg, 0.198 mmol, 66%).

R$_f$ = 0.75 (20:1 *n*-hexane/EtOAc). **^{1}H NMR** (600 MHz, $CDCl_3$) δ/ppm = 8.31 (d, *J* = 8.8 Hz, 2H), 7.62 (d, *J* = 8.8 Hz, 2H), 6.88-6.77 (m, 2H). **^{13}C NMR** (151 MHz, $CDCl_3$) δ/ppm = 162.8 (dt, *J* = 251.9, 15.6 Hz), 160.2 (ddd, *J* = 251.2, 14.7, 9.2 Hz), 147.7, 135.3, 131.4, 123.7, 113.1 (td, *J* = 18.6, 4.9 Hz), 101.4-100.8 (m). **^{19}F NMR** (564 MHz, $CDCl_3$) δ/ppm = -106.02 – -106.10 (m, 1F), -110.83 - -110.92 (m, 2F). **IR** (neat): ν/cm^{-1} = 3092, 2927, 1635, 1597, 1510, 1445, 1398, 1341, 1170, 1116, 1022, 846, 724, 691. **HRMS** (ESI) calculated for $C_{12}H_6O_2NF_3Na$: 276.0243 $[M+Na]^+$, found: 276.0244.

2-(3'-Chloro-[1,1'-biphenyl]-4-yl)-4,4,5,5-tetramethyl-1,3,2-dioxaborolane

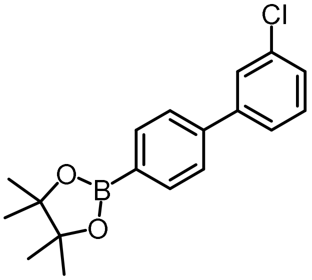

The cross-coupling was performed according to GP 1 by using triethyl(4-(4,4,5,5-tetramethyl-1,3,2-dioxaborolan-2-yl)phenyl)germane (108.9 mg, 0.300 mmol, 1.0 equiv.) and 3-chlorobenzenediazonium tetrafluoroborate (101.9 mg, 0.450 mmol, 1.5 equiv.). Reaction time: 2 h. The title product was obtained after purification by preparative TLC (20:1 *n*-hexane/EtOAc) as a white solid (75.5 mg, 0.240 mmol, 80%).

R$_f$ = 0.75 (20:1 *n*-hexane/EtOAc). **^{1}H NMR** (400 MHz, $CDCl_3$) δ/ppm = 7.89 (d, *J* = 8.3 Hz, 2H), 7.62-7.55 (m, 3H), 7.52-7.46 (m, 1H), 7.40-7.29 (m, 2H), 1.37 (s, 12H). **^{13}C NMR** (101 MHz, $CDCl_3$) δ/ppm = 143.0, 142.5, 135.5, 134.8, 130.1, 127.7, 127.5, 126.5, 125.5, 84.1, 25.0 (carbon atom attached to boron atom is not observed). **MS** (EI) *m/z* (%): 316 (16), 314 (48) $[M]^+$, 313 (14), 299 (28), 230 (25), 228 (73), 229 (18), 217 (24), 216 (38), 215 (76), 214 (100), 213 (23), 179 (23), 178 (39), 177 (15), 152 (30), 151 (14).

2-(3'-Fluoro-[1,1'-biphenyl]-4-yl)-4,4,5,5-tetramethyl-1,3,2-dioxaborolane

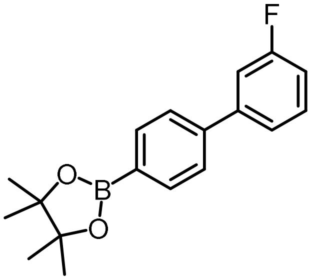

The cross-coupling was performed according to GP 1 by using triethyl(4-(4,4,5,5-tetramethyl-1,3,2-dioxaborolan-2-yl)phenyl)germane (108.9 mg, 0.300 mmol, 1.0 equiv.) and 3-fluorobenzenediazonium tetrafluoroborate (94.5 mg, 0.450 mmol, 1.5 equiv.). Reaction time: 2 h. The title product was obtained after purification by preparative TLC (20:1 *n*-pentane/EtOAc) as a white solid (71.5 mg, 0.240 mmol, 80%).

R$_f$ = 0.70 (20:1 *n*-pentane/EtOAc). **^{1}H NMR** (600 MHz, $CDCl_3$) δ/ppm = 7.89 (d, *J* = 8.2 Hz, 2H), 7.59 (d, *J* = 8.2 Hz, 2H), 7.42-7.38 (m, 2H), 7.33-7.29 (m, 1H), 7.08-7.02 (m, 1H), 1.37 (s, 12H). **^{13}C NMR** (151 MHz, $CDCl_3$) δ/ppm = 163.3 (d, *J* = 245.3 Hz), 143.4 (d, *J* = 7.5 Hz), 142.7 (d,

J = 2.0 Hz), 135.5, 130.4 (d, *J* = 8.4 Hz), 126.5, 123.0 (d, *J* = 2.7 Hz), 114.5 (d, *J* = 21.2 Hz), 114.2 (d, *J* = 22.0 Hz), 84.1, 25.0 (carbon atom attached to boron atom is not observed). **IR** (neat): *ν*/cm^{-1} = 2979, 2929, 1610, 1587, 1552, 1524, 1480, 1442, 1394, 1358, 1325, 1270, 1213, 1183, 1141, 1090, 1017, 961, 858, 829, 781, 744, 691, 657. **HRMS** (APCI) calculated for $C_{18}H_{21}BFO_2$: 299.1613 $[M+H]^+$, found: 299.1611.

Ethyl 4'-(4,4,5,5-tetramethyl-1,3,2-dioxaborolan-2-yl)-[1,1'-biphenyl]-3-carboxylate

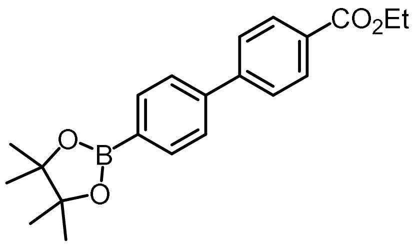

The cross-coupling was performed according to GP 1 by using triethyl(4-(4,4,5,5-tetramethyl-1,3,2-dioxaborolan-2-yl)phenyl)germane (108.9 mg, 0.300 mmol, 1.0 equiv.) and 4-(ethoxycarbonyl)benzenediazonium tetrafluoroborate (118.8 mg, 0.450 mmol, 1.5 equiv.). Reaction time: 2 h. The title product was obtained after purification by preparative TLC (20:1 *n*-hexane/EtOAc) as a white solid (61.3 mg, 0.174 mmol, 58% [71% according to NMR]).

R$_f$ = 0.56 (20:1 *n*-hexane/EtOAc). **^{1}H NMR** (600 MHz, $CDCl_3$) δ/ppm = 8.11 (d, *J* = 8.4 Hz, 2H), 7.90 (d, *J* = 8.2 Hz, 2H), 7.68 (d, *J* = 8.4 Hz, 2H), 7.63 (d, *J* = 8.2 Hz, 2H), 4.40 (q, *J* = 7.1 Hz, 2H), 1.42 (t, *J* = 7.1 Hz, 3H), 1.37 (s, 12H). **^{13}C NMR** (151 MHz, $CDCl_3$) δ/ppm = 166.6, 145.5, 142.8, 135.5, 130.2, 129.6, 127.3, 126.7, 84.1, 61.2, 25.0, 14.5 (carbon atom attached to boron atom is not observed). **IR** (neat): *ν*/cm^{-1} = 2979, 2924, 2854, 1717, 1606, 1526, 1463, 1391, 1358, 1270, 1212, 1173, 1140, 1097, 1024, 962, 857, 828, 772, 739, 702, 657. **HRMS** (ESI) calculated for $C_{19}H_{27}O_4BNa$: 353.1919 $[M+Na]^+$, found: 353.1925.

1-(4'-(4,4,5,5-Tetramethyl-1,3,2-dioxaborolan-2-yl)-[1,1'-biphenyl]-4-yl)ethan-1-one

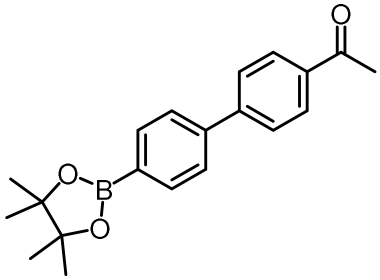

The cross-coupling was performed according to GP 1 by using triethyl(4-(4,4,5,5-tetramethyl-1,3,2-dioxaborolan-2-yl)phenyl)germane (108.9 mg, 0.300 mmol, 1.0 equiv.) and 4-acetylbenzenediazonium tetrafluoroborate (105.3 mg, 0.450 mmol, 1.5 equiv.). Reaction time: 2 h. The title product was obtained after purification by preparative TLC (20:1 *n*-hexane/EtOAc) as a white solid (59.0 mg, 0.183 mmol, 61% [79% according to NMR]).

R$_f$ = 0.31 (20:1 *n*-hexane/EtOAc). **^{1}H NMR** (600 MHz, $CDCl_3$) δ/ppm = 8.03 (d, *J* = 8.4 Hz, 2H), 7.91 (d, *J* = 8.2 Hz, 2H), 7.71 (d, *J* = 8.4 Hz, 2H), 7.64 (d, *J* = 8.2 Hz, 2H), 2.64 (s, 3H), 1.37 (s, 12H). **^{13}C NMR** (151 MHz, $CDCl_3$) δ/ppm = 197.9, 145.7, 142.6, 136.2, 135.5, 129.1, 127.5,

126.7, 84.1, 26.8, 25.0 (carbon atom attached to boron atom is not observed). **HRMS** (APCI) calculated for $C_{20}H_{24}BO_3$: 323.1813 $[M+H]^+$, found: 323.1829.

4,4,5,5-Tetramethyl-2-(4'-nitro-[1,1'-biphenyl]-4-yl)-1,3,2-dioxaborolane

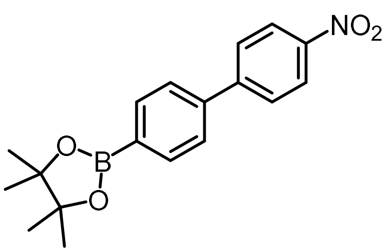

The cross-coupling was performed according to GP 1 by using triethyl(4-(4,4,5,5-tetramethyl-1,3,2-dioxaborolan-2-yl)phenyl)germane (108.9 mg, 0.300 mmol, 1.0 equiv.) and 4-nitrobenzenediazonium tetrafluoroborate (106.6 mg, 0.450 mmol, 1.5 equiv.). Reaction time: 2 h. The title product was obtained after purification by preparative TLC (20:1 *n*-hexane/EtOAc) as a yellow solid (61.5 mg, 0.189 mmol, 63% [80% according to NMR]).

R_f = 0.35 (20:1 *n*-hexane/EtOAc). **^{1}H NMR** (600 MHz, $CDCl_3$) δ/ppm = 8.30 (d, *J* = 8.8 Hz, 2H), 7.93 (d, *J* = 8.2 Hz, 2H), 7.76 (d, *J* = 8.8 Hz, 2H), 7.63 (d, *J* = 8.2 Hz, 2H), 1.37 (s, 12H). **^{13}C NMR** (151 MHz, $CDCl_3$) δ/ppm = 147.6, 147.4, 141.4, 135.7, 128.1, 126.8, 124.3, 84.2, 25.0 (carbon atom attached to boron atom is not observed). **IR** (neat): ν/cm^{-1} = 3359, 2921, 2852, 1715, 1658, 1594, 1553, 1512, 1392, 1333, 1262, 1141, 1092, 1021, 960, 857, 820, 733, 675. **HRMS** (APCI) calculated for $C_{18}H_{21}BNO_4$: 326.1558 $[M+H]^+$, found 326.1564.

3'-Fluoro-3,5-bis(trifluoromethyl)-1,1'-biphenyl

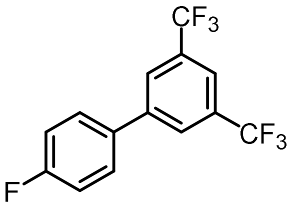

The cross-coupling was performed according to GP 1 by using triethyl(4-fluorophenyl)germane (76.5 mg, 0.300 mmol, 1.0 equiv.) and 3,5-bis(trifluoromethyl)benzenediazonium tetrafluoroborate (147.6 mg, 0.450 mmol, 1.5 equiv.). Reaction time: 7 h. The title product was obtained after purification by column chromatography (*n*-pentane) as a colorless oil (56.4 mg, 0.183 mmol, 61%).

R_f = 0.75 (*n*-pentane). **^{1}H NMR** (600 MHz, $CDCl_3$) δ/ppm = 7.97 (s, 2H), 7.86 (s, 1H), 7.63-7.55 (m, 2H), 7.23-7.17 (m, 2H). **^{13}C NMR** (151 MHz, $CDCl_3$) δ/ppm = 163.5 (d, *J* = 249.3 Hz), 142.5, 134.5 (d, *J* = 3.2 Hz), 132.4 (q, *J* = 33.3 Hz), 129.2 (d, *J* = 8.5 Hz), 127.2 (d, *J* = 4.0 Hz), 123.5 (q, *J* = 272.8 Hz), 121.1 (hept, *J* = 3.7 Hz), 116.5 (d, *J* = 22.1 Hz). **^{19}F NMR** (564 MHz, $CDCl_3$) δ/ppm = -62.92 (s, 6F), -112.81 – -112.87 (m, 1F). **HRMS** (APCI) calculated for $C_{14}H_7F_7$: 308.0430 $[M]^+$, found 308.0422. These data are in agreement with those reported previously in the literature.[7]

2-(Allyloxy)-4'-nitro-1,1'-biphenyl

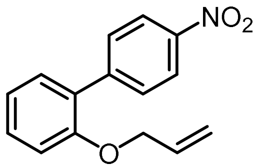

The cross-coupling was performed according to GP 1 by using (2-(allyloxy)phenyl)triethylgermane (87.9 mg, 0.300 mmol, 1.0 equiv.) and 4-nitrobenzenediazonium tetrafluoroborate (106.6 mg, 0.450 mmol, 1.5 equiv.). Reaction time: 2 h. The title product was obtained after purification by column chromatography (20:1 *n*-hexane/EtOAc) as a brown oil (48.3 mg, 0.189 mmol, 63%).

R_f = 0.59 (20:1 *n*-hexane/EtOAc). **^{1}H NMR** (600 MHz, $CDCl_3$) δ/ppm = 8.26 (d, *J* = 8.8 Hz, 2H), 7.73 (d, *J* = 8.8 Hz, 2H), 7.41-7.33 (m, 2H), 7.11-7.05 (m, 1H), 7.01 (d, *J* = 8.3 Hz, 1H), 6.04-5.93 (m, 1H), 5.32 (dd, *J* = 17.2, 1.6 Hz, 1H), 5.24 (dd, *J* = 10.7, 1.6 Hz, 1H), 4.57 (d, *J* = 4.9 Hz, 2H). **^{13}C NMR** (151 MHz, $CDCl_3$) δ/ppm = 155.5, 146.8, 145.6, 132.9, 130.9, 130.5, 130.2, 128.7, 123.3, 121.4, 117.5, 113.0, 69.2. **IR** (neat): ν/cm^{-1} = 3075, 3026, 2921, 2857, 2325, 2085, 1925, 1597, 1511, 1480, 1449, 1403, 1343, 1263, 1229, 1163, 1110, 1054, 996, 928, 855, 750, 697. **HRMS** (ESI) calculated for $C_{15}H_{13}O_3NNa$: 278.0788 $[M+Na]^+$, found: 278.0788.

2-Methyl-4'-nitro-1,1'-biphenyl

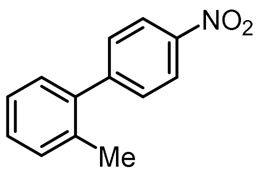

The cross-coupling was performed according to GP 1 by using triethyl(*o*-tolyl)germane (75.3 mg, 0.300 mmol, 1.0 equiv.) and 4-nitrobenzenediazonium tetrafluoroborate (106.6 mg, 0.450 mmol, 1.5 equiv.). Reaction time: 2 h. The title product was obtained after purification by column chromatography (20:1 *n*-hexane/EtOAc) as a pale yellow solid (60.8 mg, 0.285 mmol, 95%).

R_f = 0.74 (20:1 *n*-hexane/EtOAc). **^{1}H NMR** (600 MHz, $CDCl_3$) δ/ppm = 8.29 (d, *J* = 8.7 Hz, 2H), 7.50 (d, *J* = 8.7 Hz, 2H), 7.40-7.20 (m, 4H), 2.29 (s, 3H). **^{13}C NMR** (151 MHz, $CDCl_3$) δ/ppm = 148.9, 146.9, 139.7, 135.2, 130.8, 130.2, 129.5, 128.6, 126.2, 123.5, 20.5. **HRMS** (ESI) calculated for $C_{13}H_{11}O_2NNa$: 236.0682 $[M+Na]^+$, found: 236.0682. These data are in agreement with those reported previously in the literature.[8]

4-Bromo-4'-nitro-2-(trifluoromethoxy)-1,1'-biphenyl

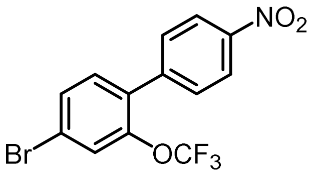

The cross-coupling was performed according to GP 1 by using (4-bromo-2-(trifluoromethoxy)phenyl)triethylgermane (119.9 mg, 0.300 mmole, 1.0 equiv.) and 4-nitrobenzenediazonium tetrafluoroborate (106.6 mg, 0.450 mmol, 1.5 equiv.). Reaction

time: 2 h. The title product was obtained after purification by column chromatography (20:1 *n*-hexane/EtOAc) as a white solid (97.7 mg, 0.270 mmol, 90%).

R$_f$ = 0.69 (20:1 *n*-hexane/EtOAc). **^{1}H NMR** (600 MHz, $CDCl_3$) δ/ppm = 8.30 (d, *J* = 8.8 Hz, 2H), 7.61 (d, *J* = 8.8 Hz, 2H), 7.59-7.55 (m, 2H), 7.34 (d, *J* = 8.2 Hz, 1H). **^{13}C NMR** (151 MHz, $CDCl_3$) δ/ppm = 147.6, 146.3, 142.4, 132.3, 132.2, 130.9, 130.2, 125.1, 123.8, 123.2, 120.3 (q, *J* = 259.7 Hz). **^{19}F NMR** (564 MHz, $CDCl_3$) δ/ppm = -57.42 (s, 3F). **IR** (neat): ν/cm^{-1} = 3087, 1597, 1516, 1476, 1388, 1345, 1267, 1165, 1006, 935, 854, 818, 751, 724, 691. **HRMS** (ESI) calculated for $C_{13}H_7O_3N^{79}BrF_3Na$: 383.9454 [M+Na]$^+$, found: 383.9454.

4-Iodo-4'-nitro-1,1'-biphenyl

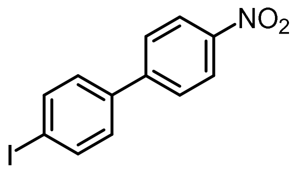

The cross-coupling was performed according to GP 1 by using triethyl(4-iodophenyl)germane (108.8 mg, 0.300 mmol, 1.0 equiv.) and 4-nitrobenzenediazonium tetrafluoroborate (106.6 mg, 0.450 mmol, 1.5 equiv.). Reaction time: 2 h. The title product was obtained after purification by column chromatography (20:1 *n*-hexane/EtOAc) as a yellow solid (67.3 mg, 0.207 mmol, 69%).

R$_f$ = 0.67 (20:1 *n*-hexane/EtOAc). **^{1}H NMR** (600 MHz, $CDCl_3$) δ/ppm = 8.30 (d, *J* = 8.8 Hz, 2H), 7.83 (d, *J* = 8.4 Hz, 2H), 7.70 (d, *J* = 8.8 Hz, 2H), 7.36 (d, *J* = 8.4 Hz, 2H). **^{13}C NMR** (151 MHz, $CDCl_3$) δ/ppm = 147.4, 146.6, 138.5, 138.4, 129.2, 127.8, 124.4, 95.3. **HRMS** (APCI) calculated for $C_{12}H_9INO_2$: 325.9672 [M+H]$^+$, found: 325.9664. These data are in agreement with those reported previously in the literature.[9]

4-Bromo-4'-iodo-1,1'-biphenyl

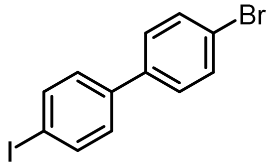

The cross-coupling was performed according to GP 1 by using triethyl(4-iodophenyl)germane (108.8 mg, 0.300 mmol, 1.0 equiv.) and 4-bromobenzenediazonium tetrafluoroborate (121.9 mg, 0.450 mmol, 1.5 equiv.). Reaction time: 2 h. The title product was obtained after purification by column chromatography (*n*-pentane) as a white solid (87.2 mg, 0.243 mmol, 81%).

R$_f$ = 0.63 (*n*-pentane). **^{1}H NMR** (600 MHz, $CDCl_3$) δ/ppm = 7.76 (d, *J* = 8.5 Hz, 2H), 7.56 (d, *J* = 8.5 Hz, 2H), 7.41 (d, *J* = 8.5 Hz, 2H), 7.28 (d, *J* = 8.5 Hz, 2H). **^{13}C NMR** (151 MHz, $CDCl_3$) δ/ppm = 139.6, 139.1, 138.1, 132.2, 128.9, 128.6, 122.1, 93.6. **HRMS** (APCI) calculated for $C_{12}H_8{}^{79}BrI$: 357.8849 [M]$^+$, found: 357.8838. These data are in agreement with those reported previously in the literature.[10]

(4'-Bromo-[1,1'-biphenyl]-4-yl)trimethylsilane

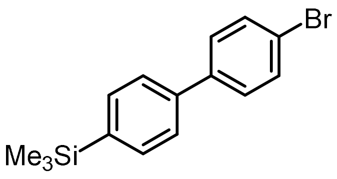

The cross-coupling was performed according to GP 1 by using trimethyl(4-(triethylgermyl)phenyl)silane (92.7 mg, 0.300 mmol, 1.0 equiv.) and 4-bromobenzenediazonium tetrafluoroborate (97.5 mg, 0.360 mmol, 1.2 equiv.). Reaction time: 2 h. The title product was obtained after purification by column chromatography (*n*-pentane) as a white solid (73.3 mg, 0.240 mmol, 80%).

R$_f$ = 0.67 (*n*-pentane). **^{1}H NMR** (600 MHz, $CDCl_3$) δ/ppm = 7.67-7.61 (m, 2H), 7.60-7.54 (m, 4H), 7.49-7.46 (m, 2H), 0.33 (s, 9H). **^{13}C NMR** (151 MHz, $CDCl_3$) δ/ppm = 140.5, 140.2, 140.0, 134.1, 132.0, 128.9, 126.4, 121.8, -1.0. **HRMS** (EI) calculated for $C_{15}H_{17}{}^{79}BrSi$: 304.0277 $[M]^+$, found: 304.0284. These data are in agreement with those reported previously in the literature.[11]

(5-Bromo-4'-nitro-[1,1'-biphenyl]-3-yl)trimethylsilane

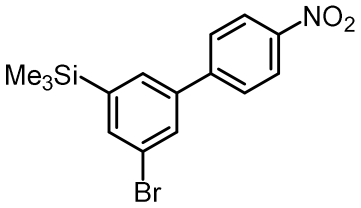

The cross-coupling was performed according to GP 1 by using (3-bromo-5-(triethylgermyl)phenyl)trimethylsilane (582.0 mg, 1.50 mmol, 1.0 equiv.) and 4-nitrobenzenediazonium tetrafluoroborate (426.5 mg, 1.80 mmol, 1.2 equiv.). Reaction time: 12 h. The title product was obtained after purification by column chromatography (20:1 *n*-pentane/EtOAc) as a pale yellow solid (428.5 mg, 1.22 mmol, 82%).

R$_f$ = 0.60 (20:1 *n*-pentane/EtOAc). **^{1}H NMR** (400 MHz, $CDCl_3$) δ/ppm = 8.31 (d, *J* = 8.7 Hz, 2H), 7.75-7.66 (m, 4H), 7.63-7.59 (m, 1H), 0.33 (s, 9H). **^{13}C NMR** (101 MHz, $CDCl_3$) δ/ppm = 147.6, 146.6, 145.0, 140.5, 136.4, 130.8, 130.7, 128.2, 124.3, 123.7, -1.1. **IR** (neat): ν/cm^{-1} = 2956, 1791, 1739, 1596, 1548, 1512, 1402, 1345, 1279, 1248, 1136, 1108, 1053, 1012, 989, 835, 793, 746, 690. **HRMS** (GC-APCI) calculated for $C_{15}H_{16}{}^{79}BrNO_2Si$: 349.0128 $[M]^+$, found: 349.0168.

4-Bromo-4'-nitro-1,1'-biphenyl

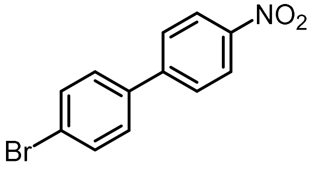

The cross-coupling was performed according to GP 1 by using (4-bromophenyl)triethylgermane (94.7 mg, 0.300 mmol, 1.0 equiv.) and 4-nitrobenzenediazonium tetrafluoroborate (106.6 mg, 0.450 mmol, 1.5 equiv.). Reaction time: 2 h. The title product was

obtained after filtration and washing with a minimal volume of cold MeCN as a pale yellow solid (77.5 mg, 0.279 mmol, 93%).

^{1}H NMR (400 MHz, $CDCl_3$) δ/ppm = 8.30 (d, *J* = 8.9 Hz, 2H), 7.71 (d, *J* = 8.9 Hz, 2H), 7.63 (d, *J* = 8.5 Hz, 2H), 7.49 (d, *J* = 8.5 Hz, 2H). **^{13}C NMR** (151 MHz, $CDCl_3$) δ/ppm = 147.5, 146.5, 137.8, 132.5, 129.1, 127.8, 124.4, 123.7. **HRMS** (GC-APCI) calculated for $C_{12}H_8{}^{79}BrNO_2$: 276.9733 $[M]^+$, found 276.9769. These data are in agreement with those reported previously in the literature.[12]

4-Chloro-4'-(trifluoromethyl)-1,1'-biphenyl

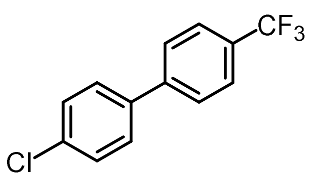

The cross-coupling was performed according to GP 1 by using (4-chlorophenyl)triethylgermane (81.4 mg, 0.300 mmol, 1.0 equiv.) and (4-trifluoromethyl)benzenediazonium tetrafluoroborate (117.0 mg, 0.450 mmol, 1.5 equiv.). Reaction time: 7 h. The title product was obtained after purification by column chromatography (*n*-pentane) as a white solid (66.2 mg, 0.258 mmol, 86%).

R_f = 0.65 (*n*-pentane). **^{1}H NMR** (600 MHz, $CDCl_3$) δ/ppm = 7.70 (d, *J* = 8.4 Hz, 2H), 7.66 (d, *J* = 8.4 Hz, 2H), 7.53 (d, *J* = 8.5 Hz, 2H), 7.45 (d, *J* = 8.5 Hz, 2H). **^{13}C NMR** (151 MHz, $CDCl_3$) δ/ppm = 143.6, 138.4, 134.6, 129.9 (q, *J* = 32.8 Hz), 129.3, 128.7, 127.4, 126.1-125.9 (m), 124.4 (q, *J* = 272.5 Hz). **^{19}F NMR** (565 MHz, $CDCl_3$) δ/ppm = -62.50 (s, 3F). **HRMS** (APCI) calculated for $C_{13}H_8{}^{35}ClF_3$: 256.0261 $[M]^+$, found: 256.0259. These data are in agreement with those reported previously in the literature.[13]

1-(4'-(Triethylsilyl)-[1,1'-biphenyl]-4-yl)ethan-1-one

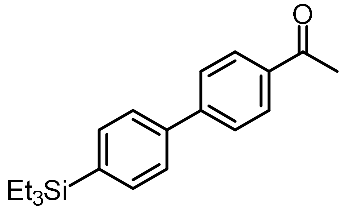

The cross-coupling was performed according to GP 1 by using triethyl(4-(triethylgermyl)phenyl)silane (105.4 mg, 0.300 mmol, 1.0 equiv.) and 4-acetylbenzenediazonium tetrafluoroborate (105.3 mg, 0.450 mmol, 1.5 equiv.). Reaction time: 2 h. The title product was obtained after purification by column chromatography (20:1 *n*-hexane/EtOAc) as a white solid (69.5 mg, 0.224 mmol, 75%).

R_f = 0.23 (20:1 *n*-hexane/EtOAc). **^{1}H NMR** (400 MHz, $CDCl_3$) δ/ppm = 8.04 (d, *J* = 8.4 Hz, 2H), 7.71 (d, *J* = 8.4 Hz, 2H), 7.65-7.56 (m, 4H), 2.64 (s, 3H), 1.05-0.94 (m, 9H), 0.89-0.77 (m, 6H). **^{13}C NMR** (101 MHz, $CDCl_3$) δ/ppm = 197.9, 145.9, 140.1, 137.9, 136.0, 135.0, 129.1, 127.3, 126.6, 26.8, 7.6, 3.5. **IR** (neat): ν/cm^{-1} = 2952, 2909, 2875, 2325, 1920, 1809, 1675, 1600,

1457, 1411, 1375, 1352, 1269, 1234, 1187, 1110, 999, 956, 855, 808, 721, 669. **HRMS** (ESI) calculated for $C_{20}H_{27}OSi$: 311.1826 [M+H]$^+$, found: 311.1825.

4-Chloro-4'-nitro-1,1'-biphenyl

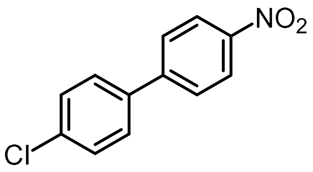

The cross-coupling was performed according to GP 1 by using (4-chlorophenyl)triethylgermane (81.4 mg, 0.300 mmol, 1.0 equiv.) and 4-nitrobenzenediazonium tetrafluoroborate (106.6 mg, 0.450 mmol, 1.5 equiv.). Reaction time: 2 h. The title product was obtained after purification by column chromatography (20:1 *n*-hexane/EtOAc) as a pale yellow solid (63.1 mg, 0.270 mmol, 90%).

R$_f$ = 0.32 (20:1 *n*-hexane/EtOAc). **^{1}H NMR** (600 MHz, $CDCl_3$) δ/ppm = 8.30 (d, *J* = 8.7 Hz, 2H), 7.71 (d, *J* = 8.7 Hz, 2H), 7.56 (d, *J* = 8.5 Hz, 2H), 7.47 (d, *J* = 8.5 Hz, 2H). **^{13}C NMR** (151 MHz, $CDCl_3$) δ/ppm = 147.4, 146.5, 137.3, 135.4, 129.5, 128.8, 127.8, 124.4. **HRMS** (ESI) calculated for $C_{12}H_9{}^{35}ClNO_2$: 234.0316 [M+H]$^+$, found: 234.0315. These data are in agreement with those reported previously in the literature.[12]

4-Chloro-3-fluoro-4'-iodo-1,1'-biphenyl

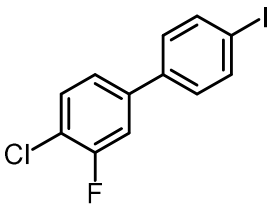

The cross-coupling was performed according to GP 1 by using (4-chloro-3-fluorophenyl)triethylgermane (86.8 mg, 0.300 mmol, 1.0 equiv.) and 4-iodobenzenediazonium tetrafluoroborate (143.0 mg, 0.450 mmol, 1.5 equiv.). Reaction time: 2 h. The title product was obtained after purification by column chromatography (*n*-pentane) and preparative HPLC (97:3 *n*-hexane/EtOAc) as a white solid (57.1 mg, 0.172 mmol, 57%).

R$_f$ = 0.48 (*n*-pentane). **^{1}H NMR** (600 MHz, $CDCl_3$) δ/ppm = 7.78 (d, *J* = 8.4 Hz, 2H), 7.49-7.41 (m, 1H), 7.33 (dd, *J* = 10.2, 2.1 Hz, 1H), 7.27 (m, 3H). **^{13}C NMR** (151 MHz, $CDCl_3$) δ/ppm = 158.5 (d, *J* = 248.9 Hz), 140.8 (d, *J* = 6.8 Hz), 138.6 (d, *J* = 2.2 Hz), 138.3, 131.2, 128.8, 123.3 (d, *J* = 3.6 Hz), 120.6 (d, *J* = 17.6 Hz), 115.1 (d, *J* = 21.8 Hz), 94.2. **^{19}F NMR** (565 MHz, $CDCl_3$) δ/ppm = -114.73 – -114.86 (m, 1F). **IR** (neat): ν/cm^{-1} = 2922, 2853, 2676, 2322, 2099, 1998, 1911, 1741, 1576, 1551, 1501, 1468, 1418, 1380, 1301, 1248, 1200, 1147, 1106, 1066, 996, 873, 806, 722, 672. **HRMS** (APCI) calculated for $C_{12}H_7{}^{35}ClFI$: 331.9260 [M]$^+$, found: 331.9257.

(5-Iodo-4'-nitro-[1,1'-biphenyl]-3-yl)trimethylsilane

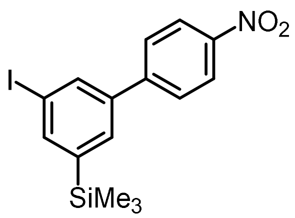

The cross-coupling was performed according to GP 1 by using (3-iodo-5-(triethylgermyl)phenyl)trimethylsilane (130.5 mg, 0.300 mmol, 1.0 equiv.) and 4-nitrobenzenediazonium tetrafluoroborate (85.3 mg, 0.360 mmol, 1.2 equiv.). Reaction time: 2 h. The title product was obtained after purification by column chromatography (30:1 *n*-pentane/EtOAc) and preparative HPLC (95:5 *n*-hexane/EtOAc) as a pale yellow solid (59.4 mg, 0.150 mmol, 50%).

R_f = 0.32 (30:1 *n*-pentane/EtOAc). **^{1}H NMR** (600 MHz, $CDCl_3$) δ/ppm = 8.30 (d, *J* = 8.8 Hz, 2H), 7.94-7.91 (m, 1H), 7.88 (s, 1H), 7.70 (d, *J* = 8.8 Hz, 2H), 7.65-7.64 (m, 1H), 0.32 (s, 9H). **^{13}C NMR** (151 MHz, $CDCl_3$) δ/ppm = 147.6, 146.5, 145.2, 142.4, 140.6, 136.7, 131.3, 128.1, 124.3, 96.1, -1.1. **IR** (neat): ν/cm^{-1} = 2951, 2325, 2085, 1730, 1595, 1543, 1513, 1404, 1383, 1341, 1247, 1193, 1137, 1106, 1044, 992, 834, 790, 745, 682. **HRMS** (ESI) calculated for $C_{15}H_{16}INNaO_2Si$: 419.9887 [M+Na]$^+$, found: 419.9888.

Trimethyl(5-(4,4,5,5-tetramethyl-1,3,2-dioxaborolan-2-yl)-4'-(trifluoromethyl)-[1,1'-biphenyl]-3-yl)silane

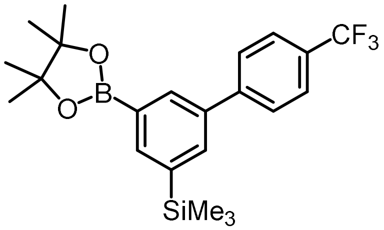

The cross-coupling was performed according to GP 1 by using trimethyl(3-(4,4,5,5-tetramethyl-1,3,2-dioxaborolan-2-yl)-5-(triethylgermyl)phenyl)silane (130.5 mg, 0.300 mmol, 1.0 equiv.) and (4-trifluoromethyl)benzenediazonium tetrafluoroborate (93.6 mg, 0.360 mmol, 1.2 equiv.). Reaction time: 7 h. The title product was obtained after purification by column chromatography (3:1 *n*-pentane/Et_2O) as a white solid (108.0 mg, 0.260 mmol, 86%).

R_f = 0.65 (3:1 *n*-pentane/Et_2O). **^{1}H NMR** (400 MHz, $CDCl_3$) δ/ppm = 8.10-7.95 (m, 2H), 7.87-7.59 (m, 5H), 1.37 (s, 12H), 0.33 (s, 9H). **^{13}C NMR** (101 MHz, $CDCl_3$) δ/ppm = 145.3, 140.8, 139.6, 138.5, 135.2, 134.4, 129.3 (q, *J* = 32.6 Hz), 127.8, 125.9-125.1 (m), 124.4 (q, *J* = 271.9 Hz), 84.1, 25.0, -0.9 (carbon atom attached to boron atom is not observed). **^{19}F NMR** (376 MHz, $CDCl_3$) δ/ppm = -62.35 (s, 3F). **IR** (neat): ν/cm^{-1} = 2966, 1589, 1443, 1398, 1361, 1321, 1256, 1115, 1071, 1017, 961, 839, 756, 706. **HRMS** (GC-APCI) calculated for $C_{21}H_{26}BF_3O_2Si$: 406.1742 [M-Me]$^+$, found: 406.1729.

2.2 Au-catalyzed Cross-coupling of Aryl Germanes with *e*-Rich ArN_2BF_4

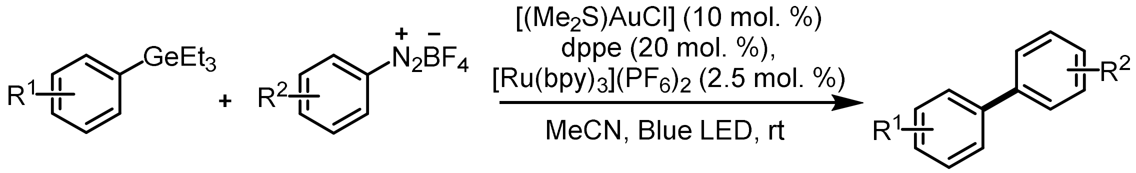

General Procedure 2 (GP 2)

In an argon-filled glovebox aryl triethylgermane (1.0 equiv.), diazonium salt (2.0 equiv.), $[(Me_2S)AuCl]$ (10 mol. %), dppe (20 mol. %), and $[Ru(bpy)_3](PF_6)_2$ (2.5 mol. %) were mixed in a screw top vial equipped with magnetic stirring bar and dissolved in anhydrous and degassed MeCN (0.1 M). Then the vial was placed into the blue LED setup. After full consumption of germane (determined by GC-MS) a crude mixture was concentrated *in vacuo* and purified by silica gel column chromatography. The reaction time is specified for the individual compounds.

4-Fluoro-4'-methoxy-1,1'-biphenyl

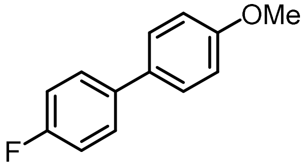

The cross-coupling was performed according to GP 2 by using triethyl(4-fluorophenyl)germane (76.5 mg, 0.300 mmol, 1.0 equiv.) and 4-methoxybenzenediazonium tetrafluoroborate (133.2 mg, 0.600 mmol, 2.0 equiv.). Reaction time: 2 h. The title product was obtained after purification by column chromatography (40:1 *n*-pentane/EtOAc) as a white solid (41.0 mg, 0.203 mmol, 68%).

R_f = 0.40 (40:1 *n*-pentane/EtOAc). **^{1}H NMR** (600 MHz, $CDCl_3$) δ/ppm = 7.52-7.45 (m, 4H), 7.12-7.08 (m, 2H), 6.97 (d, *J* = 8.7 Hz, 2H), 3.85 (s, 3H). **^{13}C NMR** (151 MHz, $CDCl_3$) δ/ppm = 162.3 (d, *J* = 245.7 Hz), 159.3, 137.1 (d, *J* = 3.1 Hz), 133.0, 128.4 (d, *J* = 8.0 Hz), 128.2, 115.7 (d, *J* = 21.3 Hz), 114.4, 55.5. **^{19}F NMR** (565 MHz, CDCl3) δ/ppm = -116.71 – -116.80 (m, 1F). **HRMS** (APCI) calculated for $C_{13}H_{11}FO$: 202.0788 $[M]^+$, found: 202.0788. These data are in agreement with those reported previously in the literature.[14]

4'-Methoxy-[1,1'-biphenyl]-4-carbonitrile

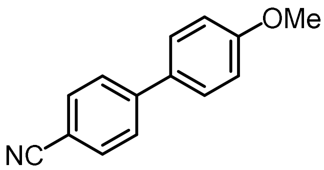

The cross-coupling was performed according to GP 2 by using 4-(triethylgermyl)benzonitrile (52.4 mg, 0.200 mmol, 1.0 equiv.) and 4-methoxybenzenediazonium tetrafluoroborate (88.8 mg, 0.400 mmol, 2.0 equiv.). Reaction time: 2 h. The title product was

obtained after purification by column chromatography (15:1 *n*-pentane/EtOAc) as a white solid (30.5 mg, 0.146 mmol, 73%).

R$_f$ = 0.40 (10:1 *n*-pentane/EtOAc). **^{1}H NMR** (400 MHz, $CDCl_3$) δ/ppm = 7.70 (d, *J* = 8.6 Hz, 2H), 7.64 (d, *J* = 8.6 Hz, 2H), 7.54 (d, *J* = 8.8 Hz, 2H), 7.01 (d, *J* = 8.8 Hz, 2H), 3.87 (s, 3H). **^{13}C NMR** (101 MHz, $CDCl_3$) δ/ppm = 160.4, 145.4, 132.7, 131.7, 128.5, 127.3, 119.3, 114.7, 110.3, 55.6. **HRMS** (ESI) calculated for $C_{14}H_{11}NNaO$: 232.0733 [M+Na]$^+$, found: 232.0730. These data are in agreement with those reported previously in the literature.[15]

4-Bromo-4'-methoxy-1,1'-biphenyl

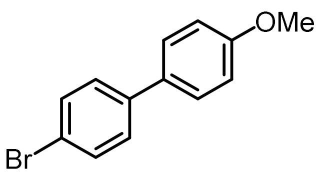

The cross-coupling was performed according to GP 2 by using (4-bromophenyl)triethylgermane (94.7 mg, 0.300 mmol, 1.0 equiv.) and 4-methoxybenzenediazonium tetrafluoroborate (133.2 mg, 0.600 mmol, 2.0 equiv.). Reaction time: 2 h. The title product was obtained after purification by column chromatography (50:1 *n*-pentane/EtOAc) as a white solid (55.2 mg, 0.210 mmol, 70%).

R$_f$ = 0.40 (50:1 *n*-pentane/EtOAc). **^{1}H NMR** (600 MHz, $CDCl_3$) δ/ppm = 7.53 (d, *J* = 8.5 Hz, 2H), 7.49 (d, *J* = 8.7 Hz, 2H), 7.41 (d, *J* = 8.5 Hz, 2H), 6.97 (d, *J* = 8.7 Hz, 2H), 3.85 (s, 3H). **^{13}C NMR** (151 MHz, $CDCl_3$) δ/ppm = 159.6, 139.9, 132.7, 131.9, 128.5, 128.1, 120.9, 114.5, 55.5. **HRMS** (APCI): calculated for $C_{13}H_{12}{}^{79}BrO$: 263.0066 [M+H]$^+$, found: 263.0058. These data are in agreement with those reported previously in the literature.[14]

4'-(*tert*-Butyl)-4-chloro-3-fluoro-1,1'-biphenyl

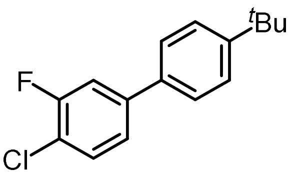

The cross-coupling was performed according to GP 2 by using (4-chloro-3-fluorophenyl)triethylgermane (86.8 mg, 0.300 mmol, 1.0 equiv.) and 4-(*tert*-butyl)benzenediazonium tetrafluoroborate (148.8 mg, 0.600 mmol, 2.0 equiv.). Reaction time: 2 h. The title product was obtained after purification by column chromatography (*n*-pentane) and preparative HPLC (95:5 *n*-hexane/EtOAc) as a white solid (66.2 mg, 0.252 mmol, 84%).

R$_f$ = 0.46 (*n*-pentane). **^{1}H NMR** (600 MHz, $CDCl_3$) δ/ppm = 7.51-7.46 (m, 4H), 7.43 (m, 1H), 7.37 (dd, *J* = 10.4, 2.1 Hz, 1H), 7.33-7.29 (m, 1H), 1.37 (s, 9H). **^{13}C NMR** (151 MHz, $CDCl_3$) δ/ppm = 158.5 (d, *J* = 248.2 Hz), 151.5, 141.9 (d, *J* = 6.8 Hz), 136.2 (d, *J* = 1.8 Hz), 130.9, 126.7, 126.1, 123.3 (d, *J* = 3.5 Hz), 119.7 (d, *J* = 17.8 Hz), 115.1 (d, *J* = 21.3 Hz), 34.8, 31.5. **^{19}F NMR** (565 MHz, $CDCl_3$) δ/ppm = -115.48 (dd, *J* = 10.0, 8.0 Hz, 1F). **IR** (neat): ν/cm^{-1} = 2964, 2933, 2870, 1606, 1576, 1557, 1476, 1421, 1391, 1366, 1308, 1266, 1248, 1200, 1145, 1110, 1070,

1028, 878, 839, 813, 739, 692. **HRMS** (APCI) calculated for $C_{16}H_{16}{}^{35}ClF$: 262.0919 $[M]^+$, found: 262.0919.

4-Bromo-*p*-terphenyl

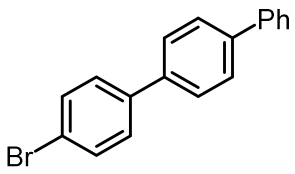

The cross-coupling was performed according to GP 2 by using (4-bromophenyl)triethylgermane (94.7 mg, 0.300 mmol, 1.0 equiv.) and 4-phenylbenzenediazonium tetrafluoroborate (160.8 mg, 0.600 mmol, 2.0 equiv.). Reaction time: 2 h. The title product was obtained after purification by column chromatography (*n*-pentane to 100:1 *n*-pentane/EtOAc) as a pale yellow solid (78.0 mg, 0.252 mmol, 79%).

$\mathbf{R_f}$ = 0.29 (*n*-pentane). **^{1}H NMR** (600 MHz, $CDCl_3$) δ/ppm = 7.68 (d, *J* = 8.4 Hz, 2H), 7.66-7.62 (m, 4H), 7.59 (d, *J* = 8.5 Hz, 2H), 7.51 (d, *J* = 8.5 Hz, 2H), 7.49-7.45 (m, 2H), 7.39-7.35 (m, 1H). **^{13}C NMR** (151 MHz, $CDCl_3$) δ/ppm = 140.7, 140.7, 139.8, 139.0, 132.1, 129.0, 128.8, 127.8, 127.6, 127.4, 127.2, 121.8. **HRMS** (APCI) calculated for $C_{18}H_{13}{}^{79}Br$: 308.0195 $[M]^+$, found 308.0195. These data are in agreement with those reported previously in the literature.[16]

4'-Methoxy-2-methyl-1,1'-biphenyl

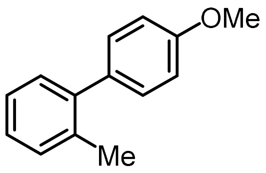

The cross-coupling was performed according to GP 2 by using triethyl(*o*-tolyl)germane (75.3 mg, 0.300 mmol, 1.0 equiv.) and 4-methoxybenzenediazonium tetrafluoroborate (133.2 mg, 0.600 mmol, 2.0 equiv.). Reaction time: 2 h. The title product was obtained after purification by column chromatography (40:1 *n*-pentane/EtOAc) and preparative HPLC (95:5 *n*-hexane/EtOAc) as a colorless oil (27.1 mg, 0.137 mmol, 46%).

$\mathbf{R_f}$ = 0.40 (40:1 *n*-pentane/EtOAc). **^{1}H NMR** (600 MHz, $CDCl_3$) δ/ppm = 7.30-7.23 (m, 6H), 6.98 (d, *J* = 8.7 Hz, 2H), 3.88 (s, 3H), 2.30 (s, 3H). **^{13}C NMR** (151 MHz, $CDCl_3$) δ/ppm = 158.7, 141.7, 135.6, 134.5, 130.4, 130.4, 130.1, 127.1, 125.9, 113.6, 55.4, 20.7. **HRMS** (ESI) calculated for $C_{14}H_{14}NaO$: 221.0937 $[M+Na]^+$, found 221.1007. These data are in agreement with those reported previously in the literature.[17]

3-(*p*-Tolyl)thiophene

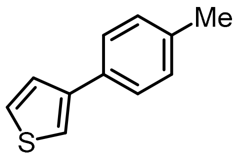

The cross-coupling was performed according to GP 2 by using triethyl(thiophen-3-yl)germane (72.9 mg, 0.300 mmole, 1.0 equiv.) and 4-methylbenzenediazonium tetrafluoroborate (123.6 mg, 0.600 mmol, 2.0 equiv.). Reaction time: 2 h. The title compound was obtained after purification by column chromatography (*n*-pentane) and preparative HPLC (97:3 *n*-hexane/EtOAc) as a white solid (26.7 mg, 0.153 mmol, 51%).

R$_f$ = 0.50 (*n*-pentane). **^{1}H NMR** (400 MHz, $CDCl_3$) δ/ppm = 7.50 (d, *J* = 8.2 Hz, 2H), 7.43-7.40 (m, 1H), 7.39-7.36 (m, 2H), 7.24-7.18 (m, 2H), 2.38 (s, 3H). **^{13}C NMR** (151 MHz, $CDCl_3$) δ/ppm = 142.5, 137.0, 133.3, 129.6, 126.5, 126.2, 119.8, 21.3. **HRMS** (ESI) calculated for $C_{11}H_{11}S$: 175.0576 $[M+H]^+$, found: 175.0573.

The resolution of the ^{13}C NMR does not allow for accurate assignment of all the signals.

(5-Bromo-4'-(*tert*-butyl)-[1,1'-biphenyl]-3-yl)trimethylsilane

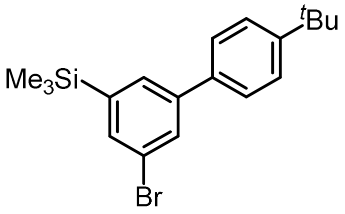

The cross-coupling was performed according to GP 2 by using (3-bromo-5-(triethylgermyl)phenyl)trimethylsilane (116.4 mg, 0.300 mmol, 1.0 equiv.) and 4-(*tert*-butyl)benzenediazonium tetrafluoroborate (148.8 mg, 0.600 mmol, 2.0 equiv.). Reaction time: 2 h. The title compound was obtained after purification by column chromatography (*n*-pentane) as a white solid (57.5 mg, 0.159 mmol, 53%).

R$_f$ = 0.59 (*n*-pentane). **^{1}H NMR** (600 MHz, $CDCl_3$) δ/ppm = 7.71-7.68 (m, 1H), 7.61-7.59 (m, 1H), 7.58-7.55 (m, 1H), 7.52-7.46 (m, 4H), 1.37 (s, 9H), 0.31 (s, 9H). **^{13}C NMR** (151 MHz, $CDCl_3$) δ/ppm = 151.0, 144.1, 142.9, 137.4, 134.5, 130.61, 130.57, 127.1, 126.0, 123.3, 34.7, 31.5, -1.1. **IR** (neat): ν/cm^{-1} = 3034, 2957, 2867, 2324, 2116, 1910, 1739, 1581, 1545, 1513, 1462, 1368, 1249, 1134, 1110, 1054, 1016, 833, 753, 692. **HRMS** (APCI) calculated for $C_{19}H_{25}{}^{79}BrSi$: 360.0903 $[M]^+$, found: 360.0913.

2-(4'-Methoxy-[1,1'-biphenyl]-4-yl)-4,4,5,5-tetramethyl-1,3,2-dioxaborolane

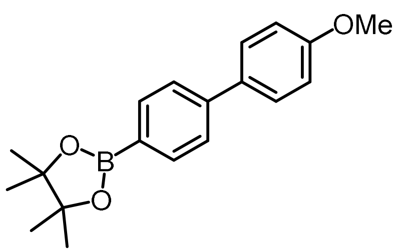

The cross-coupling was performed according to GP 2 by using triethyl(4-(4,4,5,5-tetramethyl-1,3,2-dioxaborolan-2-yl)phenyl)germane (108.9 mg, 0.300 mmol, 1.0 equiv.) and 4-methoxybenzenediazonium tetrafluoroborate (133.2 mg, 0.600 mmol, 2.0 equiv.). Reaction time: 2 h. The

title product was obtained after purification by column chromatography (DCM) as a white solid (55.8 mg, 0.180 mmol, 60%).

R_f = 0.68 (DCM). **^{1}H NMR** (600 MHz, $CDCl_3$) δ/ppm = 7.86 (d, *J* = 8.1 Hz, 2H), 7.59-7.54 (m, 4H), 6.98 (d, *J* = 8.7 Hz, 2H), 3.85 (s, 3H), 1.36 (s, 12H). **^{13}C NMR** (151 MHz, $CDCl_3$) δ/ppm = 159.5, 143.6, 135.4, 133.7, 128.4, 126.1, 114.4, 83.9, 55.5, 25.0 (carbon atom attached to boron atom is not observed). **HRMS** (ESI) calculated for $C_{19}H_{23}BNaO_3$: 333.1632 $[M+Na]^+$, found: 333.1634. These data are in agreement with those reported previously in the literature.[18]

4-Bromo-4'-(*tert*-butyl)-1,1'-biphenyl

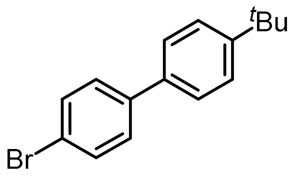

The cross-coupling was performed according to GP 2 by using (4-bromophenyl)triethylgermane (94.7 mg, 0.300 mmol, 1.0 equiv.) and 4-(*tert*-butyl)benzenediazonium tetrafluoroborate (148.8 mg, 0.600 mmol, 2.0 equiv.). Reaction time: 2 h. The title product was obtained after purification by column chromatography (*n*-pentane) as a white solid (65.1 mg, 0.225 mmol, 72%, purity 97%).

R_f = 0.63 (*n*-pentane). **^{1}H NMR** (600 MHz, $CDCl_3$) δ/ppm = 7.59-7.42 (m, 8H), 1.40 (s, 9H). **^{13}C NMR** (151 MHz, $CDCl_3$) δ/ppm = 150.9, 140.1, 137.2, 131.9, 128.7, 126.7, 126.0, 121.3, 34.7, 31.5. **HRMS** (APCI) calculated for $C_{16}H_{17}{}^{79}Br$: 288.0508 $[M]^+$, found: 288.0514. These data are in agreement with those reported previously in the literature.[19]

4-Methoxy-4'-(trifluoromethyl)-1,1'-biphenyl

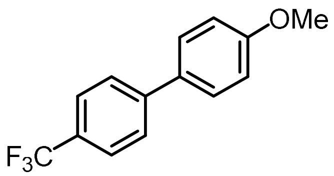

The cross-coupling was performed according to GP 2 by using triethyl(4-(trifluoromethyl)phenyl)germane (91.5 mg, 0.300 mmol, 1.0 equiv.) and 4-methoxybenzenediazonium tetrafluoroborate (133.2 mg, 0.600 mmol, 2.0 equiv.). Reaction time: 2 h. The title product was obtained after purification by column chromatography (20:1 *n*-hexane/EtOAc) as a white solid (56.0 mg, 0.222 mmol, 74%).

R_f = 0.84 (20:1 *n*-hexane/EtOAc). **^{1}H NMR** (600 MHz, $CDCl_3$) δ/ppm = 7.70-7.62 (m, 4H), 7.59-7.52 (m, 2H), 7.05-6.97 (m, 2H), 3.87 (s, 3H). **^{13}C NMR** (151 MHz, $CDCl_3$) δ/ppm = 160.0, 144.4, 132.3, 128.8 (d, *J* = 32.5 Hz), 128.5, 127.0, 125.8 (q, *J* = 3.8 Hz), 124.5 (q, *J* = 271.8 Hz), 114.6, 55.5. **^{19}F NMR** (564 MHz, $CDCl_3$) δ/ppm = -62.32 (s, 3F). **HRMS** (APCI) calculated for $C_{14}H_{11}F_3O$: 252.0757 $[M]^+$, found: 252.0762. These data are in agreement with those reported previously in the literature.[20]

4'-Fluoro-2-methoxy-1,1'-biphenyl

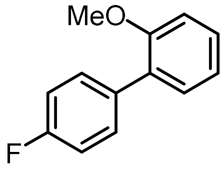

The cross-coupling was performed according to GP 2 by using triethyl(4-fluorophenyl)germane (76.5 mg, 0.300 mmol, 1.0 equiv.) and 2-methoxybenzenediazonium tetrafluoroborate (133.2 mg, 0.600 mmol, 2.0 equiv.). Reaction time: 2 h. The title product was obtained after purification by column chromatography (20:1 *n*-hexane/EtOAc) as a pale yellow oil (47.3 mg, 0.234 mmol, 78%).

R$_f$ = 0.87 (20:1 *n*-hexane/EtOAc). **^{1}H NMR** (600 MHz, $CDCl_3$) δ/ppm = 7.55-7.47 (m, 2H), 7.35 (ddd, *J* = 8.2, 7.4, 1.8 Hz, 1H), 7.32 (dd, *J* = 7.5, 1.8 Hz, 1H), 7.16-7.08 (m, 2H), 7.08-7.03 (m, 1H), 7.01 (dd, *J* = 8.3, 1.1 Hz, 1H), 3.84 (s, 3H). **^{13}C NMR** (151 MHz, $CDCl_3$) δ/ppm = 162.1 (d, *J* = 245.8 Hz), 156.5, 134.6 (d, *J* = 3.2 Hz), 131.3 (d, *J* = 8.0 Hz), 130.9, 129.8, 128.9, 121.0, 115.0 (d, *J* = 21.3 Hz), 111.4, 55.7. **^{19}F NMR** (564 MHz, $CDCl_3$) δ/ppm = -115.86 – -115.96 (m, 1F). **HRMS** (APCI) calculated for $C_{13}H_{12}FO$: 203.0867 $[M+H]^+$, found: 203.0867. These data are in agreement with those reported previously in the literature.[21]

4'-Fluoro-2-methyl-1,1'-biphenyl

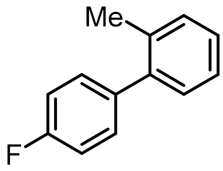

The cross-coupling was performed according to GP 2 by using triethyl(4-fluorophenyl)germane (76.5 mg, 0.300 mmol, 1.0 equiv.) and 2-methylbenzenediazonium tetrafluoroborate (123.6 mg, 0.600 mmol, 2.0 equiv.). Reaction time: 2 h. The title product was obtained after purification by column chromatography (*n*-pentane) as a pale yellow oil (46.9 mg, 0.252 mmol, 84%).

R$_f$ = 0.63 (*n*-pentane). **^{1}H NMR** (600 MHz, $CDCl_3$) δ/ppm = 7.34-7.21 (m, 6H), 7.16-7.10 (m, 2H), 2.29 (s, 3H). **^{13}C NMR** (151 MHz, $CDCl_3$) δ/ppm = 162.1 (d, *J* = 245.7 Hz), 141.1, 138.0 (d, *J* = 3.2 Hz), 135.5, 130.9 (d, *J* = 7.9 Hz), 130.5, 130.0, 127.6, 126.0, 115.1 (d, *J* = 21.2 Hz), 20.6. **^{19}F NMR** (564 MHz, $CDCl_3$) δ/ppm = -116.14 – -116.24 (m, 1F). **HRMS** (APCI) calculated for $C_{13}H_{12}FO$: 187.0918 $[M+H]^+$, found: 187.0919. These data are in agreement with those reported previously in the literature.[21]

2.3 Synthesis of Diazonium Salts

General Procedure 3 (GP 3)

Diazonium salts were synthesised according to literature known procedure.[22] The corresponding aniline (1.0 equiv.) was dissolved in EtOH (0.8 M) followed by addition of an aqueous solution of HBF_4 (48% in water, 2.0 equiv.). Then isoamyl nitrite (2.0 equiv.) was added dropwise at -20 °C. After the reaction mixture was allowed to warm up to room temperature and was stirred for 1 h. Et_2O was added to precipitate the corresponding diazonium salt followed by filtration and washing the solid with Et_2O (30 ml) or recrystallization from Et_2O/acetone mixture. The obtained salt was dried in high vacuum and stored in a freezer.

4-(Trifluoromethyl)benzenediazonium tetrafluoroborate

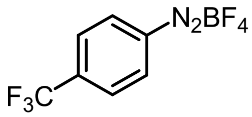

The title product was obtained by using (4-trifluoromethyl)aniline (1.21 g, 7.50 mmol, 1.0 equiv.) according to GP 3 as a white solid (1.50 g, 5.78 mmol, 77%).

^{1}H NMR (300 MHz, DMSO-d$_6$) δ/ppm = 8.90 (d, *J* = 8.7 Hz, 2H), 8.42 (d, *J* = 8.7 Hz, 2H). **^{13}C NMR** (151 MHz, DMSO-d$_6$) δ/ppm = 138.1 (q, *J* = 33.7 Hz), 133.8, 128.3 (q, *J* = 3.6 Hz), 122.3 (q, *J* = 274.1 Hz), 121.31. **^{19}F NMR** (282 MHz, DMSO-d$_6$) δ/ppm = -62.65 (s, 3F), -148.14 and -148.20 (s, BF_4). These data are in agreement with those reported previously in the literature.[23]

2-Bromo-4-chlorobenzenediazonium tetrafluoroborate

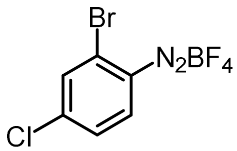

The title product was obtained by using 2-bromo-4-chloroaniline (825.9 mg, 4.00 mmol, 1.0 equiv.) according to GP 3 as a white solid (1.12 g, 3.68 mmol, 92%).

^{1}H NMR (400 MHz, DMSO-d$_6$) δ/ppm = 8.85 (d, *J* = 8.9 Hz, 1H), 8.62 (d, *J* = 2.0 Hz, 1H), 8.13 (dd, *J* = 8.9, 2.0 Hz, 1H). **^{13}C NMR** (101 MHz, DMSO-d$_6$) δ/ppm = 147.2, 135.9, 135.2, 130.9, 125.9, 117.6. **^{19}F NMR** (376 MHz, DMSO-d$_6$) δ/ppm = -148.23 and -148.28 (s, BF_4). These data are in agreement with those reported previously in the literature.[24]

3,5-Bis(trifluoromethyl)benzenediazonium tetrafluoroborate

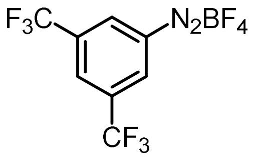

The title product was obtained by using 3,5-bis(trifluoromethyl)aniline (1.40 g, 6.10 mmol, 1.0 equiv.) according to GP 3 as a white solid (1.76 g, 5.38 mmol, 88%).

^{1}H NMR (400 MHz, DMSO-d$_6$) δ/ppm = 9.51 (s, 2H), 9.18 (s, 1H). **^{13}C NMR** (151 MHz, DMSO-d$_6$) δ/ppm = 134.8 (m), 134.0 (m), 132.0 (q, *J* = 35.8 Hz), 121.4 (q, *J* = 274.1 Hz), 120.1. **^{19}F NMR** (376 MHz, DMSO-d$_6$) δ/ppm = -61.84 (s, 6F), -148.27 and -148.32 (s, BF$_4$). These data are in agreement with those reported previously in the literature.[25]

4-(Ethoxycarbonyl)benzenediazonium tetrafluoroborate

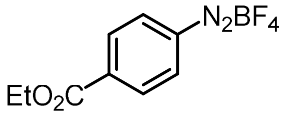

The title product was obtained by using ethyl 4-aminobenzoate (3.30 g, 20.0 mmol, 1.0 equiv.) according to GP 3 as a white solid (4.91 g, 18.6 mmol, 93%).

^{1}H NMR (300 MHz, DMSO-d$_6$) δ/ppm = 8.80 (d, *J* = 9.0 Hz, 2H), 8.44 (d, *J* = 9.0 Hz, 2H), 4.41 (q, *J* = 7.1 Hz, 2H), 1.36 (t, *J* = 7.1 Hz, 3H). **^{13}C NMR** (75 MHz, DMSO-d$_6$) δ/ppm = 163.3, 139.4, 133.2, 131.2, 120.2, 62.4, 14.0. **^{19}F NMR** (376 MHz, DMSO-d$_6$) δ/ppm = -148.23 and -148.28 (s, BF$_4$). These data are in agreement with those reported previously in the literature.[22]

4-Acetylbenzenediazonium tetrafluoroborate

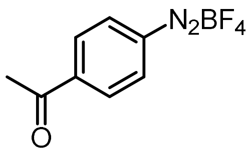

The title product was obtained by using 4-acetylaniline (865.1 mg, 6.46 mmol, 1.0 equiv.) according to GP 3 as a white solid (1.01 g, 4.33 mmol, 67%).

^{1}H NMR (400 MHz, DMSO-d$_6$) δ/ppm = 8.80 (d, *J* = 8.6 Hz, 2H), 8.41 (d, *J* = 8.6 Hz, 2H), 2.71 (s, 3H). **^{13}C NMR** (101 MHz, DMSO-d$_6$) δ/ppm = 196.7, 144.6, 133.2, 130.1, 119.5, 27.4. **^{19}F NMR** (376 MHz, DMSO-d$_6$) δ/ppm = -148.20 and -148.26 (s, BF$_4$). These data are in agreement with those reported previously in the literature.[25]

4-(Methylsulfonyl)benzenediazonium tetrafluoroborate

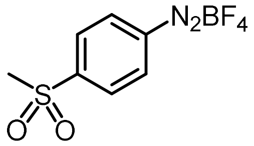

The title product was obtained by using 4-(methylsulfonyl)aniline (702.0 mg, 4.10 mmol, 1.0 equiv.) according to GP 3 as a white solid (1.07 g, 3.97 mmol, 97%).

^{1}H NMR (300 MHz, DMSO-d$_6$) δ/ppm = 8.92 (d, *J* = 9.1 Hz, 2H), 8.50 (d, *J* = 9.1 Hz, 2H), 3.46 (s, 3H). **^{13}C NMR** (101 MHz, DMSO-d$_6$) δ/ppm = 149.9, 133.8, 129.4, 121.5, 42.5. **^{19}F NMR** (376 MHz, DMSO-d$_6$) δ/ppm = -148.21 and -148.27 (s, BF_4).

4-(Phenyl)benzenediazonium tetrafluoroborate

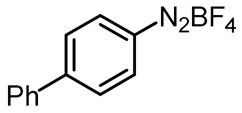

The title product was obtained by using 4-aminobiphenyl (507.7 mg, 3.00 mmol, 1.0 equiv.) according to GP 3 as a white solid (645.9 mg, 2.41 mmol, 80%).

^{1}H NMR (400 MHz, DMSO-d$_6$) δ/ppm = 8.73 (d, *J* = 8.8 Hz, 2H), 8.32 (d, *J* = 8.8 Hz, 2H), 7.96-7.87 (m, 2H), 7.65-7.56 (m, 3H). **^{13}C NMR** (101 MHz, DMSO-d$_6$) δ/ppm = 151.5, 136.4, 133.5, 130.8, 129.6, 129.0, 128.0, 113.4. **^{19}F NMR** (376 MHz, DMSO-d$_6$) δ/ppm = -148.21 and -148.26 (s, BF_4). These data are in agreement with those reported previously in the literature.[22]

4-(*tert*-Butyl)benzenediazonium tetrafluoroborate

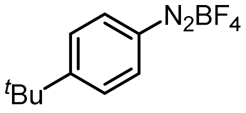

The title product was obtained by using 4-(*tert*-butyl)aniline (597.0 mg, 4.00 mmol, 1.0 equiv.) according to GP 3 as a white solid (578.4 mg, 2.33 mmol, 58%).

^{1}H NMR (600 MHz, DMSO-d$_6$) δ/ppm = 8.59 (d, *J* = 9.0 Hz, 2H), 8.03 (d, *J* = 9.0 Hz, 2H), 1.35 (s, 9H). **^{13}C NMR** (151 MHz, DMSO-d$_6$) δ/ppm = 165.5, 132.8, 128.5, 112.2, 36.5, 30.2. **^{19}F NMR** (565 MHz, DMSO-d$_6$) δ/ppm = -148.20 and -148.26 (s, BF_4). These data are in agreement with those reported previously in the literature.[22]

4-Methylbenzenediazonium tetrafluoroborate

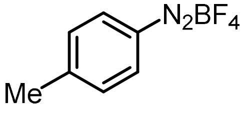

The title product was obtained by using 4-methylaniline (535.8 mg, 5.00 mmol, 1.0 equiv.) according to GP 3 as a white solid (844.9 mg, 4.10 mmol, 82%).

^{1}H NMR (400 MHz, DMSO-d$_6$) δ/ppm = 8.55 (d, *J* = 8.6 Hz, 2H), 7.79 (d, *J* = 8.6 Hz, 2H), 2.57 (s, 3H). **^{13}C NMR** (101 MHz, DMSO-d$_6$) δ/ppm = 153.9, 132.7, 131.8, 112.0, 22.4. **^{19}F NMR** (376 MHz, DMSO-d$_6$) δ/ppm = -148.23 and -148.28 (s, BF_4). These data are in agreement with those reported previously in the literature.[22]

2-Methoxybenzenediazonium tetrafluoroborate

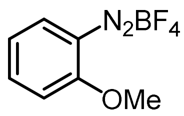

The title product was obtained by using 2-methoxyaniline (615.8 mg, 5.00 mmol, 1.0 equiv.) according to GP 3 as a light brown solid (995.8 mg, 4.49 mmol, 90%).

^{1}H NMR (600 MHz, DMSO-d$_6$) δ/ppm = 8.52 (dd, *J* = 8.4, 1.4 Hz, 1H), 8.26-8.21 (m, 1H), 7.69 (d, *J* = 8.8 Hz, 1H), 7.47-7.42 (m, 1H), 4.20 (s, 3H). **^{13}C NMR** (151 MHz, DMSO-d$_6$) δ/ppm = 162.0, 143.7, 132.4, 122.9, 114.9, 102.3, 58.7. **^{19}F NMR** (565 MHz, DMSO-d$_6$) δ/ppm = -148.25 and -148.30 (s, BF_4). These data are in agreement with those reported previously in the literature.[25]

2-Methylbenzenediazonium tetrafluoroborate

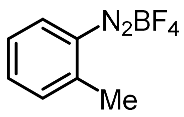

The title product was obtained by using 2-metylaniline (643.0 mg, 6.00 mmol, 1.0 equiv.) according to GP 3 as a white solid (1.10 g, 5.48 mmol, 91%).

^{1}H NMR (400 MHz, DMSO-d$_6$) δ/ppm = 8.66-8.61 (m, 1H), 8.18-8.12 (m, 1H), 7.87-7.76 (m, 2H), 2.74 (s, 3H). **^{13}C NMR** (101 MHz, DMSO-d$_6$) δ/ppm = 143.8, 140.7, 132.6, 132.5, 128.9, 116.0, 18.2. **^{19}F NMR** (376 MHz, DMSO-d$_6$) δ/ppm = -148.21 and -148.27 (s, BF_4). These data are in agreement with those reported previously in the literature.[25]

2-Nitrobenzenediazonium tetrafluoroborate

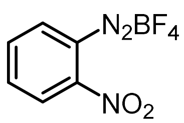

The title product was obtained by using 2-nitroaniline (1.38 g, 10.00 mmol, 1.0 equiv.) according to GP 3 after purification by recrystallization from acetone/Et_2O as a yellow solid (1.37 g, 5.78 mmol, 58%).

^{1}H NMR (600 MHz, DMSO-d$_6$) δ/ppm = 9.14-9.08 (m, 1H), 8.82-8.76 (m, 1H), 8.57-8.50 (d, 1H), 8.44-8.39 (m, 1H). **^{13}C NMR** (151 MHz, DMSO-d$_6$) δ/ppm = 144.5, 142.2, 136.52, 136.49, 128.0, 111.1. **^{19}F NMR** (565 MHz, DMSO-d$_6$) δ/ppm = -148.26 and -148.32 (s, BF_4). These data are in agreement with those reported previously in the literature.[26]

2-Cyanobenzenediazonium tetrafluoroborate

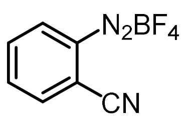

The title product was obtained by using 2-aminobenzonitrile (1.18 g, 10.00 mmol, 1.0 equiv.) according to GP 3 after purification by recrystallization from acetone/Et_2O as a pale orange solid (1.65 g, 7.61 mmol, 76%).

^{1}**H NMR** (600 MHz, DMSO-d_6) δ/ppm = 9.08-8.93 (m, 1H), 8.68-8.53 (m, 1H), 8.51-8.41 (m, 1H), 8.37-8.22 (m, 1H). ^{13}**C NMR** (151 MHz, DMSO-d_6) δ/ppm = 141.0, 136.4, 135.6, 134.6, 118.7, 113.7, 112.4. ^{19}**F NMR** (565 MHz, DMSO-d_6) δ/ppm = -148.22 and -148.28 (s, BF_4). These data are in agreement with those reported previously in the literature.[26]

3-Chlorobenzenediazonium tetrafluoroborate

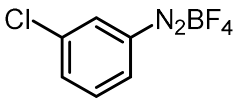

The title product was obtained by using 3-chloroaniline (1.02 g, 8.00 mmol, 1.0 equiv.) according to GP 3 after purification by recrystallization from acetone/Et_2O as a white solid (1.09 g, 4.80 mmol, 60%).

^{1}**H NMR** (600 MHz, DMSO-d_6) δ/ppm = 8.89-8.83 (m, 1H), 8.70-8.63 (m, 1H), 8.40-8.33 (m, 1H), 8.05-7.96 (m, 1H). ^{13}**C NMR** (151 MHz, DMSO-d_6) δ/ppm = 141.0, 134.5, 132.8, 131.6, 131.5, 117.8. ^{19}**F NMR** (565 MHz, DMSO-d_6) δ/ppm = -148.21 and -148.26 (s, BF_4). These data are in agreement with those reported previously in the literature.[26]

3-Fluorobenzenediazonium tetrafluoroborate

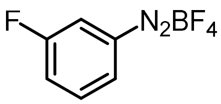

The title product was obtained by using 3-fluoroaniline (777.8 mg, 7.00 mmol, 1.0 equiv.) according to GP 3 after purification by recrystallization from acetone/Et_2O as a white solid (822.8 mg, 3.92 mmol, 56%).

^{1}**H NMR** (600 MHz, DMSO-d_6) δ/ppm = 8.72-8.65 (m, 1H), 8.62-8.55 (m, 1H), 8.27-8.18 (m, 1H), 8.11-7.99 (m, 1H). ^{13}**C NMR** (151 MHz, DMSO-d_6) δ/ppm = 160.5 (d, *J* = 252.4 Hz), 133.5 (d, *J* = 8.7 Hz), 129.8 (d, *J* = 3.6 Hz), 129.1 (d, *J* = 21.2 Hz), 119.4 (d, *J* = 30.4 Hz), 117.3 (d, *J* = 12.2 Hz). ^{19}**F NMR** (565 MHz, DMSO-d_6) δ/ppm = -105.64 – -105.77 (m, 1F), -148.20 and -148.25 (s, BF_4). These data are in agreement with those reported previously in the literature.[25]

4-Iodobenzenediazonium tetrafluoroborate

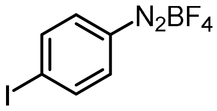

The title product was obtained by using 4-iodoaniline (657.1 mg, 3.00 mmol, 1.0 equiv.) according to GP 3 as a white solid (872.0 mg, 2.74 mmol, 91%).

^{1}**H NMR** (300 MHz, DMSO-d_6) δ/ppm = 8.43 (d, *J* = 9.1 Hz, 2H), 8.35 (d, *J* = 9.1 Hz, 2H). ^{13}**C NMR** (75 MHz, DMSO-d_6) δ/ppm = 140.2, 132.9, 115.2, 113.7. ^{19}**F NMR** (282 MHz,

DMSO-d_6) δ/ppm = -148.20 and -148.25 (s, BF_4). These data are in agreement with those reported previously in the literature.[25]

2.4 Synthesis of Aryl Triethylgermanes

General Procedure 4 (GP 4)

The corresponding aryl iodide (1.0 equiv.) and triethylgermane chloride (1.1 equiv.) were dissolved in degassed and anhydrous THF (0.2 M) under an argon atmosphere. Then a solution of *i*PrMgCl·LiCl (1.3 M in THF, 1.2 equiv.) was added dropwise and the reaction mixture and stirred for 3 h at room temperature. The reaction was quenched with water, the organic phase was separated, and the aqueous layer was extracted with DCM (2x). The organic phases were combined and dried with $MgSO_4$. After filtration and evaporation of solvents *in vacuo* the crude mixture was purified by silica gel chromatography column.

General Procedure 5 (GP 5)

The corresponding aryl bromide (1.0 equiv.) was dissolved in degassed and anhydrous THF (0.5 M) under an argon atmosphere. Solution of *i*PrMgCl·LiCl (1.3 M in THF, 1.1 equiv.) was added slowly at -78 °C, and the reaction mixture was stirred for 1 h at the same temperature. Then triethylgermane chloride (1.1 equiv.) was added dropwise, and the reaction solution was allowed to warm up to room temperature and was stirred overnight. The reaction was quenched with water, the organic phase was separated, and the aqueous layer was extracted with DCM (2x). The organic phases were combined, dried with $MgSO_4$, and filtered. After evaporation of solvents *in vacuo* the crude mixture was purified by silica gel chromatography column.

General Procedure 6 (GP 6)

The corresponding aryl bromide (1.0 equiv.) was dissolved in degassed and anhydrous THF (0.5 M) under an argon atmosphere. Solution of *n*BuLi (2.5 M in hexane, 1.2 equiv.) was added slowly at -78 °C, and the reaction mixture was stirred for 1 h at the same temperature. Then triethylgermane chloride (1.1 equiv.) was added dropwise, and the reaction solution was stirred overnight at room temperature. The reaction was quenched by addition of water, the organic phase was separated, and the aqueous layer was extracted with DCM (2x). The organic phases were combined, dried with $MgSO_4$, and filtered. After evaporation of solvents *in vacuo* a crude mixture was purified by silica gel chromatography column.

(4-Bromo-3-methylphenyl)triethylgermane

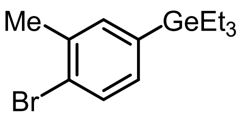

The title product was obtained by using 1-bromo-4-iodomethylbenzene (445.4 mg, 1.50 mmol, 1.0 equiv.) according to GP 4 after purification by column chromatography (*n*-hexane) as a colorless oil (456.8 mg, 1.38 mmol, 92%).

R$_f$ = 0.76 (*n*-hexane). **^{1}H NMR** (400 MHz, $CDCl_3$) δ/ppm = 7.50 (d, *J* = 7.8 Hz, 1H), 7.27 (s, 1H), 7.10 (d, *J* = 7.8 Hz, 1H), 2.41 (s, 3H), 1.10-1.02 (m, 9H), 1.02-0.93 (m, 6H). **^{13}C NMR** (101 MHz, $CDCl_3$) δ/ppm = 139.1, 137.3, 136.4, 133.0, 131.9, 125.5, 23.1, 9.0, 4.3. **IR** (neat): ν/cm^{-1} = 2948, 2873, 2330, 2080, 1898, 1559, 1462, 1365, 1230, 1017, 967, 853, 809, 694. **HRMS** (EI) calculated for $C_{13}H_{21}{}^{79}Br^{74}Ge$: 330.0033 [M]$^+$, found: 330.0000.

Triethyl(2,4,6-trifluorophenyl)germane

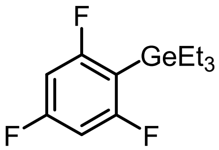

The title product was obtained by using 1,3,5-trifluoro-2-iodobenzene (309.6 mg, 1.20 mmol, 1.0 equiv.) according to GP 4 after purification by column chromatography (*n*-hexane) as a colorless oil (301.5 mg, 1.04 mmol, 86%).

R$_f$ = 0.74 (*n*-hexane). **^{1}H NMR** (600 MHz, $CDCl_3$) δ/ppm = 6.60-6.55 (m, 2H), 1.13-1.02 (m, 15H). **^{13}C NMR** (151 MHz, $CDCl_3$) δ/ppm = 167.1 (ddd, *J* = 241.9, 20.8, 14.8 Hz), 164.2 (dt, *J* = 248.4, 15.7 Hz), 108.3 (td, *J* = 40.6, 4.0 Hz), 99.8 (ddd, *J* = 32.6, 24.5, 4.2 Hz), 8.9, 5.8 (t, *J* = 2.6 Hz). **^{19}F NMR** (564 MHz, $CDCl_3$) δ/ppm = -94.59 (t, *J* = 7.9 Hz, 2F), -108.61 (quint, *J* = 8.5 Hz, 1F). **IR** (neat): ν/cm^{-1} = 2955, 2911, 2876, 2738, 2326, 1625, 1594, 1460, 1412, 1325, 1284, 1230, 1156, 1112, 1066, 1004, 838, 708. **HRMS** (EI) calculated for $C_{10}H_{12}F_3{}^{70}Ge$: 259.0134 [M-Et]$^+$, found 259.0127.

Triethyl(2-isopropylphenyl)germane

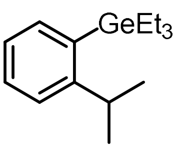

The title product was obtained by using 1-iodo-2-isopropylbenzene (443.0 mg, 1.80 mmol, 1.0 equiv.) according to GP 4 after purification by column chromatography (*n*-hexane) as a colorless oil (402.5 mg, 1.44 mmol, 80%).

R$_f$ = 0.91 (*n*-hexane). **^{1}H NMR** (600 MHz, $CDCl_3$) δ/ppm = 7.38-7.29 (m, 3H), 7.18-7.14 (m, 1H), 2.96 (sept, *J* = 6.9 Hz, 1H), 1.26 (d, *J* = 6.9 Hz, 6H), 1.11-1.01 (m, 15H). **^{13}C NMR** (151 MHz, $CDCl_3$) δ/ppm = 154.9, 137.3, 134.7, 128.9, 125.3, 125.2, 34.6, 25.0, 9.2, 5.7. **IR** (neat): ν/cm^{-1} = 3055, 2952, 2873, 2329, 2118, 2000, 1918, 1585, 1459, 1376, 1220, 1159, 1108, 1013, 966, 757, 700. **HRMS** (EI) calculated for $C_{15}H_{26}{}^{74}Ge$: 280.1246 [M]$^+$, found: 280.1252.

(4-Bromo-2-(trifluoromethoxy)phenyl)triethylgermane

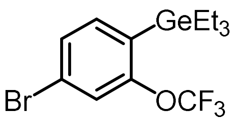

The title product was obtained by using 4-bromo-1-iodo-2-(trifluoromethoxy)benzene (477.0 mg, 1.30 mmol, 1.0 equiv.) according to GP 4 after purification by column chromatography (*n*-hexane) as a colorless oil (350.0 mg, 0.880 mmol, 67%).

R_f = 0.78 (*n*-hexane). **^{1}H NMR** (600 MHz, $CDCl_3$) δ/ppm = 7.40-7.35 (m, 2H), 7.27 (d, *J* = 7.8 Hz, 1H), 1.07-0.99 (m, 15H). **^{13}C NMR** (151 MHz, $CDCl_3$) δ/ppm = 154.4, 137.1, 130.3, 129.1, 123.2, 121.3-121.1 (m), 120.6 (q, *J* = 258.6 Hz), 8.9, 4.7. **^{19}F NMR** (564 MHz, $CDCl_3$) δ/ppm = -56.25 (d, *J* = 1.5 Hz, 3F). **IR** (neat): ν/cm^{-1} = 2952, 2876, 2332, 2095, 1900, 1572, 1464, 1377, 1239, 1168, 1014, 938, 818, 700. **HRMS** (EI) calculated for $C_{13}H_{18}{}^{79}BrF_3{}^{74}GeO$: 399.9699 [M]$^+$, found: 399.9721.

Triethyl(2-methoxyphenyl)germane

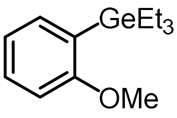

The title product was obtained by using 2-iodoanisole (561.7 mg, 2.40 mmol, 1.0 equiv.) according to GP 4 after purification by column chromatography (*n*-pentane) as a colorless oil (528.1 mg, 1.98 mmol, 82%).

R_f = 0.68 (*n*-pentane). **^{1}H NMR** (600 MHz, $CDCl_3$) δ/ppm = 7.34-7.28 (m, 2H), 6.97-6.93 (m, 1H), 6.85-6.82 (m, 1H), 3.79 (s, 3H), 1.08-0.98 (m, 15H). **^{13}C NMR** (151 MHz, $CDCl_3$) δ/ppm = 163.7, 135.3, 129.9, 127.7, 120.6, 109.5, 55.1, 9.2, 4.8. **IR** (neat): ν/cm^{-1} = 2945, 2871, 2834, 2331, 2159, 1739, 1578, 1458, 1427, 1235, 1169, 1014, 966, 840, 769, 707. **HRMS** (EI) calculated for $C_{13}H_{22}{}^{74}GeO$: 268.0877 [M]$^+$, found: 268.0873.

(3-Bromo-5-chlorophenyl)triethylgermane

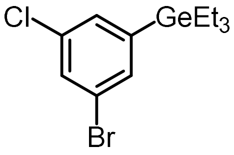

The title product was obtained by using 1-bromo-3-chloro-5-iodobenzene (634.7 mg, 2.00 mmol, 1.0 equiv.) according to GP 4 after purification by column chromatography (*n*-hexane) as a colorless oil (493.8 mg, 1.41 mmol, 70%).

R_f = 0.80 (*n*-hexane). **^{1}H NMR** (300 MHz, $CDCl_3$) δ/ppm = 7.48-7.44 (m, 1H), 7.39 (dd, *J* = 1.9, 0.8 Hz, 1H), 7.29 (dd, *J* = 1.9, 0.8 Hz, 1H), 1.10-0.94 (m, 15H). **^{13}C NMR** (75 MHz, $CDCl_3$) δ/ppm = 145.2, 135.0, 134.6, 132.3, 131.0, 123.1, 8.9, 4.4. **IR** (neat): ν/cm^{-1} = 3854, 2950, 2873, 2659, 2330, 2109, 1906, 1546, 1458, 1422, 1378, 1228, 1114, 1015, 968, 852, 772, 738, 690. **HRMS** (EI) calculated for $C_{12}H_{18}{}^{79}Br^{35}Cl^{74}Ge$: 349.9487 [M]$^+$, found: 349.9501.

Trimethyl(4-(triethylgermyl)phenyl)silane

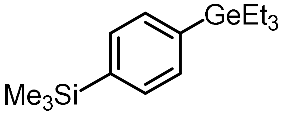

The title product was obtained by using (4-bromophenyl)trimethylsilane (687.6 mg, 3.00 mmol, 1.0 equiv.) according to GP 6 after purification by column chromatography (*n*-hexane) as a colorless oil (862.1 mg, 2.79 mmol, 93%).

R$_f$ = 0.70 (*n*-hexane). **^{1}H NMR** (300 MHz, $CDCl_3$) δ/ppm = 7.51 (d, *J* = 8.0 Hz, 2H), 7.44 (d, *J* = 8.0 Hz, 2H), 1.14-0.93 (m, 15H), 0.28 (s, 9H). **^{13}C NMR** (75 MHz, $CDCl_3$) δ/ppm = 140.8, 140.1, 133.5, 132.8, 9.1, 4.3, -1.0. **HRMS** (EI) calculated for $C_{15}H_{28}{}^{74}GeSi$: 310.1172 $[M]^+$, found: 310.1180. These data are in agreement with those reported previously in the literature.[27]

Benzo[*d*][1,3]dioxol-5-yltriethylgermane

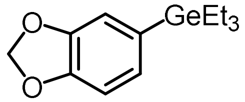

The title product was obtained by using 5-iodo-1,3-benzodioxole (402.0 mg, 2.00 mmol, 1.0 equiv.) according to GP 4 after purification by column chromatography (*n*-hexane) as a colorless oil (475.3 mg, 1.69 mmol, 85%).

R$_f$ = 0.30 (*n*-hexane). **^{1}H NMR** (400 MHz, $CDCl_3$) δ/ppm = 6.93-6.82 (m, 3H), 5.93 (s, 2H), 1.11-1.01 (m, 9H), 1.00-0.91 (m, 6H). **^{13}C NMR** (101 MHz, $CDCl_3$) δ/ppm = 147.8, 147.5, 132.5, 127.4, 113.5, 108.8, 100.5, 9.0, 4.5. **IR** (neat): ν/cm^{-1} = 2946, 2318, 2098, 1856, 1600, 1477, 1413, 1320, 1227, 1108, 1038, 937, 876, 803, 697. **HRMS** (EI) calculated for $C_{13}H_{20}{}^{74}GeO_2$: 282.0670 $[M]^+$, found 282.0680.

Triethyl(thiophen-2-yl)germane

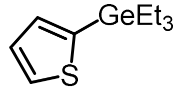

The title product was obtained by using 2-iodothiophene (630.1 mg, 3.00 mmol, 1.0 equiv.) according to GP 4 after purification by column chromatography (5:1 *n*-hexane/EtOAc) as a colorless oil (715.3 mg, 2.94 mmol, 98%).

R$_f$ = 0.91 (5:1 *n*-hexane/EtOAc). **^{1}H NMR** (400 MHz, $CDCl_3$) δ/ppm = 7.59 (dd, *J* = 4.6, 0.9 Hz, 1H), 7.22 (dd, *J* = 4.6, 3.3 Hz, 1H), 7.18 (dd, *J* = 3.3, 0.9 Hz, 1H), 1.14-0.99 (m, 15H). **^{13}C NMR** (101 MHz, $CDCl_3$) δ/ppm = 137.6, 133.4, 129.7, 127.9, 9.0, 5.7. **HRMS** (EI) calculated for $C_{10}H_{18}{}^{74}GeS$: 244.0336 $[M]^+$, found: 244.0342. These data are in agreement with those reported previously in the literature.[27]

Triethyl(thiophen-3-yl)germane

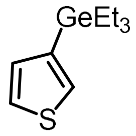

The title product was obtained by using 3-iodothiophene (363.0 mg, 1.72 mmol, 1.0 equiv.) according to GP 4 after purification by column chromatography (*n*-hexane) as a colorless oil (378.0 mg, 1.56 mmol, 90%).

R$_f$ = 0.70 (*n*-hexane). ^{1}**H NMR** (600 MHz, $CDCl_3$) δ/ppm = 7.42 (dd, *J* = 4.7, 2.5 Hz, 1H), 7.34 (dd, *J* = 2.5, 1.1 Hz, 1H), 7.14 (dd, *J* = 4.7, 1.1 Hz, 1H), 1.10-1.05 (m, 9H), 1.02-0.95 (m, 6H). ^{13}**C NMR** (151 MHz, $CDCl_3$) δ/ppm = 138.4, 131.7, 129.9, 125.2, 9.1, 5.0. **HRMS** (EI) calculated for $C_8H_{13}{}^{74}GeS$: 214.9944 [M-Et]$^+$, found: 214.9950. These data are in agreement with those reported previously in the literature.[27]

2-Methoxy-5-(triethylgermyl)benzonitrile

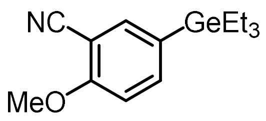

The title product was obtained by using 5-iodo-2-methoxybenzonitrile (362.7 mg, 1.40 mmol, 1.0 equiv.) according to GP 5 after purification by column chromatography (10:1 *n*-pentane/EtOAc) as a colorless oil (320.7 mg, 1.10 mmol, 78%).

R$_f$ = 0.50 (8:1 *n*-pentane/EtOAc). ^{1}**H NMR** (600 MHz, $CDCl_3$) δ/ppm = 7.58 (d, *J* = 1.5 Hz, 1H), 7.56 (dd, *J* = 8.3, 1.5 Hz, 1H), 6.96 (d, *J* = 8.3 Hz, 1H), 3.92 (s, 3H), 1.07-1.01 (m, 9H), 1.00-0.94 (m, 6H). ^{13}**C NMR** (151 MHz, $CDCl_3$) δ/ppm = 161.4, 139.9, 139.2, 131.7, 117.2, 111.0, 101.9, 56.0, 8.9, 4.3. **IR** (neat): ν/cm^{-1} = 2948, 2226, 2035, 1911, 1584, 1493, 1458, 1375, 1266, 1186, 1139, 1091, 1017, 968, 899, 817, 698. **HRMS** (ESI) calculated for $C_{14}H_{21}{}^{74}GeNNaO$: 316.0727 [M+Na]$^+$, found: 316.0720.

4-(Triethylgermyl)benzonitrile

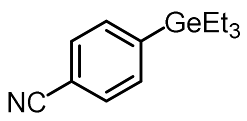

The title product was obtained by using 4-iodobenzonitrile (251.9 mg, 1.10 mmol, 1.0 equiv.) according to GP 5 after purification by column chromatography (50:1 *n*-pentane/Et_2O) as a yellow oil (199.3 mg, 0.76 mmol, 69%).

R$_f$ = 0.30 (50:1 *n*-pentane/Et_2O). ^{1}**H NMR** (400 MHz, $CDCl_3$) δ/ppm = 7.59 (d, *J* = 8.3 Hz, 2H), 7.53 (d, *J* = 8.3 Hz, 2H), 1.09-0.96 (m, 15H). ^{13}**C NMR** (101 MHz, $CDCl_3$) δ/ppm = 147.7, 134.6, 131.1, 119.3, 112.0, 8.9, 4.2. **IR** (neat): ν/cm^{-1} = 2949, 2228, 2161, 1922, 1591, 1460, 1383, 1310, 1228, 1081, 1015, 967, 819, 697. **HRMS** (ESI) calculated for $C_{13}H_{19}{}^{74}GeNNa$: 286.0621 [M+Na]$^+$, found: 286.0613.

(4-Chloro-3-fluorophenyl)triethylgermane

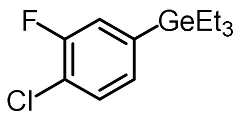

The title product was obtained by using 1-chloro-2-fluoro-4-iodobenzene (615.5 mg, 2.40 mmol, 1.0 equiv.) according to GP 4 after purification by column chromatography (*n*-pentane) as a colorless oil (590.7 mg, 2.04 mmol, 85%).

R$_f$ = 0.80 (*n*-pentane). **^{1}H NMR** (600 MHz, $CDCl_3$) δ/ppm = 7.38-7.33 (m, 1H), 7.18 (dd, *J* = 8.9, 1.3 Hz, 1H), 7.12 (dd, *J* = 7.7, 1.3 Hz, 1H), 1.08-1.02 (m, 9H), 1.01-0.95 (m, 6H). **^{13}C NMR** (151 MHz, $CDCl_3$) δ/ppm = 158.1 (d, *J* = 251.9 Hz), 141.5 (d, *J* = 3.1 Hz), 130.4 (d, *J* = 4.0 Hz), 130.3, 121.6 (d, *J* = 17.8 Hz), 121.0 (d, *J* = 17.6 Hz), 8.9, 4.4. **^{19}F NMR** (565 MHz, $CDCl_3$) δ/ppm = -117.07 – -117.14 (m, 1F). **IR** (neat): ν/cm^{-1} = 2952, 2873, 2332, 2113, 1562, 1479, 1427, 1380, 1274, 1223, 1148, 1049, 1015, 967, 876, 811, 696. **HRMS** (GC-APCI) calculated for $C_{10}H_{13}{}^{35}ClF^{74}Ge$: 260.9896 [M-Et]$^+$, found: 260.9916.

3,5-Dimethyl-4-(triethylgermyl)isoxazole

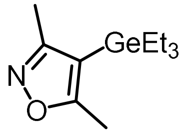

The title product was obtained by using 4-iodo-3,5-dimethylisoxazole (446.0 mg, 2.00 mmol, 1.0 equiv.) according to GP 4 after purification by column chromatography (13:1 *n*-pentane/EtOAc) as a colorless oil (440.0 mg, 1.72 mmol, 86%).

R$_f$ = 0.51 (13:1 *n*-pentane/EtOAc). **^{1}H NMR** (600 MHz, $CDCl_3$) δ/ppm = 2.36 (s, 3H), 2.23 (s, 3H), 1.06-0.95 (m, 15H). **^{13}C NMR** (151 MHz, $CDCl_3$) δ/ppm = 173.0, 163.8, 105.2, 13.2, 12.8, 8.9, 4.7. **IR** (neat): ν/cm^{-1} = 3467, 2953, 2873, 2735, 2184, 1578, 1457, 1397, 1351, 1239, 1093, 1014, 971, 901, 803, 754, 705, 579. **HRMS** (EI) calculated for $C_{11}H_{21}{}^{74}GeNONa$: 280.0727 [M+Na]$^+$, found: 280.0725.

(3-Bromo-5-(triethylgermyl)phenyl)trimethylsilane

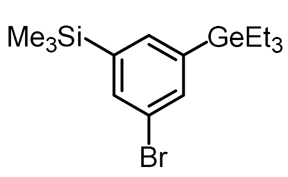

The title product was obtained by using (3,5-dibromophenyl)trimethylsilane (616.2 mg, 2.00 mmol, 1.0 equiv.) according to GP 6 (using 1.0 equiv. of *n*BuLi) after purification by column chromatography (*n*-pentane) as a colorless oil (634.2 mg, 1.63 mmol, 82%).

R$_f$ = 0.65 (*n*-pentane). **^{1}H NMR** (400 MHz, $CDCl_3$) δ/ppm = 7.57-7.53 (m, 1H), 7.51-7.49 (m, 1H), 7.45-7.42 (m, 1H), 1.11-0.94 (m, 15H), 0.27 (s, 9H). **^{13}C NMR** (101 MHz, $CDCl_3$) δ/ppm = 143.1, 142.6, 137.1, 136.8, 135.8, 123.6, 9.0, 4.4, -1.1. **IR** (neat): ν/cm^{-1} = 3019, 2952, 2906, 2872, 2734, 2327, 2110, 1989, 1902, 1535, 1458, 1423, 1372, 1248, 1132, 1104, 1015, 968,

838, 754, 692. **HRMS** (GC-APCI) calculated for $C_{13}H_{22}{}^{81}Br^{74}GeSi$: 375.0016 $[M-Me]^+$, found: 375.0024.

Triethyl(*o*-tolyl)germane

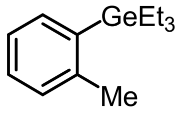

The title product was obtained by using 1-iodo-2-methylbenzene (654.1 mg, 3.00 mmol, 1.0 equiv.) according to GP 4 after purification by column chromatography (*n*-pentane) as a colorless oil (730.0 mg, 2.91 mmol, 97%).

R_f = 0.88 (*n*-pentane). **^{1}H NMR** (600 MHz, $CDCl_3$) δ/ppm = 7.37 (dd, *J* = 7.3, 1.5 Hz, 1H), 7.24 (m, 1H), 7.19-7.13 (m, 2H), 2.41 (s, 3H), 1.05 (s, 15H). **^{13}C NMR** (151 MHz, $CDCl_3$) δ/ppm = 143.5, 138.3, 134.7, 129.7, 128.5, 125.0, 23.2, 9.2, 5.1. **IR** (neat): ν/cm^{-1} = 3415, 3057, 2944, 2653, 2329, 2100, 2000, 1906, 1588, 1453, 1375, 1272, 1220, 1122, 1013, 967, 798, 739, 701. **HRMS** (EI) calculated for $C_{13}H_{22}{}^{74}Ge$: 252.0933 $[M]^+$, found: 252.0927.

(2-(Allyloxy)phenyl)triethylgermane

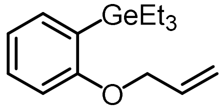

The title product was obtained by using 1-(allyloxy)-2-iodobenzene (780.2 mg, 3.00 mmol, 1.0 equiv.) according to GP 4 after purification by column chromatography (*n*-hexane) as a brown oil (751.3 mg, 2.56 mmol, 85%).

R_f = 0.53 (*n*-hexane). **^{1}H NMR** (600 MHz, $CDCl_3$) δ/ppm = 7.35-7.27 (m, 2H), 6.99-6.91 (m, 1H), 6.83-6.78 (m, 1H), 6.12-6.01 (m, 1H), 5.39 (dd, *J* = 17.3, 1.6 Hz, 1H), 5.27 (dd, *J* = 10.5, 1.6 Hz, 1H), 4.53 (d, *J* = 5.4 Hz, 2H), 1.11-0.98 (m, 15H). **^{13}C NMR** (151 MHz, $CDCl_3$) δ/ppm = 162.6, 135.5, 133.8, 129.9, 127.8, 120.7, 117.3, 110.5, 68.8, 9.2, 4.8. **IR** (neat): ν/cm^{-1} = 3370, 3068, 2944, 2326, 2096, 1896, 1577, 1435, 1375, 1223, 1122, 1013, 922, 828, 751, 702. **MS** (EI) *m/z (%)*: 267 (22), 265 (100) $[M-Et(^{74}Ge)]^+$, 264 (32), 263 (76), 261 (58), 237 (25), 235 (20), 233 (15), 224 (10), 209 (23), 207 (25), 205 (19), 195 (10), 179 (19), 177 (14), 169 (17), 167 (72), 166 (28), 165 (56), 164 (10), 163 (41), 151 (10), 133 (39), 131 (46), 129 (21), 105 (34), 103 (24), 101 (16), 91 (19), 77 (35).

Triethyl(4-(trifluoromethyl)phenyl)germane

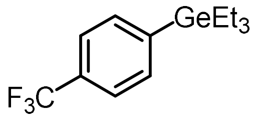

The title product was obtained by using 1-iodo-4-(trifluoromethyl)benzene (1088.5 mg, 3.00 mmol, 1.0 equiv.) according to GP 4 after purification by column chromatography (*n*-pentane) as a colorless oil (815.0 mg, 2.67 mmol, 89%).

R_f = 0.93 (*n*-pentane). **^{1}H NMR** (600 MHz, $CDCl_3$) δ/ppm = 7.57 (d, *J* = 8.1 Hz, 2H), 7.55 (d, *J* = 8.1 Hz, 2H), 1.08-0.99 (m, 15H). **^{13}C NMR** (151 MHz, $CDCl_3$) δ/ppm = 145.3, 134.4, 130.4 (q, *J* = 32.0 Hz), 124.5 (q, *J* = 272.0 Hz), 124.4 (q, *J* = 3.7 Hz), 9.0, 4.3. **^{19}F NMR** (376 MHz, $CDCl_3$) δ/ppm = -62.83 (s, 3F). **HRMS** (EI) calculated for $C_{13}H_{19}F_3{}^{74}Ge$: 306.0651 $[M]^+$, found: 306.0645. These data are in agreement with those reported previously in the literature.[28]

(4-Chlorophenyl)triethylgermane

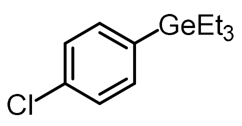

The title product was obtained by using 1-chloro-4-iodobenzene (814.1 mg, 3.00 mmol, 1.0 equiv.) according to GP 4 after purification by column chromatography (*n*-pentane) as a colorless oil (797.0 mg, 2.94 mmol, 98%).

R_f = 0.87 (*n*-pentane). **^{1}H NMR** (600 MHz, $CDCl_3$) δ/ppm = 7.36 (d, *J* = 8.2 Hz, 2H), 7.31 (d, *J* = 8.2 Hz, 2H), 1.08-1.02 (m, 9H), 1.01-0.94 (m, 6H). **^{13}C NMR** (151 MHz, $CDCl_3$) δ/ppm = 138.2, 135.4, 134.5, 128.2, 9.0, 4.3. **HRMS** (EI) calculated for $C_{12}H_{19}{}^{35}Cl^{74}Ge$: 272.0387 $[M]^+$, found: 272.0385. These data are in agreement with those reported previously in the literature.[28]

(4-Bromophenyl)triethylgermane

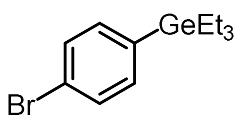

The title product was obtained by using 1-bromo-4-iodobenzene (848.7 mg, 3.00 mmol, 1.0 equiv.) according to GP 4 after purification by column chromatography (*n*-pentane) as a colorless oil (820.0 mg, 2.60 mmol, 87%).

R_f = 0.90 (*n*-pentane). **^{1}H NMR** (600 MHz, $CDCl_3$) δ/ppm = 7.47 (d, *J* = 8.1 Hz, 2H), 7.29 (d, *J* = 8.1 Hz, 2H), 1.09-1.01 (m, 9H), 1.01-0.94 (m, 6H). **^{13}C NMR** (151 MHz, $CDCl_3$) δ/ppm = 138.8, 135.7, 131.1, 123.0, 9.0, 4.3. **HRMS** (EI) calculated for $C_{12}H_{19}{}^{79}Br^{74}Ge$: 315.9882 $[M]^+$, found: 315.9867. These data are in agreement with those reported previously in the literature.[28]

Triethyl(4-iodophenyl)germane

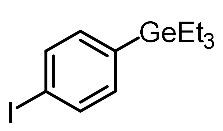

The title product was obtained by using 1,4-diiodobenzene (993.0 mg, 3.01 mmol, 1.0 equiv.) according to GP 4 using *i*PrMgCl (2.0 M in THF, 1.8 ml, 3.61 mmol, 1.2 equiv.) after purification by column chromatography (*n*-pentane) as a colorless oil (963.0 mg, 2.65 mmol, 88%).

R$_f$ = 0.90 (*n*-pentane). **^{1}H NMR** (600 MHz, $CDCl_3$) δ/ppm = 7.67 (d, *J* = 8.0 Hz, 2H), 7.16 (d, *J* = 8.0 Hz, 2H), 1.09-1.01 (m, 9H), 1.01-0.93 (m, 6H). **^{13}C NMR** (151 MHz, $CDCl_3$) δ/ppm = 139.4, 137.0, 135.9, 95.0, 9.0, 4.2. **HRMS** (EI) calculated for $C_{12}H_{19}{}^{74}GeI$: 363.9738 [M]$^+$, found: 363.9744. **IR** (neat): ν/cm^{-1} = 3060, 2948, 2872, 2734, 2329, 2097, 1900, 1741, 1631, 1561, 1463, 1426, 1372, 1228, 1051, 1014, 966, 797, 696. These data are in agreement with those reported previously in the literature.[28]

Triethyl(4-fluorophenyl)germane

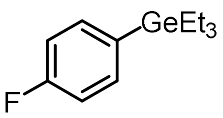

4-Fluorophenyl magnesium chloride (1.0 M in THF, 3 ml, 3.00 mmol, 1.0 equiv.) was added slowly to a solution of triethylgermanium chloride (644.4 mg, 3.30 mmol, 1.1 equiv.) in anhydrous and degassed THF (15 ml), and reaction mixture was stirred for 3 h at room temperature. The reaction was quenched by addition of aqueous solution of NH_4Cl (sat.). Organic phase was separated, and aqueous layer was extracted with DCM (3x 20ml). Organic phases were combined and dried with $MgSO_4$. After evaporation of solvents under reduced pressure, residue was purified by column chromatography (*n*-pentane) providing the final product as a colorless oil (732.0 mg, 2.87 mmol, 96%).

R$_f$ = 0.85 (*n*-pentane). **^{1}H NMR** (600 MHz, $CDCl_3$) δ/ppm = 7.39 (dd, *J* = 7.9, 1.6 Hz, 2H), 7.09-7.01 (m, 2H), 1.05 (t, *J* = 7.5 Hz, 9H), 1.01-0.94 (m, 6H). **^{13}C NMR** (151 MHz, $CDCl_3$) δ/ppm = 163.4 (d, *J* = 246.4 Hz), 135.7 (d, *J* = 6.9 Hz), 135.0 (d, *J* = 3.7 Hz), 115.1 (d, *J* = 19.1 Hz), 9.0, 4.4. **^{19}F NMR** (564 MHz, $CDCl_3$) δ/ppm = -113.75 – -113.84 (m, 1F). **HRMS** (EI) calculated for $C_{12}H_{19}F^{74}Ge$: 256.0683 [M]$^+$, found: 256.0673. These data are in agreement with those reported previously in the literature.[28]

Triethyl(4-(triethylgermyl)phenyl)silane

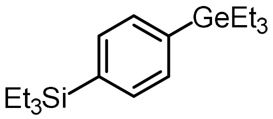

(4-Bromophenyl)triethylgermane (315.8 mg, 1.00 mmol, 1.0 equiv.) was placed into round bottom flask and dissolved in anhydrous and degassed THF (5 ml) under argon. Solution of *n*BuLi (2.5 M in hexane, 480 µl, 3.60 mmol, 1.2 equiv.) was added dropwise at -78 °C, and the obtained solution was stirred at the same temperature for 1 h. After triethylsilylchloride (186.3 µl, 1.10 mmol, 1.1 equiv.) was added dropwise. The reaction mixture was allowed to warm up to room temperature and was stirred overnight. The reaction was quenched by addition of aqueous solution of NH_4Cl (sat.), the organic phase was separated, and the aqueous layer was extracted with DCM (3x 20 ml). The organic phases were combined, dried with $MgSO_4$, and evaporated

in vacuo. The residue was purified by column chromatography (*n*-hexane) to afford the title product as a colorless oil (285.0 mg, 0.810 mmol, 80%).

R$_f$ = 0.68 (*n*-hexane). **^{1}H NMR** (600 MHz, $CDCl_3$) δ/ppm = 7.45 (d, *J* = 7.8 Hz, 2H), 7.41 (d, *J* = 7.8 Hz, 2H), 1.10-1.04 (m, 9H), 1.02-0.94 (m, 15H), 0.79 (q, *J* = 7.8 Hz, 6H). **^{13}C NMR** (151 MHz, $CDCl_3$) δ/ppm = 140.6, 137.2, 133.7, 133.4, 9.1, 7.6, 4.3, 3.5. **IR** (neat): ν/cm^{-1} = 3047, 2951, 2908, 2874, 2733, 2325, 2089, 1910, 1811, 1745, 1648, 1459, 1421, 1377, 1236, 1123, 1009, 967, 798, 697. **MS** (EI) *m/z (%)*: 325 (25), 324 (22), 323 (100) [M-Et(^{74}Ge)]$^+$, 322 (36), 321 (73), 320 (13), 319 (54), 295 (26), 293 (19), 291 (14), 269 (11), 267 (46), 265 (36), 263 (27), 179 (14), 177 (10), 133 (15), 119 (17), 118 (13), 105 (26), 104 (14), 103 (13), 91 (10).

(3-Iodo-5-(triethylgermyl)phenyl)trimethylsilane

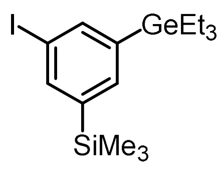

(3-Bromo-5-(triethylgermyl)phenyl)trimethylsilane (232.8 mg, 0.600 mmol, 1.0 equiv.) was placed into a round bottom flask and dissolved in anhydrous and degassed THF (3 ml) under argon. Solution of *n*BuLi (2.5 M in hexane, 0.600 mmol, 1.0 equiv.) was added dropwise at -78 °C and the reaction mixture was stirred for 30 min. Then iodine (167.5 mg, 0.660 mmol, 1.1 equiv.) was added in small portions at the same temperature. After that the reaction mixture was stirred for 4 h at room temperature. The reaction was quenched by addition of water and organic phase was separated. The aqueous phase was extracted with DCM (3x 20 ml). Organic layers were combined, dried with $MgSO_4$ and the solvents were removed under reduced pressure. The title product was obtained after purification by column chromatography (*n*-pentane) as a colorless oil (244.9 mg, 0.563 mmol, 94%).

R$_f$ = 0.72 (*n*-pentane). **^{1}H NMR** (600 MHz, $CDCl_3$) δ/ppm = 7.79-7.74 (m, 1H), 7.72-7.68 (m, 1H), 7.50-7.47 (m, 1H), 1.15-1.01 (m, 9H), 1.02-0.93 (m, 6H), 0.26 (s, 9H). **^{13}C NMR** (151 MHz, $CDCl_3$) δ/ppm = 143.5, 143.0, 142.8, 141.7, 137.6, 97.1, 9.0, 4.4, -1.1. **IR** (neat): ν/cm^{-1} = 2951, 2906, 2872, 2733, 2328, 2088, 1998, 1936, 1528, 1457, 1425, 1370, 1248, 1135, 1095, 1014, 968, 838, 754, 694. **MS** (EI) *m/z* (%): 409 (24), 408 (19), 407 (100), 406 (34), 405 (73), 404 (11), 403 (53), 379 (29), 377 (21), 375 (16), 353 (13), 351 (55), 350 (18), 349 (46), 347 (34), 168 (27), 167 (23), 166 (16), 73 (37).

2.5 Synthesis of Bpin-substituted Aryl Germanes

Triethyl(4-(4,4,5,5-tetramethyl-1,3,2-dioxaborolan-2-yl)phenyl)germane

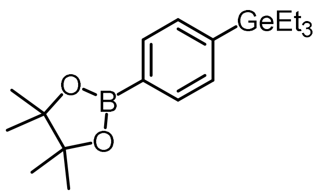

Triethyl(4-iodophenyl)germane (1.09 g, 3.00 mmol, 1.0 equiv.) was added to a round bottom flask and dissolved in anhydrous and degassed THF (20 ml) under argon. Solution of *i*PrMgCl (2.0 M in THF, 1.8 ml, 3.60 mmol, 1.2 equiv.) was added dropwise at 0 °C and the reaction was stirred for 30 min. Tris *iso*-propylborate (1.0 M in THF, 3.3 mL, 3.30 mmol, 1.2 equiv.) was added and the reaction was stirred at room temperature for 12 h. The solvent was removed *in vacuo* and anhydrous toluene (20 ml) and pinacol (1.42 g, 12.0 mmol, 4.0 equiv.) were added. The reaction was stirred for 8 h under reflux. It was quenched by addition of aqueous solution of NH_4Cl (sat.), the organic phase was separated and the aqueous phase was extracted with DCM (3x 20 ml). The combined organic phases were dried with $MgSO_4$ and the solvent was removed under reduced pressure. The title product was obtained after purification by column chromatography (50:1 *n*-hexane/EtOAc) as a white solid (496.0 mg, 1.34 mmol, 45%).

R_f = 0.47 (50:1 *n*-hexane/EtOAc). **^{1}H NMR** (600 MHz, $CDCl_3$) δ/ppm = 7.77 (d, *J* = 7.7 Hz, 2H), 7.45 (d, *J* = 7.7 Hz, 2H), 1.34 (s, 12H), 1.08-1.02 (m, 9H), 1.01-0.95 (m, 6H). **^{13}C NMR** (151 MHz, $CDCl_3$) δ/ppm = 144.1, 136.4, 134.0, 133.5, 83.8, 25.0, 9.1, 4.2. **HRMS** (EI) calculated for $C_{18}H_{31}B^{74}GeO_2$: 364.1623 $[M]^+$, found: 364.1636. These data are in agreement with those reported previously in the literature.[27]

Trimethyl(3-(4,4,5,5-tetramethyl-1,3,2-dioxaborolan-2-yl)-5-(triethylgermyl)phenyl)silane

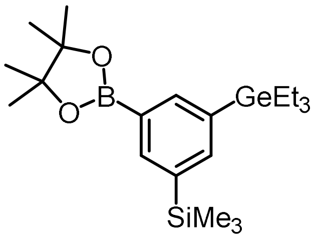

In an argon-filled glovebox, (3-bromo-5-(triethylgermyl)phenyl)trimethylsilane (970.0 mg, 2.50 mmol, 1.0 equiv.), bis(pinacolato)diboron (698.3 mg, 2.75 mmol, 1.1 equiv.), KOAc (736.1 mg, 7.50 mmol, 3.0 equiv.), and $Pd(dppf)Cl_2 \cdot CH_2Cl_2$ (61.2 mg, 0.075 mmol, 3 mol. %) were mixed in a screw top vial and dissolved in anhydrous and degassed DMF (4 ml). The solution was stirred overnight at 100 °C. Then the reaction was quenched by addition of water, and the aqueous phase was extracted with DCM (3x 20 ml). Organic phase was dried with $MgSO_4$ followed by removing solvent *in vacuo*. The title product was obtained after purification of the residue by column chromatography (3:1 *n*-pentane/Et_2O) as a yellow oil (783.1 mg, 1.80 mmol, 72%).

R_f = 0.67 (3:1 *n*-pentane/Et_2O). **^{1}H NMR** (600 MHz, $CDCl_3$) δ/ppm = 7.92 (m, 1H), 7.85 (m, 1H), 7.67 (m, 1H), 1.34 (s, 12H), 1.10-1.04 (m, 9H), 1.04-0.98 (m, 6H), 0.28 (s, 9H). **^{13}C NMR** (151 MHz, $CDCl_3$) δ/ppm = 142.0, 141.0, 139.8, 138.5, 138.0, 83.8, 25.0, 9.1, 4.4, -0.9 (carbon atom attached to boron atom is not observed). **IR** (neat): ν/cm^{-1} = 2952, 2873, 2733, 2328, 2093, 1863, 1808, 1579, 1459, 1397, 1340, 1309, 1247, 1135, 1015, 964, 839, 754, 696. **HRMS** (GC-APCI) calculated for $C_{19}H_{34}B^{74}GeO_2Si$: 407.1627 $[M\text{-}Et]^+$, found: 407.1668.

2.6 Synthesis of *p*-F-PhB(MIDA) and *p*-F-PhSiMe$_3$

(4-Fluorophenyl)boronic acid MIDA ester

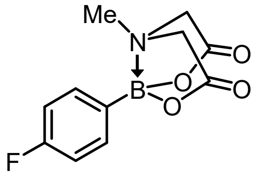

(4-Fluorophenyl)boronic acid (559.7 mg, 4.00 mmol, 1.0 equiv.), methyliminodiacetic acid (MIDA) (588.5 mg, 4.00 mmol, 1.0 equiv.), were mixed in a flask equipped with Dean-Stark trap and dissolved in DMSO/PhMe (9 ml, 98 : 2). The solution was stirred for 4 h under reflux. After evaporation of a significant part of the solvents minimal volumes of acetone and Et_2O were added subsequently to cause precipitation of the product. The obtained solid was filtered, washed with Et_2O (15 ml) and dried in high vacuum to afford the final product as a colorless solid (931.1 mg, 3.71 mmol, 93%).

^{1}H NMR (600 MHz, CD_3CN) δ/ppm = 7.57-7.47 (m, 2H), 7.16-7.09 (m, 2H), 4.06 (d, *J* = 17.1 Hz, 2H), 3.88 (d, *J* = 17.1 Hz, 2H), 2.50 (s, 3H). **^{13}C NMR** (151 MHz, CD_3CN) δ/ppm = 169.4, 164.8 (d, *J* = 245.3 Hz), 135.7 (d, *J* = 7.9 Hz), 115.7 (d, *J* = 20.2 Hz), 62.8, 48.5 (carbon atom attached to boron atom is not observed). **^{19}F NMR** (565 MHz, CD_3CN) δ/ppm = -114.18 – -114.30 (m, 1F). **IR** (neat): ν/cm^{-1} = 3014, 2961, 1752, 1596, 1508, 1458, 1297, 1213, 1154, 1002, 873, 823, 708. **HRMS** (ESI) calculated for $C_{11}H_{11}BFNNaO_4$: 274.0657 $[M+Na]^+$, found: 274.0651.

(4-Fluorophenyl)trimethylsilane

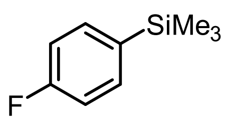

4-Fluorophenyl magnesium bromide (0.8 M in THF, 8.3 ml, 6.60 mmol, 1.1 equiv.) was added dropwise to the solution of trimethylsilyl flouride (762 µl, 6.00 mmol, 1.0 equiv.) in degassed and anhydrous THF (15 ml). The obtained solution was stirred overnight at room temperature. The reaction was quenched by addition of water and organic layer was separated. The aqueous phase was extracted with DCM (2x 20 ml). Organic layers were combined, dried with $MgSO_4$ and the solvents were removed under reduced pressure. The title product was obtained after purification by column chromatography (*n*-hexane) as a colorless oil (519.3 mg, 3.09 mmol, 51%).

R_f = 0.70 (*n*-hexane)**.** **^{1}H NMR** (400 MHz, $CDCl_3$) δ/ppm = 7.53-7.43 (m, 2H), 7.14-6.95 (m, 2H), 0.27 (s, 9H). **^{13}C NMR** (101 MHz, $CDCl_3$) δ/ppm = 163.7 (d, *J* = 247.5 Hz), 136.0 (d, *J* = 3.9 Hz), 135.3 (d, *J* = 7.5 Hz), 115.0 (d, *J* = 19.3 Hz), -0.9. **^{19}F NMR** (376 MHz, $CDCl_3$) δ/ppm = -112.52 – -112.84 (m, 1F). **MS** (EI) *m/z (%)*: 154 (14), 153 (100) $[M-Me]^+$. These data are in agreement with those reported previously in the literature.[29]

3. Derivatization of Biaryal Scaffolds

3.1 Pd-catalyzed Desilylative Acetoxylation of Ethyl 4'-(trimethylsilyl)-[1,1'-biphenyl]-4-carboxylate

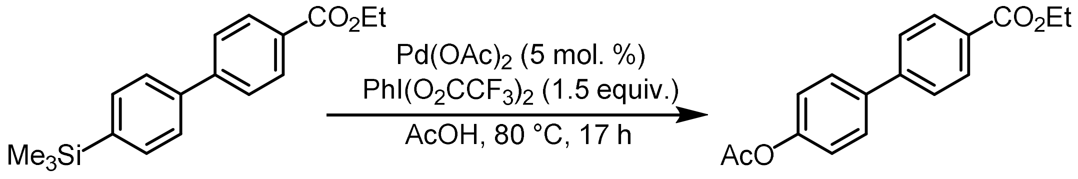

Ethyl 4'-acetoxy-[1,1'-biphenyl]-4-carboxylate

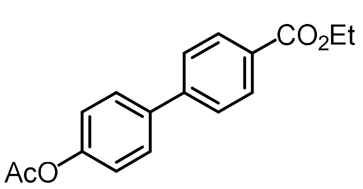

The synthesis was carried out according to the literature.[30] In an argon filled glovebox, 4'-(trimethylsilyl)-[1,1'-biphenyl]-4-carboxylate (59.7 mg, 0.200 mmol, 1.0 equiv.) synthesized according to GP 1, $Pd(OAc)_2$ (2.2 mg, 0.010 mmol, 5 mol. %) and $PhI(O_2CCF_3)_2$ (129.0 mg, 0.300 mmol, 1.5 equiv.) were placed into a screw top vial equipped with magnetic stirring bar and dissolved in AcOH (667 µl). The mixture was stirred at 80 °C for 17 h, after that the reaction mixture was quenched by addition of an aqueous solution of $NaHCO_3$ (sat.). The aqueous phase was extracted with DCM (3x 20 ml) and the organic phases were combined, dried with $MgSO_4$, filtered, before the solvent was removed *in vacuo*. The title product was obtained after purification by column chromatography (15:1 *n*-pentane/EtOAc) as a white solid (49.6 mg, 0.174 mmol, 87%).

R_f = 0.30 (10:1 *n*-pentane/EtOAc). **^{1}H NMR** (600 MHz, $CDCl_3$) δ/ppm = 8.11 (d, *J* = 8.4 Hz, 2H), 7.67-7.59 (m, 4H), 7.19 (d, *J* = 8.4 Hz, 2H), 4.40 (q, *J* = 7.1 Hz, 2H), 2.34 (s, 3H), 1.42 (t, *J* = 7.1 Hz, 3H). **^{13}C NMR** (151 MHz, $CDCl_3$) δ/ppm = 169.6, 166.6, 150.9, 144.8, 138.0, 130.3, 129.5, 128.5, 127.1, 122.2, 61.2, 21.3, 14.5. **IR** (neat): ν/cm^{-1} = 2922, 2858, 2334, 1758, 1697, 1600, 1481, 1373, 1278, 1177, 1115, 1003, 904, 844, 769, 704, 662. **HRMS** (ESI) calculated for $C_{17}H_{16}NaO_4$: 307.0941 $[M+Na]^+$, found: 307.0937.

3.2 Pd-catalyzed Negishi Cross-coupling with (5-Bromo-4'-nitro-[1,1'-biphenyl]-3-yl)trimethylsilane

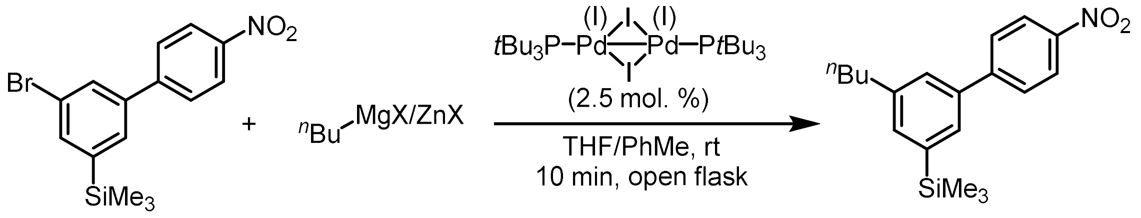

(5-*n*-Butyl-4'-nitro-[1,1'-biphenyl]-3-yl)trimethylsilane

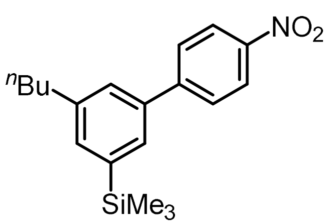

The synthesis was carried out according to the literature.[31] In an oven dried screw top vial equipped with magnetic stirring bar, to a solution of *n*BuMgCl (2.0 M in THF, 200 µl, 0.400 mmol, 2.0 equiv.) were added $ZnCl_2$ (1.0 M in THF, 420 µl, 0.420 mmol, 2.1 equiv.) followed by addition of LiCl (0.5 M in THF, 920 ml, 0.460 mmol, 2.3 equiv.) under argon atmosphere. The resulting solution was stirred for 20 minutes. Thereafter, it was added slowly to a mixture of (5-bromo-4'-nitro-[1,1'-biphenyl]-3-yl)trimethylsilane (70.1 mg, 0.200 mmol, 1.0 equiv.) and Pd$^{(I)}$-I-dimer (4.4 mg, 0.005 mmol, 2.5 mol. %) in anhydrous toluene (750 µl). The reaction mixture was stirred in an open vial for 10 minutes, diluted with 2 ml of pentane, and filtered through a pad of silica. Solvents were removed under reduced pressure, and the residue was purified by silica gel column chromatography (40:1 *n*-pentane/EtOAc) to afford the final product as a yellow oil (49.4 mg, 0.151 mmol, 75%).

R_f = 0.40 (40:1 *n*-pentane/EtOAc). **^{1}H NMR** (600 MHz, $CDCl_3$) δ/ppm = 8.30 (d, *J* = 8.6 Hz, 2H), 7.74 (d, *J* = 8.6 Hz, 2H), 7.56-7.53 (m, 1H), 7.42-7.39 (m, 2H), 2.72-2.68 (m, 2H), 1.70-1.62 (m, 2H), 1.42 (sext, *J* = 7.4 Hz, 2H), 0.96 (t, *J* = 7.4 Hz, 3H), 0.32 (s, 9H). **^{13}C NMR** (151 MHz, $CDCl_3$) δ/ppm = 148.5, 147.1, 143.2, 141.8, 138.4, 134.2, 129.8, 128.2, 128.1, 124.2, 36.0, 34.0, 22.7, 14.1, -0.9. **IR** (neat): ν/cm^{-1} = 2954, 2861, 2106, 1595, 1517, 1456, 1385, 1343, 1248, 1192, 1142, 1107, 1011, 898, 837, 794, 751, 694. **HRMS** (GC-APCI) calculated for $C_{18}H_{22}NO_2Si$: 312.1414 [M-Me]$^+$, found: 312.1442.

3.3 Iodination of (5-*n*-Butyl-4'-nitro-[1,1'-biphenyl]-3-yl)trimethylsilane

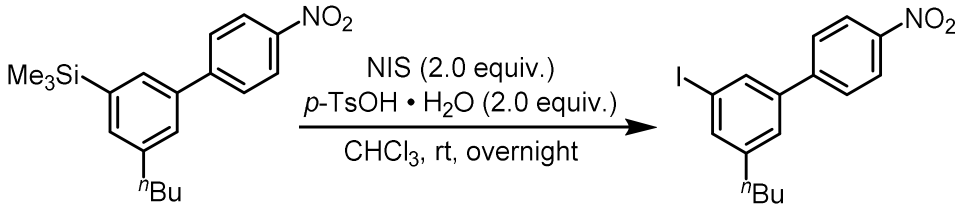

3-*n*-Butyl-5-iodo-4'-nitro-1,1'-biphenyl

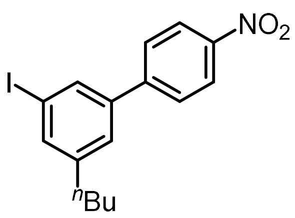

(5-Butyl-4'-nitro-[1,1'-biphenyl]-3-yl)trimethylsilane (49.4 mg, 0.151 mmol, 1.0 equiv.), *N*-iodosuccinimide (NIS) (67.9 mg, 0.302 mmol, 2.0 equiv.), *p*-toluenesulfonic acid monohydrate (*p*-TsOH·H_2O) (57.4 mg, 0.600 mmol, 2.0 equiv.) were placed into a screw top vial equipped with magnetic stirring bar, dissolved in anhydrous $CHCl_3$ (1.5 ml), and stirred overnight at room temperature. The reaction mixture was quenched by addition of DCM, and washed with water (1x 20 ml). The organic phase was separated, dried with $MgSO_4$, and filtered. After that solvents were removed under reduced pressure and the crude mixture was purified by silica gel column chromatography (40:1 *n*-pentane/EtOAc) to afford the title product as a yellow oil (30.1 mg, 0.079 mmol, 52%).

R_f = 0.33 (40:1 *n*-pentane/EtOAc). **^{1}H NMR** (600 MHz, $CDCl_3$) δ/ppm = 8.29 (d, *J* = 8.5 Hz, 2H), 7.79-7.74 (m, 1H), 7.69 (d, *J* = 8.6 Hz, 2H), 7.62-7.60 (m, 1H), 7.40-7.32 (m, 1H), 2.64 (t, *J* = 7.8 Hz, 2H), 1.63 (quint, *J* = 7.8 Hz, 2H), 1.39 (sext, *J* = 7.4 Hz, 2H), 0.95 (t, *J* = 7.4 Hz, 3H). **^{13}C NMR** (151 MHz, $CDCl_3$) δ/ppm = 147.5, 146.4, 146.2, 140.9, 138.0, 133.8, 128.0, 127.1, 124.3, 95.2, 35.5, 33.6, 22.5, 14.0. **IR** (neat): ν/cm^{-1} = 2927, 2858, 2325, 2087, 1992, 1930, 1594, 1559, 1516, 1438, 1389, 1343, 1290, 1182, 1107, 994, 846, 753, 692. **HRMS** (APCI) calculated for $C_{16}H_{17}INO_2$: 382.0298 $[M+H]^+$, found: 382.0295.

3.4 Pd-catalyzed Suzuki Cross-coupling with 2-(4'-Methoxy-[1,1'-biphenyl]-4-yl)-4,4,5,5-tetramethyl-1,3,2-dioxaborolane

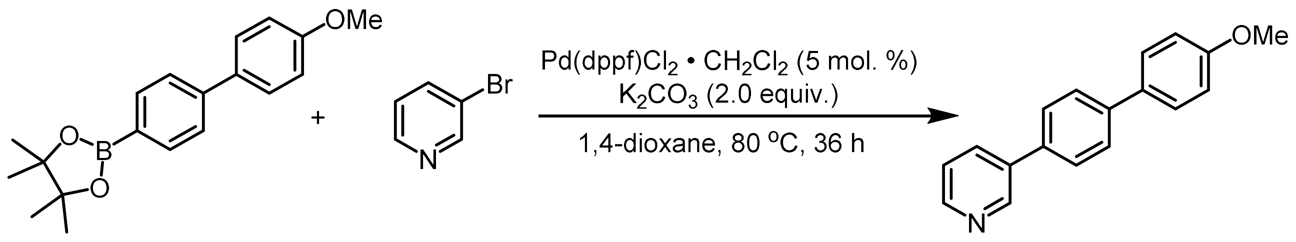

3-(4'-Methoxy-[1,1'-biphenyl]-4-yl)pyridine

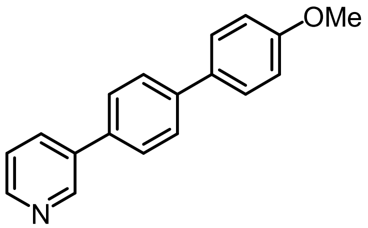

In an argon filled glovebox, 2-(4'-methoxy-[1,1'-biphenyl]-4-yl)-4,4,5,5-tetramethyl-1,3,2-dioxaborolane (62.0 mg, 0.200 mmol, 1.0 equiv.) synthesized according to GP 2, 3-bromopyridine (21.2 µl, 0.220 mmol, 1.2 equiv.), $Pd(dppf)Cl_2 \cdot CH_2Cl_2$ (8.2 mg, 0.010 mmol, 5 mol. %), K_2CO_3 (55.3 mg, 0.400 mmol, 2.0 equiv.) were placed into a screw top vial equipped with magnetic stirring bar, and anhydrous 1,4-dioxane (200 µl) was added. The mixture was stirred at 80 °C for 36 h. Thereafter, the reaction mixture was quenched by the addition of water, and the aqueous phase was extracted with DCM (3x 20 ml). Organic phases were combined, dried with $MgSO_4$, and solvents were removed *in vacuo*. The title product was obtained after purification by column chromatography (20:1 *n*-pentane/EtOAc with 1% Et_3N) as a grey solid (42.3 mg, 0.162 mmol, 81%).

R$_f$ = 0.30 (20:1 *n*-pentane/EtOAc with 1% Et_3N). **^{1}H NMR** (600 MHz, DMSO-d$_6$) δ/ppm = 8.95 (d, *J* = 2.4 Hz, 1H), 8.58 (dd, *J* = 4.7, 1.6 Hz, 1H), 8.14-8.10 (m, 1H), 7.80 (d, *J* = 8.4 Hz, 2H), 7.76 (d, *J* = 8.4 Hz, 2H), 7.69 (d, *J* = 8.7 Hz, 2H), 7.50 (dd, *J* = 8.0, 4.7 Hz, 1H), 7.05 (d, *J* = 8.7 Hz, 2H), 3.81 (s, 3H). **^{13}C NMR** (151 MHz, DMSO-d$_6$) δ/pm = 159.1, 148.5, 147.5, 139.5, 135.3, 135.1, 133.9, 131.7, 127.8, 127.4, 126.8, 123.9, 114.5, 55.2. **IR** (neat): ν/cm^{-1} = 3007, 2960, 2839, 2063, 1907, 1748, 1602, 1535, 1502, 1469, 1438, 1394, 1346, 1322, 1286, 1252, 1218, 1180, 1145, 1117, 1027, 826, 800, 763, 705. **HRMS** (ESI) calculated for $C_{18}H_{16}NO$: 262.1226 [M+H]$^+$, found 262.1225.

3.5 Cu-catalyzed Chan-Lam Coupling with (4'-Bromo-5-(4,4,5,5-tetramethyl-1,3,2-dioxaborolan-2-yl)-[1,1'-biphenyl]-3-yl)trimethylsilane

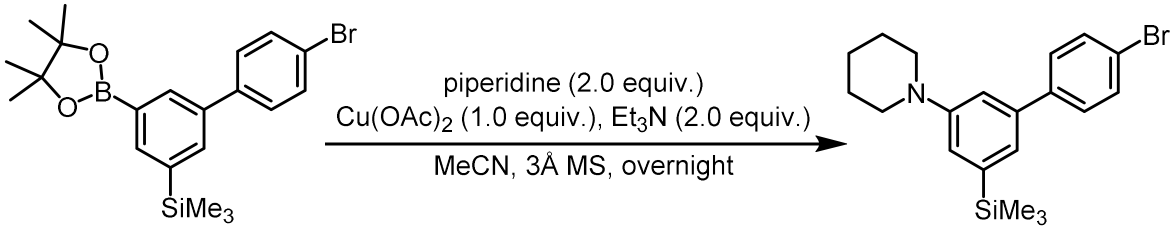

1-(4'-Bromo-5-(trimethylsilyl)-[1,1'-biphenyl]-3-yl)piperidine

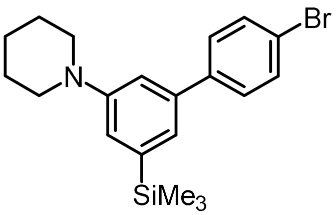

The synthesis was carried out according to the literature.[32] (4'-Bromo-5-(4,4,5,5-tetramethyl-1,3,2-dioxaborolan-2-yl)-[1,1'-biphenyl]-3-yl)trimethylsilane (86.3 mg, 0.200 mmol, 1.0 equiv.) synthesized according to GP 1, $Cu(OAc)_2$ (36.3 mg, 0.200 mmol, 1.0 equiv.), Et_3N (55.8 µl, 0.400 mmol, 2.0 equiv.), piperidine (39.5 µl, 0.400 mmol, 2.0 equiv.) and powdered 3Å molecular sieves (50.0 mg) were placed into a screw top vial equipped with magnetic stirring bar, before MeCN (400 µl) was added. The mixture was stirred overnight at 80 °C. The reaction was quenched by the addition of DCM and filtrated. The resultant filtrate was washed with water (15 ml). The organic phase was dried with $MgSO_4$, and solvents were removed *in vacuo* to afford the final product. The title product was obtained as a colorless oil (60.0 mg, 0.154 mmol, 77%).

^{1}H NMR (600 MHz, $CDCl_3$) δ/ppm = 7.54 (d, *J* = 8.5 Hz, 2H), 7.45 (d, *J* = 8.5 Hz, 2H), 7.14-7.09 (m, 2H), 7.06 (s, 1H), 3.25-3.17 (m, 4H), 1.81-1.70 (m, 4H), 1.64-1.53 (m, 2H), 0.29 (s, 9H). **^{13}C NMR** (151 MHz, $CDCl_3$) δ/ppm = 152.4, 142.0, 141.3, 140.5, 131.8, 129.1, 123.2, 121.2, 121.1, 116.1, 51.1, 26.1, 24.4, -0.9. **IR** (neat): ν/cm^{-1} = 2934, 2854, 2798, 1578, 1488, 1447, 1414, 1386, 1345, 1280, 1245, 1218, 1133, 1073, 1008, 955, 823, 753, 693. **HRMS** (ESI) calculated for $C_{20}H_{27}{}^{79}BrNSi$: 388.1091 $[M+H]^+$, found: 388.1091.

4. Reaction Development

4.1 Catalyst Screening for Electron Poor System

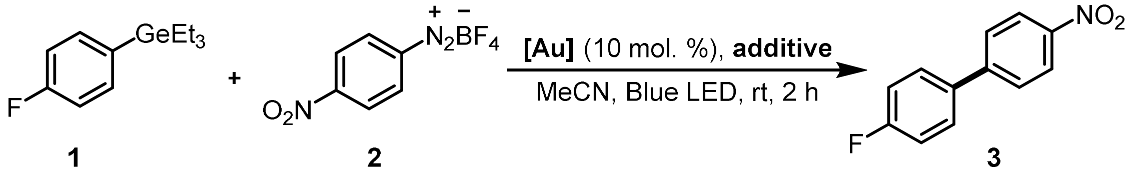

In an argon filled glovebox, (4-fluorophenyl)triethylgermane **1** (12.7 mg, 0.050 mmol, 1.0 equiv.), 4-nitrobenzenediazonium tetrafluoroborate **2** (17.8 mg, 0.075 mmol, 1.5 equiv.), [Au] (0.005 mmol, 10 mol. %) were mixed in a screw top vial equipped with a magnetic stirring bar and dissolved in anhydrous and degassed MeCN (500 µl). The obtained solutions were placed into the blue LED setup and stirred for 2 h. A yield of the product **3** was calculated by ^{19}F NMR using 1,4-difluorobenzene (0.5 M in MeCN, 100 µl, 0.050 mmol, 1.0 equiv.) as internal standard. Results are shown in Table S1.

Table S1. Screening of catalytic systems

Entry	[Au]	Additive	Yield of **3**, %
1	**[(Ph$_3$P)AuCl]**	**–**	**99**
2	[(Ph$_3$P)Au]NTf$_2$	–	93
3	[(Me$_2$S)AuCl]	–	40
4	[(Ph$_3$P)AuCl]	[Ru(bpy)$_3$](PF$_6$)$_2$[a]	67
5[b]	[(Ph$_3$P)AuCl]	–	0
6	–	–	0

[a]1.1 mg (0.00125 mmol, 2.5 mol. %) was used; [b]in the dark

4.2 Ligand Screening for Electron Rich System

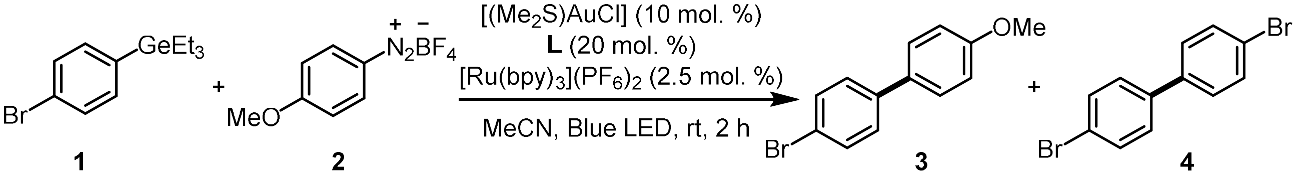

In an argon filled glovebox, (4-bromophenyl)triethylgermane **1** (9.5 mg, 0.030 mmol, 1.0 equiv.), 4-methoxybenzenediazonium tetrafluoroborate **2** (13.3 mg, 0.060 mmol, 2.0 equiv.), [$(Me_2S)AuCl$] (0.9 mg, 0.003 mmol, 10 mol. %), L (0.006 mmol, 20 mol. %), and [$Ru(bpy)_3$]$(PF_6)_2$ (0.6 mg, 0.00075 mmol, 2.5 mol. %) were mixed in a screw top vial equipped with a magnetic stirring bar and dissolved in anhydrous and degassed MeCN (300 µl). The obtained solutions were placed into the blue LED setup and stirred for 2 h. Ratios between heterocoupling **3** and homocoupling **4** products were determined by GC-MS. Results are shown in Table S2.

Table S2. Screening of ligands with ratios of desired product **3** to homocoupling **4**

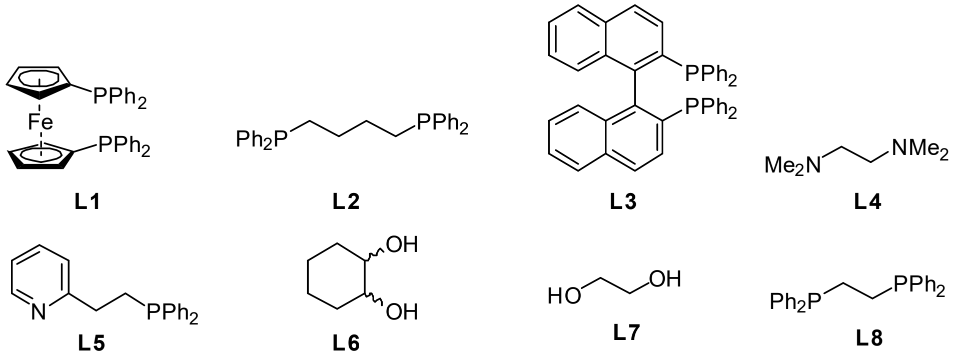

Entry	L	3 : 4
1	Ph_3P	0.8 : 1
2	L1	1.5 : 1
3	L2	2.9 : 1
4	L3	3.6 : 1
5	L4	2.4 : 1
6	L5	2.5 : 1
7	L6	2.0 : 1
8	L7	2.1 : 1
**9**	**L8**	**4.5 : 1**

5. Mechanistic Investigations

5.1 Stoichiometric Experiments

5.1.1 Stoichiometric Experiments with [(Ph3P)AuCl] and Diazonium Salts in the Presence and Absence of [Ru(bpy)3](PF6)2

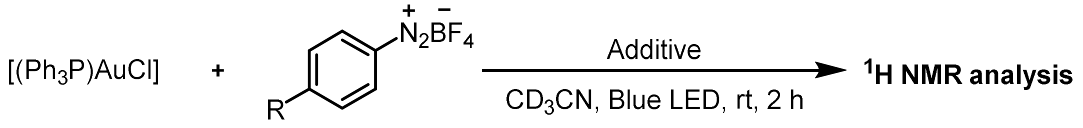

1 **2a - CF3** **2b - OMe**

In an argon filled glovebox **1** (14.8 mg, 0.03 mmol, 1.0 equiv.) and **2a** (7.8 mg, 0.030 mmol, 1.0 equiv) or **2b** (6.7 mg, 0.030 mmol, 1.0 equiv.) we added to a glass vial. To the required experiments, [(Ru(bpy)$_3$](PF$_6$)$_2$ **3** (25.8 mg, 0.03 mmol, 1.0 equiv.) was added. The solids were dissolved in CD_3CN (600 µl) and stirred for 2 h under blue LED. Thereafter mesitylene (**IS$_H$**) (4.2 µl, 0.030 mmol, 1.0 equiv.) or 1,4-difluorobenzene (**IS$_F$**) (3.1 µl, 0.030 mmol, 1.0 equiv.) was added to the solutions and the mixtures were transferred into NMR tubes for analysis.

Table S3. Experimental conditions for assessment of light and photocatalyst with electron poor diazonium salt **2a**. Red = Not used in experiment. Green = Present during experiment.

Experiment	Blue light	Diazonium	3 [Ru(bpy)3]2PF6)
A		4-CF3	
B		4-CF3	
C		4-CF3	
D		4-CF3	

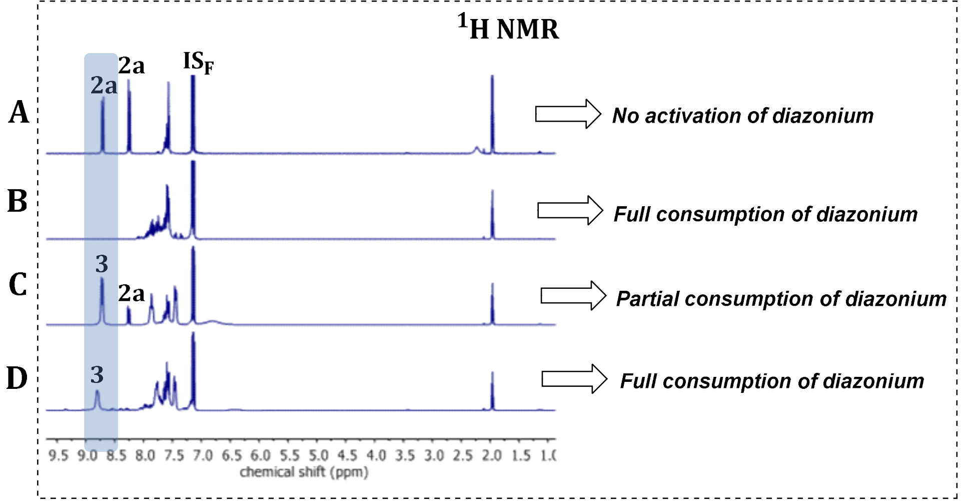

Figure S2. ^{1}H NMR spectra (right) for experiments A-D. Electron rich diazonium salts under light and photocatalyst conditions.

Table S4. Experimental conditions for assessment of light and photocatalyst with electron rich diazonium salt **2b**. Red = Not used in experiment. Green = Present during experiment.

Experiment	Blue light	Diazonium	3<br>[Ru(bpy)$_3$]2PF$_6$)
**E**		4-OMe	
**F**		4-OMe	
**G**		4-OMe	
**H**		4-OMe	

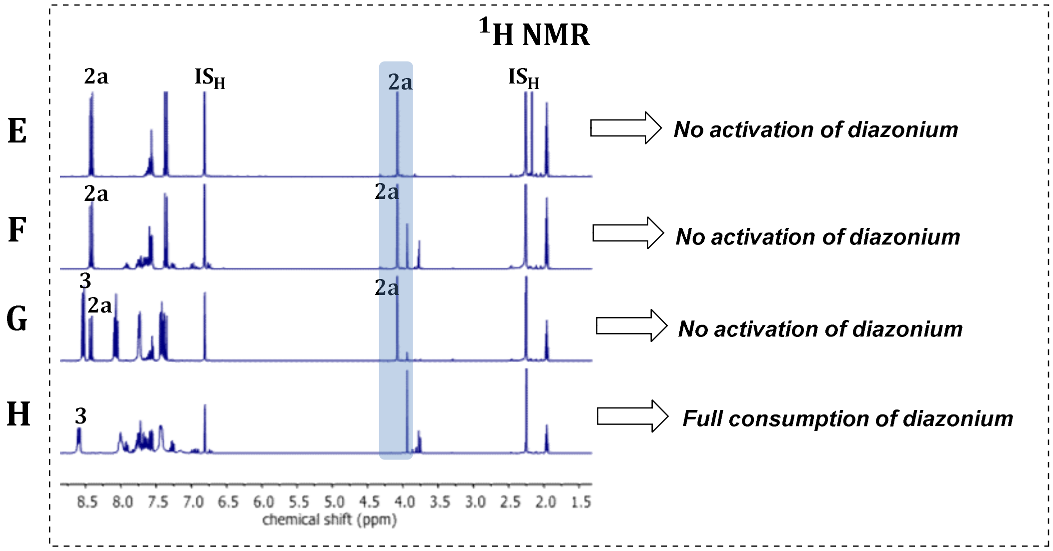

Figure S3. ^{31}P NMR spectra (left) and ^{1}H NMR spectra (right) for experiments E-F. Electron rich diazonium salts under light and photocatalyst conditions.

5.2 UV/Vis Analysis

For the UV/Vis measurements the solutions of 4-methoxybenzenediazonium tetrafluoroborate **1** (blue line) 4-nitrobenzenediazonium tetrafluoroborate **2** (maroon line), triethyl(4-fluorophenyl)germane **3** (black line), and a 1:1:1 combination of **1,** **3** and (PPh3)AuCl (gray line) in anhydrous MeCN (0.5 μμ) were prepared and then measured in 1 ml cuvettes. From the obtained spectra it was found that **2** has absorption in visible area (λ_{max} = 402 nm), whereas **1, 3** and the mixture absorb in UV field (Figure S4).

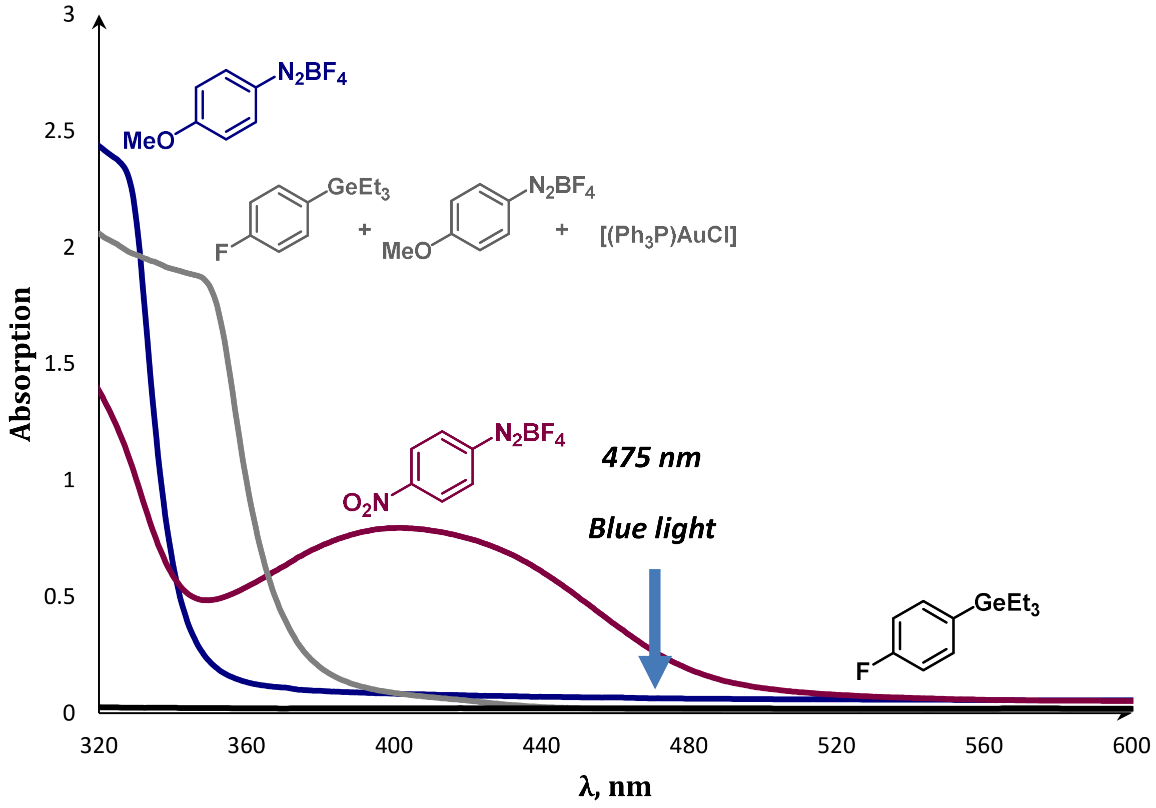

Figure S4. UV-Vis spectrum of relevant reaction components (0.5 μM in MeCN)

5.3 Experiment with UV Light

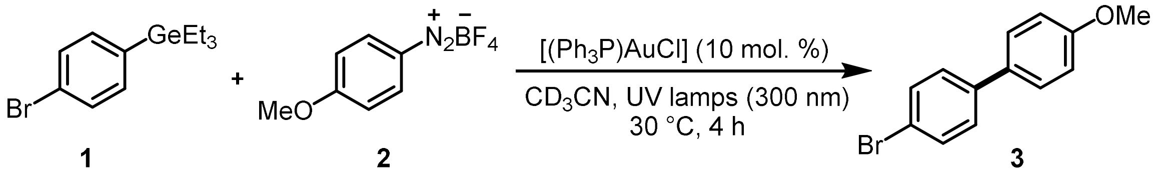

In an argon filled glovebox, (4-bromophenyl)triethylgermane **1** (15.8 mg, 0.050 mmol, 1.0 equiv.), 4-methoxybenzenediazonium salt **2** (22.2 mg, 0.100 mmol, 2.0 equiv.), and [(Ph_3P)AuCl] (2.5 mg, 0.005 mmol, 10 mol. %) were mixed in a screw top quartz vial and dissolved in CD_3CN (500 µl). The obtained solution was stirred being irradiated by using of UV reactor (Rayonet Reactor 200 equipped with 6 lamps and air cooling, λ_{max} = 300 nm) for 4 h. The reaction mixture was analyzed by ^1H NMR using mesitylene (13.9 µl, 0.100 mmol, 2.0 equiv.) as internal standard. Results are shown in the Table S5.

Table S5. The reaction carried out under UV light

**Consumption of 1**, %	**Consumption of 2**, %	**Yield of 3**, %
16	100	traces

6. Computational Details

6.1 General Computational Details

All the DFT calculations were carried out in Gaussian 16 (A.03) program package.[33] Following the methodology of previous work in a related reaction in the group[28], ωB97xD functional was used for optimizations and frequency calculation, combined with 6-31G(d) basis set for all the atoms except gold and ruthenium, where LANL2DZ and the associated pseudopotential was used. Solvation was introduced in optimizations due to the presence of highly charged species through the CPCM implicit solvent model (solvent = acetonitrile). Frequency calculations were analyzed to characterize the nature of the stationary point as minima (no imaginary frequency) or transition state (one imaginary frequency). TD-DFT vertical excitations were also calculated as this level of theory. Additionally, relaxation of transition states towards previous and next intermediates and IRC analysis, when needed, were used to verify the connectivity of the transition states. Finally, potential energies were refined using M06L and 6-311++G(d.p) basis set (LANL2DZ for Au and Ru). 1 M standard state correction was added to the calculations in the solvent phase (1.89 kcal/mol). Reported energies are a sum of potential energies of refined method plus the thermochemistry corrections at 1 M standard state and at standard conditions (298.15 K and 1 atm).

6.2 Competing Pathways in the Activation of *e*-Rich and *e*-Poor ArN_2BF_4

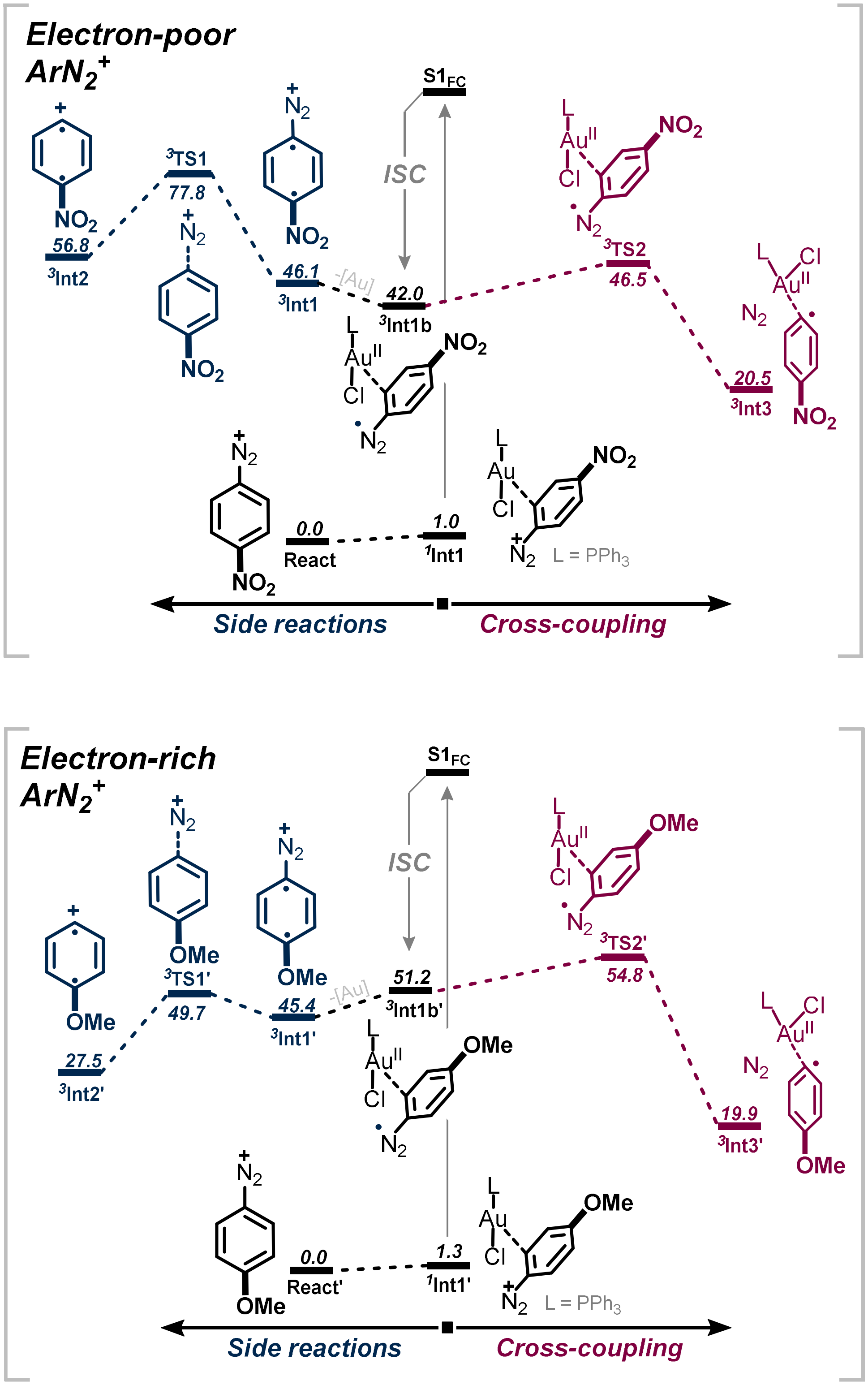

Figure S5. Free energy profile of light mediated diazonium activation. Free energies in kcal/mol.

Table S6. Calculated vertical excitations of diazonium arenes and the corresponding gold complexes with three different methods. S2 is always the bright state (higher oscillator strength).

Intermediate	B3LYPD3		ωB97xD		CAM-B3LYP	
	λ (nm)	f	λ (nm)	f	λ (nm)	f
**React**	329	0.044	292	0.066	294	0.066
**^{1}Int1**	513	0.004	329	0.011	342	0.015
**React'**	303	0.546	283	0.649	285	0.655
**^{1}Int1'**	374	0.005	289	0.460	292	0.440

The vertical excitations show the same trend independently of the method used. As observed experimentally, diazonium salt with electron withdrawing group has the absorption red-shifted (in blue light region) respect to the electron donating group, which need UV light to be reactive.

6.3 Photoredox Reduction of *e*-Rich ArN_2BF_4

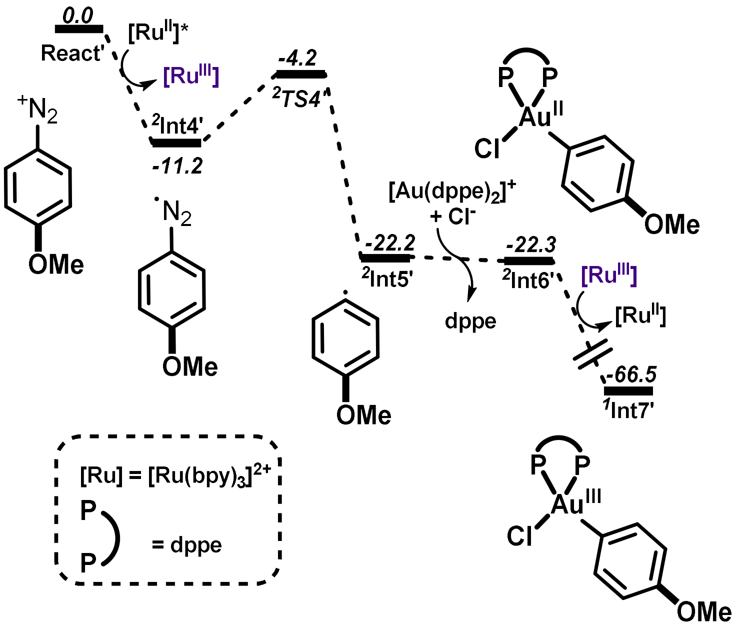

Figure S6. Free energy profile of the photoredox activation of electron donor diazonium arene by [Ru] photocatalyst.

6.4 XYZ Coordinates and Energies for Optimized Structures

[Au]

$Ph_3P—Au—Cl$

Au	-2.04366100	0.33204300	-0.08931900
Cl	0.33170400	0.48150500	-0.13522600
P	-4.33774700	0.19057600	-0.02974300
C	-5.04612400	0.91751600	1.48313300
C	-6.24195800	1.64061500	1.45702500
C	-4.38819400	0.70625000	2.70061200
C	-6.77345500	2.14679400	2.64074100
H	-6.75973600	1.81187300	0.51847000
C	-4.92618700	1.20888600	3.88031700
H	-3.45464700	0.14988400	2.72706200
C	-6.11825800	1.93114100	3.85058600
H	-7.70029700	2.71088900	2.61496700
H	-4.41146900	1.04196200	4.82105300
H	-6.53426700	2.32864200	4.77109000
C	-4.94212800	-1.52720600	-0.08789800
C	-4.31816500	-2.42884000	-0.95824700
C	-6.03240700	-1.94527700	0.68013100
C	-4.78608100	-3.73421900	-1.06216300
H	-3.46669100	-2.11161000	-1.55509300
C	-6.49359500	-3.25546700	0.57611800
H	-6.52282500	-1.25459200	1.35893000
C	-5.87301800	-4.14863200	-0.29354400
H	-4.29871800	-4.42898600	-1.73858400
H	-7.33868400	-3.57619900	1.17684500
H	-6.23397400	-5.16944700	-0.37111700
C	-5.14324800	1.04446200	-1.42225500
C	-4.59893700	2.24818700	-1.88430100
C	-6.31120800	0.54207700	-2.00370200
C	-5.22195100	2.94434700	-2.91484200
H	-3.68756400	2.64074100	-1.44043600
C	-6.92862800	1.24084400	-3.03777000
H	-6.74019000	-0.39183600	-1.65391100
C	-6.38597000	2.44020800	-3.49286200
H	-4.79495100	3.87667000	-3.27016500
H	-7.83359400	0.84538600	-3.48783500
H	-6.86811200	2.98140400	-4.30097300

Zero-point correction = 0.280369 (Hartree/Particle)
Thermal correction to Energy = 0.300130
Thermal correction to Enthalpy = 0.301074
Thermal correction to Gibbs Free Energy = 0.226604
Sum of electronic and zero-point Energies = -1631.505842
Sum of electronic and thermal Energies = -1631.486081
Sum of electronic and thermal Enthalpies = -1631.559608
Sum of electronic and thermal Free Energies = -545.149195
E(M06L) = -1632.179224

React

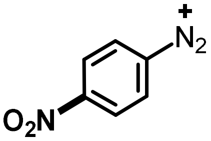

C	-1.07453300	-0.68246800	0.00003200
C	0.31468900	-0.66227900	0.01466500
C	0.96117100	0.56275300	0.01419300
C	0.15408500	1.70336100	-0.00066100
C	-1.24310400	1.69708000	-0.01534000
C	-1.86553300	0.45978300	-0.01504600
H	0.87884300	-1.58524300	0.02597700
H	2.04139700	0.63315100	0.02481900
H	-1.81165500	2.61826900	-0.02648500
H	-2.94454000	0.38280100	-0.02633700
N	0.78872300	2.94013000	-0.00093900
N	1.29177600	3.92166500	-0.00116700
N	-1.75118300	-1.99699900	0.00001600
O	-2.96892000	-1.99871100	-0.01940900
O	-1.04551100	-2.98932300	0.01923500

Zero-point correction = 0.103298 (Hartree/Particle)
Thermal correction to Energy = 0.111897
Thermal correction to Enthalpy = 0.112841
Thermal correction to Gibbs Free Energy = 0.068452
Sum of electronic and zero-point Energies = -545.114350
Sum of electronic and thermal Energies = -545.105751
Sum of electronic and thermal Enthalpies = -545.104806
Sum of electronic and thermal Free Energies = -545.149195
E(M06L) = -545.504526

[1]Int1

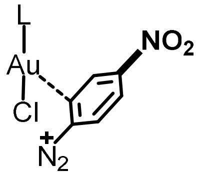

Au	-0.76175800	-0.83376700	2.21565400
P	-2.79688900	-0.63903000	1.16336100
C	-4.12641600	-0.10327000	2.28883800
C	-5.11202200	0.80018300	1.88036700
C	-4.17662400	-0.65270000	3.57519500
C	-6.14006700	1.14781000	2.75323700
H	-5.08016400	1.23657700	0.88679000
C	-5.20747000	-0.30456200	4.44137500
H	-3.41018900	-1.35116500	3.90152800
C	-6.18884700	0.59670900	4.03123500
H	-6.90155000	1.85139500	2.43236400
H	-5.24078600	-0.73228100	5.43821800
H	-6.98990700	0.87190700	4.71013400
C	-3.39554100	-2.17381600	0.39169000
C	-2.49311000	-3.19393600	0.07709200
C	-4.75176600	-2.32707500	0.07899100
C	-2.93871100	-4.35089100	-0.55583100
H	-1.44430400	-3.09196200	0.33714800
C	-5.19284700	-3.48492400	-0.55280100
H	-5.46429600	-1.54639400	0.32849400
C	-4.28713500	-4.49565400	-0.87213500
H	-2.23269900	-5.13963800	-0.79459700
H	-6.24499800	-3.59904000	-0.79276100
H	-4.63507000	-5.39939800	-1.36250300
C	-2.74403600	0.62348500	-0.14956000
C	-2.25109400	1.89130000	0.18319900
C	-3.21617500	0.37918200	-1.44054300
C	-2.26267200	2.91303000	-0.75928000
H	-1.86874400	2.08423600	1.18242500
C	-3.21101200	1.40252200	-2.38682600
H	-3.59103800	-0.60181400	-1.71330300
C	-2.74726000	2.66961600	-2.04490700
H	-1.88907000	3.89627800	-0.49336900
H	-3.57587800	1.20761600	-3.39004500
H	-2.74949600	3.46582500	-2.78237000
C	1.79430200	0.38505200	-0.11924700
C	1.33507700	-0.81744900	-0.66406700

C	0.51136200	-0.92719900	-1.78692700
C	0.16890200	0.25085800	-2.43052200
C	0.64887500	1.45042800	-1.92010200
C	1.42705900	1.54884300	-0.77290600
H	2.40405200	0.39752500	0.77560900
H	0.16893800	-1.88975200	-2.14556400
H	-0.44740000	0.23869700	-3.31933900
H	1.75367600	2.51442900	-0.41058200
N	1.73847000	-1.99076300	-0.03867900
N	2.06577300	-2.91352200	0.46901000
N	0.34454800	2.69065900	-2.65905400
O	-0.13395900	2.57342400	-3.77293100
O	0.59715800	3.74788700	-2.10925700
Cl	1.39759700	-1.02497600	3.21283300

Zero-point correction = 0.385168 (Hartree/Particle)
Thermal correction to Energy = 0.415189
Thermal correction to Enthalpy = 0.416133
Thermal correction to Gibbs Free Energy = 0.318738
Sum of electronic and zero-point Energies = -2176.641576
Sum of electronic and thermal Energies = -2176.611554
Sum of electronic and thermal Enthalpies = -2176.610610
Sum of electronic and thermal Free Energies = -2176.708006
E(M06L) = -2177.702842

³Int1b

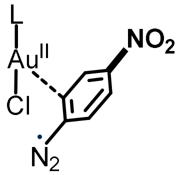

Au	-0.60146800	-0.90458900	-0.12854900
P	-2.94830300	-0.73424900	0.01249600
C	-3.36752200	0.13652000	1.53029100
C	-4.28091200	1.19760000	1.52096100
C	-2.79047300	-0.29574800	2.73334400
C	-4.61163300	1.82377500	2.71809400
H	-4.73594900	1.52981300	0.59407300
C	-3.13230500	0.33552000	3.92192100
H	-2.07709800	-1.11557500	2.73757500
C	-4.03842800	1.39602900	3.91355200
H	-5.31959000	2.64564700	2.71444900
H	-2.68688400	0.00423900	4.85381100
H	-4.29806300	1.89098400	4.84376500
C	-3.72318100	-2.35573100	-0.01844000
C	-3.28842700	-3.30910300	-0.94925800
C	-4.77036100	-2.64729000	0.86223300
C	-3.91426400	-4.54751300	-1.00349700
H	-2.46788300	-3.08714700	-1.62579100
C	-5.38632300	-3.89342100	0.80149500
H	-5.10575600	-1.91174100	1.58599100
C	-4.96039900	-4.83932300	-0.12778000
H	-3.58150700	-5.28704200	-1.72384100
H	-6.19941800	-4.12299100	1.48184800
H	-5.44305900	-5.81041700	-0.16948900
C	-3.43450300	0.23296800	-1.42311000
C	-2.94203400	1.54371200	-1.53800200
C	-4.24158800	-0.31052300	-2.43018700
C	-3.26457700	2.30554900	-2.65157900
H	-2.30614800	1.96345300	-0.76377500
C	-4.55876700	0.46328100	-3.54077800
H	-4.62604100	-1.32129700	-2.34689300
C	-4.07096000	1.76457600	-3.65336500
H	-2.88304000	3.31709300	-2.73856700
H	-5.19018300	0.04871500	-4.31924000
H	-4.31984800	2.35986900	-4.52586000
C	2.70662300	0.45803000	0.94283700
C	2.47791300	-0.35115800	-0.16036000
C	1.59486000	0.03061200	-1.18736400
C	0.91880400	1.25083400	-1.07879400
C	1.14017600	2.03528400	0.05526000
C	2.02161500	1.66566200	1.05884500
H	3.40684100	0.14264300	1.70730200
H	1.51527900	-0.56395900	-2.09303900
H	0.26829400	1.59901200	-1.87275700
H	2.16990700	2.30874300	1.91681200
N	3.22226700	-1.59203300	-0.23842200
N	2.88084900	-2.52315000	-0.87672500
N	0.40875900	3.30099700	0.18313000
O	-0.41478300	3.56815000	-0.68000700
O	0.66207600	4.00889600	1.14166500
Cl	-0.13784600	-2.81006300	1.31219700

Zero-point correction = 0.382901 (Hartree/Particle)
Thermal correction to Energy = 0.413581
Thermal correction to Enthalpy = 0.414525
Thermal correction to Gibbs Free Energy = 0.312942
Sum of electronic and zero-point Energies = -2176.568050
Sum of electronic and thermal Energies = -2176.537371
Sum of electronic and thermal Enthalpies = -2176.536426
Sum of electronic and thermal Free Energies = -2176.638009
E(M06L) = -2177.631701

³Int1

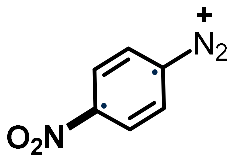

C	-1.07947600	-0.68621600	-0.00730800
C	0.33007900	-0.65608600	0.03995000
C	0.96535200	0.56113100	0.04704200
C	0.16535900	1.71955600	0.00604200
C	-1.24199000	1.70314300	-0.04362000
C	-1.86938200	0.48187600	-0.05075500
H	0.90120600	-1.57575500	0.07059600
H	2.04522800	0.63072800	0.08338000
H	-1.80715000	2.62615600	-0.07493600
H	-2.94977000	0.41708600	-0.08890500
N	0.79302200	2.93550900	0.01663900
N	1.30153500	3.92073100	0.02522100
N	-1.70600200	-1.89854500	-0.01199200
O	-2.96681000	-2.11996500	-0.04666900
O	-1.15549800	-3.05537900	0.02886900

Zero-point correction = 0.099572 (Hartree/Particle)
Thermal correction to Energy = 0.108779
Thermal correction to Enthalpy = 0.109723
Thermal correction to Gibbs Free Energy = 0.063595
Sum of electronic and zero-point Energies = -545.030107
Sum of electronic and thermal Energies = -545.020900
Sum of electronic and thermal Enthalpies = -545.019956
Sum of electronic and thermal Free Energies = -545.066084
E(M06L) = -545.4262706

³TS1

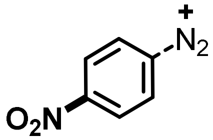

C	-1.08287700	-0.70496100	-0.01388800
C	0.32760800	-0.75435800	0.01870700

C	1.00396100	0.44991200	0.04499400
C	0.23946700	1.60393200	0.03716000
C	-1.14368300	1.68000600	0.00971000
C	-1.83967900	0.48750700	-0.01594200
H	0.85562000	-1.70166500	0.01397300
H	2.08674000	0.48891300	0.06570900
H	-1.66050900	2.63375100	0.00944200
H	-2.92389100	0.46443700	-0.03255800
N	1.22422600	3.17864700	0.05184300
N	0.66599700	4.16050300	0.02511700
N	-1.76407800	-1.89773700	-0.05677100
O	-2.99588800	-2.04659900	-0.15517900
O	-1.26731000	-3.03831700	-0.00876100

Zero-point correction = 0.095513 (Hartree/Particle)
Thermal correction to Energy = 0.105113
Thermal correction to Enthalpy = 0.106057
Thermal correction to Gibbs Free Energy = 0.057982
Sum of electronic and zero-point Energies = -544.976085
Sum of electronic and thermal Energies = -544.966485
Sum of electronic and thermal Enthalpies= -544.965540
Sum of electronic and thermal Enthalpies = -544.965540
Sum of electronic and thermal Free Energies = -545.013615
E(M06L) = -545.3700317

[3]Int2

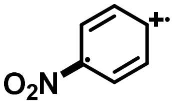

C	-1.08252300	-0.70407900	-0.01579600
C	0.33065900	-0.75350800	0.01686600
C	1.01146700	0.44943200	0.04521100
C	0.23139000	1.59187100	0.03914600
C	-1.14843000	1.68616100	0.01010900
C	-1.84113300	0.48989000	-0.01809600
H	0.85693000	-1.70208600	0.01117000
H	2.09434200	0.48012600	0.06639800
H	-1.66965800	2.63610000	0.01163000
H	-2.92543300	0.46419600	-0.03541700
N	-1.76141400	-1.89311500	-0.05582800
O	-2.99467600	-2.04428200	-0.15457500
O	-1.26603900	-3.03588800	-0.00422100

Zero-point correction = 0.089039 (Hartree/Particle)
Thermal correction to Energy = 0.096070
Thermal correction to Enthalpy = 0.097014
Thermal correction to Gibbs Free Energy = 0.055968
Sum of electronic and zero-point Energies = -435.518611
Sum of electronic and thermal Energies = -435.511581
Sum of electronic and thermal Enthalpies = -435.510636
Sum of electronic and thermal Free Energies = -435.551682
E(M06L) = -435.8440535

N_2

N	1.37986500	1.28183400	0.00000000
N	0.27855900	1.28183400	0.0000000

Zero-point correction = 0.005707 (Hartree/Particle)
Thermal correction to Energy = 0.008067
Thermal correction to Enthalpy = 0.009012
Thermal correction to Gibbs Free Energy = -0.012736
Sum of electronic and zero-point Energies = -109.477078
Sum of electronic and thermal Energies = -109.474718
Sum of electronic and thermal Enthalpies = -109.473774
Sum of electronic and thermal Free Energies = -109.495521
E(M06L) = -109.544745

[3]TS2

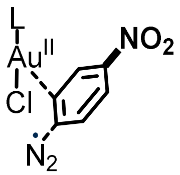

Au	-0.51525700	-1.03576500	0.20848400
P	-2.88134700	-0.76255100	0.16929800
C	-3.43466900	0.10687600	1.64704100
C	-4.45297200	1.06513900	1.55503600
C	-2.88529900	-0.22639000	2.89250500
C	-4.91440100	1.68623400	2.71038700
H	-4.88812400	1.32109900	0.59487700
C	-3.35594300	0.40110400	4.03929200
H	-2.09555600	-0.96833700	2.96420900
C	-4.36629300	1.35736600	3.94838600
H	-5.70358100	2.42720700	2.64084900
H	-2.92932500	0.14625700	5.00351600
H	-4.72747100	1.84843200	4.84623900
C	-3.67185900	-2.37523300	0.05609700
C	-3.20613900	-3.30933100	-0.87993200
C	-4.74748000	-2.68648300	0.89437200
C	-3.83129100	-4.54484400	-0.98443800
H	-2.36118000	-3.07567600	-1.52182400
C	-5.36207700	-3.93007600	0.78430800
H	-5.10575800	-1.96836300	1.62451900
C	-4.90668600	-4.85522200	-0.15165200
H	-3.47462700	-5.26794500	-1.71004200
H	-6.19714800	-4.17409400	1.43226200
H	-5.38825000	-5.82444000	-0.23173600
C	-3.24385400	0.23353500	-1.28424400
C	-2.80283000	1.56682300	-1.29519000
C	-3.89652500	-0.30412900	-2.39993200
C	-3.02326300	2.35635000	-2.41454200
H	-2.28853200	1.98508200	-0.43457000
C	-4.11208100	0.49671100	-3.51610900
H	-4.24405500	-1.33150400	-2.39707100
C	-3.67541500	1.82025600	-3.52582400
H	-2.68111600	3.38550100	-2.42013100
H	-4.62478100	0.08521900	-4.37889100
H	-3.84443700	2.43774100	-4.40198700
C	2.67185400	0.45478400	0.60449000
C	2.18703700	-0.33389700	-0.41729500
C	1.19627900	0.04711700	-1.32007100
C	0.65516600	1.33651200	-1.16901700
C	1.12783400	2.13726400	-0.13431500
C	2.12394200	1.72906500	0.74750500
H	3.44986000	0.09940500	1.27062700
H	0.93930500	-0.56769700	-2.18039100
H	-0.09734400	1.70378700	-1.85600400
H	2.45887400	2.39586000	1.53223600
N	2.99924500	-2.00882400	-0.60197000
N	2.57367900	-2.73424600	-1.35579400
N	0.54564600	3.47379700	0.03358500
O	-0.40052700	3.77479600	-0.67954300
O	1.03669900	4.20581400	0.87528400
Cl	-0.35318300	-2.80594000	1.86558900

Zero-point correction = 0.379731 (Hartree/Particle)
Thermal correction to Energy = 0.410738
Thermal correction to Enthalpy = 0.411683
Thermal correction to Gibbs Free Energy = 0.309003
Sum of electronic and zero-point Energies = -2176.554248
Sum of electronic and thermal Energies = -2176.523241
Sum of electronic and thermal Enthalpies = -2176.522296
Sum of electronic and thermal Free Energies = -2176.624976
E(M06L) = -2177.620548

[3]Int3

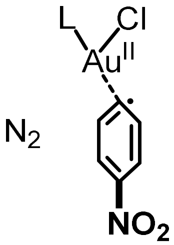

Au	-0.16839400	-1.19922700	1.15455800
P	-2.61473100	-0.73736600	0.70508200
C	-3.48211900	-0.16670600	2.17776600
C	-4.48854700	0.80370300	2.06951900
C	-3.18121700	-0.73767400	3.42150500
C	-5.18724100	1.19543800	3.20568500
H	-4.73172000	1.24528400	1.10906100
C	-3.88780200	-0.33744800	4.54947800
H	-2.40015600	-1.48693900	3.50712100
C	-4.88657300	0.62872800	4.44278200
H	-5.96759800	1.94432400	3.12202000
H	-3.65321100	-0.77732800	5.51293500
H	-5.43233500	0.94190000	5.32701400
C	-3.32714000	-2.27733000	0.10775300
C	-2.68719500	-2.97714600	-0.92572600
C	-4.49066500	-2.78969100	0.69346600
C	-3.22535600	-4.17235800	-1.38324600
H	-1.77322500	-2.59640400	-1.37144100
C	-5.01810400	-3.99059300	0.22954900
H	-4.98419500	-2.25728000	1.49962600
C	-4.38905600	-4.67943900	-0.80493600
H	-2.73143100	-4.71169200	-2.18430300
H	-5.92209600	-4.38717000	0.67937900
H	-4.80358000	-5.61738100	-1.16000800
C	-2.68269400	0.55135600	-0.55095600
C	-2.31581800	1.85298700	-0.17559300
C	-3.05660200	0.27914600	-1.87181100
C	-2.36192900	2.87927100	-1.11149400
H	-2.00349400	2.06529900	0.84300800
C	-3.07781100	1.31044500	-2.80497300
H	-3.34456400	-0.72252900	-2.17134600
C	-2.73827600	2.60786200	-2.42664800
H	-2.08900700	3.88720300	-0.81661600
H	-3.37139100	1.09977500	-3.82767300
H	-2.76239800	3.40892100	-3.15813400
C	1.23754100	1.31488900	0.45542300
C	0.89284000	0.06335600	-0.08117800
C	0.72354600	-0.15645000	-1.45813600
C	0.57256700	0.95579000	-2.27098400
C	0.74388200	2.21541600	-1.70009600
C	1.09424900	2.42193500	-0.36609300
H	1.53136000	1.42854300	1.49267900
H	0.63150700	-1.15540600	-1.86868600
H	0.34066200	0.85591400	-3.32326600
H	1.25341500	3.42423300	0.00995200
N	1.63400000	-4.02419500	-0.52069000
N	0.65879300	-4.27394200	-0.96742100
N	0.56438100	3.39482200	-2.56154100
O	0.32558800	3.20106500	-3.74187600
O	0.64778600	4.49370900	-2.03831700
Cl	-0.41890400	-3.06067200	2.64590400

Zero-point correction = 0.380043 (Hartree/Particle)
Thermal correction to Energy = 0.412196
Thermal correction to Enthalpy = 0.413140
Thermal correction to Gibbs Free Energy = 0.308186
Sum of electronic and zero-point Energies = -2176.592435
Sum of electronic and thermal Energies = -2176.560282
Sum of electronic and thermal Enthalpies = -2176.559338
Sum of electronic and thermal Free Energies = -2176.664292
E(M06L) = -2177.661246

React'

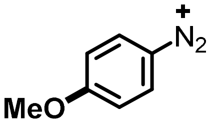

C	-0.98502000	-0.72677500	0.04394000
C	0.42122000	-0.61193100	0.05328300
C	1.00543400	0.63404400	0.02918500
C	0.16819700	1.76223500	-0.00482000
C	-1.23994200	1.67586100	-0.01573500
C	-1.80204600	0.42725000	0.00886800
H	1.05547400	-1.48807000	0.07899600
H	2.08326500	0.74491700	0.03640300
H	-1.84968400	2.57075500	-0.04239900
H	-2.87797700	0.30221500	0.00238700
N	0.74474100	2.99652400	-0.02953200
N	1.21165500	4.00247800	-0.04970000
O	-1.63560100	-1.87749000	0.06685100
C	-0.90523500	-3.10644700	0.10874900
H	-0.29651800	-3.16056100	1.01541500
H	-1.66076800	-3.88920800	0.12354700
H	-0.27897000	-3.21176200	-0.78126000

Zero-point correction = 0.133993 (Hartree/Particle)
Thermal correction to Energy = 0.142589
Thermal correction to Enthalpy = 0.143533
Thermal correction to Gibbs Free Energy = 0.100322
Sum of electronic and zero-point Energies = -455.158319
Sum of electronic and thermal Energies = -455.149723
Sum of electronic and thermal Enthalpies = -455.148779
Sum of electronic and thermal Free Energies = -455.191990
E(M06L) = -455.5195688

[1]Int1'

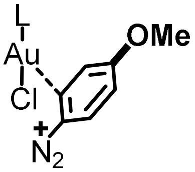

Au	-8.17902900	-2.69084700	-0.13433800
P	-10.16752900	-1.53266900	-0.05883900
C	-10.29978300	-0.42530500	1.38083400
C	-11.01807600	0.77252200	1.32656800
C	-9.69946000	-0.81826300	2.58218500
C	-11.12573100	1.57199100	2.46097400
H	-11.48683900	1.09267500	0.40149200
C	-9.81285300	-0.01820800	3.71426600
H	-9.13593400	-1.74640100	2.63270000
C	-10.52172100	1.17991100	3.65282300
H	-11.67976500	2.50389100	2.40980700
H	-9.34173300	-0.32823800	4.64162900
H	-10.60329300	1.80765000	4.53458900
C	-11.60366300	-2.65266100	0.04015700
C	-11.65105000	-3.74792600	-0.83126000
C	-12.65607200	-2.42832600	0.93072400
C	-12.74524400	-4.60495600	-0.81411600
H	-10.83276200	-3.93128000	-1.52319200
C	-13.74828600	-3.29374900	0.94708600
H	-12.62947500	-1.58409100	1.61230300

C	-13.79434100	-4.37917400	0.07687300
H	-12.77681400	-5.45200500	-1.49185100
H	-14.56234600	-3.11651100	1.64265400
H	-14.64592700	-5.05210100	0.09331200
C	-10.46335900	-0.51131800	-1.53665700
C	-9.37421400	-0.05696800	-2.28709000
C	-11.76183400	-0.14840700	-1.91212600
C	-9.57775600	0.77503600	-3.38432600
H	-8.36640100	-0.35883400	-2.01714200
C	-11.96155300	0.68128800	-3.01130700
H	-12.61730200	-0.51350200	-1.35086400
C	-10.87120800	1.14722300	-3.74392500
H	-8.72676900	1.12399800	-3.96040400
H	-12.97037900	0.96097200	-3.29711300
H	-11.03099100	1.79313000	-4.60147100
C	-5.49278600	-0.67001400	1.65964600
C	-4.96522000	-0.73434600	0.35397100
C	-5.43515200	0.06036800	-0.70282600
C	-6.44953000	0.95530700	-0.44588800
C	-6.98766700	1.04987500	0.85437400
C	-6.50209400	0.22559300	1.89588100
H	-5.10933500	-1.31211400	2.44325600
H	-5.00685500	-0.02894200	-1.69378300
H	-6.81307300	1.58200000	-1.24959400
H	-6.94678900	0.31887500	2.87884800
N	-3.96155800	-1.61805700	0.09835900
N	-3.14119600	-2.33428500	-0.10884100
O	-7.94833300	1.88917900	1.19789000
C	-8.52696700	2.74565500	0.21159900
H	-9.29393100	3.31083600	0.73676000
H	-7.77379700	3.42563600	-0.19622800
H	-8.98611100	2.15612600	-0.58765700
Cl	-6.18896500	-3.99629400	-0.23439600

Zero-point correction = 0.416096 (Hartree/Particle)
Thermal correction to Energy = 0.446047
Thermal correction to Enthalpy = 0.446991
Thermal correction to Gibbs Free Energy = 0.350018
Sum of electronic and zero-point Energies = -2086.686299
Sum of electronic and thermal Energies = -2086.656348
Sum of electronic and thermal Enthalpies = -2086.655404
Sum of electronic and thermal Free Energies = -2086.752377
E(M06L) = -2087.716814

^{3}Int1b′

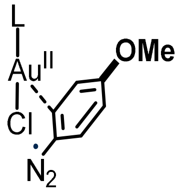

Au	-8.86627100	-1.28464100	-1.01472700
P	-10.66336200	0.15749100	-0.51244600
C	-10.28103000	0.95395000	1.04824000
C	-10.10377400	2.34079200	1.12673700
C	-10.17468200	0.15389100	2.19916500
C	-9.85738300	2.92650400	2.36265000
H	-10.17094700	2.95775700	0.23713300
C	-9.93704400	0.75203900	3.42759800
H	-10.28407300	-0.92478400	2.13073200
C	-9.78329500	2.13735900	3.50923500
H	-9.72591800	4.00080100	2.42925500
H	-9.86535100	0.14011300	4.32004100
H	-9.59583600	2.60208000	4.47175500
C	-12.18000200	-0.79988400	-0.35901000
C	-12.51415600	-1.69810400	-1.38256200
C	-13.04716100	-0.60380300	0.72334900
C	-13.71222400	-2.39639000	-1.31795500
H	-11.83721200	-1.85838100	-2.21649100
C	-14.24318800	-1.31177100	0.77752400
H	-12.80190600	0.10006200	1.51105700
C	-14.57408200	-2.20612800	-0.23769000
H	-13.97037100	-3.09369000	-2.10769300
H	-14.91712100	-1.16009600	1.61381900
H	-15.50737000	-2.75767700	-0.18840600
C	-10.85343200	1.37303200	-1.82694000
C	-9.72156200	1.93674100	-2.43010100
C	-12.13678000	1.77898300	-2.21262400
C	-9.87476900	2.91354600	-3.40557200
H	-8.72625400	1.61072000	-2.14533100
C	-12.27868500	2.75748900	-3.19096600
H	-13.01712700	1.34115000	-1.75374800
C	-11.15266900	3.32337700	-3.78483000
H	-8.99819100	3.34786000	-3.87441900
H	-13.27181800	3.07535700	-3.48973100
H	-11.27044700	4.08349000	-4.55020400
C	-6.71914200	-0.30184800	1.72259000
C	-6.39137300	-0.97846000	0.54236600
C	-6.22178000	-0.26902100	-0.65670800
C	-6.38833000	1.11541500	-0.67586600
C	-6.71677300	1.78909800	0.50783400
C	-6.87333600	1.06862400	1.70366000
H	-6.83727000	-0.86224600	2.64358900
H	-5.90249100	-0.79055800	-1.55509800
H	-6.23769400	1.64660800	-1.60792200
H	-7.12925500	1.61235200	2.60520600
N	-6.11693900	-2.39779500	0.65770800
N	-5.73758200	-3.09402600	-0.21798500
O	-6.89716700	3.11459200	0.60405200
C	-6.78890400	3.90939100	-0.56844000
H	-6.97336400	4.93383900	-0.24831200
H	-5.78777800	3.83664900	-1.00569300
H	-7.54176200	3.62024700	-1.30949000
Cl	-9.26138600	-2.92526900	-2.72241900

Zero-point correction = 0.413630 (Hartree/Particle)
Thermal correction to Energy = 0.444113
Thermal correction to Enthalpy = 0.445057
Thermal correction to Gibbs Free Energy = 0.345555
Sum of electronic and zero-point Energies = -2086.600066
Sum of electronic and thermal Energies = -2086.569583
Sum of electronic and thermal Enthalpies = -2086.568639
Sum of electronic and thermal Free Energies = -2086.668140
E(M06L) = -2087.632874

^{3}Int1′

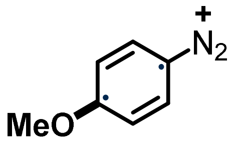

C	-0.88686500	-0.70439000	0.06561700
C	0.54572200	-0.68243900	0.01144600
C	1.17643400	0.52767900	-0.03680400
C	0.41581000	1.72261600	-0.03083900
C	-0.99983100	1.71578100	0.02206100
C	-1.64815100	0.51835600	0.06944500
H	1.11592600	-1.60241000	0.00885800
H	2.25709400	0.59611600	-0.07956300
H	-1.54479800	2.65241000	0.02340100
H	-2.72830100	0.44465900	0.11084600
N	1.16805400	2.94336400	-0.08558100
N	0.66355400	4.01623400	-0.05550200

O	-1.59660900	-1.78266300	0.11434100
C	-1.00076000	-3.10236800	0.11920900
H	-0.38359500	-3.21533600	1.01128700
H	-1.84550600	-3.78427300	0.14632500
H	-0.42030900	-3.24139700	-0.79352000

Zero-point correction = 0.131210 (Hartree/Particle)
Thermal correction to Energy = 0.140396
Thermal correction to Enthalpy = 0.141340
Thermal correction to Gibbs Free Energy = 0.094767
Sum of electronic and zero-point Energies = -455.091995
Sum of electronic and thermal Energies = -455.082809
Sum of electronic and thermal Enthalpies = -455.081865
Sum of electronic and thermal Free Energies = -455.128438
E(M06L) = -455.4416453

³TS1'

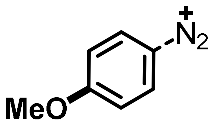

C	-0.90104800	-0.73317000	0.06237200
C	0.53361000	-0.71538500	0.02676100
C	1.16908300	0.49718500	-0.01364300
C	0.38085400	1.65714400	-0.01789200
C	-1.01657400	1.69045800	0.01653200
C	-1.66811800	0.48749700	0.05680600
H	1.09973600	-1.63838000	0.03214300
H	2.25066700	0.56798700	-0.04194800
H	-1.55558200	2.63200600	0.01065100
H	-2.74878000	0.40844900	0.08528600
N	1.32503400	3.23672700	-0.07865500
N	0.74419400	4.20674500	-0.09453700
O	-1.61305000	-1.81329600	0.10210900
C	-1.01667700	-3.13145400	0.11583000
H	-0.40783800	-3.24312400	1.01389600
H	-1.86066600	-3.81469500	0.13604700
H	-0.42697300	-3.27275400	-0.79072800

Zero-point correction = 0.128576 (Hartree/Particle)
Thermal correction to Energy = 0.138016
Thermal correction to Enthalpy = 0.138960
Thermal correction to Gibbs Free Energy = 0.092206
Sum of electronic and zero-point Energies = -455.078670
Sum of electronic and thermal Energies = -455.069230
Sum of electronic and thermal Enthalpies = -455.068286
Sum of electronic and thermal Free Energies = -455.115040
E(M06L) = -455.4323254

³Int2'

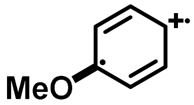

C	-0.90110900	-0.73054100	0.06135000
C	0.53356700	-0.71578100	0.02593500
C	1.17559900	0.49589600	-0.01342800
C	0.37457200	1.64528900	-0.01584800
C	-1.02032900	1.69604700	0.01696000
C	-1.66908300	0.48899800	0.05573100
H	1.09786300	-1.64015200	0.03067400
H	2.25750100	0.55781000	-0.04129600
H	-1.56305700	2.63412000	0.01211100
H	-2.74983800	0.40868000	0.08363300
O	-1.61396100	-1.81191100	0.10130700
C	-1.01508500	-3.12789200	0.11613400
H	-0.40482600	-3.23832500	1.01349700
H	-1.85724500	-3.81350100	0.13818200
H	-0.42592500	-3.27026900	-0.79071800

Zero-point correction = 0.122050 (Hartree/Particle)
Thermal correction to Energy = 0.128967
Thermal correction to Enthalpy = 0.129911
Thermal correction to Gibbs Free Energy = 0.090024
Sum of electronic and zero-point Energies = -345.622067
Sum of electronic and thermal Energies = -345.615151
Sum of electronic and thermal Enthalpies = -345.614206
Sum of electronic and thermal Free Energies = -345.654094
E(M06L) = -345.9079829

³TS2'

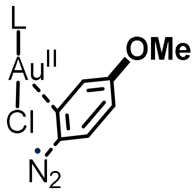

Au	-8.76127300	-1.76683500	-0.47034000
P	-10.49245400	-0.02923100	-0.30523400
C	-10.28727800	0.85893300	1.25195600
C	-10.17402200	2.25139400	1.30117500
C	-10.19390500	0.10560600	2.43276400
C	-9.98952400	2.88601300	2.52620900
H	-10.21861500	2.84478000	0.39475600
C	-10.01472800	0.74746300	3.65051400
H	-10.24911000	-0.97959100	2.40046400
C	-9.91308100	2.13858900	3.69787500
H	-9.89725600	3.96621500	2.55863700
H	-9.94509200	0.16239500	4.56121000
H	-9.76545400	2.63747200	4.65016500
C	-12.11739900	-0.82308300	-0.31843900
C	-12.52550600	-1.43455900	-1.51206900
C	-12.95736100	-0.83097000	0.79915500
C	-13.76917600	-2.04578300	-1.58350200
H	-11.87158800	-1.43619800	-2.37921200
C	-14.20205600	-1.44961100	0.71839100
H	-12.65798700	-0.34872900	1.72301700
C	-14.60682000	-2.05663900	-0.46721000
H	-14.08500600	-2.51687100	-2.50834900
H	-14.85568900	-1.45054600	1.58432800
H	-15.57793800	-2.53785300	-0.52484200
C	-10.48459400	1.12130000	-1.70187400
C	-9.54441500	1.01182200	-2.72847600
C	-11.47268100	2.11560400	-1.75835700
C	-9.56564700	1.91413900	-3.78824700
H	-8.80411600	0.21998300	-2.71571700
C	-11.48223200	3.01770100	-2.81493700
H	-12.23013100	2.18591400	-0.98302500
C	-10.52546500	2.92133100	-3.82547400
H	-8.83307600	1.82467400	-4.58325600
H	-12.24089100	3.79226600	-2.85224200
H	-10.53784600	3.62712600	-4.64975800
C	-6.95073800	-0.18833400	1.35261900
C	-6.84409400	-0.72623500	0.03869800
C	-6.61452200	0.14539400	-1.05949400
C	-6.75178400	1.50464900	-0.89912900
C	-6.99115100	2.02388900	0.39202700
C	-7.05521400	1.16478500	1.51799500
H	-6.98440200	-0.86079900	2.20349000
H	-6.36167800	-0.27203200	-2.03029600
H	-6.64963900	2.15797100	-1.75606900
H	-7.20888700	1.60648300	2.49562800
N	-5.86700000	-2.17294300	0.05222700

N	-5.37668800	-2.57469100	-0.89641000
O	-7.15217100	3.30919300	0.65019500
C	-7.20084500	4.24696400	-0.42912100
H	-7.42306400	5.20628700	0.03336500
H	-6.23657000	4.29532400	-0.94148900
H	-7.99522800	3.97800900	-1.13177600
Cl	-9.92473100	-3.79131000	-1.06659400

Zero-point correction = 0.411668 (Hartree/Particle)
Thermal correction to Energy = 0.441909
Thermal correction to Enthalpy = 0.442853
Thermal correction to Gibbs Free Energy = 0.345462
Sum of electronic and zero-point Energies = -2086.592794
Sum of electronic and thermal Energies = -2086.562553
Sum of electronic and thermal Enthalpies = -2086.561609
Sum of electronic and thermal Free Energies = -2086.659001
E(M06L) = -2087.626918

[3]Int3'

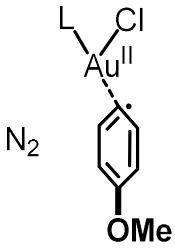

Au	-8.57399000	-1.18352400	-1.40180000
P	-10.69091800	0.18446800	-1.03698100
C	-10.32522600	1.49322500	0.15733900
C	-10.10627900	2.81360300	-0.24932700
C	-10.14560600	1.13540700	1.50116200
C	-9.71655900	3.76869600	0.68487600
H	-10.23900500	3.09901000	-1.28777900
C	-9.76096600	2.09511100	2.42847400
H	-10.29971300	0.10880400	1.82130100
C	-9.54366700	3.41229400	2.02045800
H	-9.55004300	4.79267700	0.36745700
H	-9.62658500	1.81585500	3.46848300
H	-9.24752300	4.16188900	2.74780200
C	-12.06740800	-0.79435400	-0.39023400
C	-12.21734900	-2.11993200	-0.81550900
C	-13.00374700	-0.22640300	0.48375800
C	-13.29979800	-2.86986700	-0.36765500
H	-11.49427100	-2.56207900	-1.49499600
C	-14.08207900	-0.98541900	0.92638000
H	-12.89604100	0.80132900	0.81526400
C	-14.22939700	-2.30501600	0.50322100
H	-13.41349500	-3.89758300	-0.69658100
H	-14.80716800	-0.54390300	1.60212400
H	-15.07064300	-2.89451800	0.85381100
C	-11.19115400	0.93372100	-2.60552900
C	-10.20573400	1.41294800	-3.47821000
C	-12.54336400	1.03855400	-2.94857300
C	-10.57515200	2.01214400	-4.67703400
H	-9.15306600	1.31581800	-3.22726100
C	-12.90366400	1.63292900	-4.15419900
H	-13.31159900	0.66025500	-2.28182100
C	-11.92319800	2.12052600	-5.01542300
H	-9.81038300	2.38533400	-5.35009100
H	-13.95265400	1.71340600	-4.41976300
H	-12.20932000	2.58148800	-5.95545100
C	-7.33834300	-0.82108000	1.32890300
C	-7.55474700	-0.21526400	0.05640300
C	-7.11265400	1.13176300	-0.13351600
C	-6.61933200	1.88238700	0.89669300
C	-6.50528900	1.28211200	2.18334900
C	-6.84715200	-0.09506700	2.37545300
H	-7.60916700	-1.85983900	1.48234700
H	-7.20563500	1.58811100	-1.11309300
H	-6.32990200	2.91326300	0.73765300
H	-6.72041200	-0.51714300	3.36598700
N	-4.79927500	-1.31915400	-1.77865200
N	-4.93391400	-2.22782900	-1.17116000
O	-6.08760900	1.90332500	3.25244800
C	-5.73221400	3.29723400	3.20617100
H	-5.48157400	3.55864800	4.23088400
H	-4.86771300	3.43821900	2.55484600
H	-6.58508000	3.88518700	2.86085500
Cl	-9.34683900	-2.56174200	-3.23054500

Zero-point correction = 0.411509 (Hartree/Particle)
Thermal correction to Energy = 0.443687
Thermal correction to Enthalpy = 0.444632
Thermal correction to Gibbs Free Energy = 0.339155
Sum of electronic and zero-point Energies = -2086.647741
Sum of electronic and thermal Energies = -2086.615562
Sum of electronic and thermal Enthalpies = -2086.614618
Sum of electronic and thermal Free Energies = -2086.720095
E(M06L) = -2087.676346

[2]Int4'

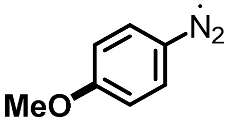

C	-0.96224200	-0.72554300	0.04508400
C	0.43452500	-0.65115600	0.05535600
C	1.05214700	0.59619700	0.03119700
C	0.28766400	1.75421000	-0.00300100
C	-1.11018000	1.68660700	-0.01424000
C	-1.72889400	0.45272600	0.00966400
H	1.04626100	-1.54458000	0.08135500
H	2.13470100	0.67235100	0.03894200
H	-1.69577500	2.59995900	-0.04109700
H	-2.81060100	0.36916100	0.00199000
N	0.99917500	3.00883800	-0.02616900
N	0.49899200	4.08002000	-0.05635000
O	-1.66577500	-1.87514500	0.06604500
C	-0.95327700	-3.10137600	0.10597200
H	-0.33707000	-3.17187100	1.00915800
H	-1.71009500	-3.88494800	0.12133300
H	-0.32133200	-3.22141400	-0.78106100

Zero-point correction = 0.132102 (Hartree/Particle)
Thermal correction to Energy = 0.140829
Thermal correction to Enthalpy = 0.141773
Thermal correction to Gibbs Free Energy = 0.097565
Sum of electronic and zero-point Energies = -455.322221
Sum of electronic and thermal Energies = -455.313493
Sum of electronic and thermal Enthalpies = -455.312549
Sum of electronic and thermal Free Energies = -455.356758
E(M06L) = -455.6767421

[2]TS4'

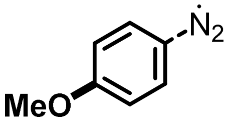

C	-0.97616700	-0.76112500	0.04692200
C	0.42149700	-0.69225100	0.05752300
C	1.05123700	0.55778000	0.03252400
C	0.25531600	1.67495700	-0.00180900

C	-1.12405700	1.65643800	-0.01355500
C	-1.74526800	0.41315600	0.01135500
H	1.02897000	-1.58938400	0.08464900
H	2.13452300	0.63135900	0.04026400
H	-1.70556900	2.57412100	-0.04107200
H	-2.82740400	0.32766200	0.00387500
N	1.16838800	3.38529500	-0.03556800
N	0.53400400	4.31681000	-0.06349700
O	-1.68350300	-1.91682700	0.06906700
C	-0.96808100	-3.13868400	0.10611200
H	-0.35032500	-3.21201300	1.00888700
H	-1.72127300	-3.92646200	0.11898300
H	-0.33406600	-3.25679500	-0.78048200

Zero-point correction = 0.128720 (Hartree/Particle)
Thermal correction to Energy = 0.137963
Thermal correction to Enthalpy = 0.138907
Thermal correction to Gibbs Free Energy = 0.092923
Sum of electronic and zero-point Energies = -455.302597
Sum of electronic and thermal Energies = -455.293355
Sum of electronic and thermal Enthalpies = -455.292411
Sum of electronic and thermal Free Energies = -455.338394
E(M06L) = -455.6610448

^{2}Int5’

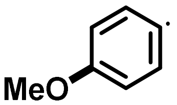

C	-1.03289100	3.33777000	2.39694200
C	-1.91972200	4.28578500	2.84402200
C	-0.35815600	2.44807100	3.20873000
C	-2.15914400	4.35648900	4.22514600
H	-2.43060700	4.96825300	2.17107300
C	-0.60157400	2.52485600	4.57859300
H	0.33658800	1.71226900	2.81470300
C	-1.49934300	3.47550800	5.08725700
H	-2.85559800	5.09741100	4.60108600
H	-0.10343200	1.85412200	5.27220600
O	-1.66041700	3.46034600	6.43622200
C	-2.55270300	4.39630300	7.01028100
H	-3.57489600	4.25434400	6.63929900
H	-2.53101900	4.21290900	8.08464800
H	-2.23434600	5.42666100	6.81163200

Zero-point correction = 0.121966 (Hartree/Particle)
Thermal correction to Energy = 0.128709
Thermal correction to Enthalpy = 0.129653
Thermal correction to Gibbs Free Energy = 0.090374
Sum of electronic and zero-point Energies = -345.839997
Sum of electronic and thermal Energies = -345.833255
Sum of electronic and thermal Enthalpies = -345.832310
Sum of electronic and thermal Free Energies = -345.871590
E(M06L) = -346.1296417

^{2}Int6’

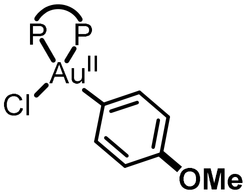

P	-2.11605700	0.64066300	0.06018500
C	-2.15300900	0.01004600	-1.67555600
C	-3.82189000	1.15314500	0.43018500
C	-1.74672600	-0.74616900	1.16318700
C	-2.16877900	1.18124800	-2.66790200
H	-1.25564500	-0.59907700	-1.83131100
H	-3.02766800	-0.63044500	-1.82499000
C	-4.21044200	2.47452700	0.17977700
C	-4.75753600	0.24377800	0.93748600
C	-1.51377600	-2.04534000	0.69951500
C	-1.65123800	-0.47267600	2.53628700
H	-2.25299700	0.81217700	-3.69381400
H	-3.03738600	1.82739800	-2.49538900
P	-0.67651200	2.25653200	-2.46479700
C	-5.52058600	2.87800500	0.42229400
H	-3.48966600	3.19744500	-0.19287300
C	-6.06412600	0.65291200	1.18449100
H	-4.46548200	-0.78180900	1.14301800
C	-1.19165500	-3.05862100	1.59967700
H	-1.59001600	-2.28161000	-0.35673700
C	-1.33408400	-1.49007400	3.42812400
H	-1.82191200	0.53405800	2.90720300
C	-0.96379300	3.65033800	-3.59666100
C	0.72415200	1.29306900	-3.11755800
C	-6.44707800	1.96826000	0.92594300
H	-5.81233500	3.90472600	0.22584800
H	-6.78401600	-0.05674200	1.57987500
C	-1.10106200	-2.78350200	2.96166400
H	-1.01586800	-4.06492400	1.23280500
H	-1.26239100	-1.26943200	4.48857500
C	-1.69489300	4.74436500	-3.11935400
C	-0.50455300	3.64639700	-4.91682200
C	1.99760400	1.54956200	-2.59353900
C	0.57138800	0.31506900	-4.10735500
H	-7.46686600	2.28459000	1.12200700
H	-0.84887500	-3.57574200	3.65971300
C	-1.97719000	5.81613300	-3.96019900
H	-2.03568600	4.76290500	-2.08704200
C	-0.78199900	4.72521100	-5.75234200
H	0.07260700	2.80752600	-5.29407300
C	3.10085100	0.83992300	-3.05850700
H	2.12163500	2.30986200	-1.82620800
C	1.67732200	-0.39630700	-4.56461500
H	-0.40428500	0.09905800	-4.53098100
C	-1.51930400	5.80753900	-5.27672900
H	-2.54514100	6.66135100	-3.58451100
H	-0.42014900	4.71942600	-6.77573200
C	2.94164900	-0.13483000	-4.04122400
H	4.08444900	1.04522600	-2.64787300
H	1.54887200	-1.15519600	-5.32982200
H	-1.73252200	6.64764700	-5.93043600
H	3.80205100	-0.69248500	-4.39806300
C	-0.49803200	2.78740300	2.02556500
C	-1.61859600	3.20870500	2.74533000
C	0.58836300	2.29849700	2.76786800
C	-1.67523600	3.14809500	4.14217000
H	-2.49868600	3.57578100	2.22217900
C	0.55116300	2.22290200	4.15461400
H	1.48545800	1.95245900	2.25941100
C	-0.58583900	2.64343700	4.85279000
H	-2.57132900	3.48589000	4.65124900
H	1.39438600	1.83283400	4.71761400
O	-0.53298500	2.52192100	6.20743900
C	-1.67246200	2.91010900	6.94910700
H	-2.55310800	2.31859800	6.66979600
H	-1.43026400	2.72327700	7.99579800
H	-1.89619200	3.97548400	6.81429900
Au	-0.50814700	2.65449900	-0.05017600
Cl	1.41436100	4.63648300	-0.45130800

Zero-point correction = 0.556305 (Hartree/Particle)
Thermal correction to Energy = 0.593486
Thermal correction to Enthalpy = 0.594430

Thermal correction to Gibbs Free Energy = 0.478878
Sum of electronic and zero-point Energies = -2628.749143
Sum of electronic and thermal Energies = -2628.711962
Sum of electronic and thermal Enthalpies = -2628.711018
Sum of electronic and thermal Free Energies = -2628.826570
E(M06L) = -2630.015603

[1]Int7'

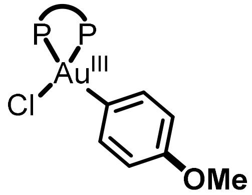

P	-2.18784600	0.65492700	-0.05351000
C	-2.39406900	0.20083400	-1.83236000
C	-3.77479700	1.13673800	0.65240800
C	-1.54263000	-0.81447300	0.77348100
C	-2.23508200	1.37775600	-2.79619000
H	-1.62393500	-0.55399700	-2.02419500
H	-3.36180100	-0.29564000	-1.95335200
C	-4.84209000	1.48616900	-0.18177900
C	-3.91312200	1.22585200	2.04469800
C	-2.37233300	-1.64065800	1.53643100
C	-0.19699900	-1.15414900	0.57736300
H	-2.29461800	1.02628600	-3.82961200
H	-3.02263300	2.12619100	-2.66158900
P	-0.63454600	2.23404000	-2.47340000
C	-6.04177900	1.91736500	0.37523000
H	-4.75573700	1.42578000	-1.26130900
C	-5.11716400	1.65153300	2.59088800
H	-3.08942100	0.96544800	2.70167300
C	-1.84959100	-2.79496600	2.11314200
H	-3.41862300	-1.39475400	1.68212400
C	0.31439900	-2.30894400	1.15518000
H	0.45481900	-0.51993700	-0.01760900
C	-0.66192900	3.78515300	-3.40316900
C	0.68381900	1.16314200	-3.10049500
C	-6.17935900	2.00040900	1.75852200
H	-6.86752800	2.18633000	-0.27470100
H	-5.22056100	1.71652700	3.66885700
C	-0.51057200	-3.12667200	1.92696300
H	-2.49355400	-3.43394700	2.70798100
H	1.35744800	-2.56710600	1.00650900
C	-1.54239900	4.78674700	-2.97386200
C	0.17094300	4.00665300	-4.50211000
C	1.85134000	1.00856700	-2.34310000
C	0.55060600	0.48717400	-4.31997400
H	-7.11648500	2.33775300	2.18952800
H	-0.10731200	-4.02548800	2.38215500
C	-1.60008900	5.99559800	-3.65667400
H	-2.17518500	4.62828400	-2.10388100
C	0.11187600	5.22363500	-5.17710200
H	0.86374500	3.23912300	-4.83230500
C	2.87160500	0.17933400	-2.79930700
H	1.96772700	1.53455000	-1.39960200
C	1.57429500	-0.33994500	-4.76981900
H	-0.34233900	0.60424900	-4.92651400
C	-0.77228000	6.21420800	-4.75788500
H	-2.28380900	6.76991700	-3.32439700
H	0.75971200	5.39548200	-6.03052800
C	2.73206000	-0.49586300	-4.00941600
H	3.77278600	0.06017400	-2.20699500
H	1.46618800	-0.86321400	-5.71410800
H	-0.81387200	7.16161600	-5.28595900
H	3.52687100	-1.14544900	-4.36197700
C	-0.89642700	2.49797100	2.01503800
C	-1.70006900	3.49812300	2.55045500
C	-0.31304100	1.55817300	2.86436500
C	-1.93856100	3.56448300	3.92347100
H	-2.17132400	4.23579200	1.90708900
C	-0.54663600	1.61253200	4.23275200
H	0.31374000	0.76306700	2.47215500
C	-1.36747000	2.61095100	4.77014000
H	-2.57495200	4.35222900	4.30938400
H	-0.10446500	0.88125900	4.90193700
O	-1.54644600	2.56915200	6.11231400
C	-2.40249100	3.53239300	6.70028500
H	-3.41893100	3.46136200	6.29515500
H	-2.42133400	3.30594300	7.76631600
H	-2.02099500	4.54942300	6.55221500
Au	-0.65161400	2.43322500	-0.03218800
Cl	0.93711200	4.25708900	0.02335700Zero-point

correction = 0.559430 (Hartree/Particle)
Thermal correction to Energy = 0.595424
Thermal correction to Enthalpy = 0.596368
Thermal correction to Gibbs Free Energy = 0.487254
Sum of electronic and zero-point Energies = -2628.611216
Sum of electronic and thermal Energies = -2628.575223
Sum of electronic and thermal Enthalpies = -2628.574279
Sum of electronic and thermal Free Energies = -2628.683392
E(M06L) = -2629.879482

$[Au(dppe)_2]^+$

C	-0.81795400	0.42761000	-1.47694600
C	0.09595900	-0.66661600	-0.90515800
H	-0.22619700	1.16178500	-2.03611200
H	-1.31326300	0.95629600	-0.65703100
H	0.82700600	-0.22162500	-0.22224900
H	-0.49978900	-1.38970500	-0.33597000
C	1.89946300	-2.92005100	-1.42575700
C	2.70154200	-3.74170400	-2.22917400
C	1.80383200	-3.18884000	-0.05796700
C	3.39656700	-4.80931100	-1.67533800
H	2.78957300	-3.53883600	-3.29335600
C	2.49608500	-4.26638200	0.49452900
H	1.19656000	-2.56587300	0.59016900
C	3.29076200	-5.07682800	-0.31047800
H	4.01603100	-5.43622000	-2.30896000
H	2.41371900	-4.46672700	1.55828800
H	3.82658100	-5.91568900	0.12262700
C	2.23148500	-0.40717700	-2.82448500
C	1.91260800	0.44079000	-3.89064100
C	3.47919800	-0.28823500	-2.20354400
C	2.82321200	1.39878600	-4.32796300
H	0.94492900	0.34686700	-4.37781000
C	4.39350300	0.66204200	-2.64953600
H	3.74082900	-0.94131300	-1.37586200
C	4.06709900	1.50627500	-3.71000000
H	2.56336000	2.05345700	-5.15432300
H	5.36266200	0.74414000	-2.16721300
H	4.78323800	2.24563300	-4.05517100
C	-3.45883000	-0.79361200	-1.57891800
C	-4.59783700	-0.01413400	-1.35831100
C	-3.36434600	-2.05226200	-0.97240400
C	-5.62150700	-0.48592700	-0.53946700
H	-4.69495800	0.95906000	-1.82992100
C	-4.37968300	-2.51485600	-0.14243200
H	-2.50342200	-2.68528500	-1.17136200
C	-5.51310900	-1.73303500	0.07189300
H	-6.50585100	0.12364100	-0.38069700
H	-4.29436400	-3.49415500	0.31826400

H	-6.31412600	-2.09863500	0.70709600
C	-2.68436200	1.18403100	-3.58467200
C	-2.73960300	2.47129400	-3.03633800
C	-3.09306000	0.98514400	-4.90770800
C	-3.19608600	3.53931700	-3.80387700
H	-2.42867800	2.64698200	-2.01068600
C	-3.55548800	2.05243400	-5.67163500
H	-3.03398700	-0.00632900	-5.34798300
C	-3.60431700	3.33071800	-5.12048400
H	-3.23220800	4.53504900	-3.37307000
H	-3.85937500	1.88440000	-6.69969500
H	-3.95603600	4.16665700	-5.71736900
P	-2.08079700	-0.26068800	-2.65653300
P	0.96388100	-1.59592200	-2.25630700
Au	-0.87731100	-2.06939200	-3.82728100
P	-1.94136700	-4.29081600	-3.99683400
C	-1.78920100	-4.72322200	-5.79951300
C	-3.74116000	-4.33715600	-3.67455300
C	-1.22500300	-5.71474800	-3.11815400
C	-1.95191100	-3.49256600	-6.70391800
H	-0.79637500	-5.16704600	-5.93814300
H	-2.53113400	-5.47953200	-6.07261000
C	-4.49090700	-3.19718900	-3.98983400
C	-4.38554400	-5.44311600	-3.11295100
C	-1.27580800	-7.02108300	-3.61991200
C	-0.58518700	-5.47439200	-1.89757200
H	-1.85285500	-3.78619800	-7.75401700
H	-2.94968200	-3.05949500	-6.56807400
P	-0.70877500	-2.18029600	-6.28573600
C	-5.86366700	-3.17076700	-3.76889400
H	-3.99413200	-2.31555700	-4.38681200
C	-5.75813100	-5.40894600	-2.87705800
H	-3.81922700	-6.33132400	-2.84966900
C	-0.69394000	-8.06667400	-2.90838800
H	-1.76871200	-7.22910800	-4.56510100
C	-0.00979300	-6.52153500	-1.18409800
H	-0.52188200	-4.46090200	-1.51119100
C	-1.10788200	-0.75868800	-7.35269600
C	0.84776300	-2.85871300	-6.96393200
C	-6.49919000	-4.27652400	-3.20753500
H	-6.43159600	-2.27896800	-4.01522900
H	-6.24793400	-6.27014600	-2.43295300
C	-0.06164500	-7.81786900	-1.69102700
H	-0.73400300	-9.07606400	-3.30578600
H	0.49221500	-6.31795400	-0.24385600
C	-2.23464900	-0.70801600	-8.17740400
C	-0.26513800	0.35858100	-7.27812700
C	1.65985500	-3.62309900	-6.11897700
C	1.22496700	-2.67901200	-8.29877000
H	-7.56828600	-4.25253400	-3.01983300
H	0.39455500	-8.63506600	-1.14069000
C	-2.51521300	0.44474900	-8.91085700
H	-2.90169600	-1.55949000	-8.26137000
C	-0.54220000	1.50267200	-8.01566500
H	0.61837400	0.32941100	-6.64582200
C	2.82960200	-4.20490500	-6.60011400
H	1.37061800	-3.76144700	-5.07977900
C	2.40111000	-3.25124900	-8.77546800
H	0.60452900	-2.08713500	-8.96564500
C	-1.67278200	1.54941400	-8.83126100
H	-3.39397700	0.47319200	-9.54759900
H	0.12054300	2.35985600	-7.95017800
C	3.20348400	-4.01449300	-7.92852900
H	3.45032100	-4.79882900	-5.93609300
H	2.69088000	-3.10162600	-9.81098500
H	-1.89523000	2.44559400	-9.40199400
H	4.12021600	-4.45857100	-8.30412000

Zero-point correction = 0.862896 (Hartree/Particle)
Thermal correction to Energy = 0.915676
Thermal correction to Enthalpy = 0.916621
Thermal correction to Gibbs Free Energy = 0.768742
Sum of electronic and zero-point Energies = -3509.697640
Sum of electronic and thermal Energies = -3509.644859
Sum of electronic and thermal Enthalpies = -3509.643915
Sum of electronic and thermal Free Energies = -3509.791793
E(M06L) = -3511.546419

Cl^-

Cl	-1.10982800	6.16592900	1.42396900

Zero-point correction = 0.000000 (Hartree/Particle)
Thermal correction to Energy = 0.001416
Thermal correction to Enthalpy = 0.002360
Thermal correction to Gibbs Free Energy = -0.015023
Sum of electronic and zero-point Energies = -460.347818
Sum of electronic and thermal Energies = -460.346402
Sum of electronic and thermal Enthalpies = -460.345458
Sum of electronic and thermal Free Energies = -460.362841
E(M06L) = -460.383708

dppe

C	-1.51928600	-0.15518300	0.51484500
C	-0.01577200	-0.29856100	0.77789400
H	-1.82281100	0.89761500	0.47815500
H	-2.09119800	-0.61266500	1.32996800
H	0.21219700	-1.32928800	1.07192900
H	0.56112300	-0.07440400	-0.12786900
P	0.52290100	0.84427300	2.14860200
P	-2.04794600	-1.01860600	-1.04533100
C	0.42271000	2.43252500	1.21377800
C	1.37765200	2.82198700	0.26660900
C	-0.71324500	3.23046400	1.39204800
C	1.19589800	3.97659600	-0.48877400
H	2.26586400	2.21524500	0.11293800
C	-0.90620500	4.37754000	0.62289800
H	-1.45881400	2.94900300	2.13175600
C	0.04881900	4.75198900	-0.31871900
H	1.94493400	4.26672800	-1.21977400
H	-1.79854700	4.97970200	0.76501800
H	-0.09617100	5.64735600	-0.91575800
C	2.34273300	0.57792600	2.20634700
C	3.07189400	1.35746400	3.11631200
C	3.03146100	-0.35765200	1.42720700
C	4.45074600	1.21853900	3.23244300
H	2.55502400	2.08534500	3.73795500
C	4.41289900	-0.50643500	1.55188000
H	2.50184900	-0.97901400	0.71222400
C	5.12589900	0.28189000	2.45016300
H	4.99823400	1.83648600	3.93793500
H	4.93055200	-1.23890100	0.93951100
H	6.20158200	0.16676100	2.54334300
C	-3.82070300	-0.50591100	-1.07493700
C	-4.23100600	0.82318200	-1.24007500
C	-4.79461200	-1.49209100	-0.88698200
C	-5.58172900	1.15654100	-1.21483600
H	-3.49198600	1.60475400	-1.39691700
C	-6.14879900	-1.16013100	-0.85742600
H	-4.49298900	-2.52930200	-0.76373300
C	-6.54365200	0.16460600	-1.02228800
H	-5.88538600	2.19100100	-1.34627200
H	-6.89297600	-1.93717900	-0.71005500
H	-7.59769800	0.42538600	-1.00397400
C	-1.32375700	0.05354000	-2.36011300
C	-0.87229700	1.36588700	-2.16915400

C	-1.22764400	-0.50377600	-3.64238400
C	-0.34778800	2.09919900	-3.23141900
H	-0.91776500	1.83510200	-1.19096700
C	-0.71594300	0.23200900	-4.70803000
H	-1.55650600	-1.52702600	-3.80889700
C	-0.27217700	1.53706700	-4.50338900
H	0.00170500	3.11252500	-3.05708600
H	-0.65513900	-0.21698500	-5.69496900
H	0.13585500	2.11070900	-5.33024900

Zero-point correction = 0.429631 (Hartree/Particle)
Thermal correction to Energy = 0.454309
Thermal correction to Enthalpy = 0.455253
Thermal correction to Gibbs Free Energy = 0.371353
Sum of electronic and zero-point Energies = -1687.124064
Sum of electronic and thermal Energies = -1687.099387
Sum of electronic and thermal Enthalpies = -1687.098442
Sum of electronic and thermal Free Energies = -1687.182343
E(M06L) = -1688.047522

[Ru^{II}(bpy)$_3$]*

N	-0.67336300	-1.75160900	-0.98586100
N	1.79489200	0.32592300	-1.02048000
N	-1.13707000	1.36959200	-1.03606200
C	-0.02162100	-2.36162100	-1.98431900
H	0.89036100	-1.88739700	-2.32698500
C	-0.48908800	-3.53316900	-2.55897600
H	0.06428000	-3.99353900	-3.36792600
C	-1.66583500	-4.08856300	-2.07177500
H	-2.06000600	-5.00606800	-2.49363200
C	-2.33840000	-3.45836500	-1.03140300
H	-3.25200600	-3.88757600	-0.64141800
C	-1.82057800	-2.28200200	-0.50265200
C	-2.45036800	-1.52792600	0.60293900
C	-2.26594400	0.33311200	2.00344100
H	-1.67389400	1.19637500	2.28100100
C	-3.46338300	0.03240000	2.63209700
C	-4.16845800	-1.09125900	2.21874900
H	-5.11034300	-1.35483200	2.68613700
C	-3.65888100	-1.87851100	1.19250500
H	-4.20593900	-2.75042900	0.85873700
C	1.93543700	1.21623300	-2.01243700
H	1.04047500	1.75055600	-2.30656800
C	3.15677800	1.43602600	-2.62873400
H	3.23119600	2.16133300	-3.42928800
C	4.26144300	0.71412500	-2.19380000
H	5.23315500	0.86267400	-2.65074900
C	4.11451600	-0.20286900	-1.15934700
H	4.97137600	-0.76411300	-0.81027700
C	2.86308800	-0.38183500	-0.58255200
C	2.59191200	-1.31850600	0.52920000
C	3.54688400	-2.16348000	1.08210900
H	4.56290100	-2.17171800	0.70991600
C	3.18329500	-3.00885700	2.12387500
H	3.91886600	-3.67218800	2.56431700
C	1.87367100	-2.99587100	2.58802900
C	0.96812700	-2.13294800	1.99139100
H	-0.06423900	-2.08230500	2.31613300
C	-1.99618400	1.08049600	-2.04157300
H	-2.02936900	0.04178600	-2.35114700
C	-2.76460600	2.03316100	-2.65620500
H	-3.42544000	1.75698900	-3.46821700
C	-2.66363600	3.37586200	-2.20113300
H	-3.25984600	4.15356300	-2.66679700
C	-1.82121100	3.68527300	-1.17044100
H	-1.75095000	4.70537500	-0.81181100
C	-1.03755300	2.66976100	-0.55391000
C	-0.15734900	2.86063100	0.53383800
C	1.36526600	1.80907600	2.02851400
H	1.82216900	0.87673700	2.34115000
C	1.67024900	2.99441400	2.64326100
C	1.02703800	4.17580500	2.18419300
H	1.24759200	5.13051500	2.65003500
C	0.13615300	4.10954600	1.14994600
H	-0.34759000	5.00944600	0.78884000
H	2.38245900	3.01599900	3.45851000
H	1.55045800	-3.63989700	3.39627000
H	-3.82822000	0.67283700	3.42529000
Ru	-0.00413500	0.00394100	-0.00543200
N	0.46809000	1.71772900	1.01902700
N	1.32094600	-1.31310700	0.99286700
N	-1.77250600	-0.43249200	1.02024000

Zero-point correction = 0.489379 (Hartree/Particle)
Thermal correction to Energy = 0.518362
Thermal correction to Enthalpy = 0.519306
Thermal correction to Gibbs Free Energy = 0.428245
Sum of electronic and zero-point Energies = -1578.844081
Sum of electronic and thermal Energies = -1578.815098
Sum of electronic and thermal Enthalpies = -1578.814153
Sum of electronic and thermal Free Energies = -1578.905215
E(M06L) = -1580.048677

[Ru^{III}(bpy)$_3$]

N	-0.65222600	-1.73226200	-0.99068000
N	1.79578600	0.30094500	-1.03976600
N	-1.18776100	1.40895800	-1.00637400
C	0.00950600	-2.32369300	-1.99655700
H	0.91776400	-1.83947700	-2.33293900
C	-0.44954300	-3.48973800	-2.58659100
H	0.10969700	-3.93451900	-3.39993400
C	-1.62357700	-4.05986400	-2.11027900
H	-2.01000700	-4.97408200	-2.54582700
C	-2.30407400	-3.44967800	-1.06284600
H	-3.21585800	-3.89039100	-0.68202900
C	-1.79786200	-2.27861800	-0.51511800
C	-2.43025300	-1.54679500	0.60029300
C	-2.25681700	0.30059100	2.02117000
H	-1.67819700	1.16760900	2.31363800
C	-3.44346100	-0.02377400	2.65714400
C	-4.13750300	-1.15161400	2.23690900
H	-5.07079200	-1.43359400	2.71031700
C	-3.62721500	-1.92028900	1.19692300
H	-4.16393200	-2.79694000	0.85934000
C	1.94717200	1.16665800	-2.05276700
H	1.06165200	1.70172400	-2.37201800
C	3.17201200	1.36155000	-2.66928400
H	3.25386200	2.06628300	-3.48704700
C	4.26942300	0.64233300	-2.21231800
H	5.24392000	0.77353800	-2.66823600
C	4.11170400	-0.25053900	-1.15830400
H	4.96227700	-0.81105900	-0.79357000
C	2.85747100	-0.40711900	-0.58346700
C	2.57007300	-1.31896200	0.54132700
C	3.51065500	-2.15961400	1.12120600
H	4.53116300	-2.17968200	0.76241700
C	3.12793500	-2.98595200	2.17172200
H	3.85384100	-3.64597400	2.63229000
C	1.81250600	-2.95916800	2.61799900
C	0.91831500	-2.10260600	1.99772200
H	-0.11627200	-2.04322500	2.31078700
C	-2.03384300	1.11083500	-2.00328200
H	-2.07642600	0.07388600	-2.31182300
C	-2.80787100	2.08081100	-2.61837200
H	-3.47515700	1.80178600	-3.42397300
C	-2.70335300	3.39428300	-2.17779800

H	-3.29518100	4.17861100	-2.63528100
C	-1.83228200	3.70000900	-1.13820000
H	-1.74809000	4.71966000	-0.78622300
C	-1.07933800	2.68507300	-0.56305000
C	-0.12869700	2.88644800	0.54816300
C	1.39026900	1.83715900	1.98200400
H	1.85802200	0.90883000	2.28461400
C	1.69069600	3.03533100	2.60845900
C	1.05129000	4.19099800	2.17730100
H	1.26235200	5.14582600	2.64471600
C	0.13384900	4.11682600	1.13530400
H	-0.36712800	5.01219100	0.79179200
H	2.41137500	3.05119600	3.41626900
H	1.47463800	-3.58871800	3.43133200
H	-3.80733600	0.60207600	3.46205300
Ru	-0.00262800	-0.00082800	-0.01463400
N	0.49906100	1.76606300	0.98217500
N	1.29125600	-1.29900200	0.99077200
N	-1.76180100	-0.44606800	1.02307600

Zero-point correction = 0.493486 (Hartree/Particle)
Thermal correction to Energy = 0.521935
Thermal correction to Enthalpy = 0.522879
Thermal correction to Gibbs Free Energy = 0.433530
Sum of electronic and zero-point Energies = -1578.713679
Sum of electronic and thermal Energies = -1578.685229
Sum of electronic and thermal Enthalpies = -1578.684285
Sum of electronic and thermal Free Energies = -1578.773634
E(M06L) = -1579.911815

[Ru^{II}(bpy)$_3$]

N	-0.68385900	-1.70225200	-1.02335100
N	1.78917500	0.26276600	-1.06462900
N	-1.14439200	1.41585900	-1.04369400
C	-0.07077000	-2.26729500	-2.07260200
H	0.83212600	-1.77777700	-2.41749200
C	-0.55602800	-3.40862500	-2.69263700
H	-0.02572900	-3.82798600	-3.53899200
C	-1.72081000	-3.98701000	-2.20318000
H	-2.13128000	-4.88068100	-2.65967700
C	-2.35825500	-3.40541000	-1.11482100
H	-3.26454600	-3.84883800	-0.72293500
C	-1.81942600	-2.25841500	-0.53930900
C	-2.42385000	-1.56324400	0.61991100
C	-2.21092200	0.19911800	2.12219200
H	-1.61852400	1.04807100	2.44127000
C	-3.38426400	-0.14939500	2.77306600
C	-4.09618100	-1.25137100	2.31503800
H	-5.01918800	-1.55479800	2.79611300
C	-3.61016800	-1.96534200	1.22713500
H	-4.15667100	-2.82452900	0.86021300
C	1.94507800	1.07843500	-2.11640800
H	1.05799600	1.60503100	-2.44710000
C	3.16414900	1.24489700	-2.75545100
H	3.24002100	1.91555800	-3.60269800
C	4.26442100	0.54024800	-2.28250900
H	5.23544200	0.64622800	-2.75301400
C	4.10708100	-0.30631900	-1.19256500
H	4.95756800	-0.85752800	-0.81255500
C	2.85433600	-0.43080600	-0.59861700
C	2.58201800	-1.30791400	0.56276000
C	3.53887600	-2.13403600	1.14548900
H	4.54791800	-2.17395300	0.75591800
C	3.18942300	-2.91867300	2.23710600
H	3.92597400	-3.56636300	2.69912200
C	1.88870800	-2.86084800	2.72259600
C	0.98321000	-2.01977800	2.09434200
H	-0.04194300	-1.93784900	2.43465800
C	-1.94319600	1.14303900	-2.08452300
H	-1.96649700	0.10985300	-2.40946800
C	-2.69597400	2.11786900	-2.72101500
H	-3.32600800	1.84844400	-3.55997400
C	-2.61840500	3.42565200	-2.25776400
H	-3.19234300	4.21603500	-2.72827500
C	-1.79274900	3.71230600	-1.17826100
H	-1.72536200	4.72687700	-0.80716300
C	-1.06178500	2.68688800	-0.58510300
C	-0.15131600	2.88919300	0.56472200
C	1.29183200	1.86143300	2.07142900
H	1.74300000	0.93343900	2.40149900
C	1.56954700	3.06482700	2.70137100
C	0.95428800	4.21868100	2.23118100
H	1.14594900	5.17888300	2.69673600
C	0.08520400	4.12915400	1.15138800
H	-0.39888200	5.02121800	0.77522800
H	2.25369400	3.08728800	3.54090600
H	1.57210300	-3.45455500	3.57149000
H	-3.72460800	0.43645400	3.61820900
Ru	-0.00075400	-0.00099500	0.00086500
N	0.45275300	1.77083300	1.03052200
N	1.31666400	-1.26068500	1.04154300
N	-1.73880800	-0.48706600	1.07241100

Zero-point correction = 0.492088 (Hartree/Particle)
Thermal correction to Energy = 0.520574
Thermal correction to Enthalpy = 0.521519
Thermal correction to Gibbs Free Energy = 0.432281
Sum of electronic and zero-point Energies = -1578.928339
Sum of electronic and thermal Energies = -1578.899852
Sum of electronic and thermal Enthalpies = -1578.899852
Sum of electronic and thermal Free Energies = -1578.988146
E(M06L) = -1580.125433

7. NMR Spectra

3,5-Dimethyl-4-(4-(methylsulfonyl)phenyl)isoxazole

1H NMR
(CDCl3, 600.44)

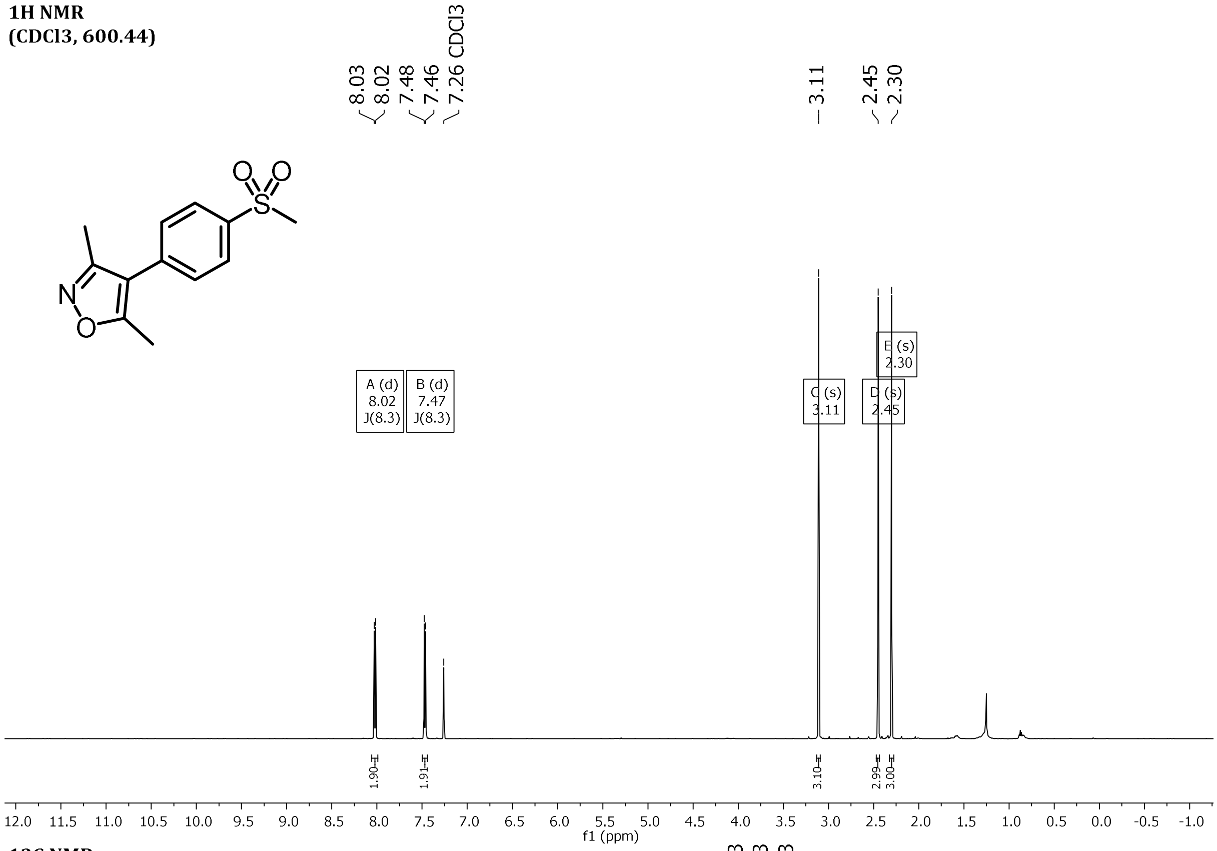

13C NMR
(CDCl3, 151.00 MHz)

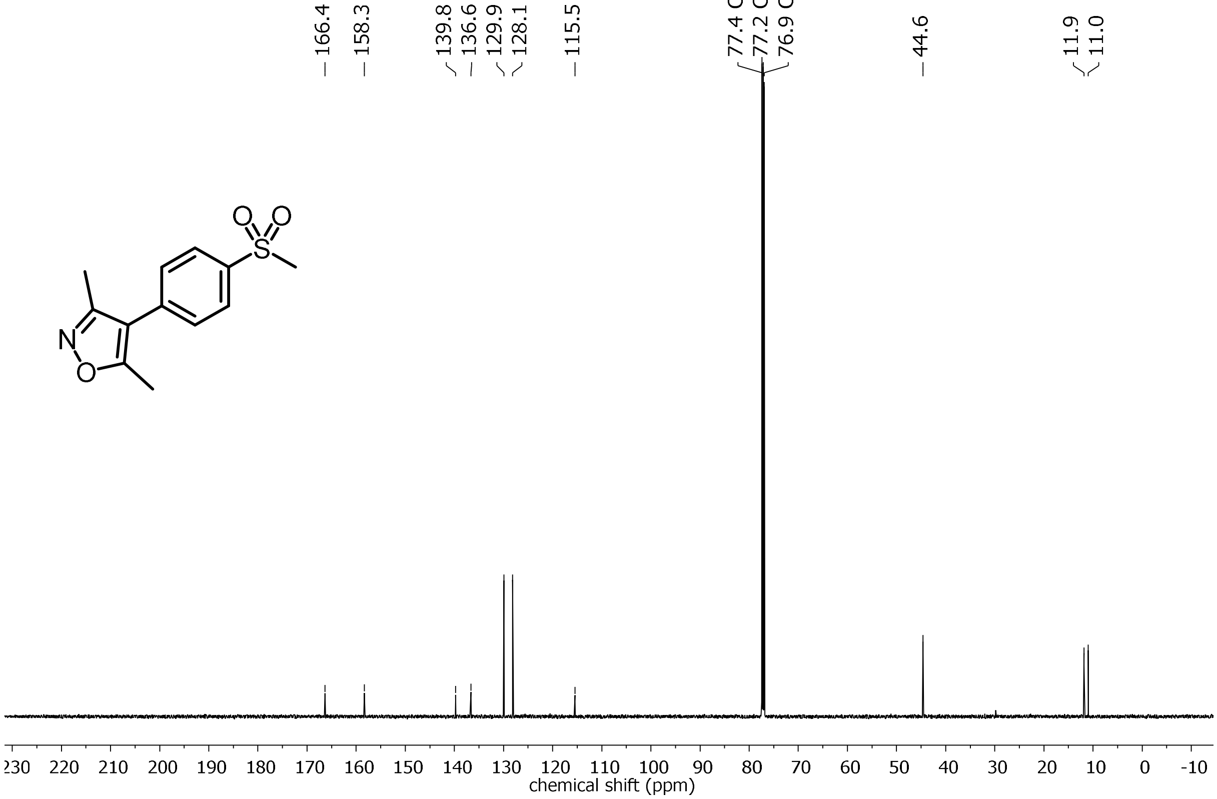

4'-Bromo-2-isopropyl-1,1'-biphenyl

1H NMR
(CDCl3, 399.97)

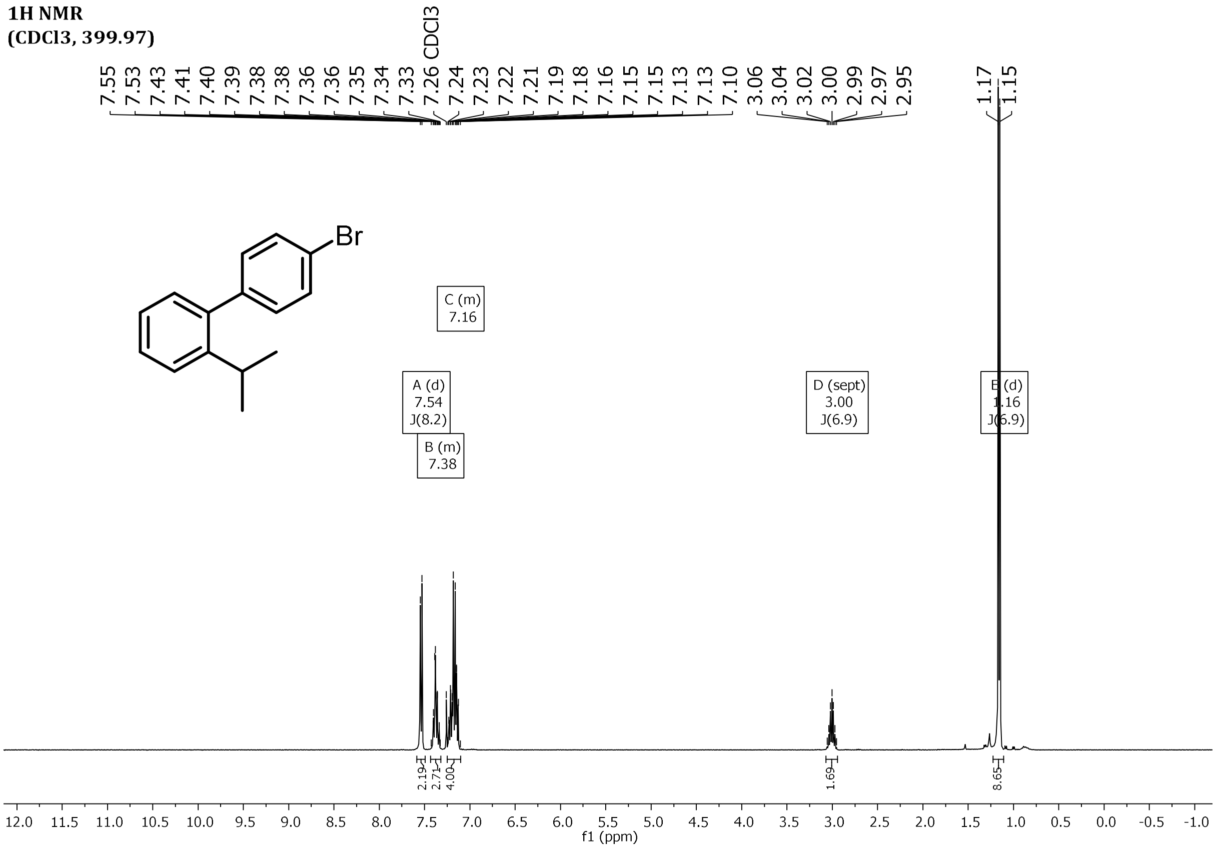

13C NMR
(CDCl3, 100.58 MHz)

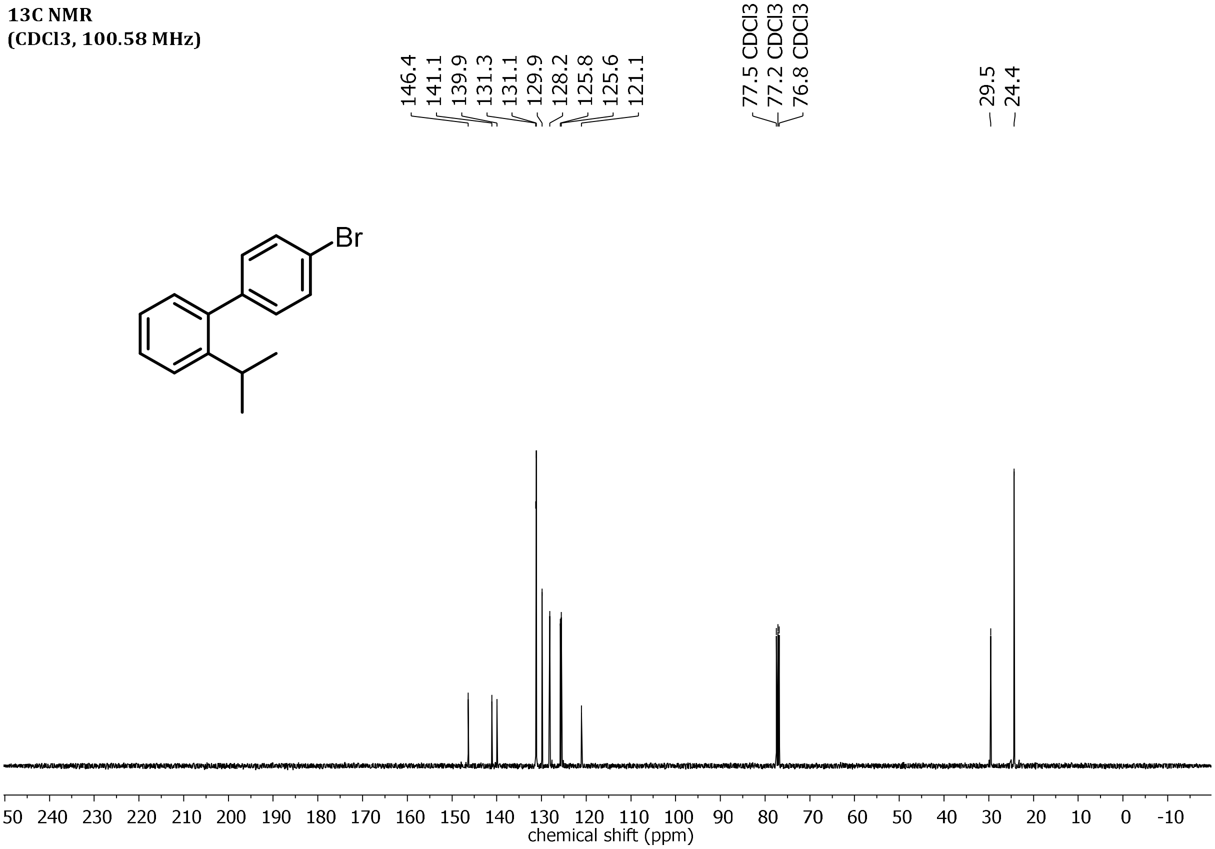

4-Bromo-3-methyl-4'-(trifluoromethyl)-1,1'-biphenyl

1H NMR
(600.44 MHz, CDCl3)

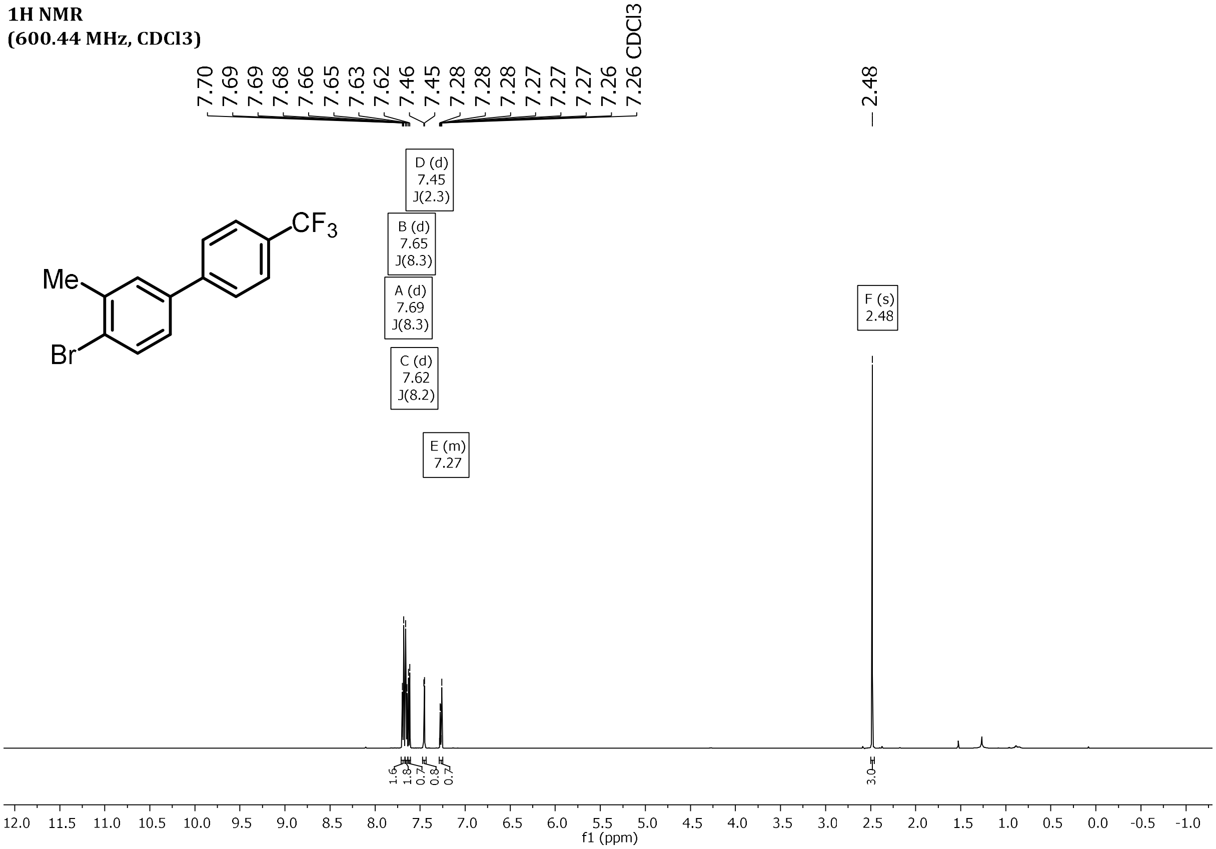

13C NMR
(151.00 MHz, CDCl3)

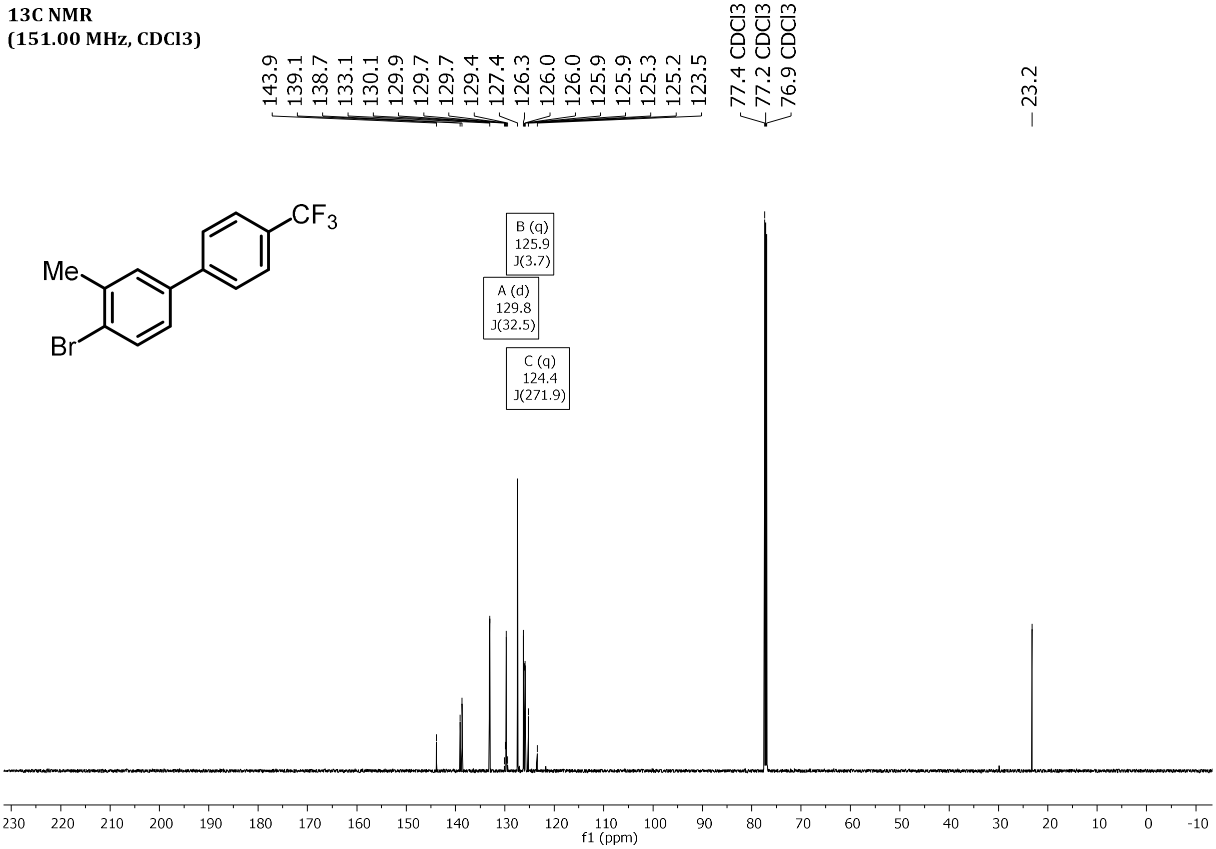

19F NMR
(564.92 MHz, CDCl3)

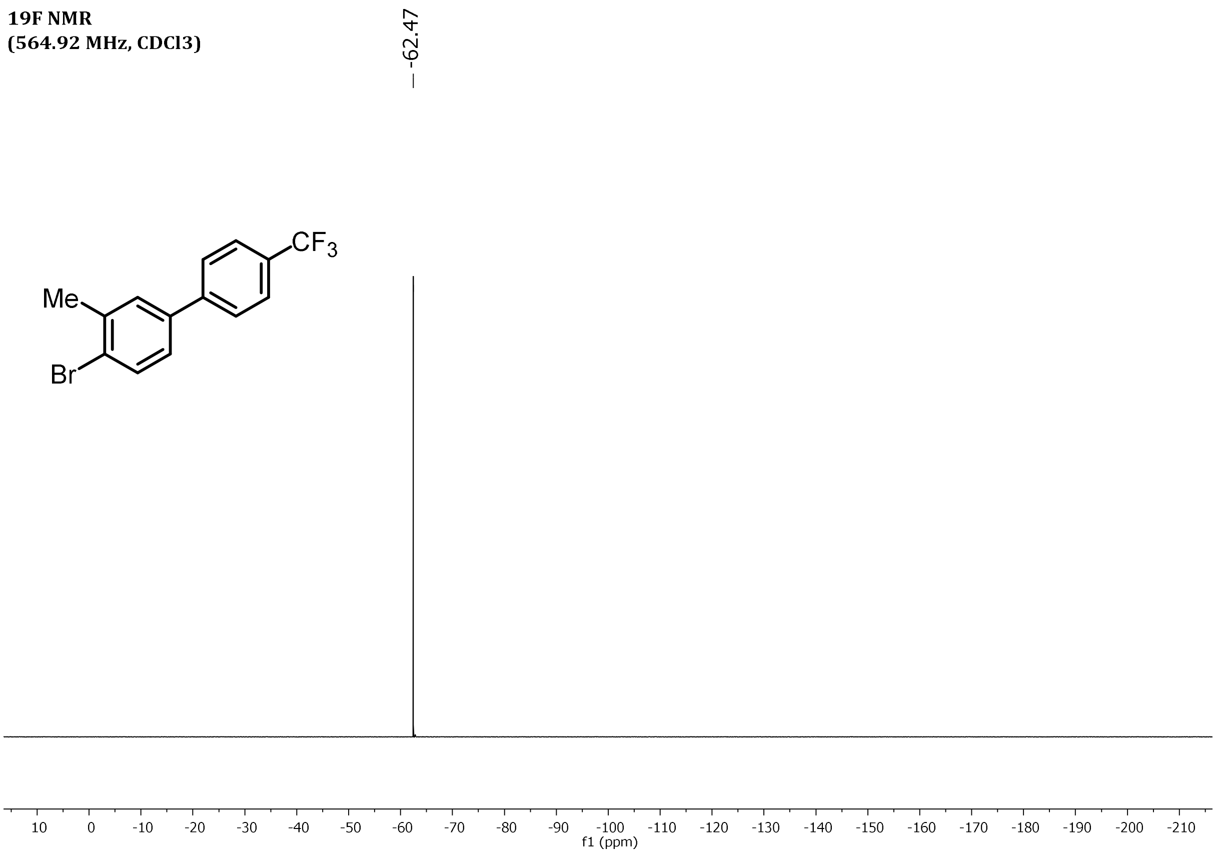

Trimethyl(4'-nitro-[1,1'-biphenyl]-4-yl)silane

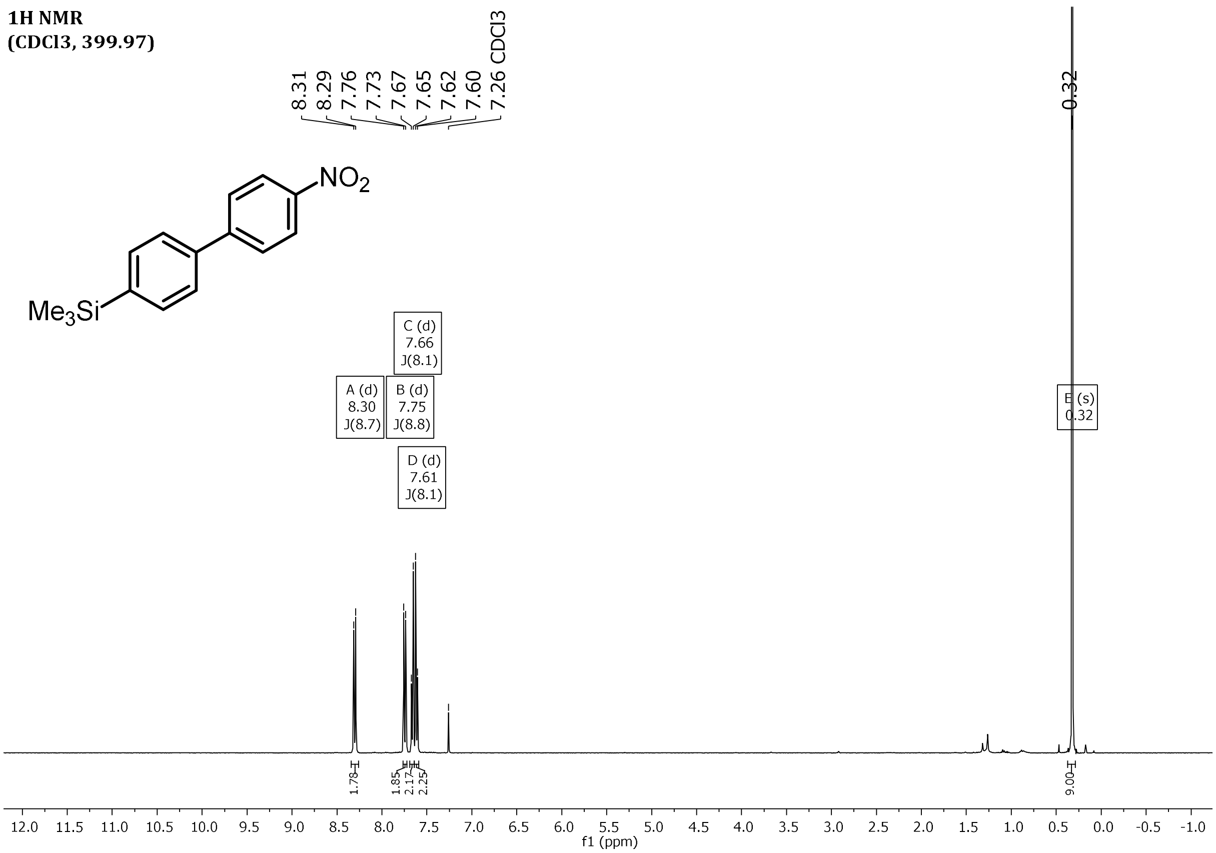

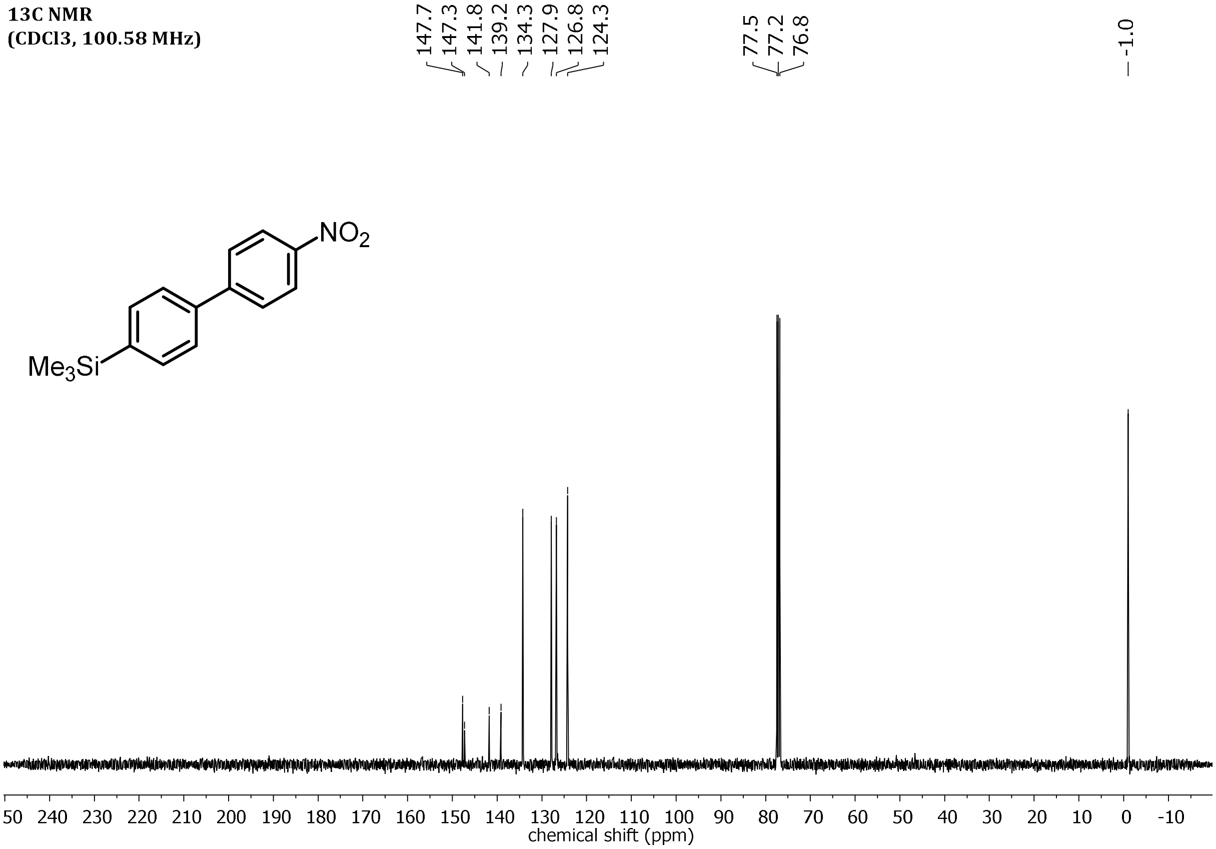

Trimethyl(4'-(trifluoromethyl)-[1,1'-biphenyl]-4-yl)silane

1H NMR
(CDCl3, 599.86)

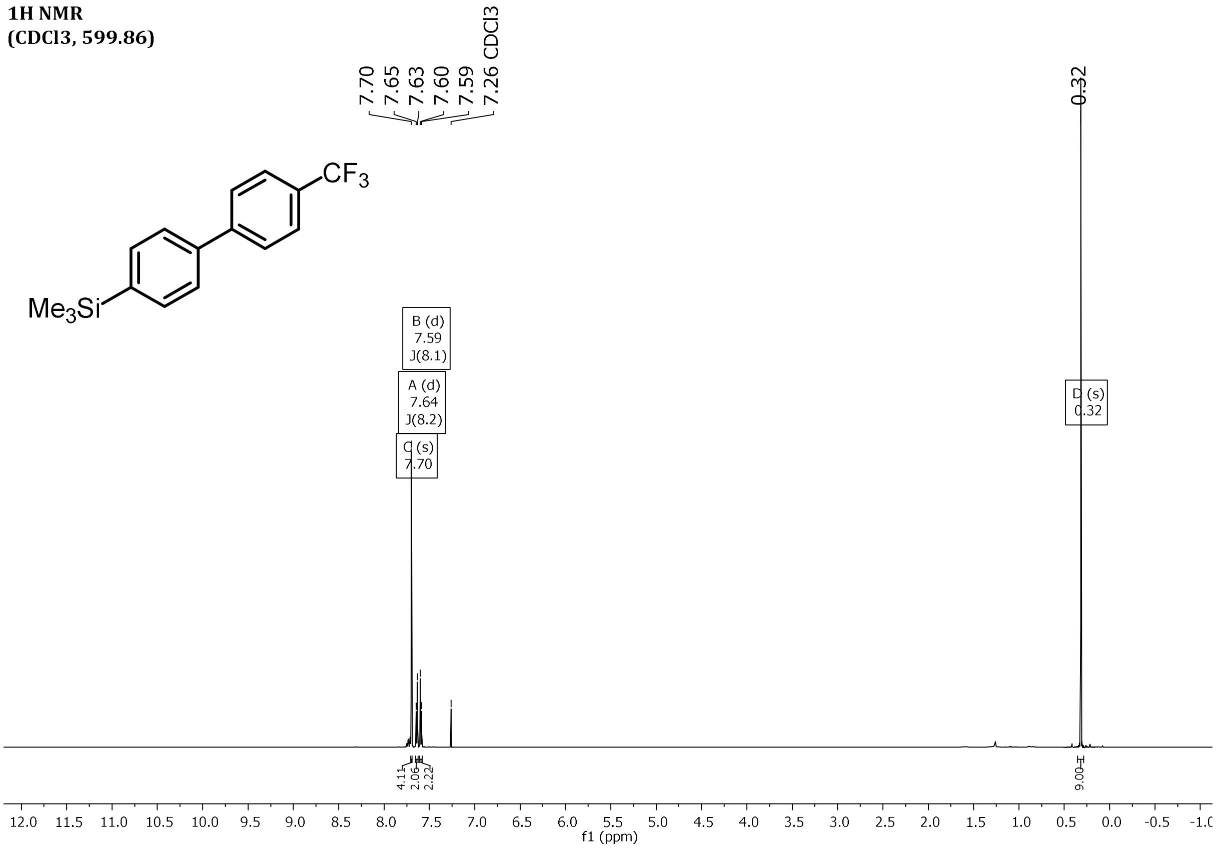

13C NMR
(CDCl3, 150.85 MHz)

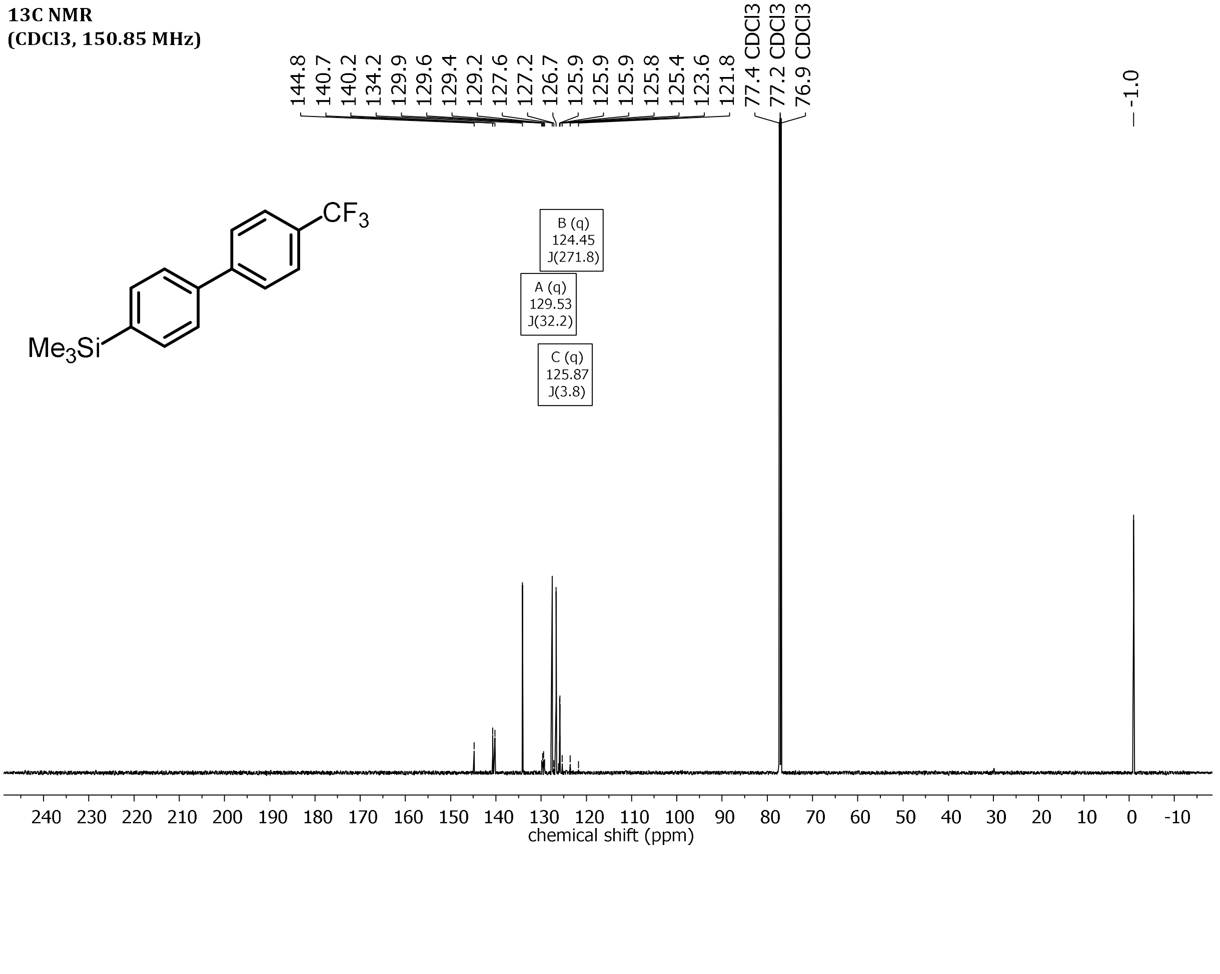

19F NMR
(CDCl3, 376.33)

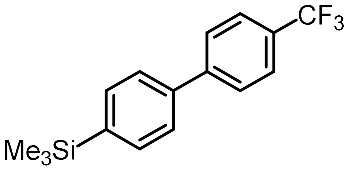

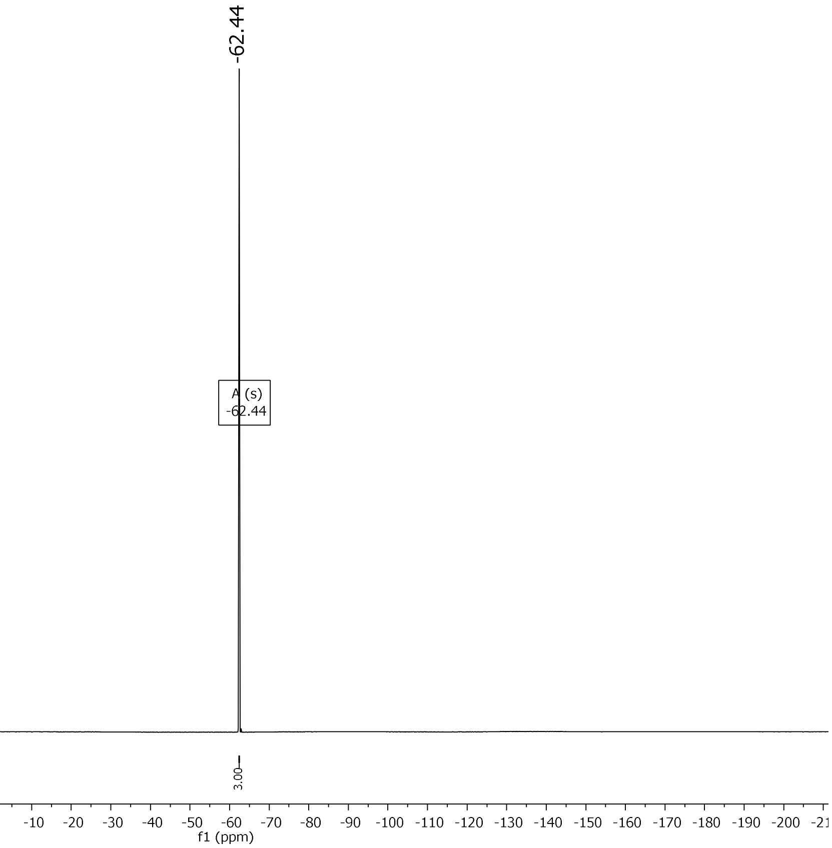

Ethyl 4'-(trimethylsilyl)-[1,1'-biphenyl]-4-carboxylate

1H NMR
(CDCl3, 599.86)

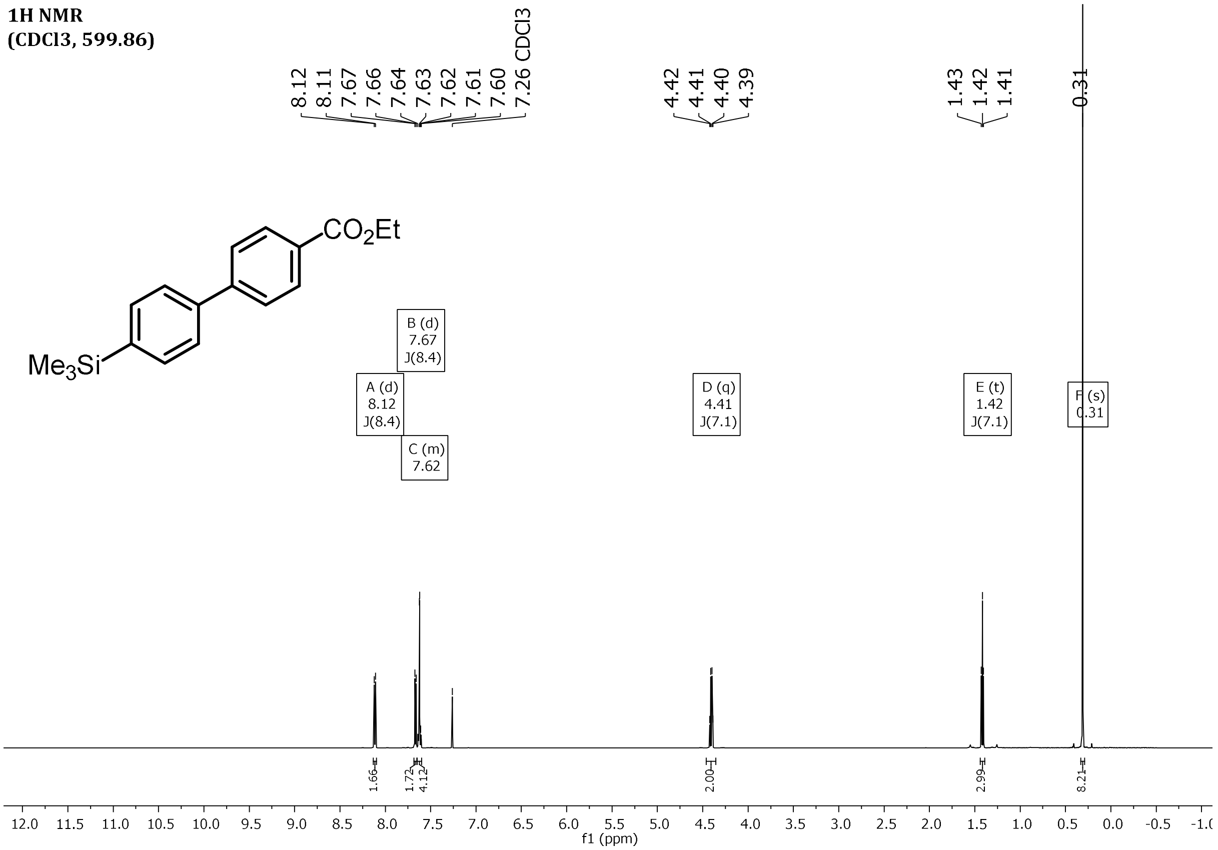

13C NMR
(CDCl3, 150.85 MHz)

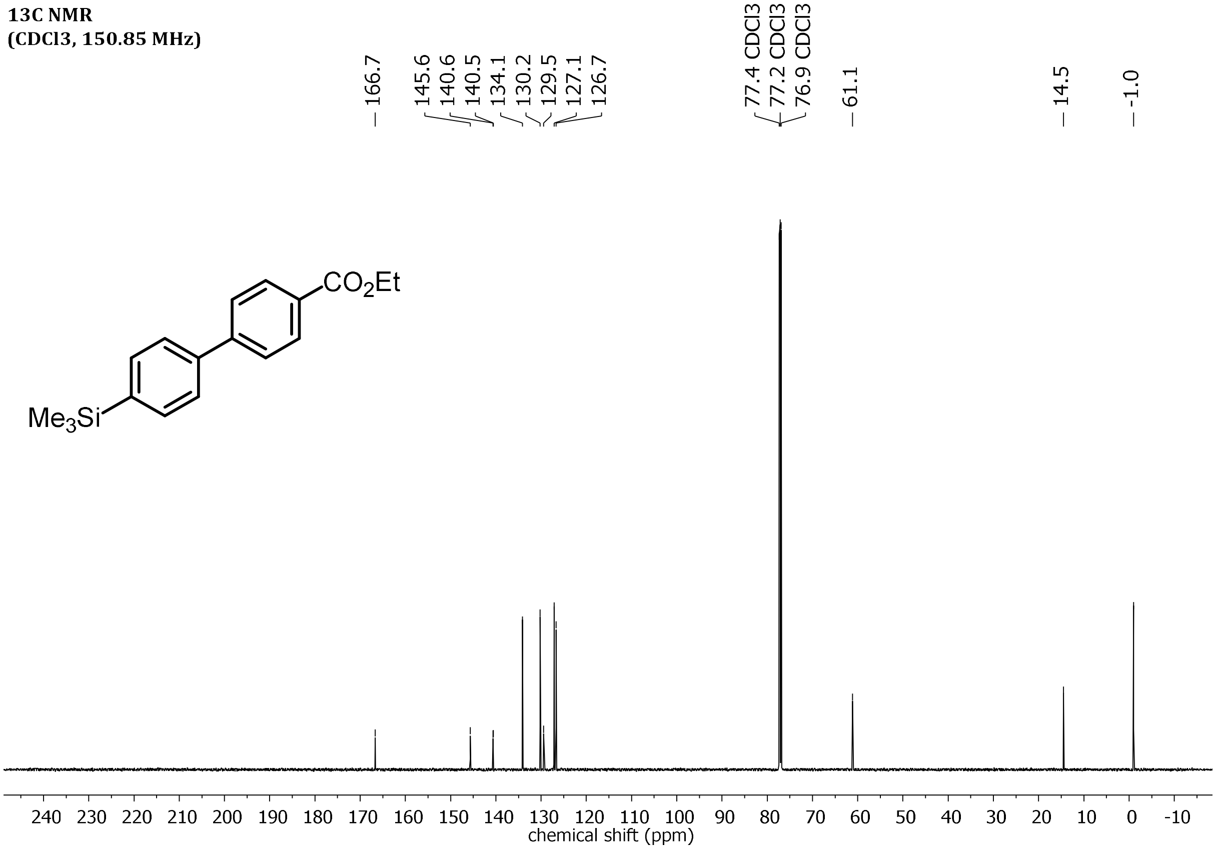

4'-(Trimethylsilyl)-[1,1'-biphenyl]-2-carbonitrile

1H NMR
(CDCl3, 399.97 MHz)

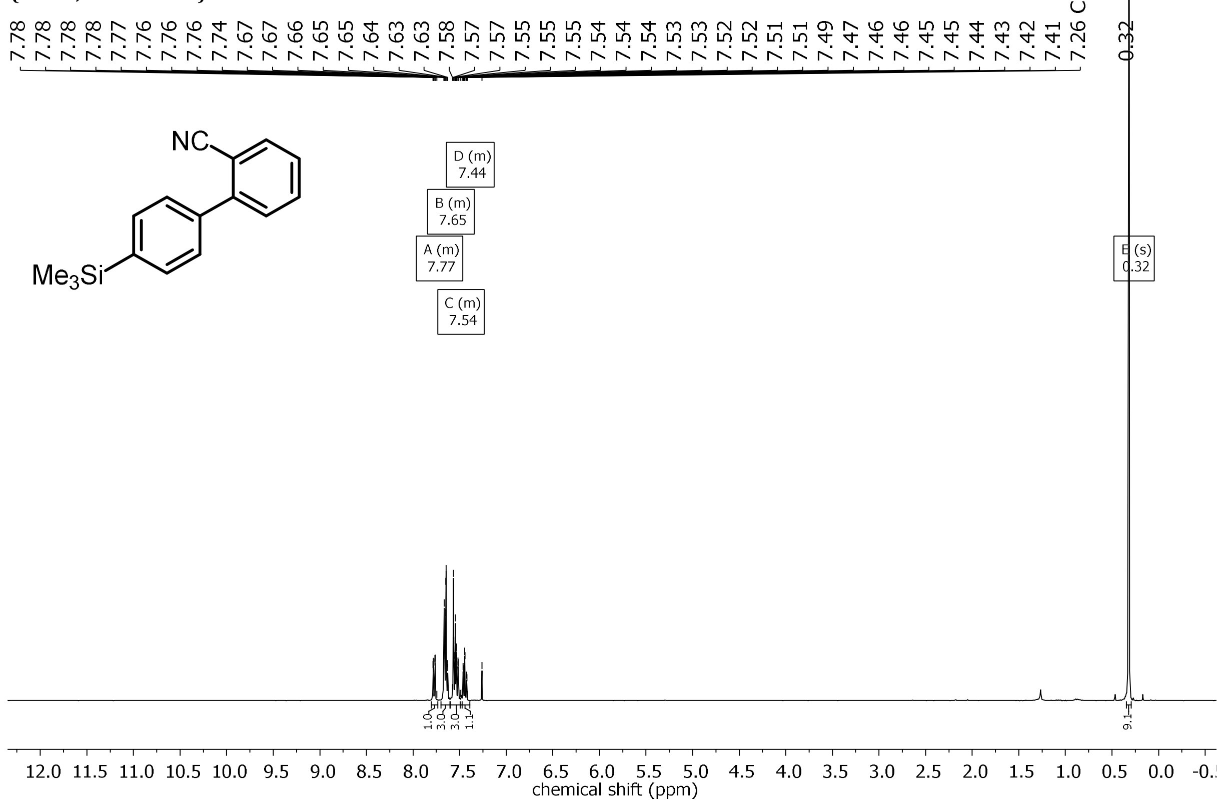

13C NMR
(CDCl3, 100.58 MHz)

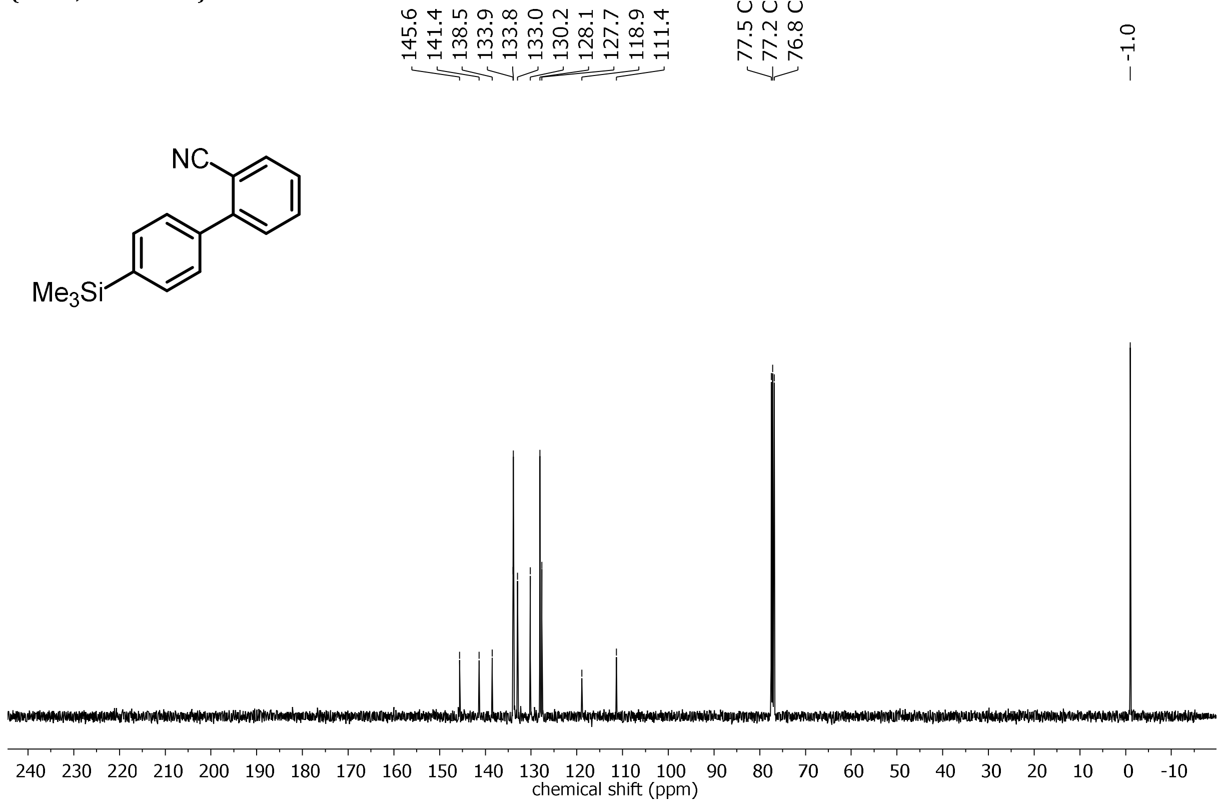

1-(4'-(Trimethylsilyl)-[1,1'-biphenyl]-4-yl)ethan-1-one

1H NMR
(CDCl3, 399.97)

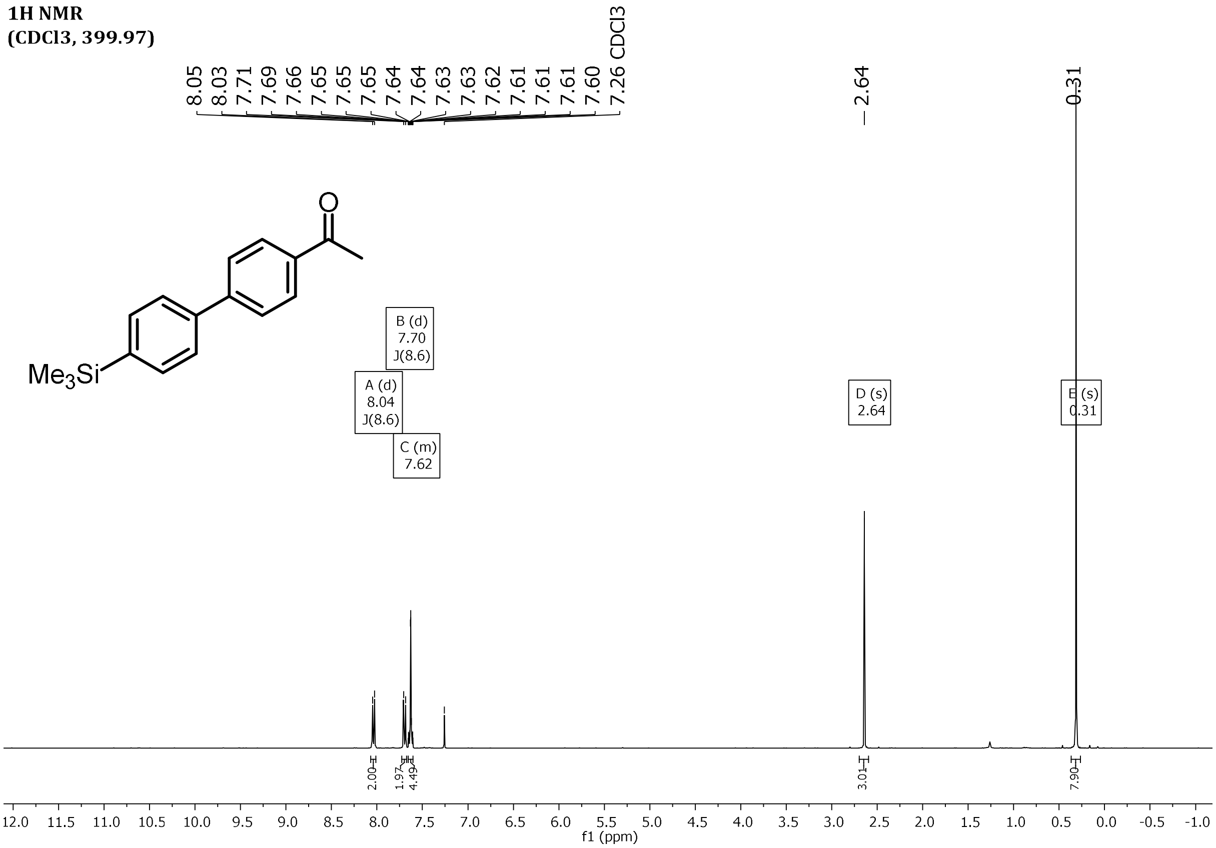

13C NMR
(CDCl3, 100.58 MHz)

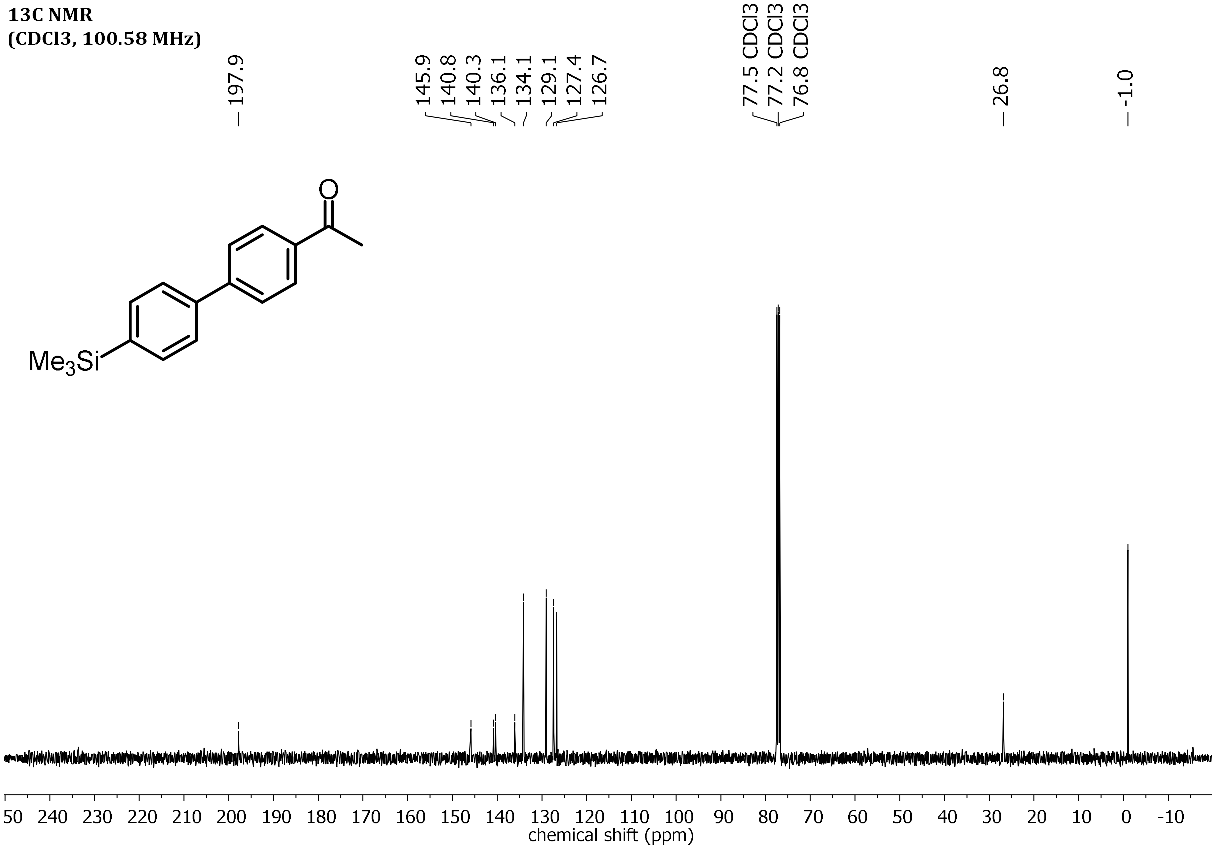

5-(2-Nitrophenyl)benzo[*d*][1,3]dioxole

1H NMR
(CDCl3, 599.86 MHz)

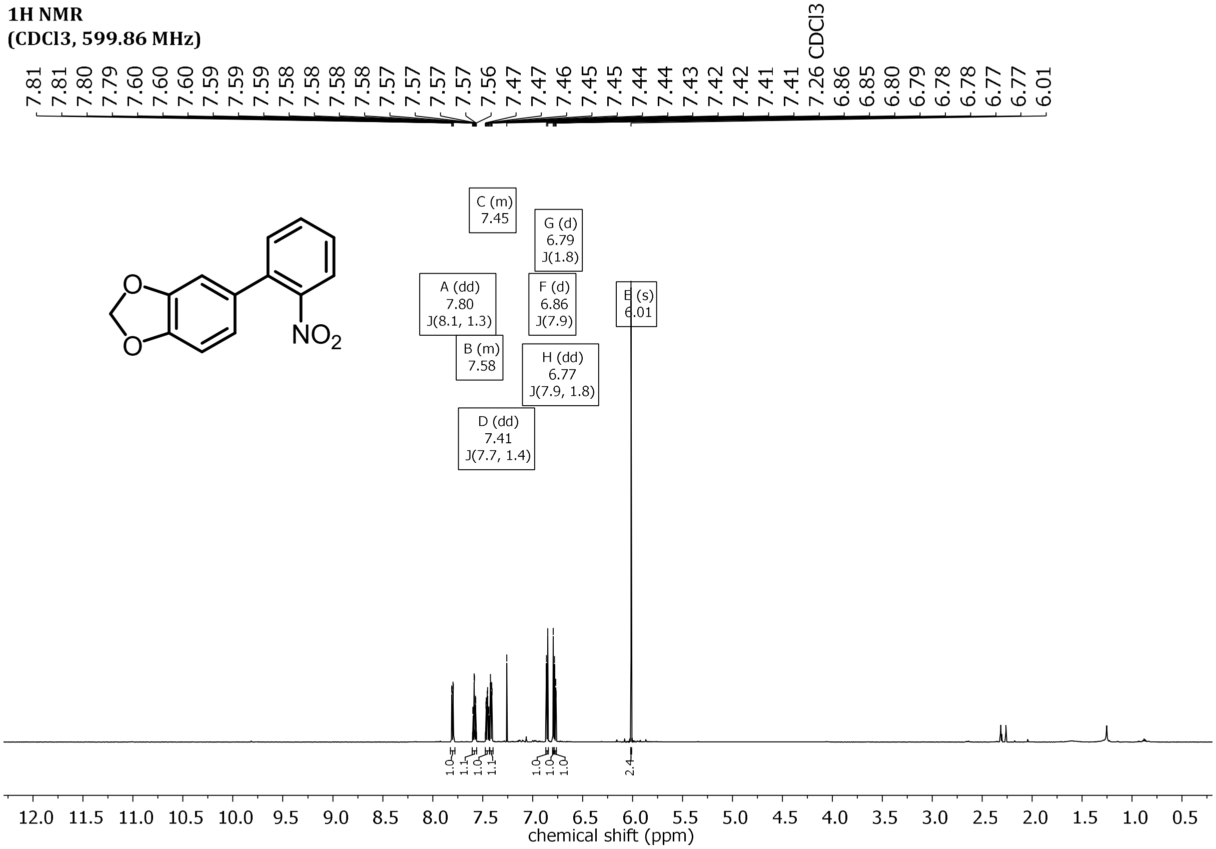

13C NMR
(CDCl3, 150.85 MHz)

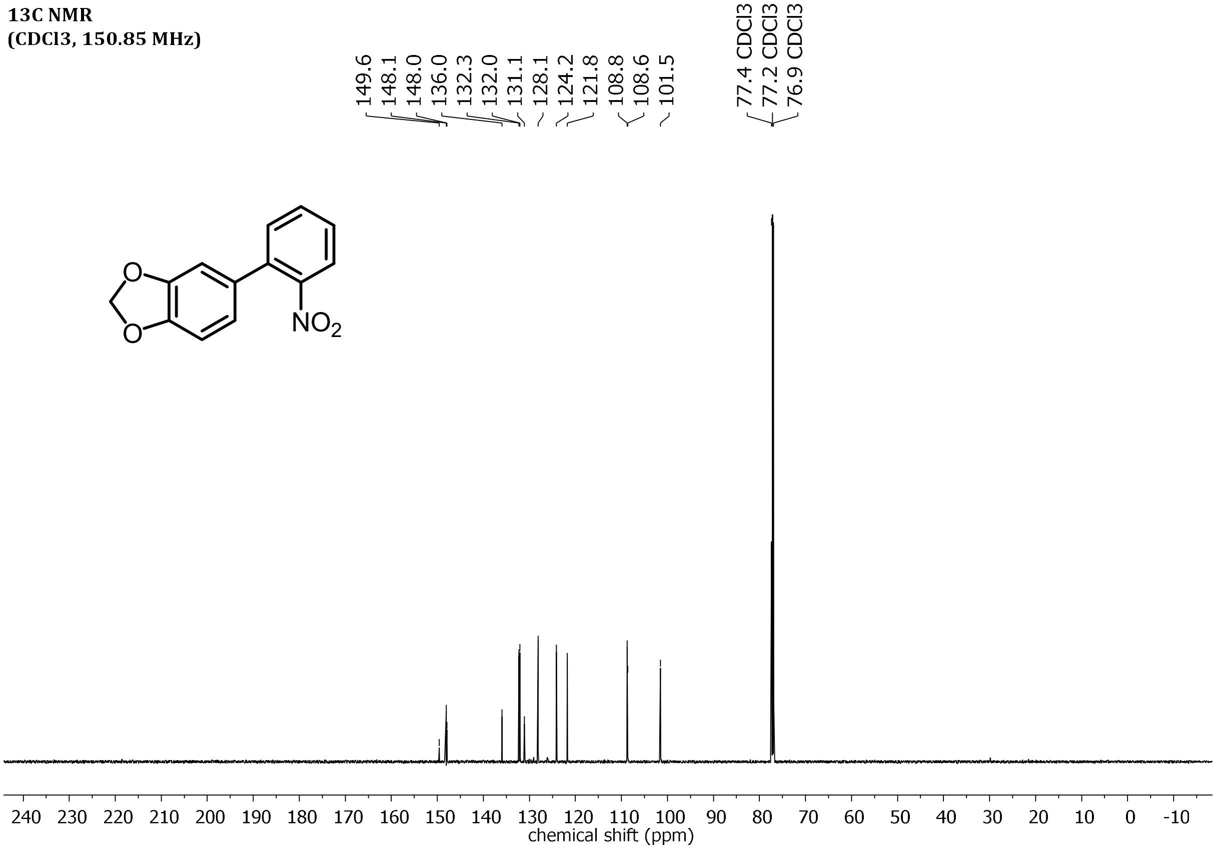

1-(2'-Isopropyl-[1,1'-biphenyl]-4-yl)ethan-1-one

1H NMR
(CDCl3, 599.86 MHz)

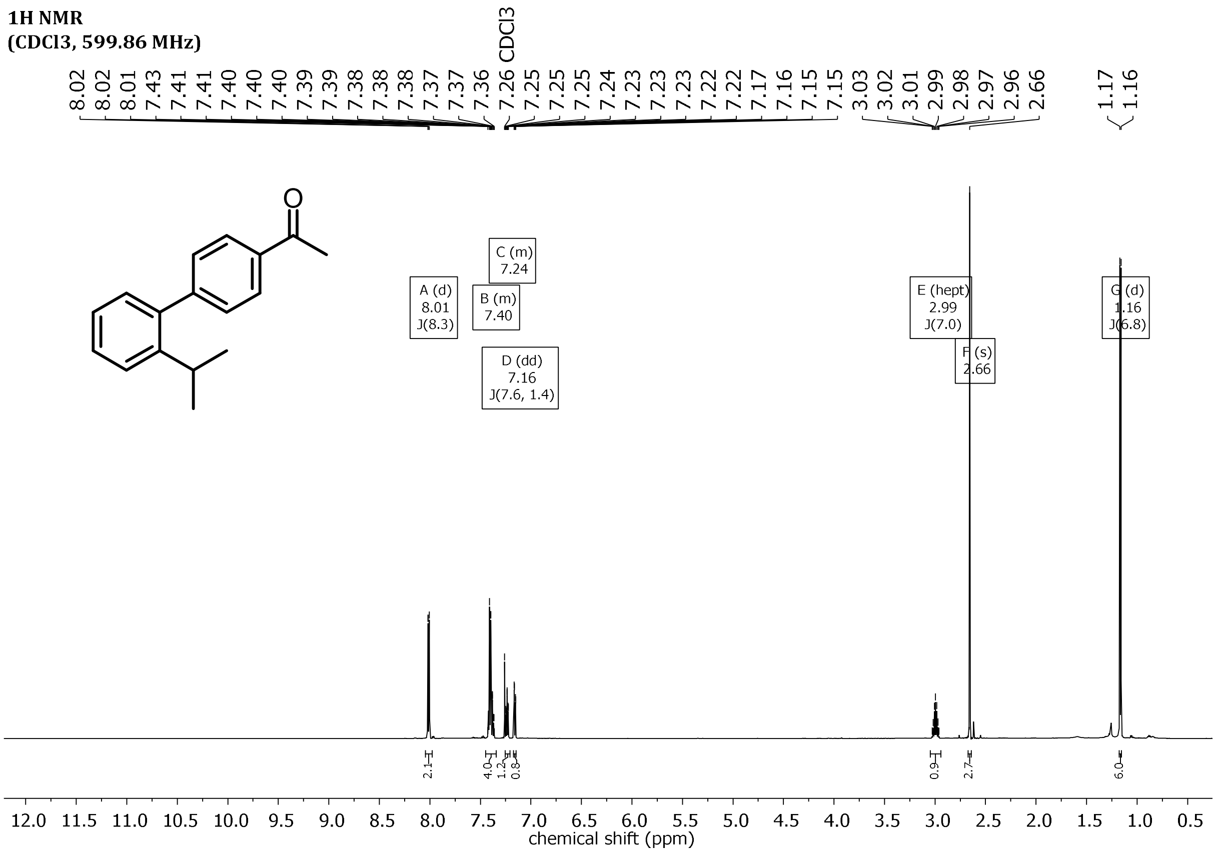

13C NMR
(CDCl3, 150.85 MHz)

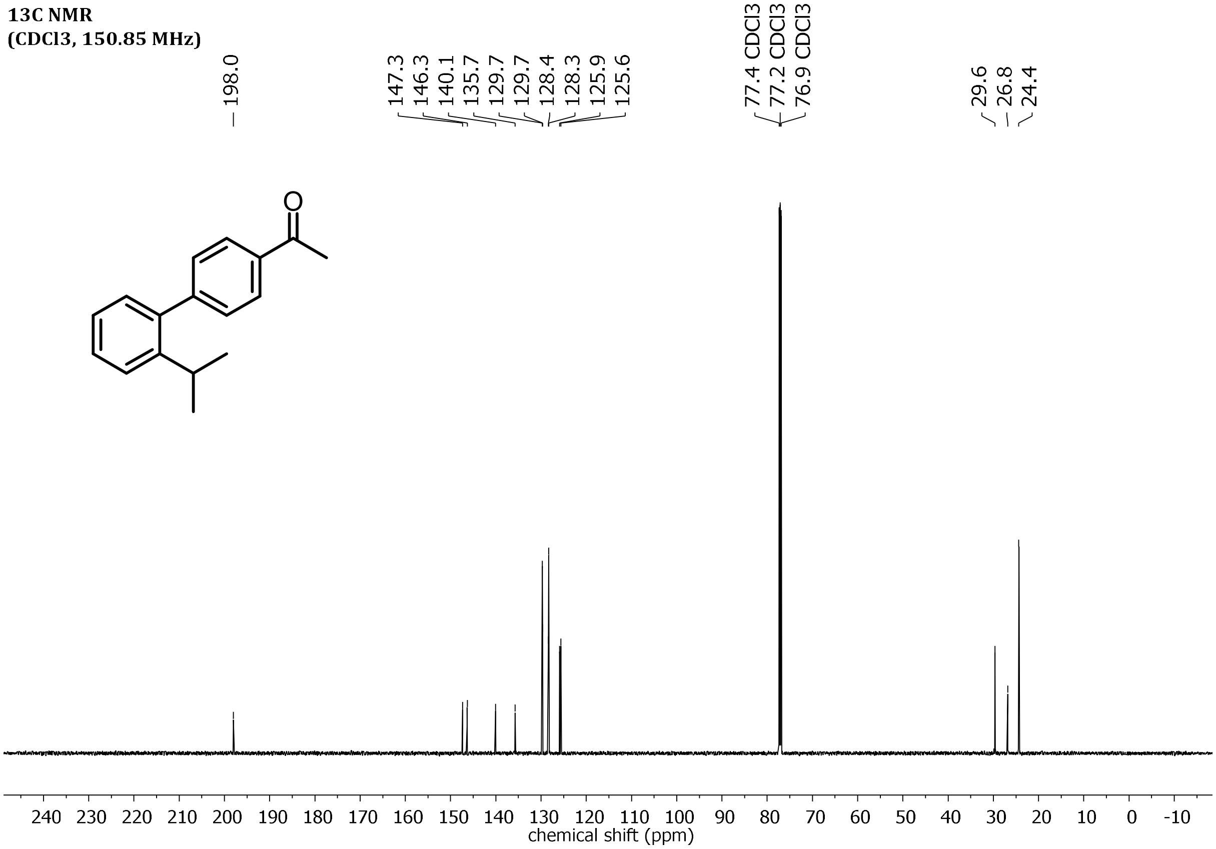

3'-Bromo-5'-chloro-2-nitro-1,1'-biphenyl

1H NMR
(CDCl3, 599.86 MHz)

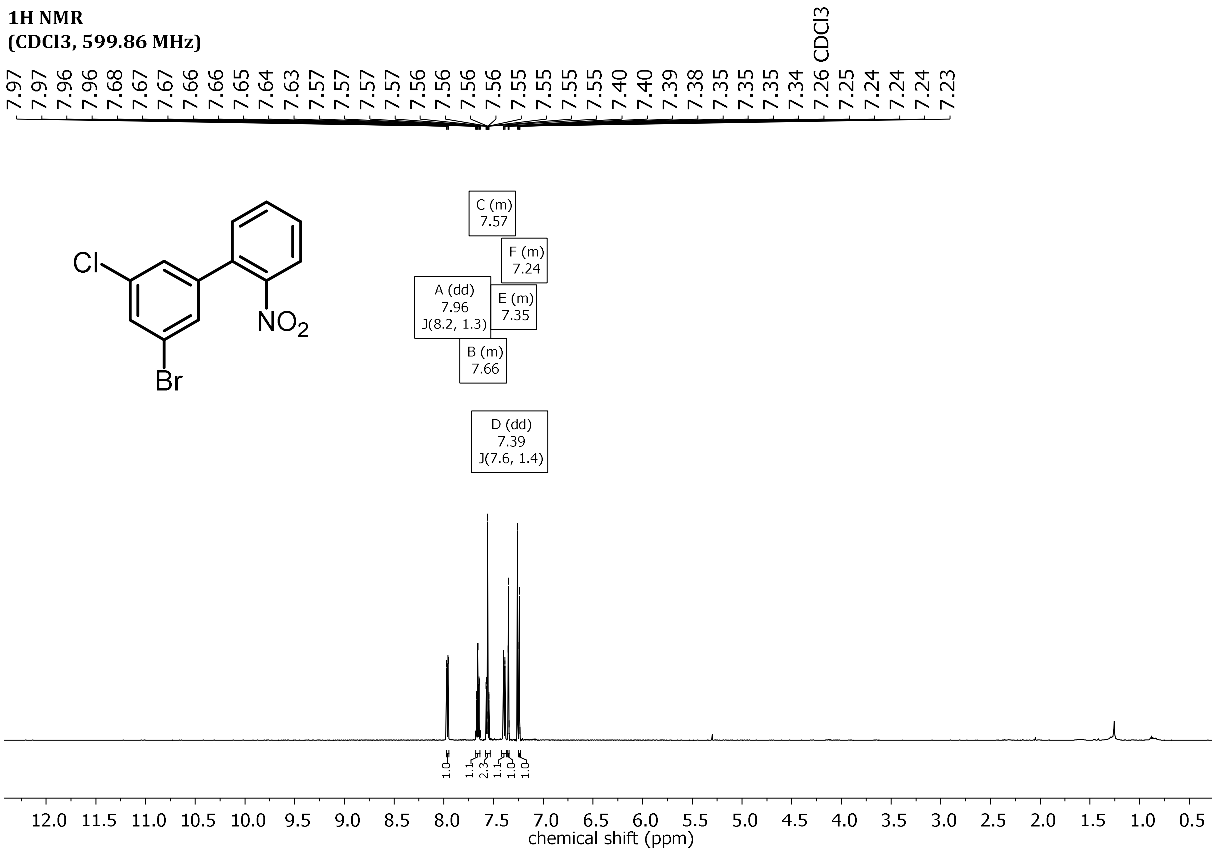

13C NMR
(CDCl3, 150.85 MHz)

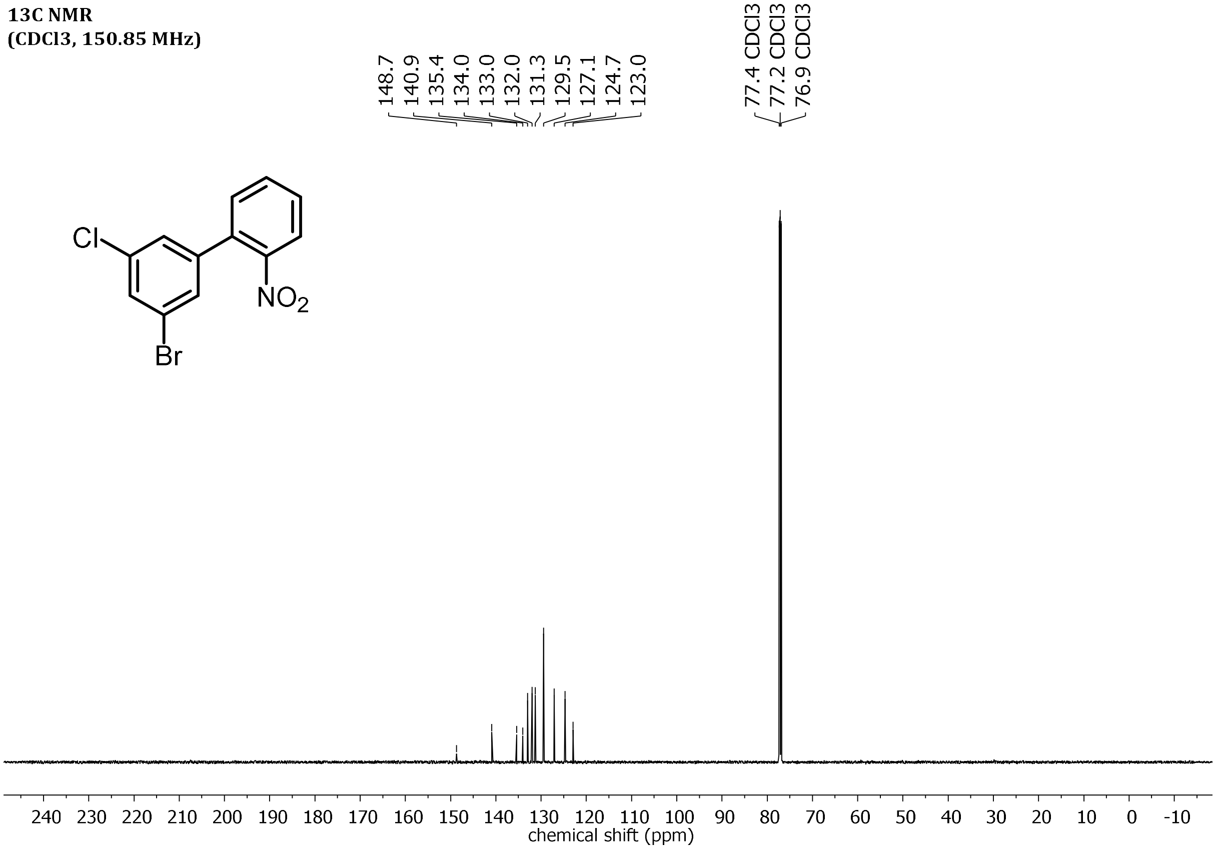

2'-Bromo-4'-chloro-4-methoxy-[1,1'-biphenyl]-3-carbonitrile

1H NMR
(CDCl3, 599.86)

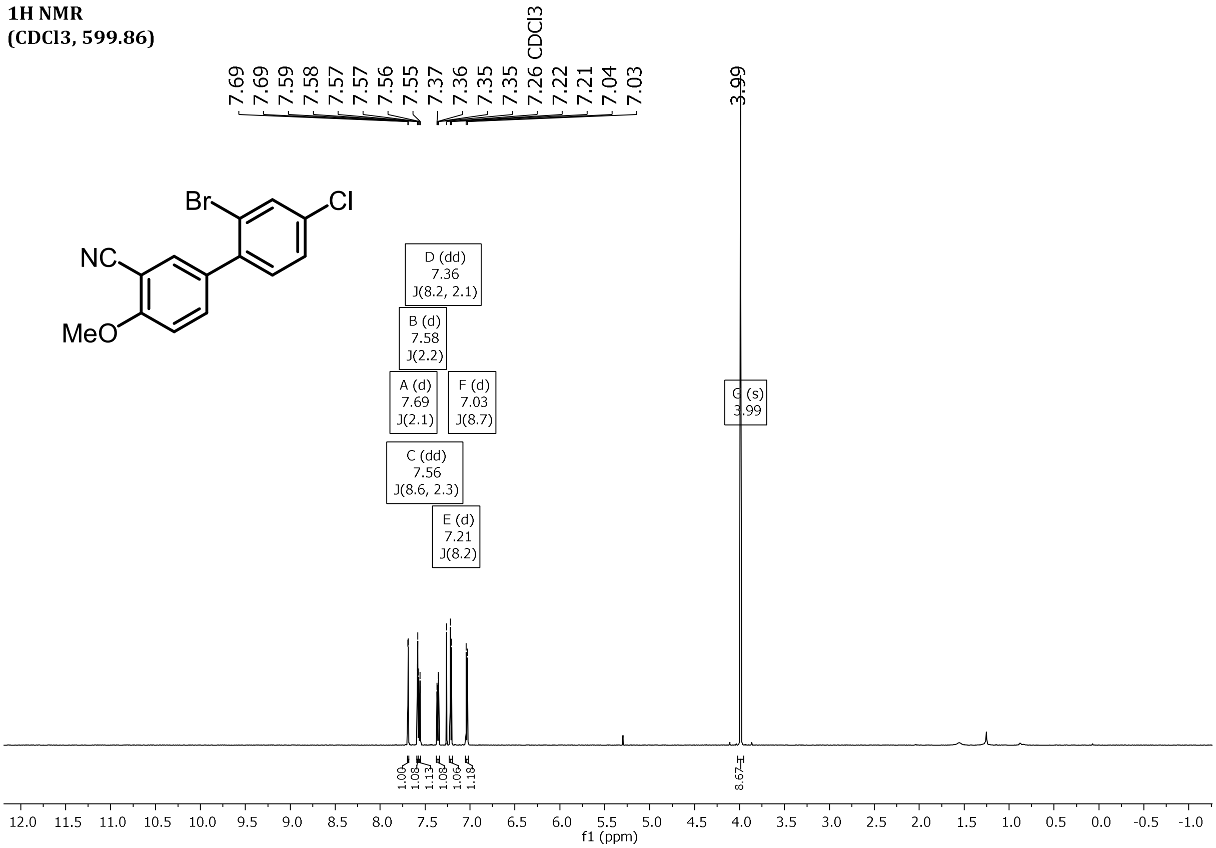

13C NMR
(CDCl3, 150.85 MHz)

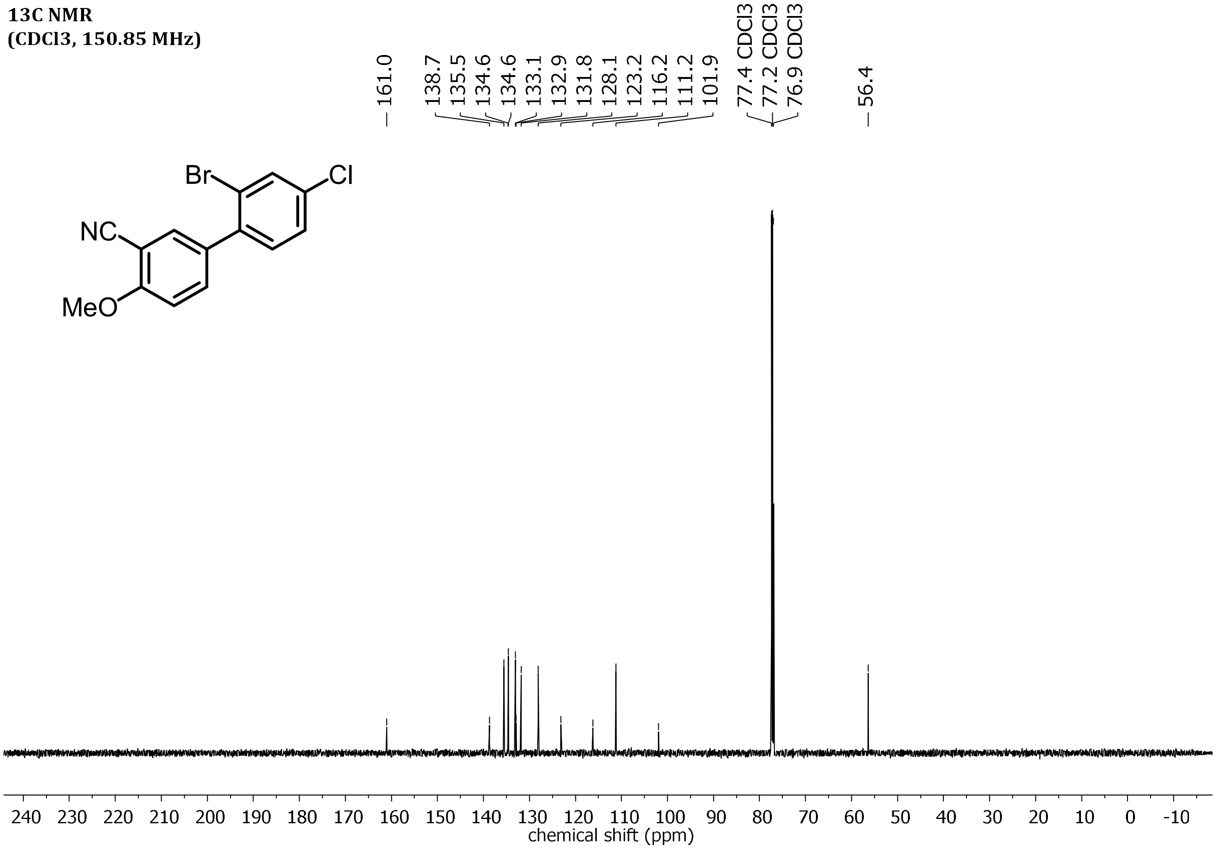

1-(4'-Bromo-3'-methyl-[1,1'-biphenyl]-4-yl)ethan-1-one

1H NMR (CDCl3, 599.86)

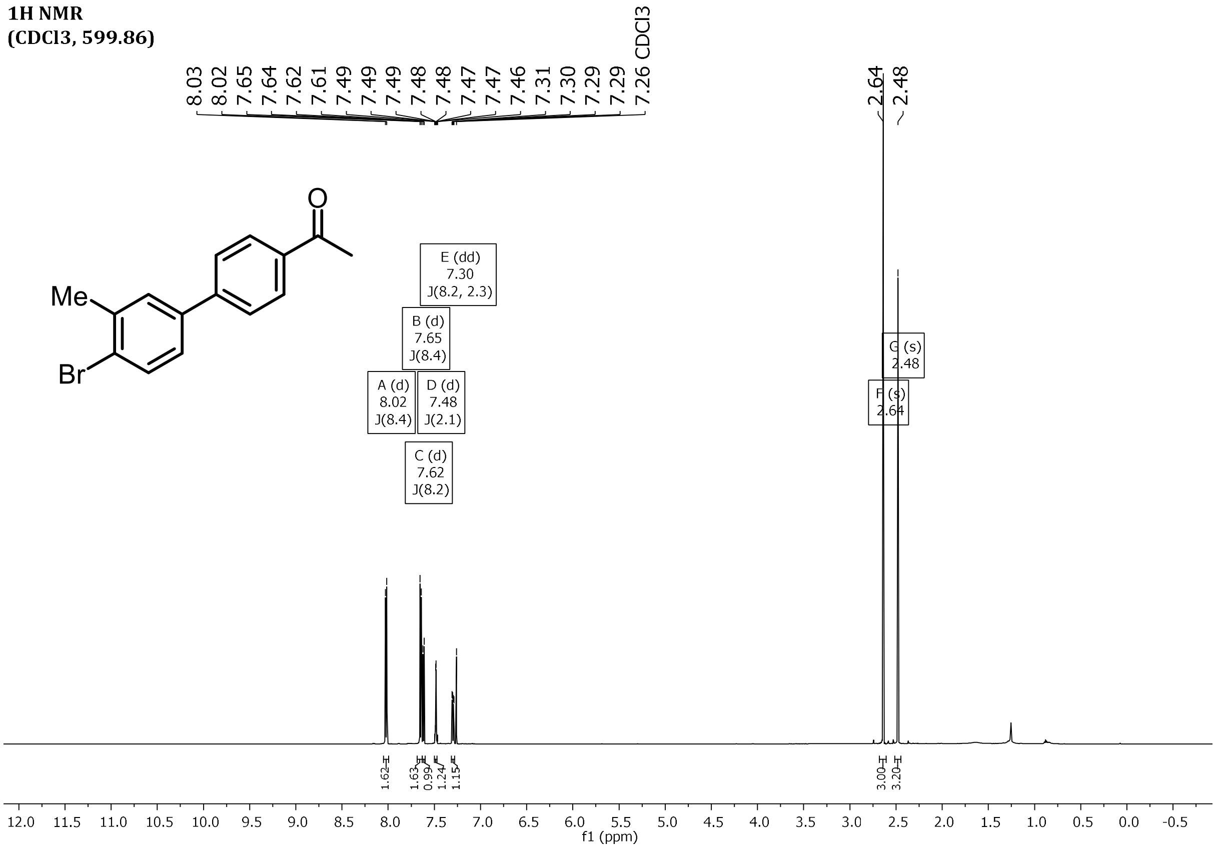

13C NMR (CDCl3, 150.85 MHz)

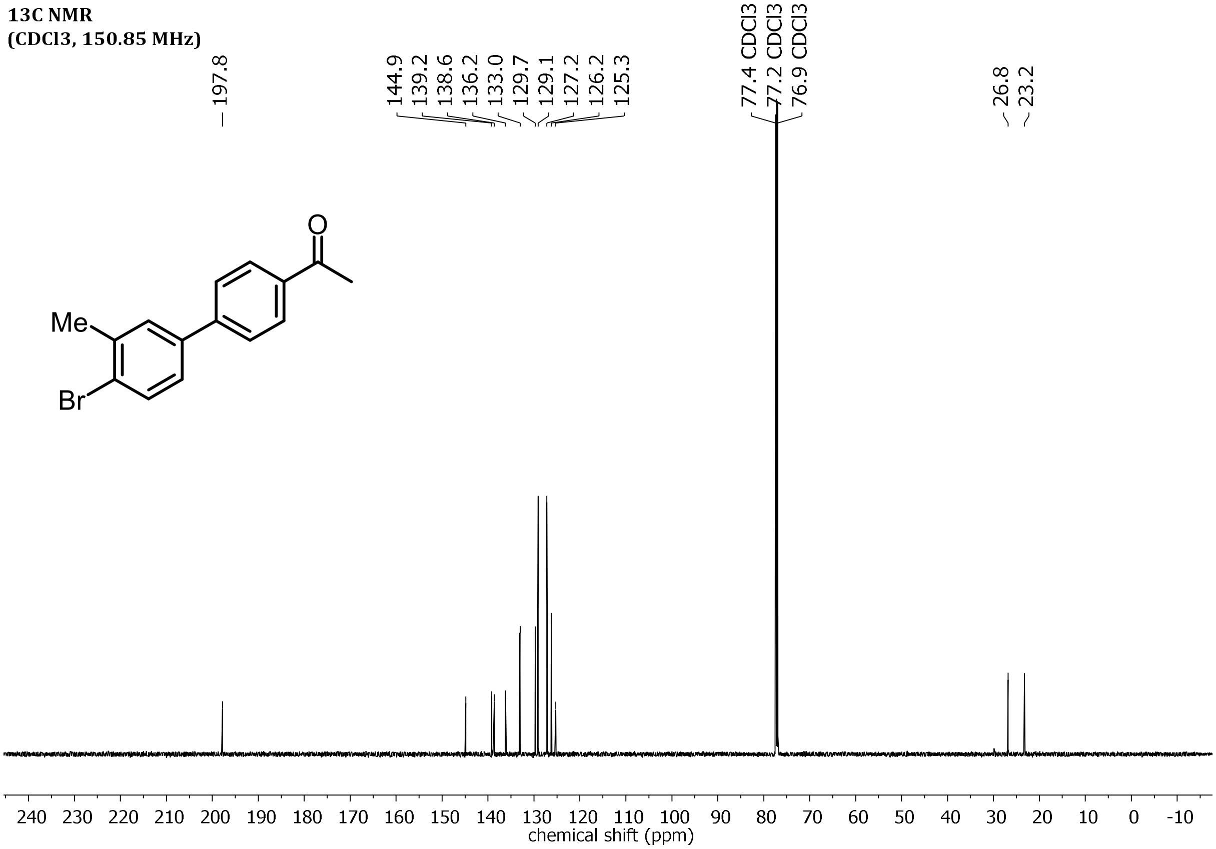

4-Methoxy-3',5'-bis(trifluoromethyl)-[1,1'-biphenyl]-3-carbonitrile

1H NMR
(CDCl3, 599.86)

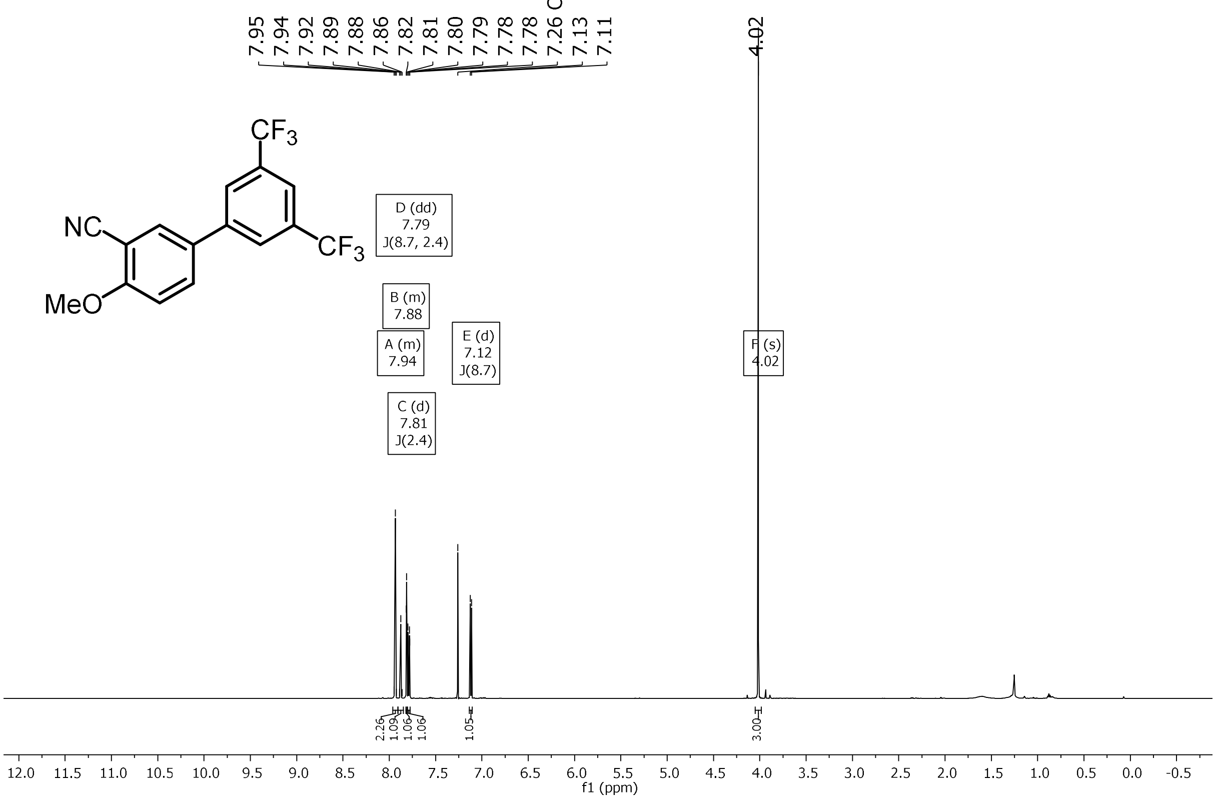

13C NMR
(CDCl3, 150.85 MHz)

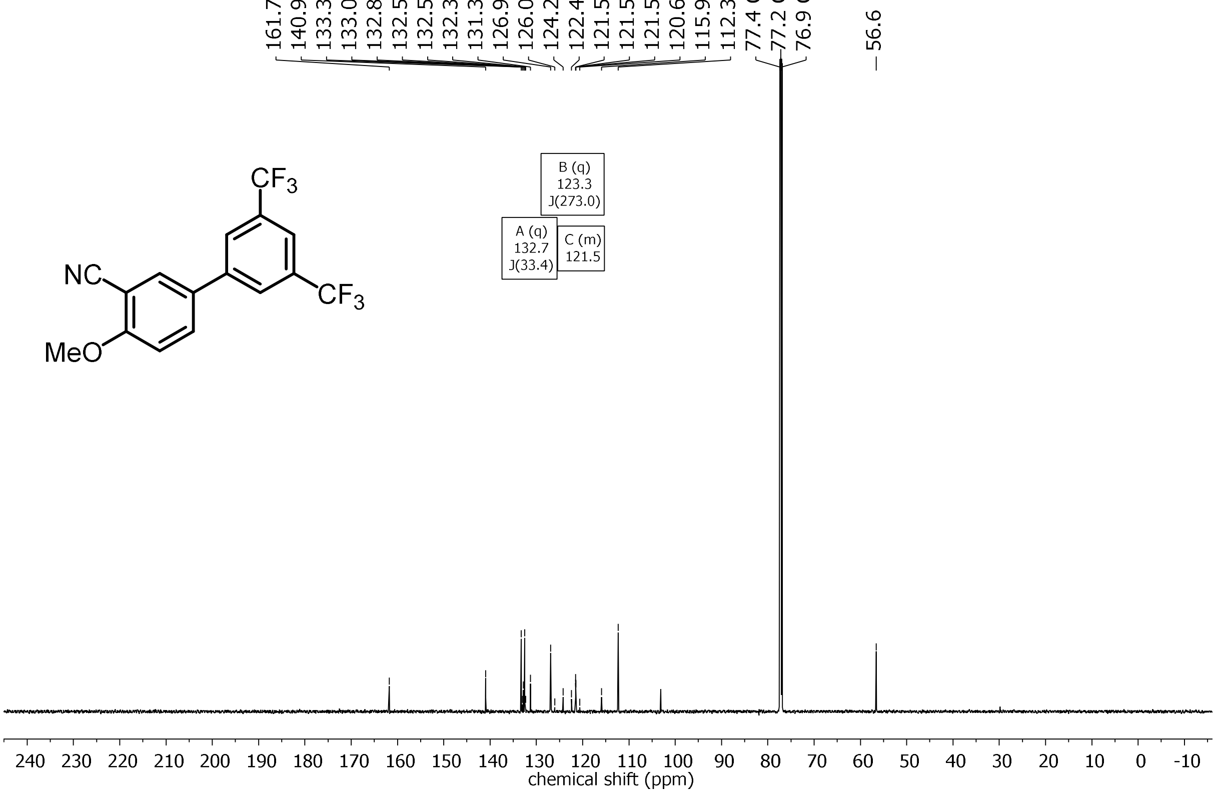

19F NMR
(CDCl3, 376.33 MHz)

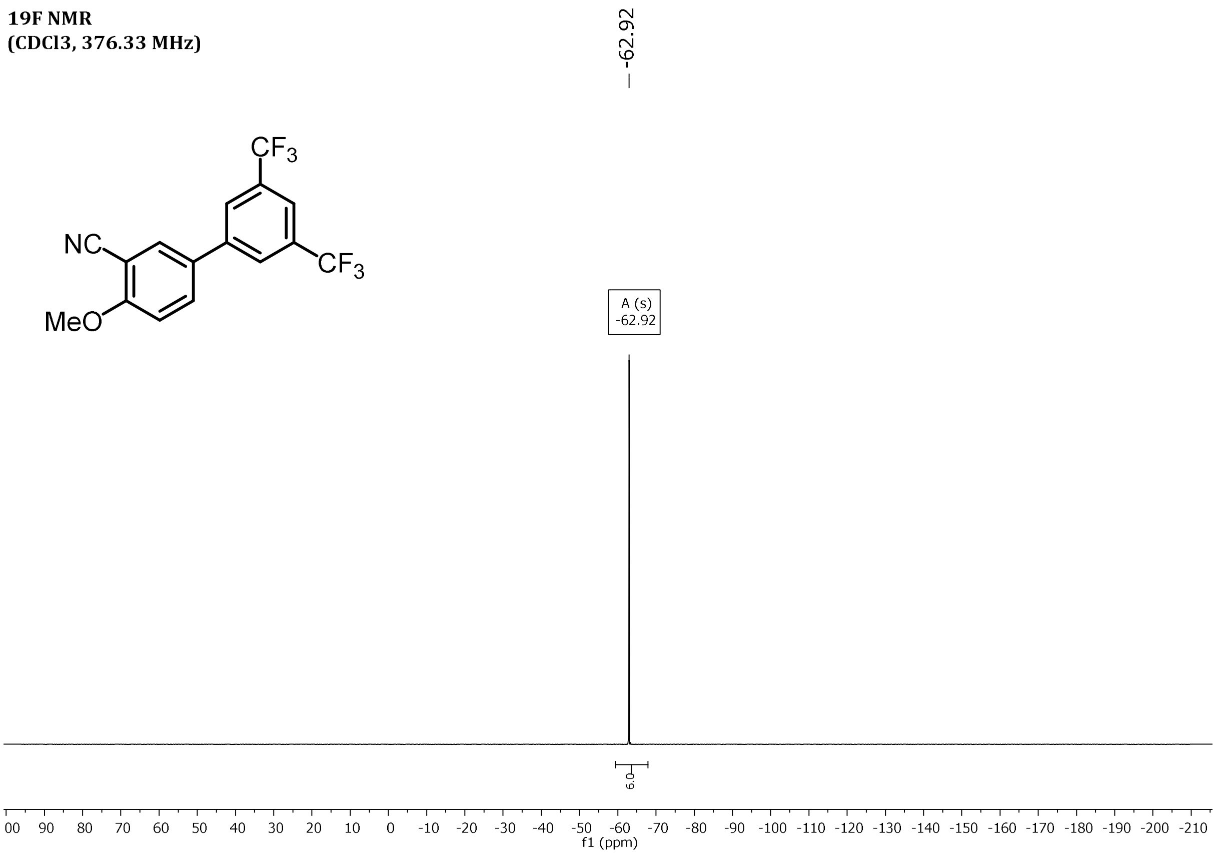

2,4,6-Trifluoro-4'-nitro-1,1'-biphenyl

1H NMR
599.86 MHz, CDCl3

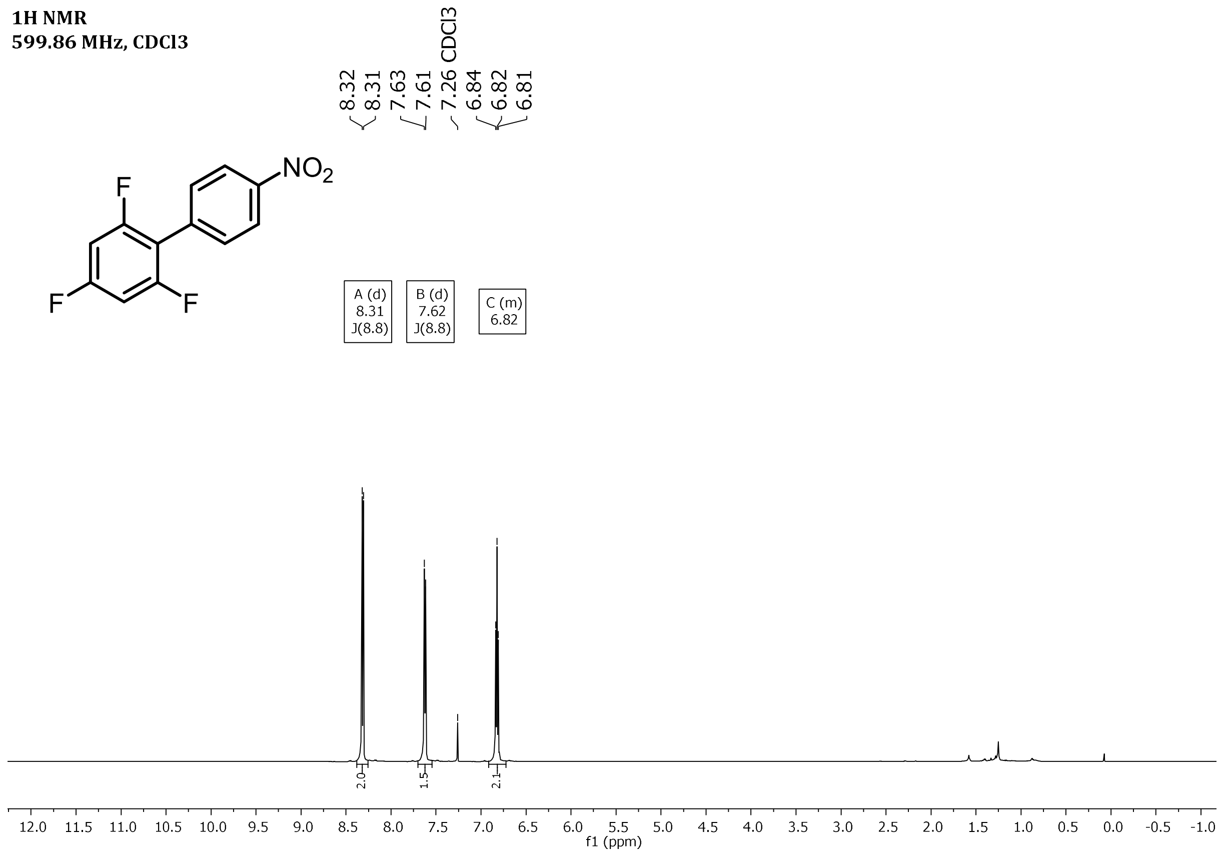

13C NMR
(CDCl3, 150.85 MHz)

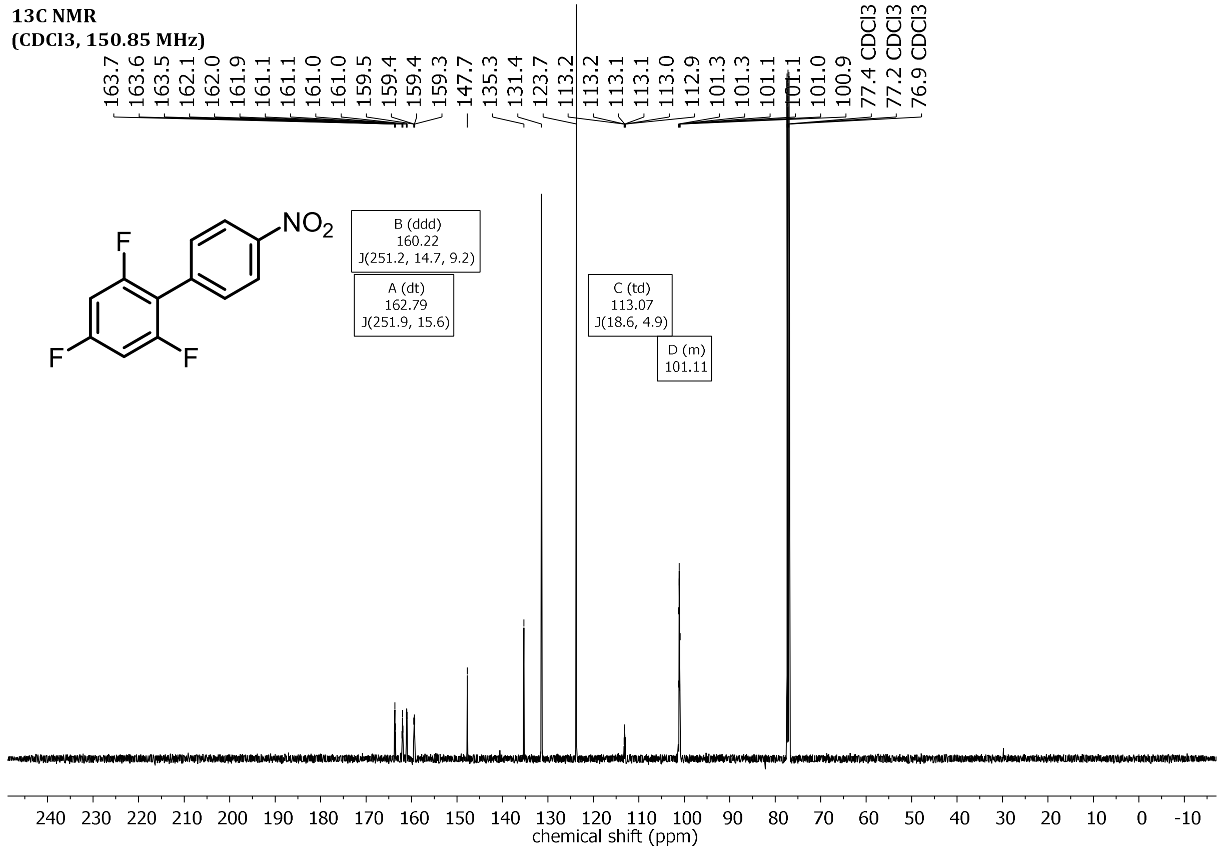

19F NMR
564.40 MHz, CDCl3

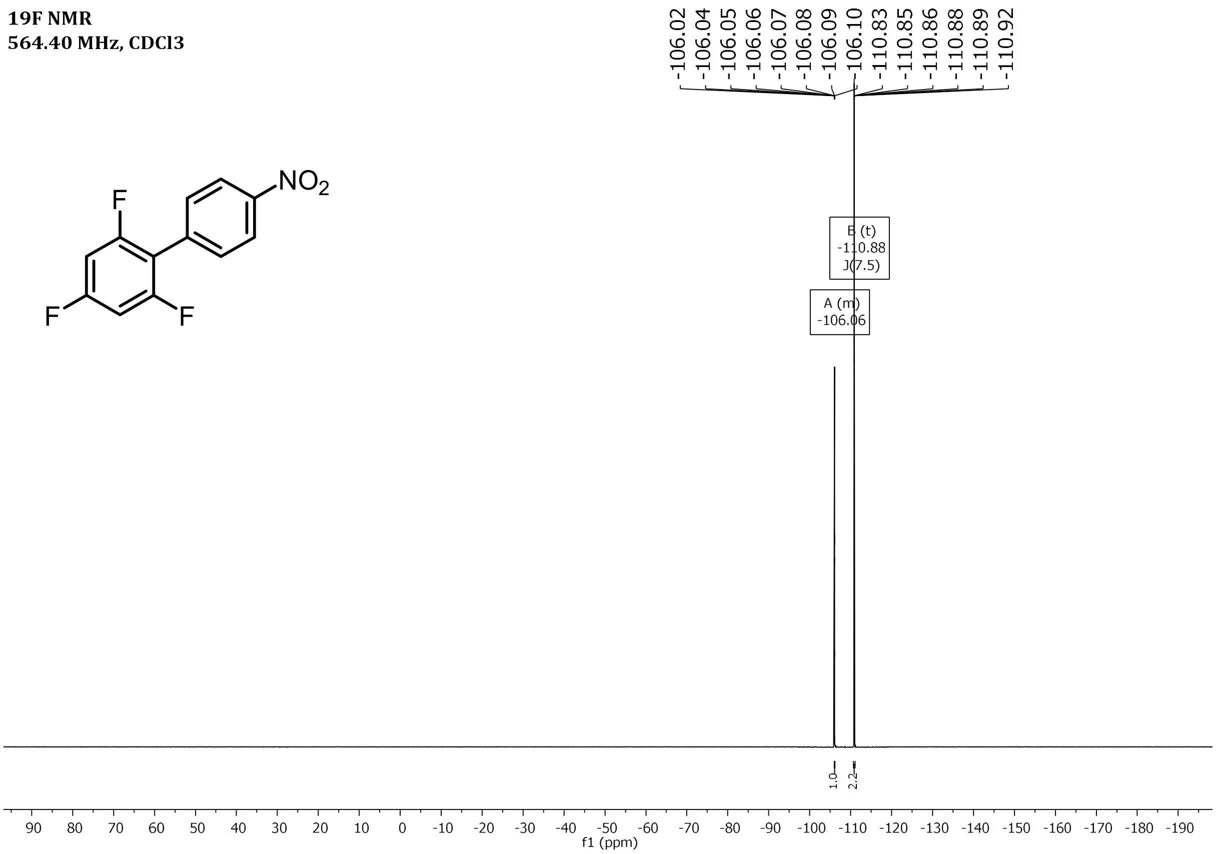

2-(3'-Chloro-[1,1'-biphenyl]-4-yl)-4,4,5,5-tetramethyl-1,3,2-dioxaborolane

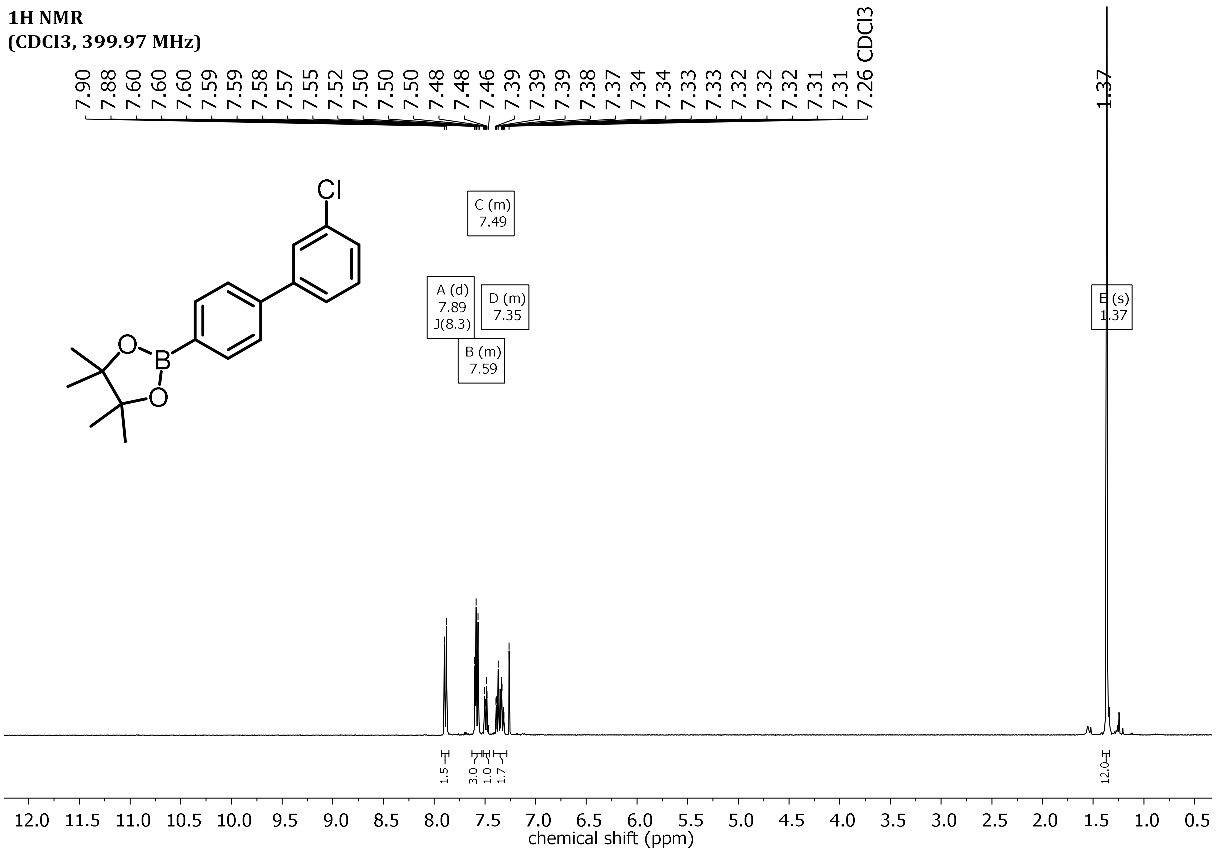

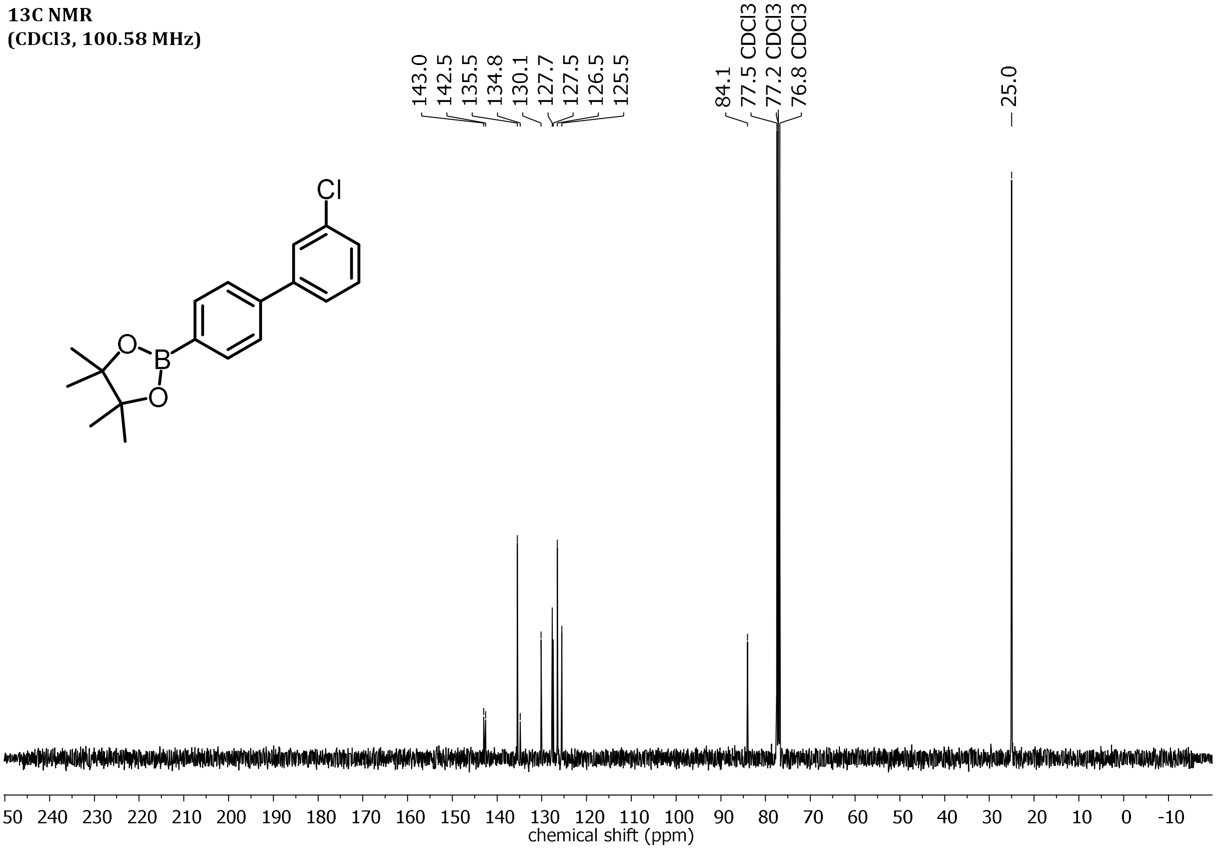

2-(3'-Fluoro-[1,1'-biphenyl]-4-yl)-4,4,5,5-tetramethyl-1,3,2-dioxaborolane

1H NMR
(CDCl3, 599.86 MHz)

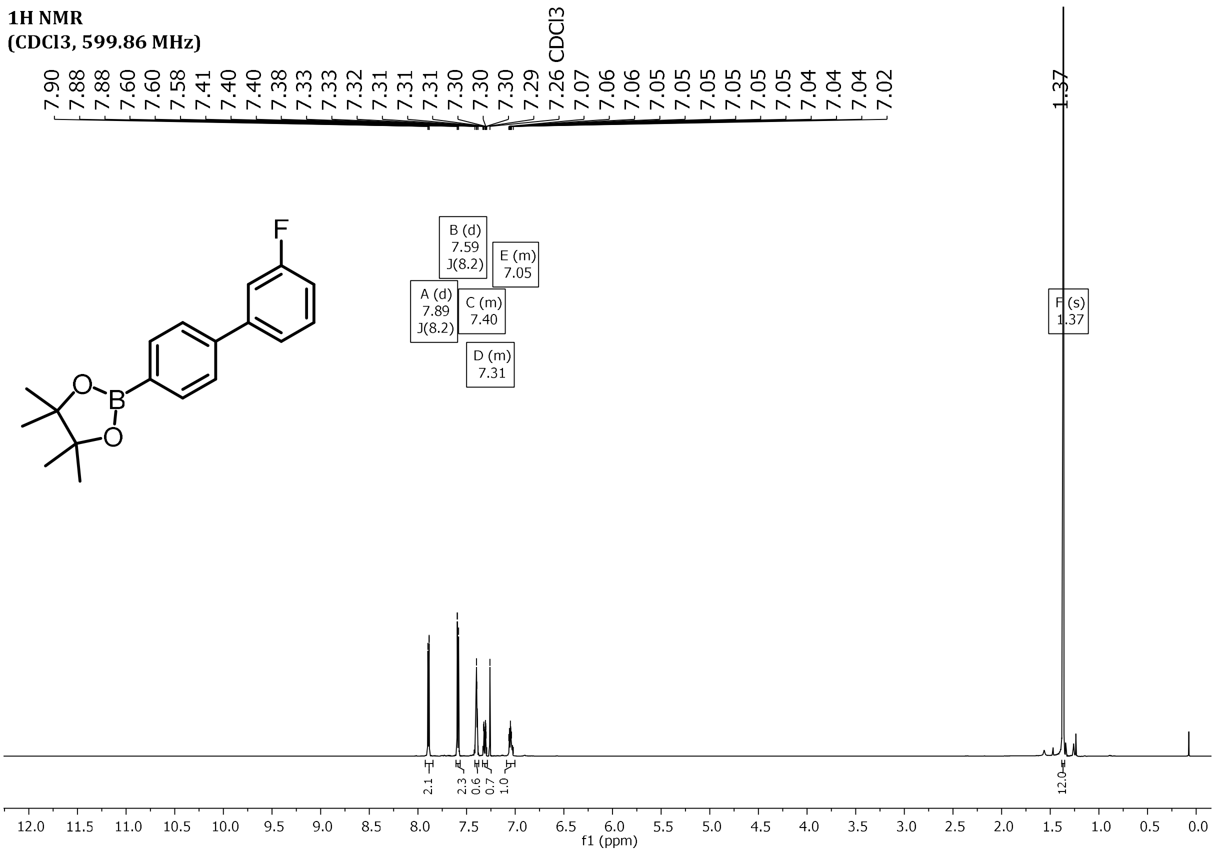

13C NMR
(CDCl3, 150.85 MHz)

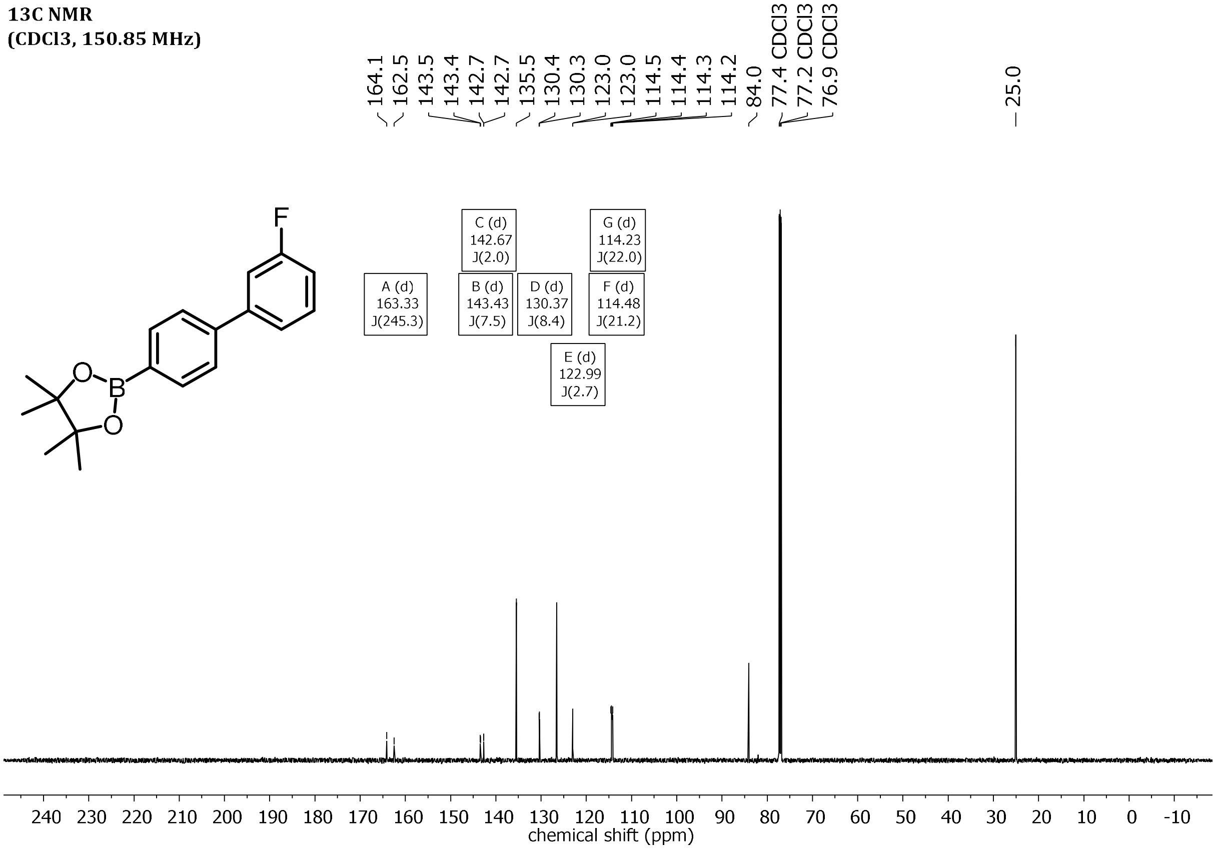

19F NMR
564.40 MHz, CDCl3

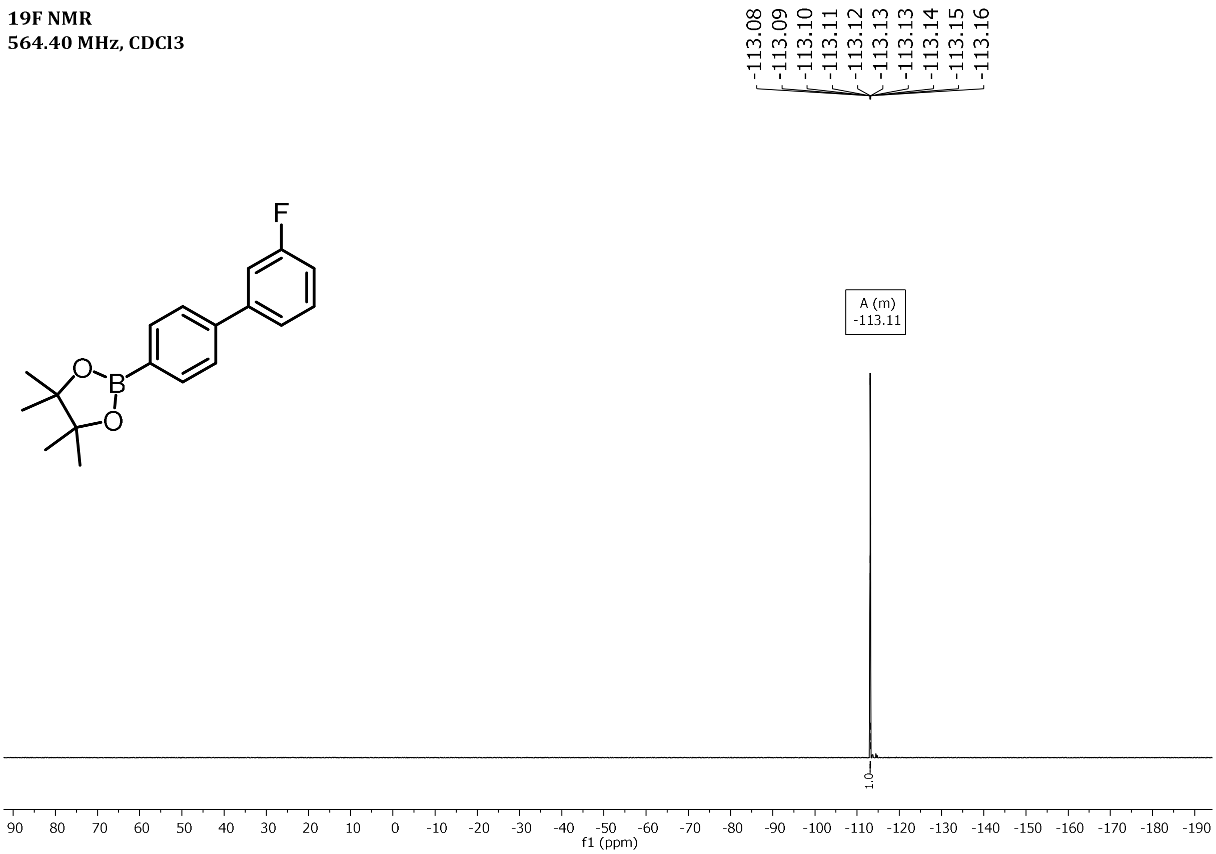

Ethyl 4'-(4,4,5,5-tetramethyl-1,3,2-dioxaborolan-2-yl)-[1,1'-biphenyl]-3-carboxylate

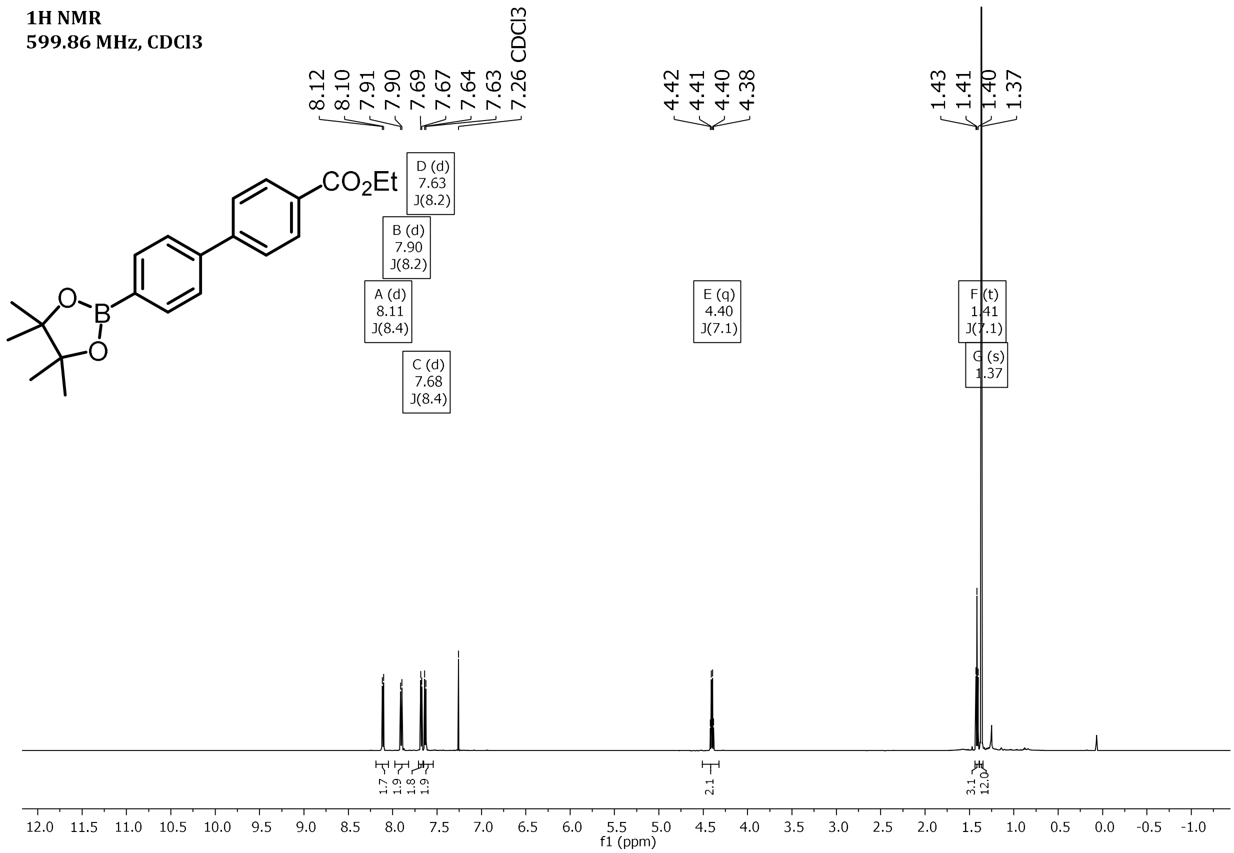

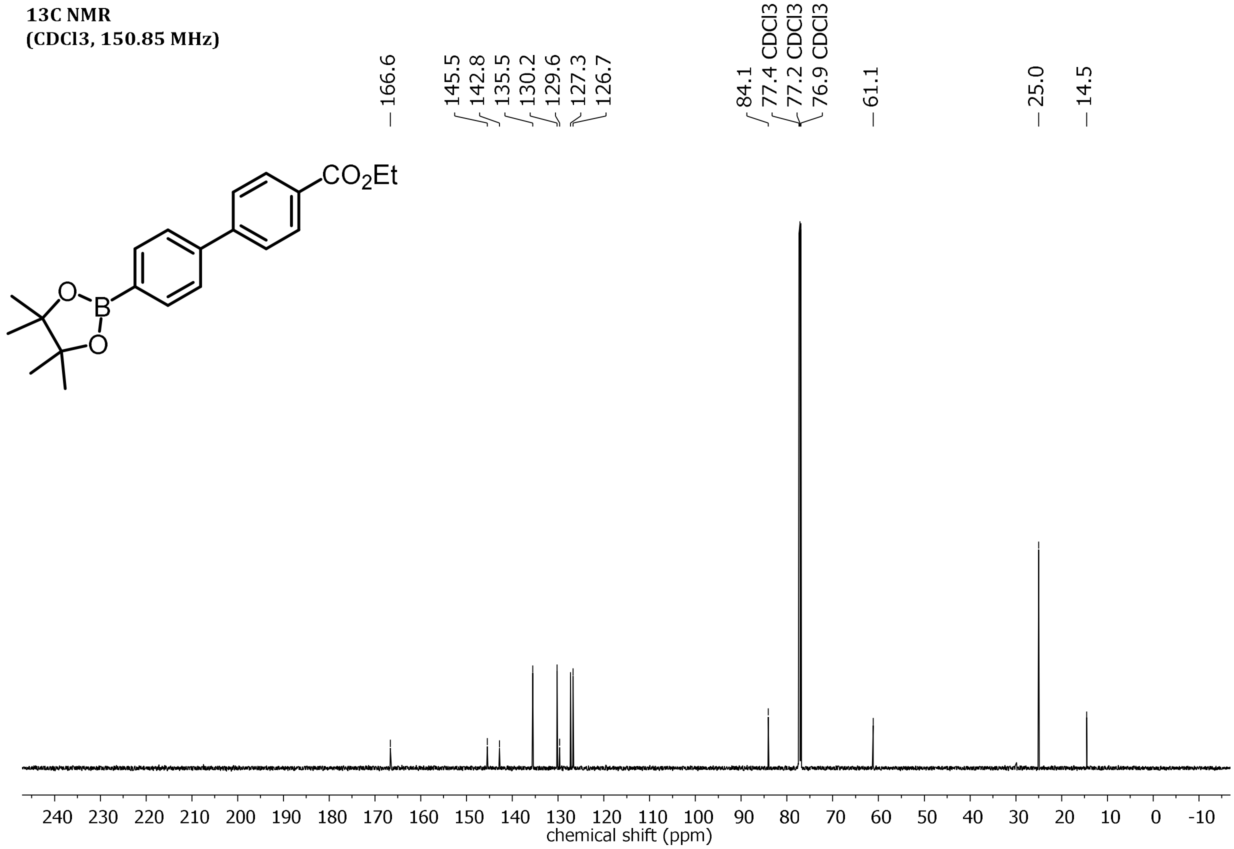

4,4,5,5-Tetramethyl-2-(4'-nitro-[1,1'-biphenyl]-4-yl)-1,3,2-dioxaborolane

1H NMR
599.86 MHz, CDCl3

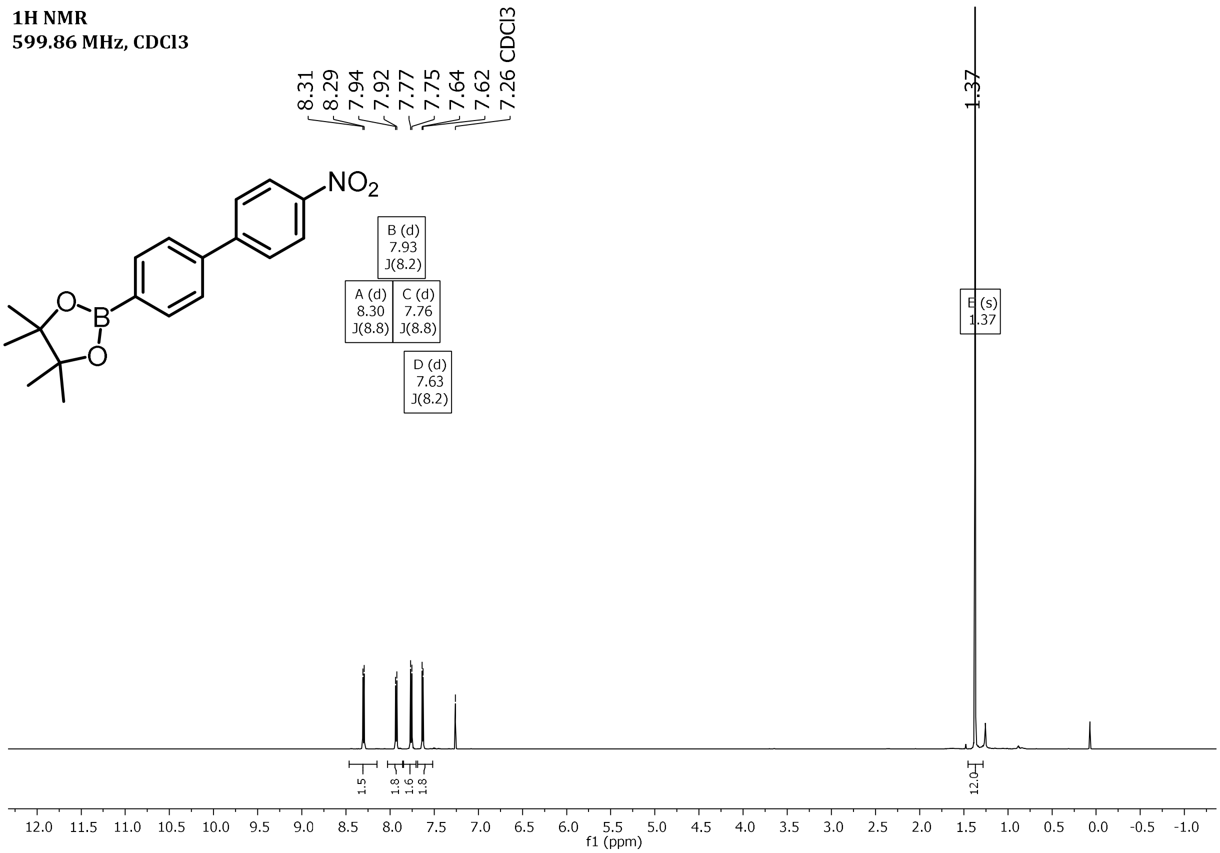

13C NMR
(CDCl3, 150.85 MHz)

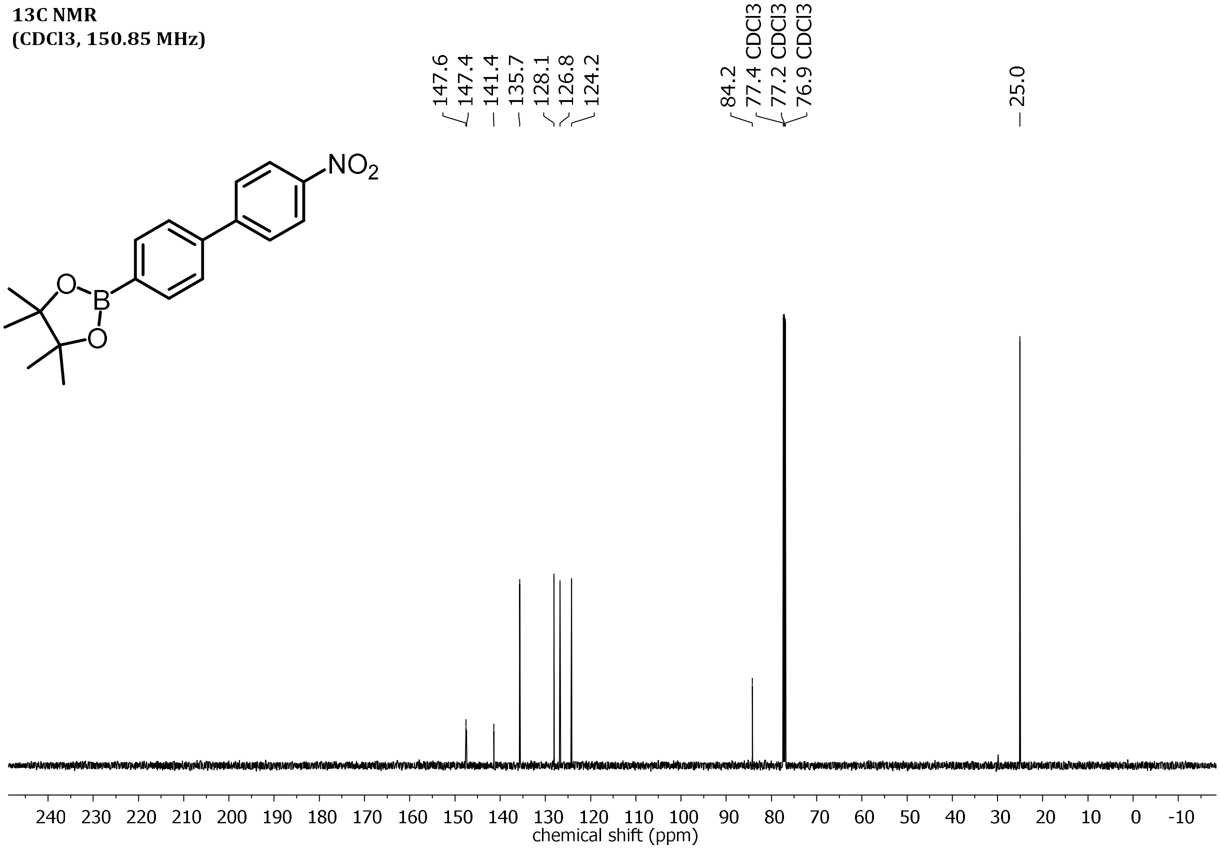

2-(Allyloxy)-4'-nitro-1,1'-biphenyl

1H NMR
599.86 MHz, CDCl3

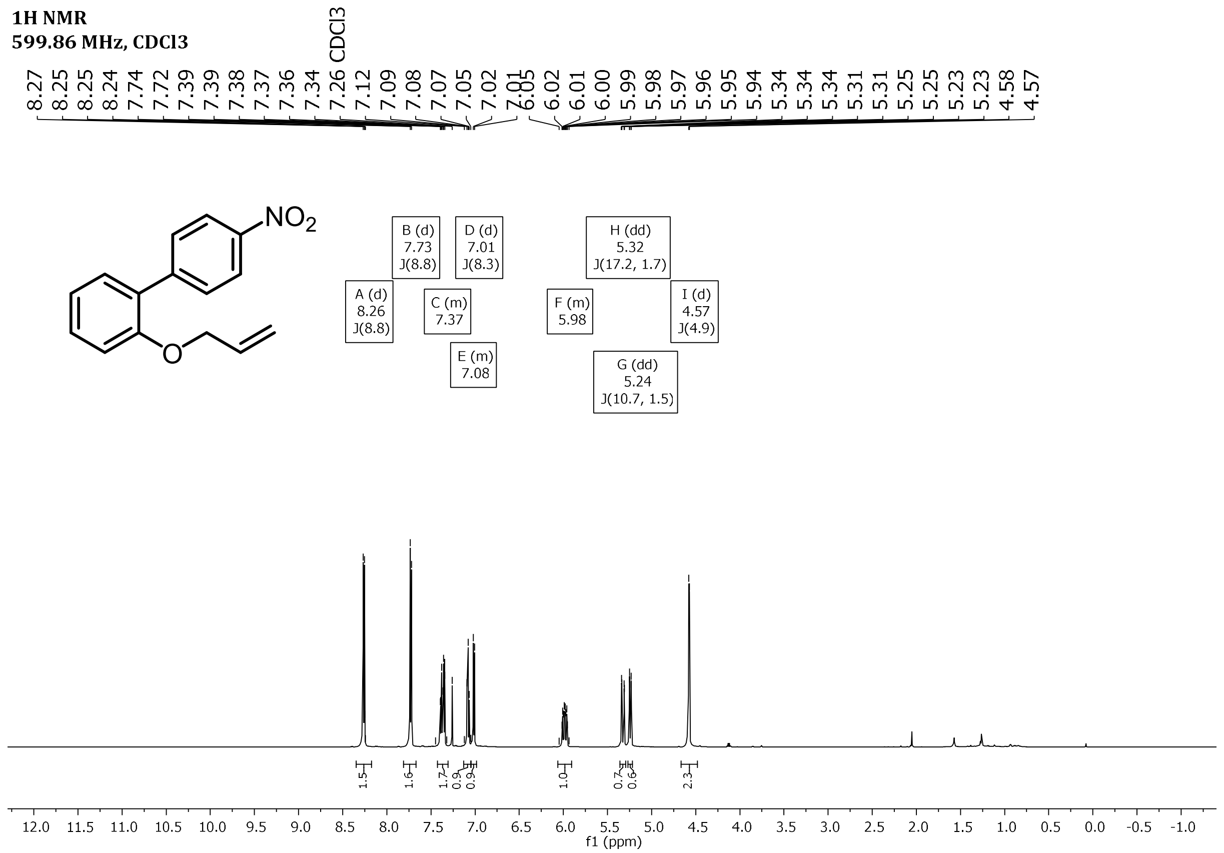

13C NMR
(CDCl3, 150.85 MHz)

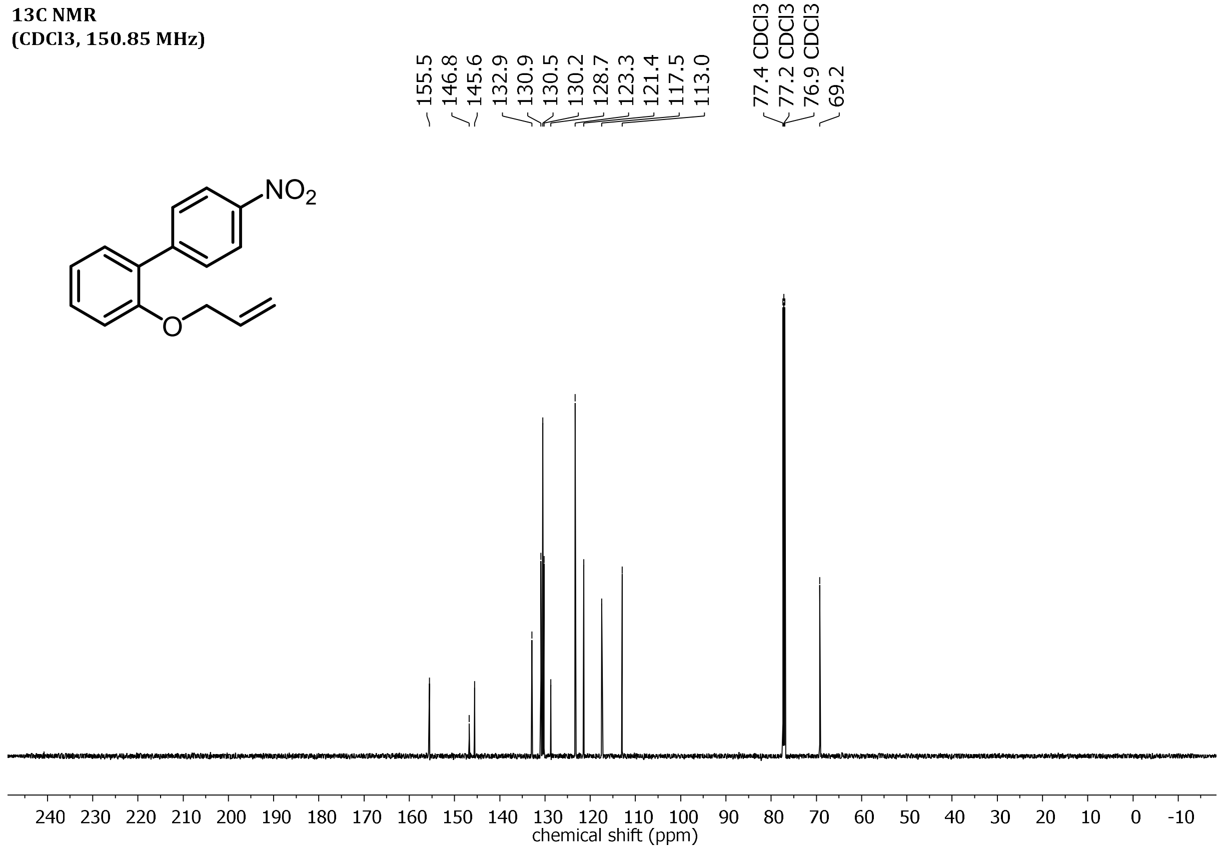

4-Bromo-4'-nitro-2-(trifluoromethoxy)-1,1'-biphenyl

1H NMR
599.86 MHz, CDCl3

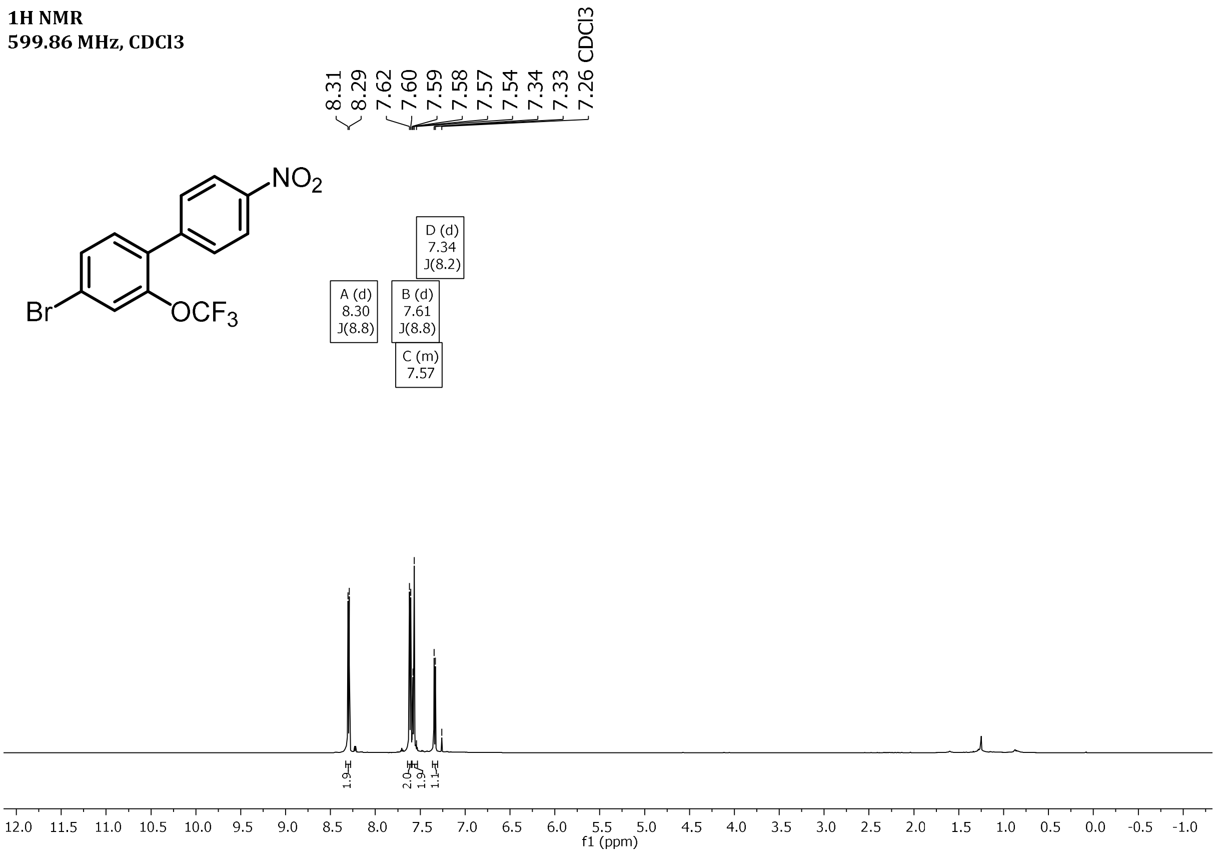

13C NMR
(CDCl3, 150.85 MHz)

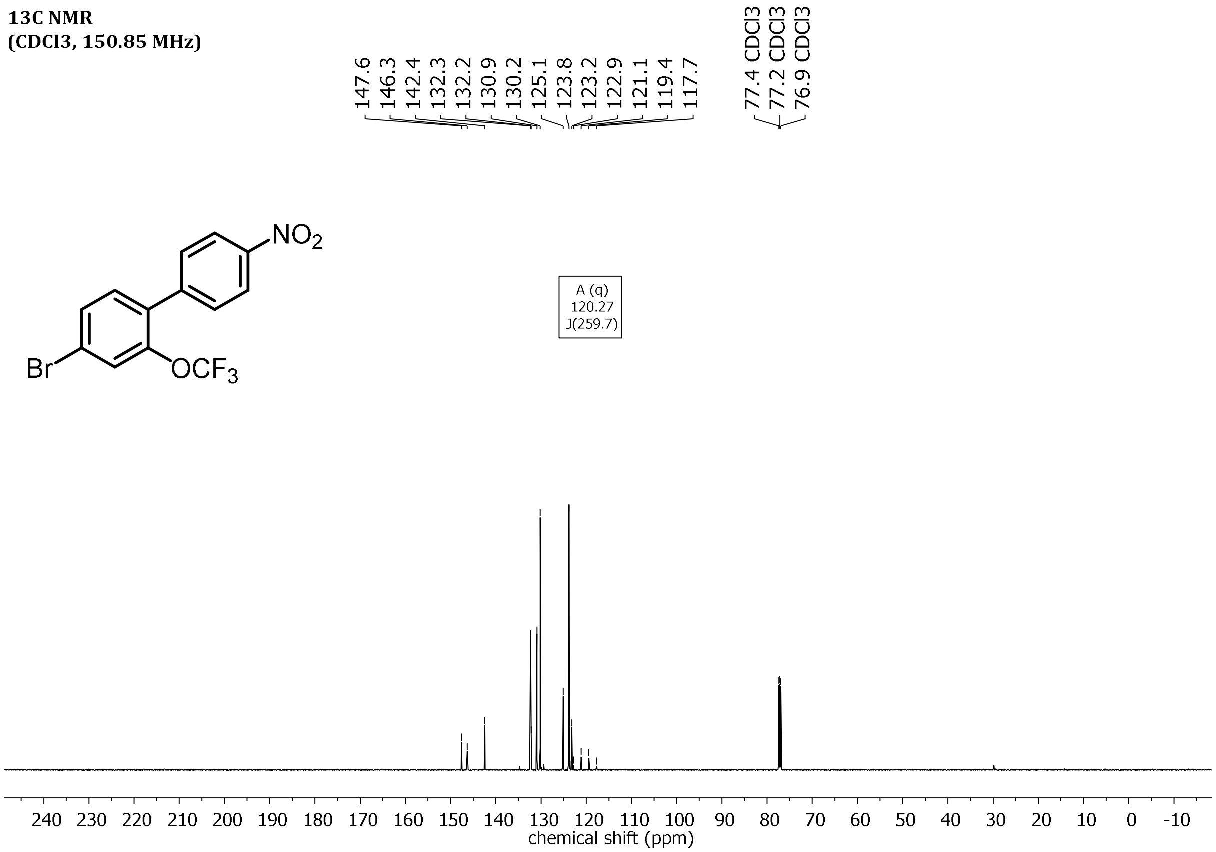

19F NMR
564.40 MHz, CDCl3

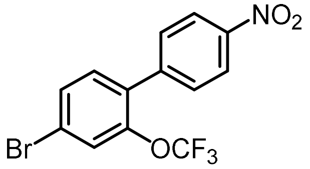

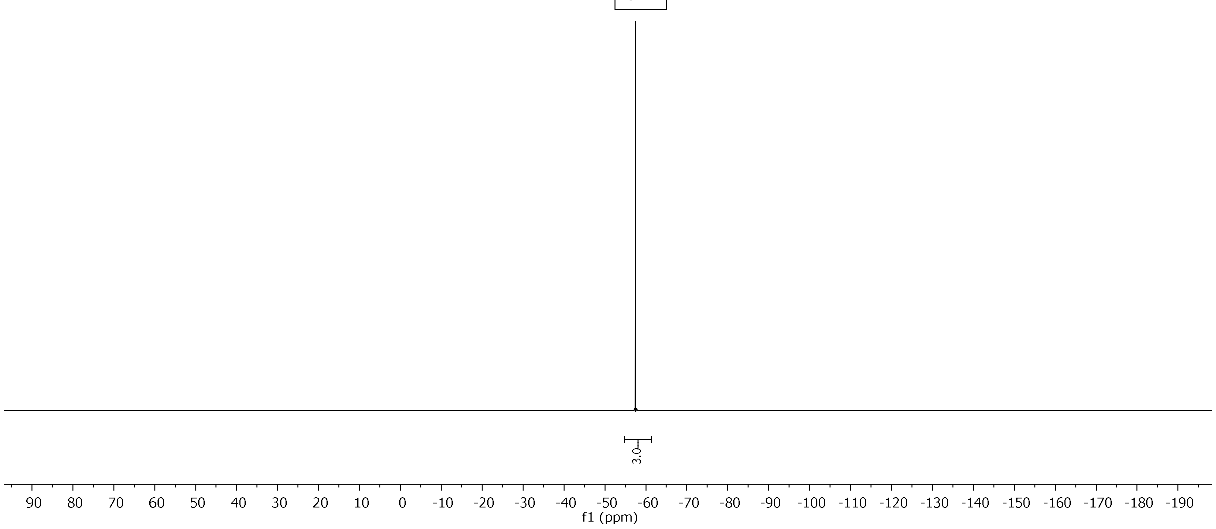

(5-Bromo-4'-nitro-[1,1'-biphenyl]-3-yl)trimethylsilane

1H NMR
(CDCl3, 399.97 MHz)

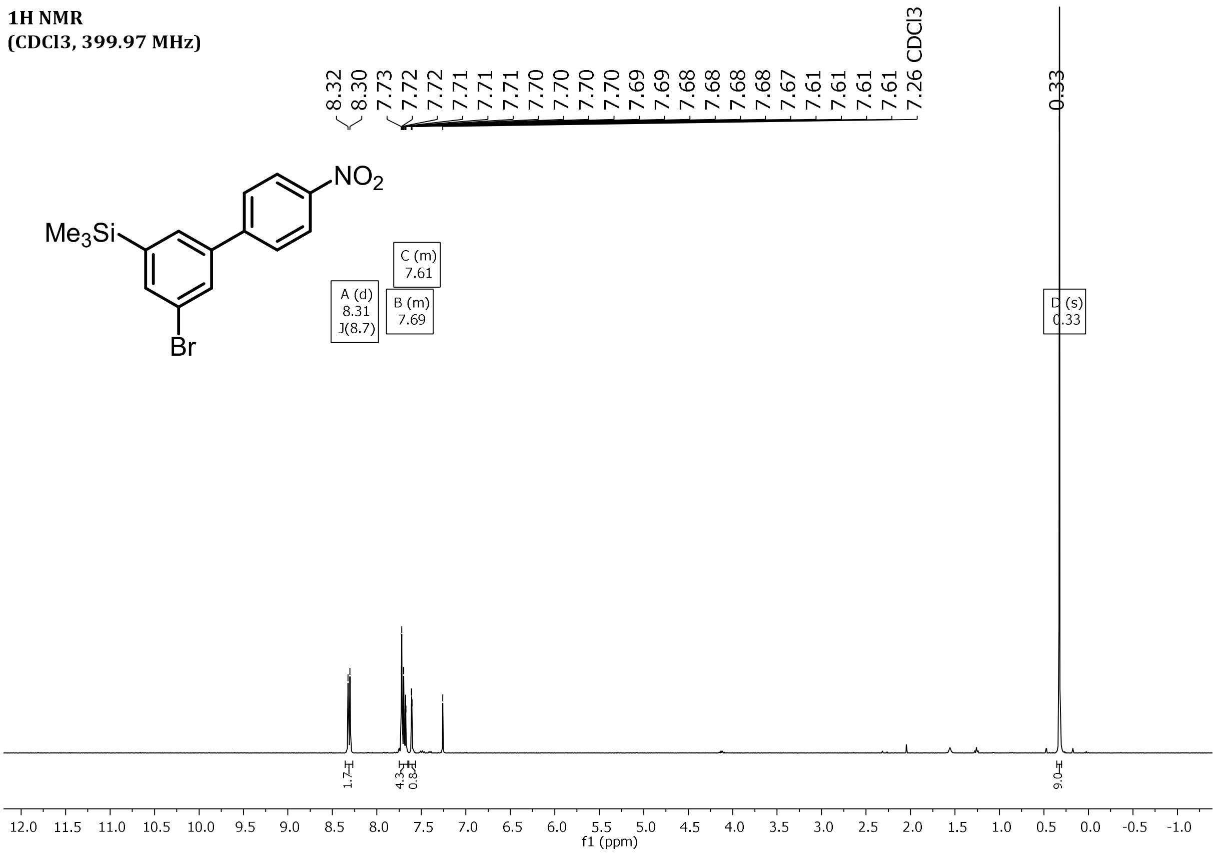

13C NMR
(CDCl3, 100.58 MHz)

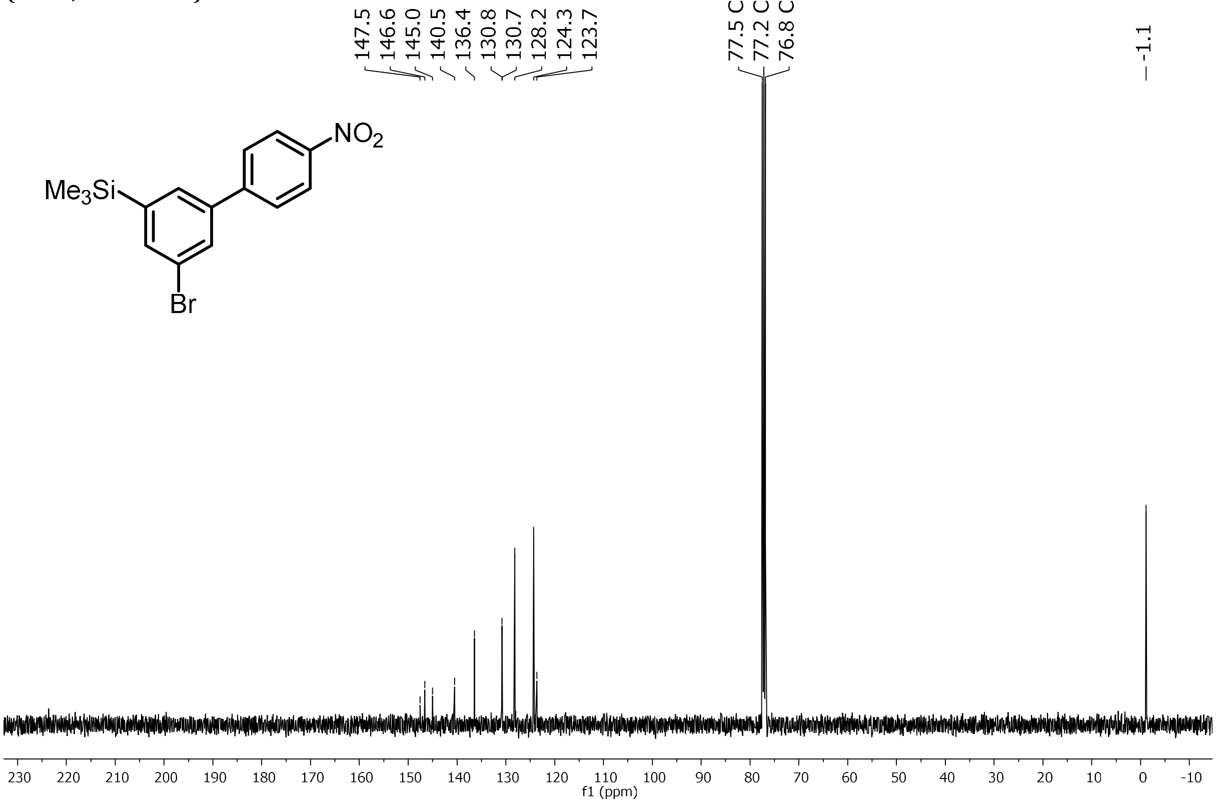

1-(4'-(Triethylsilyl)-[1,1'-biphenyl]-4-yl)ethan-1-one

1H NMR
(CDCl3, 399.97 MHz)

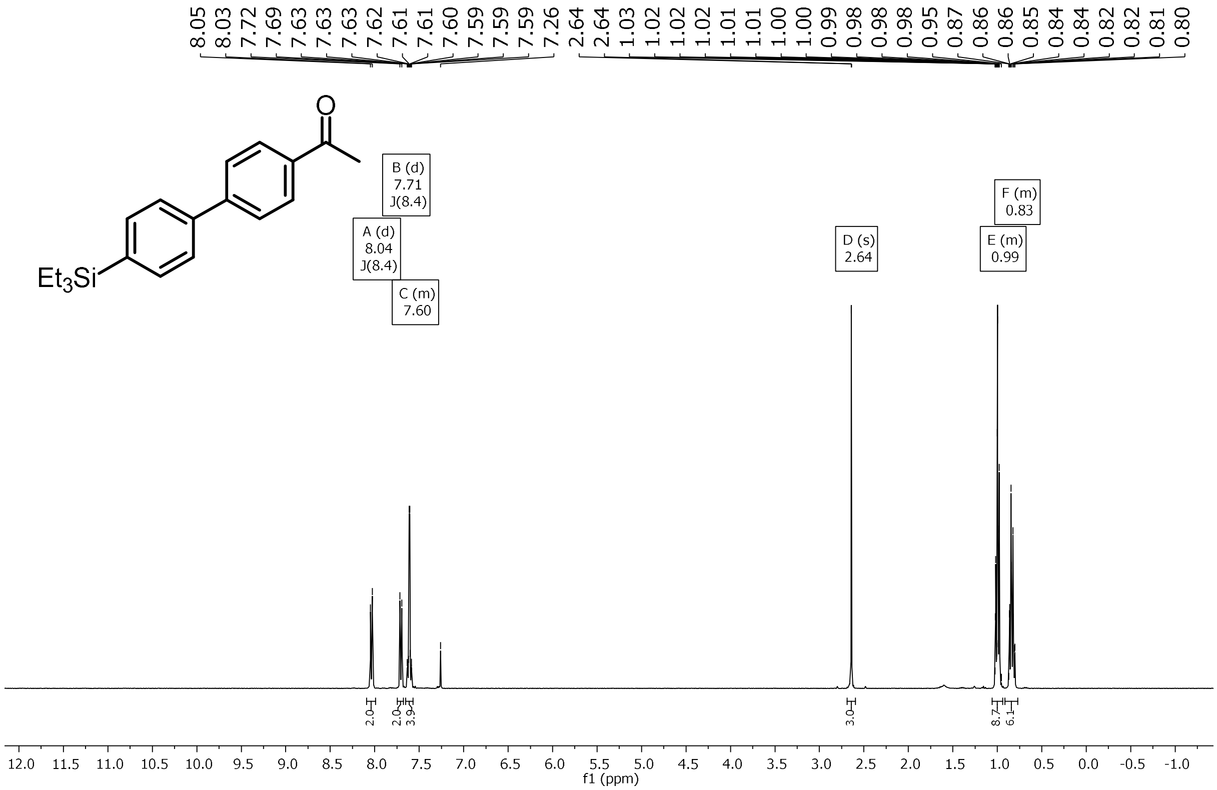

13C NMR
(CDCl3, 100.58 MHz)

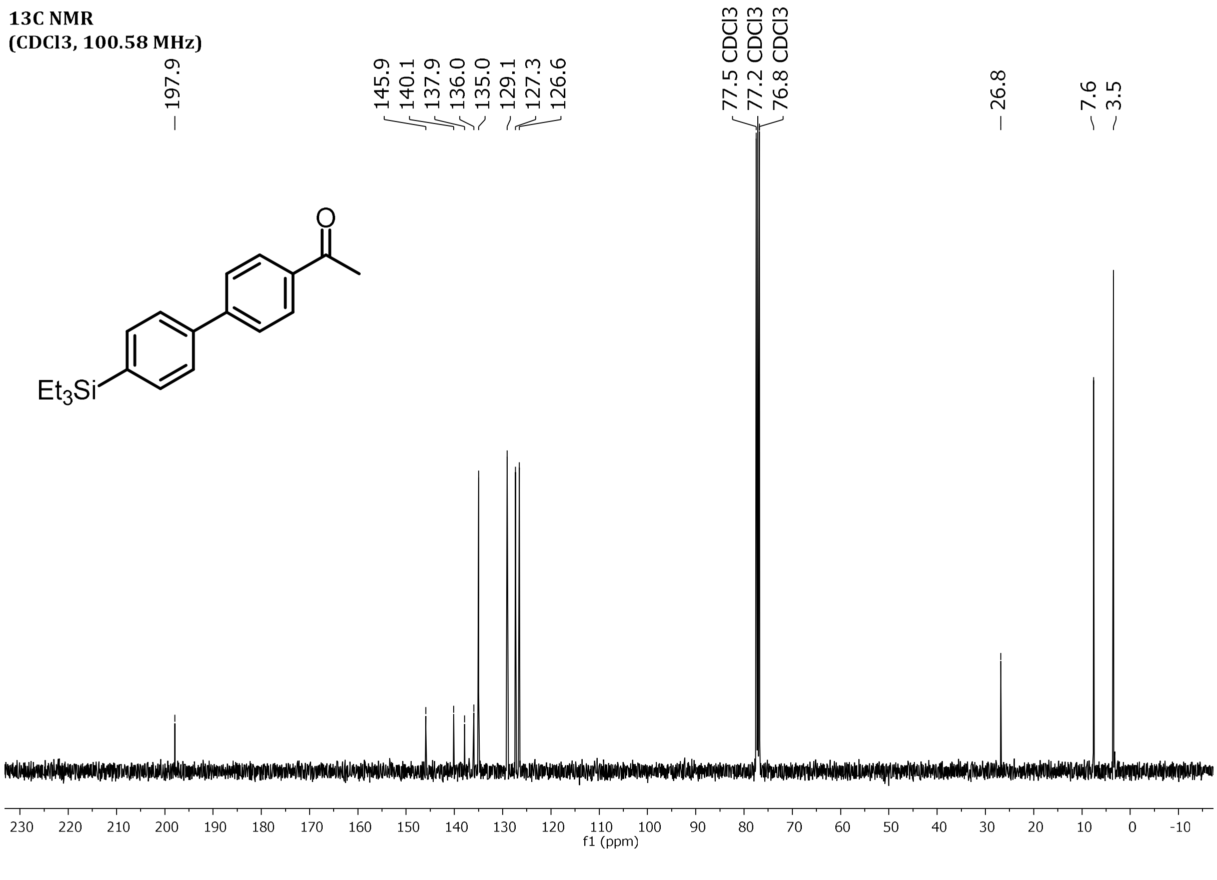

4-Chloro-3-fluoro-4'-iodo-1,1'-biphenyl

1H NMR
(CDCl3, 600.44 MHz)

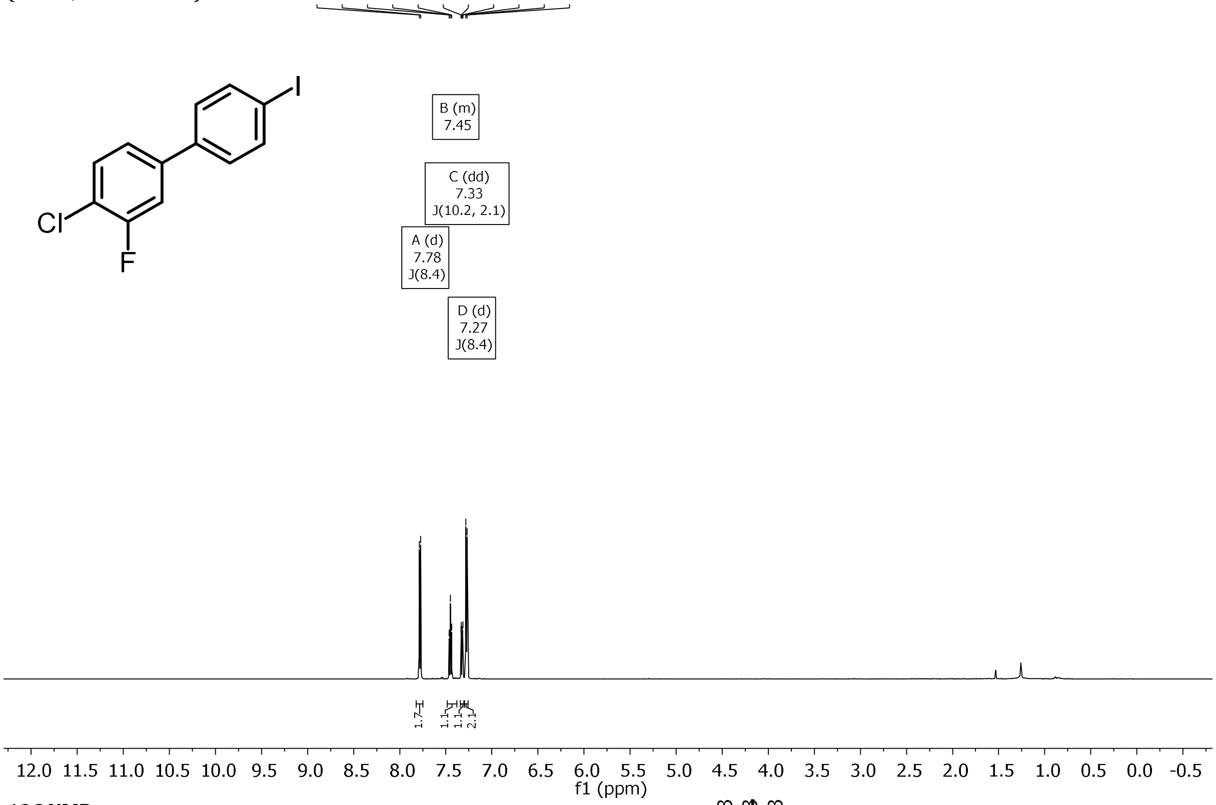

13C NMR
(CDCl3, 151.00 MHz)

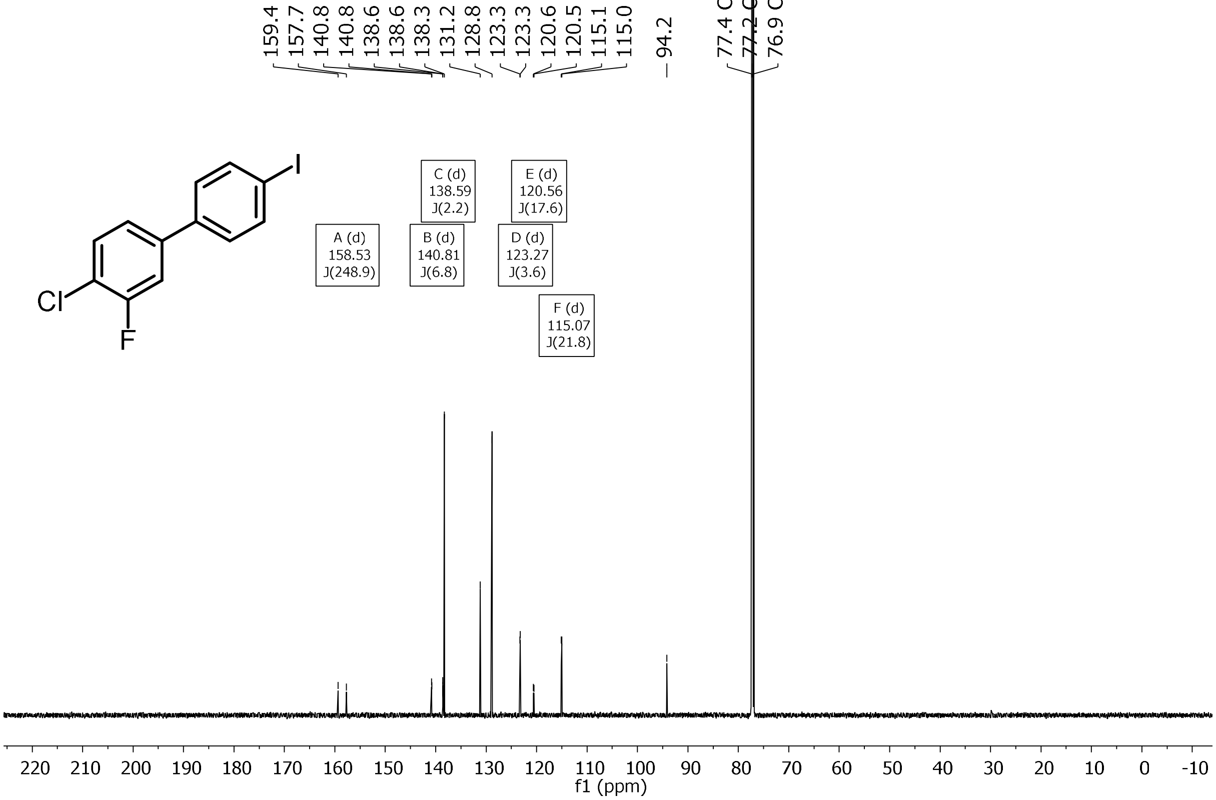

19F NMR
(CDCl3, 564.92 MHz)

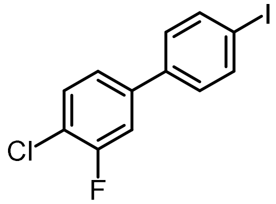

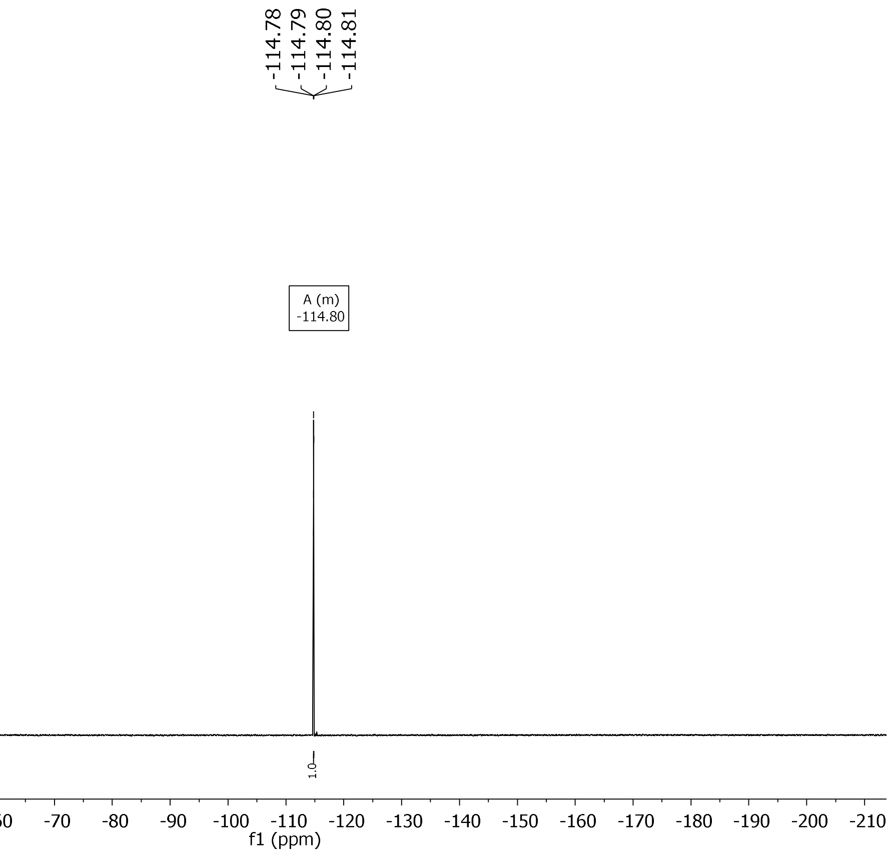

(5-Iodo-4'-nitro-[1,1'-biphenyl]-3-yl)trimethylsilane

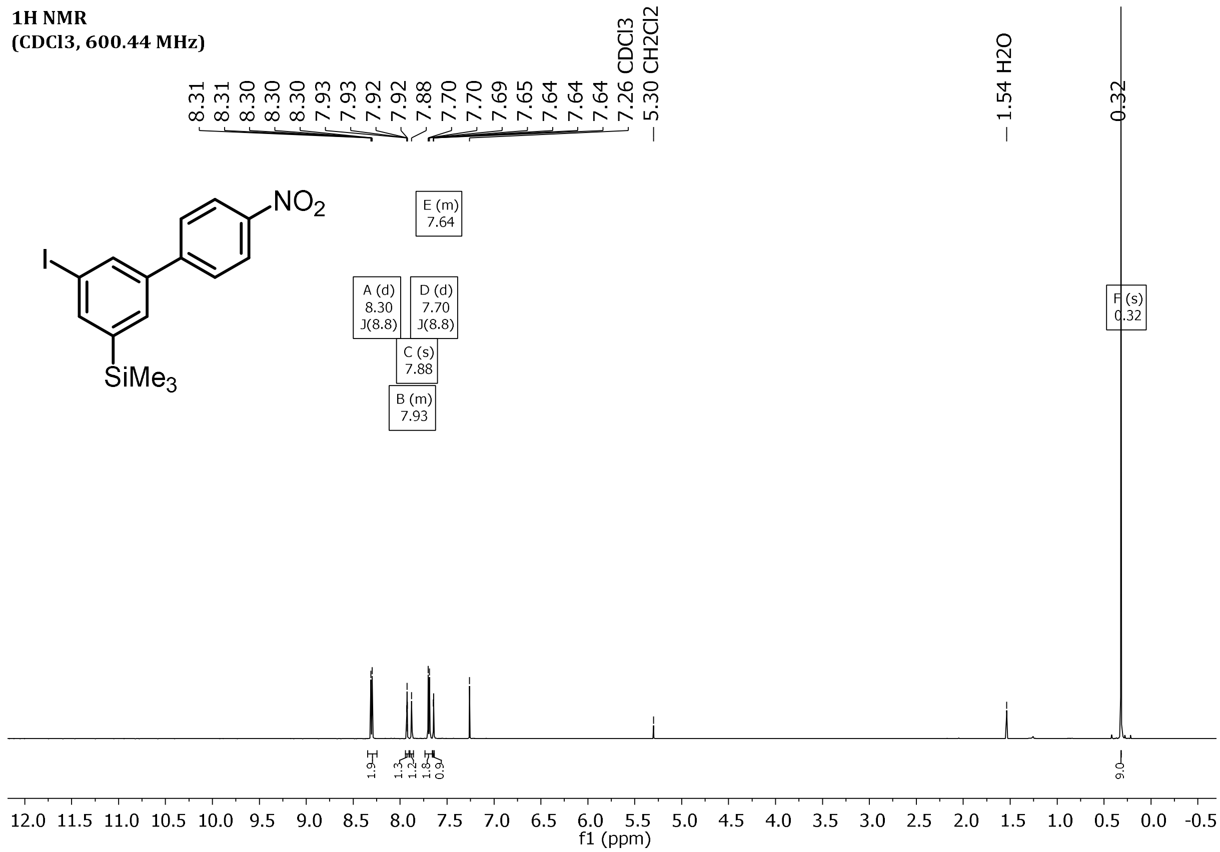

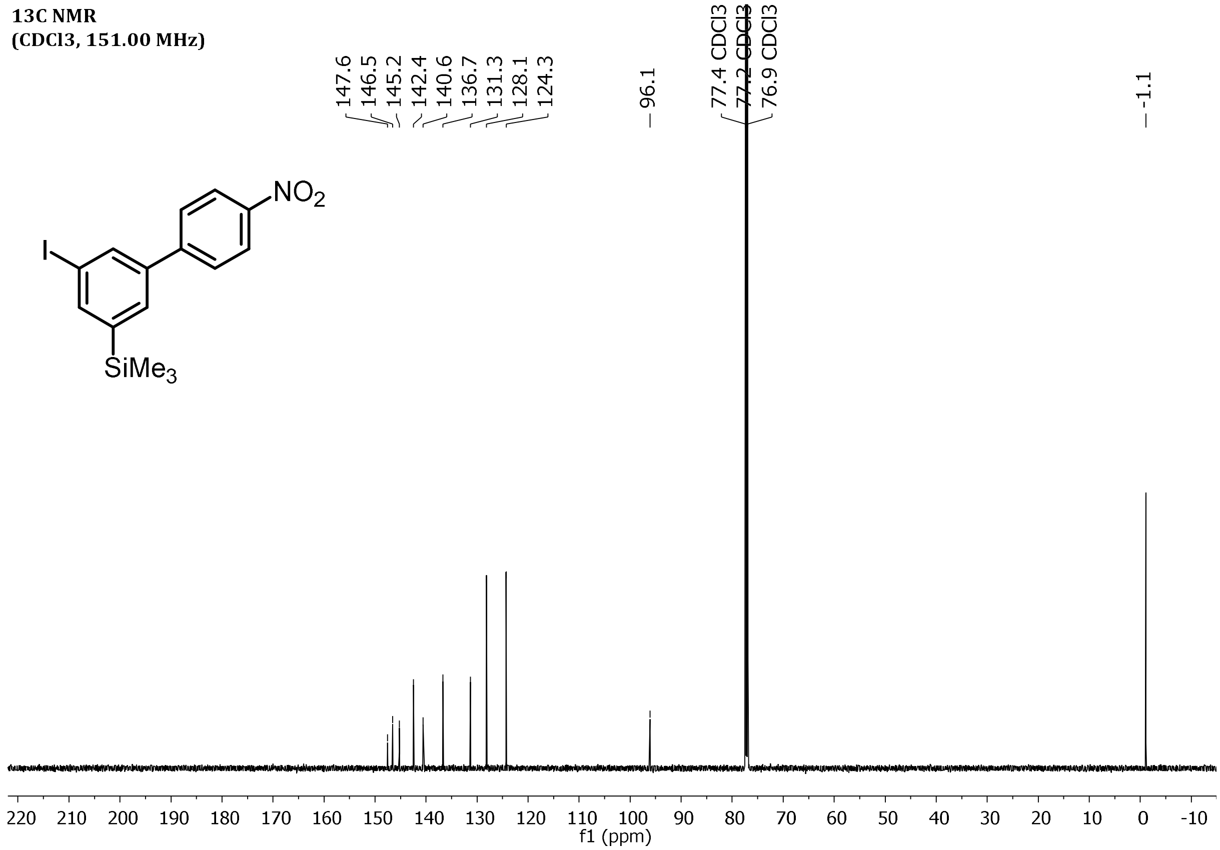

Trimethyl(5-(4,4,5,5-tetramethyl-1,3,2-dioxaborolan-2-yl)-4'-(trifluoromethyl)-[1,1'-biphenyl]-3-yl)silane

1H NMR
(CDCl3, 399.97 MHz)

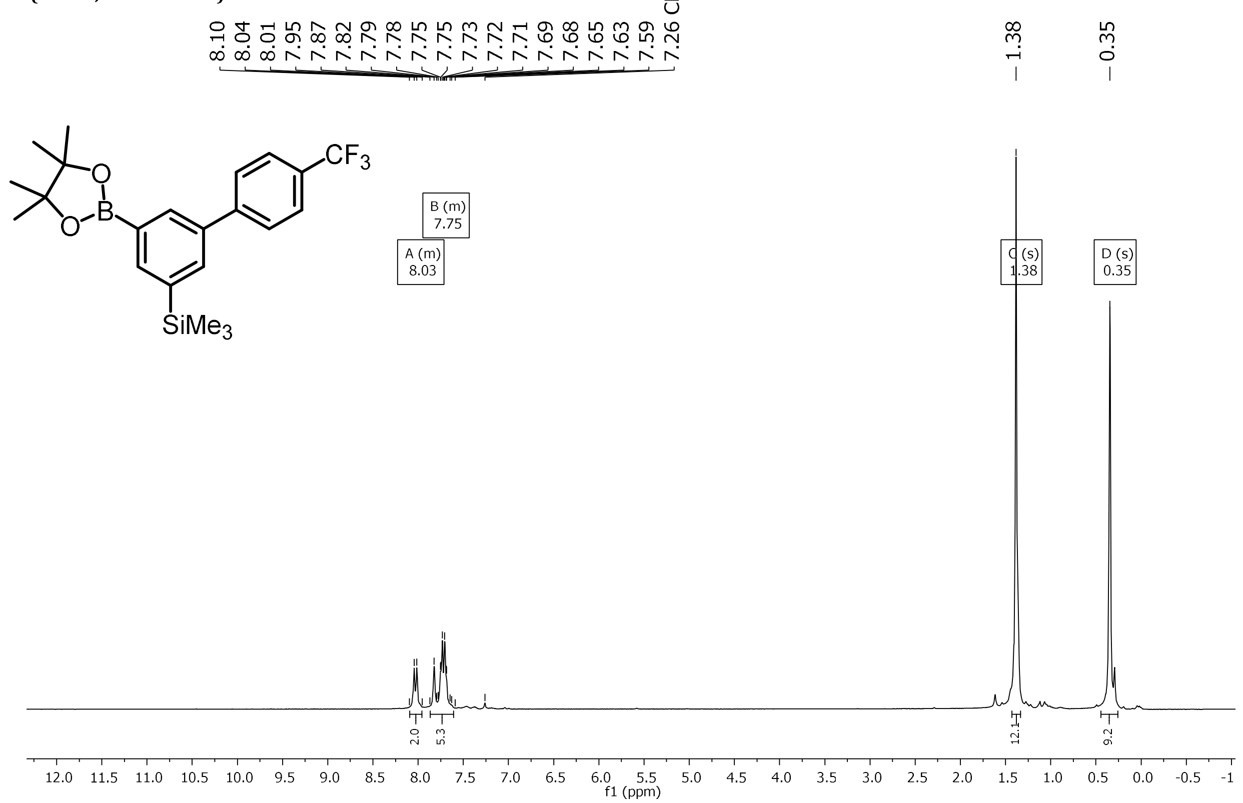

13C NMR
(CDCl3, 100.58 MHz)

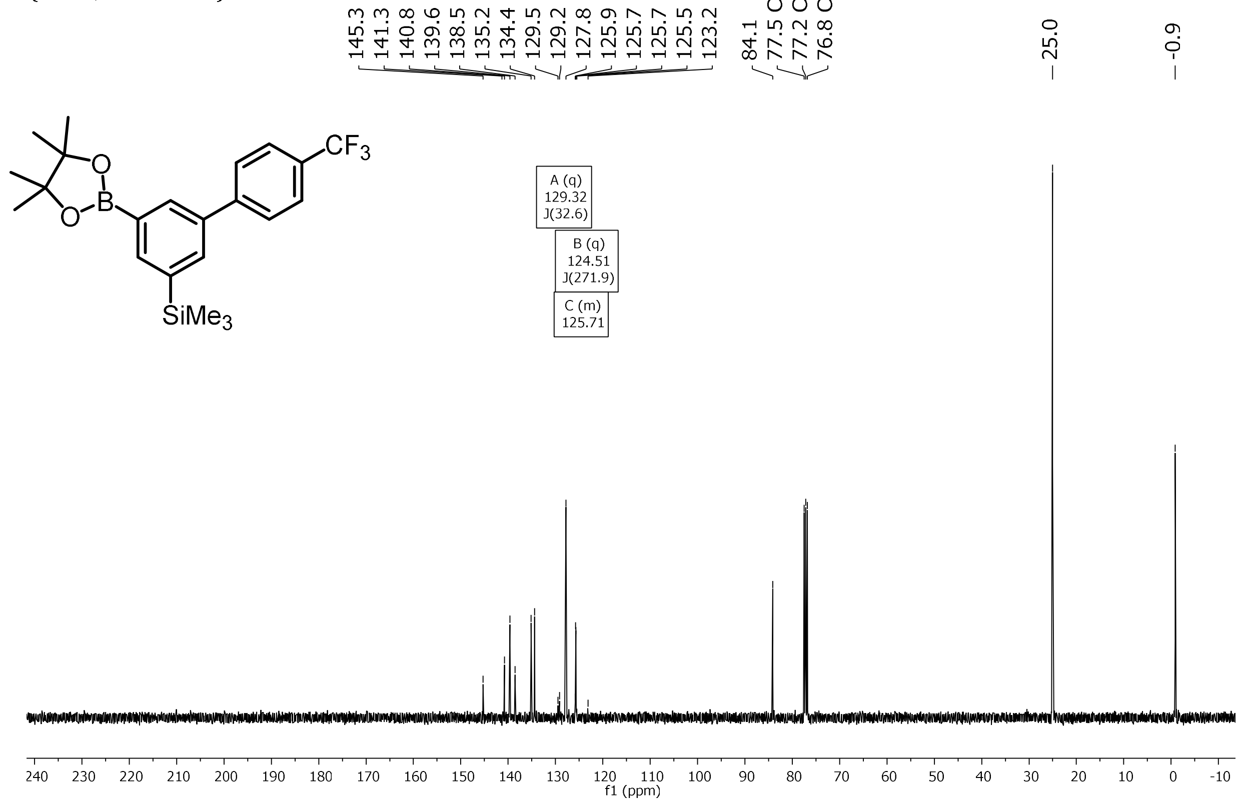

19F NMR
(CDCl3, 376.33 MHz)

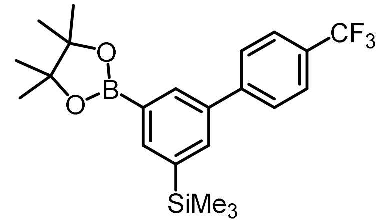

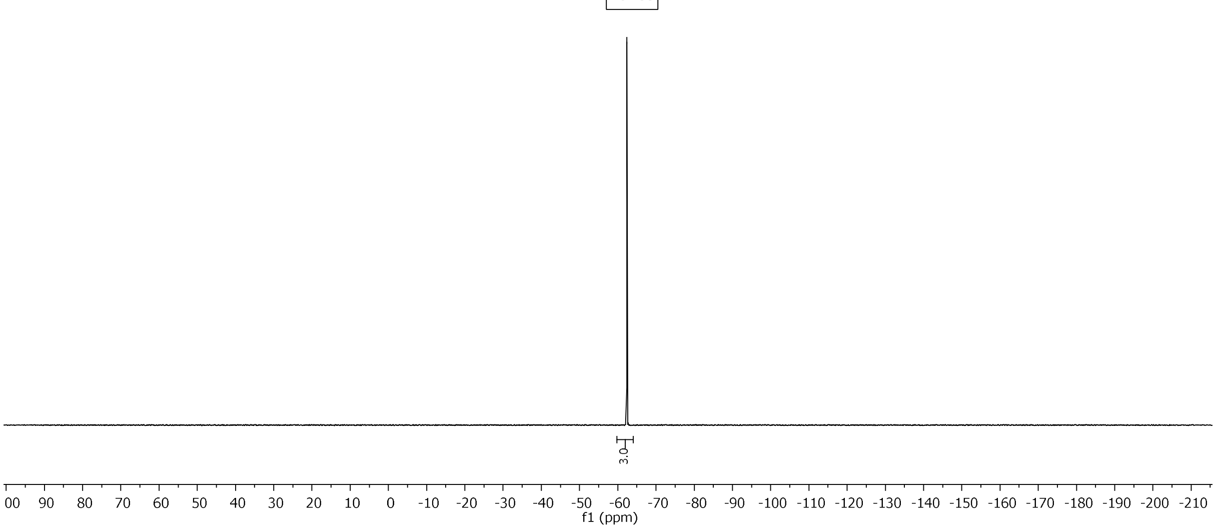

4'-(*tert*-Butyl)-4-chloro-3-fluoro-1,1'-biphenyl

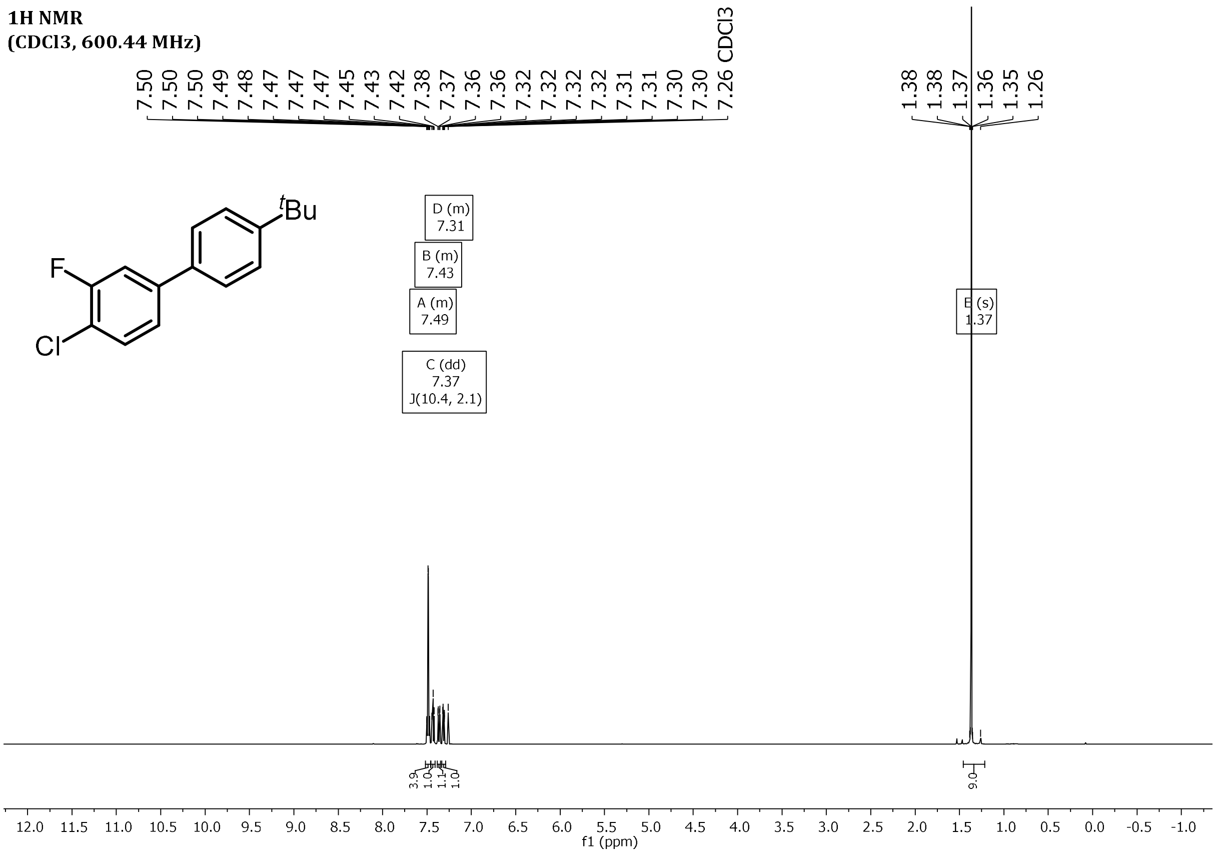

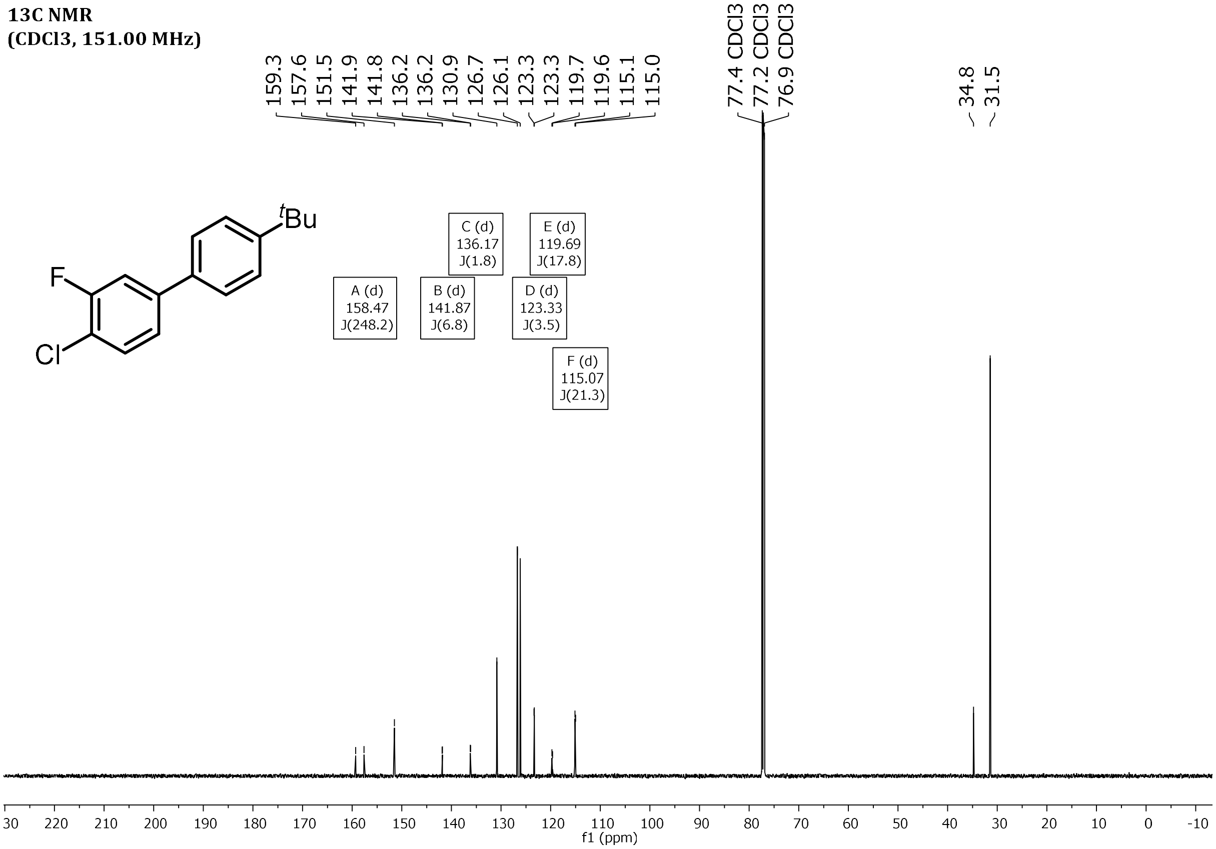

19F NMR
(CDCl3, 564.92 MHz)

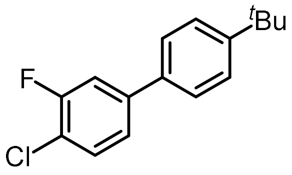

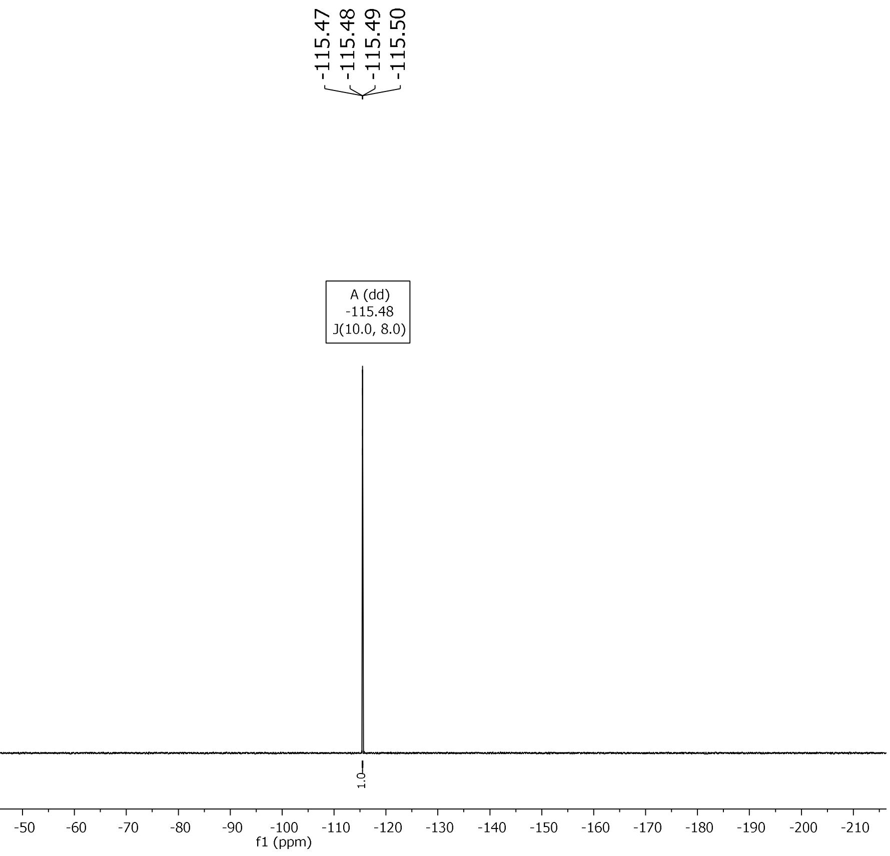

(5-Bromo-4'-(*tert*-butyl)-[1,1'-biphenyl]-3-yl)trimethylsilane

1H NMR
(CDCl3, 600.44 MHz)

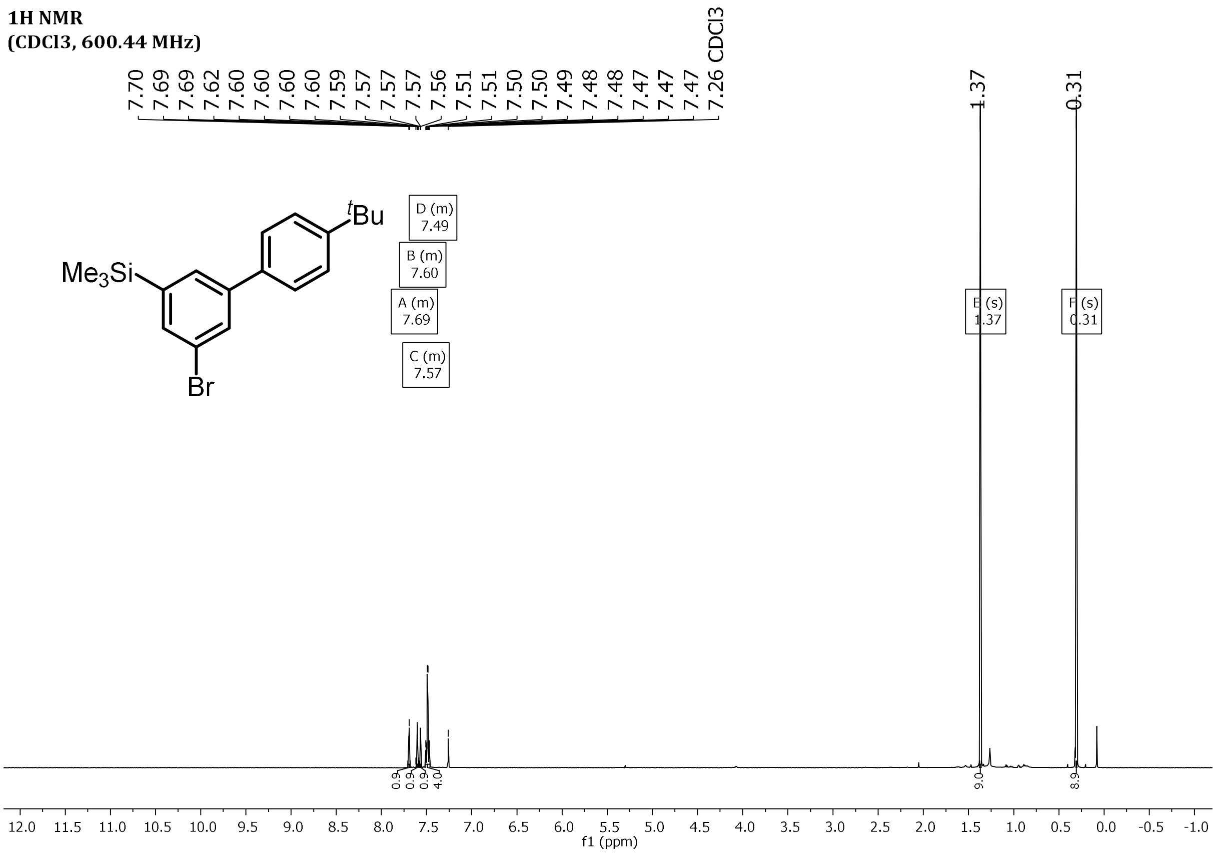

13C NMR
(CDCl3, 151.00 MHz)

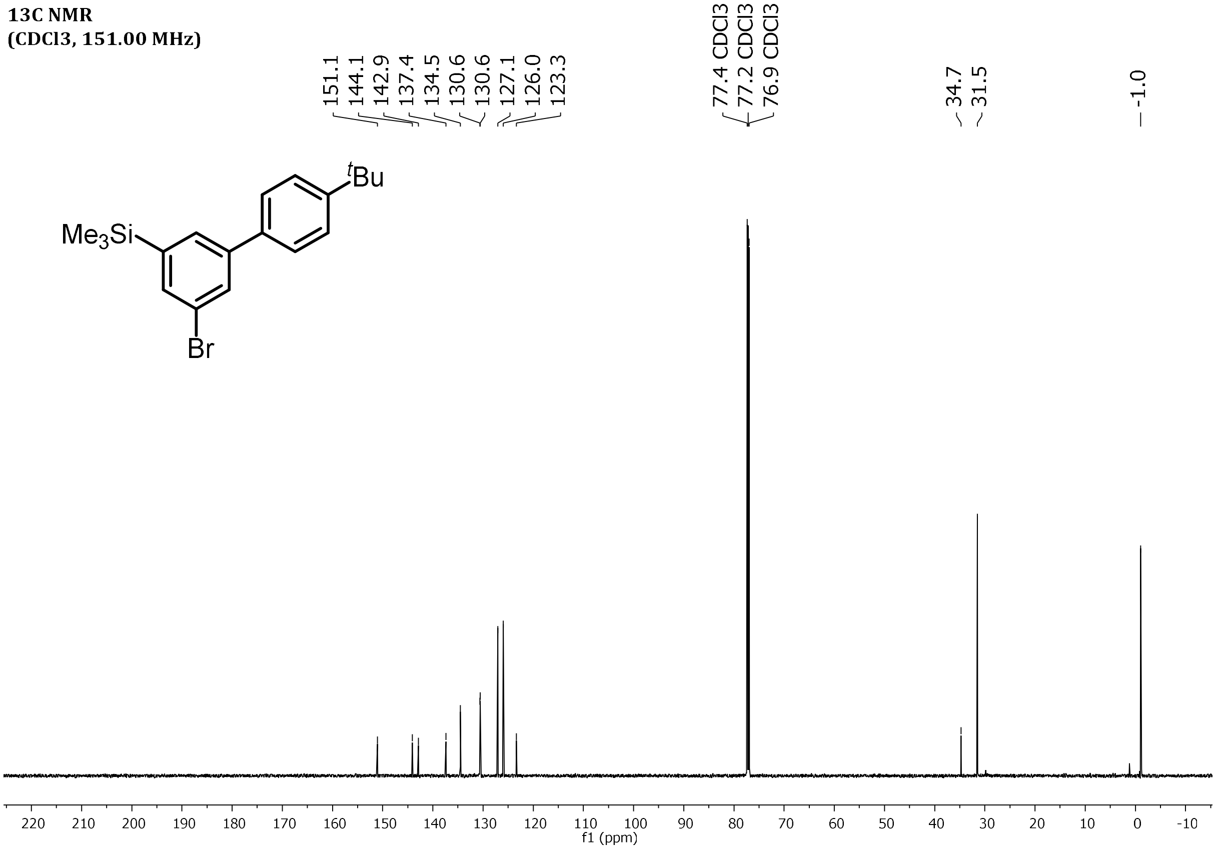

(4-Bromo-3-methylphenyl)triethylgermane

1H NMR
(CDCl3, 399.97)

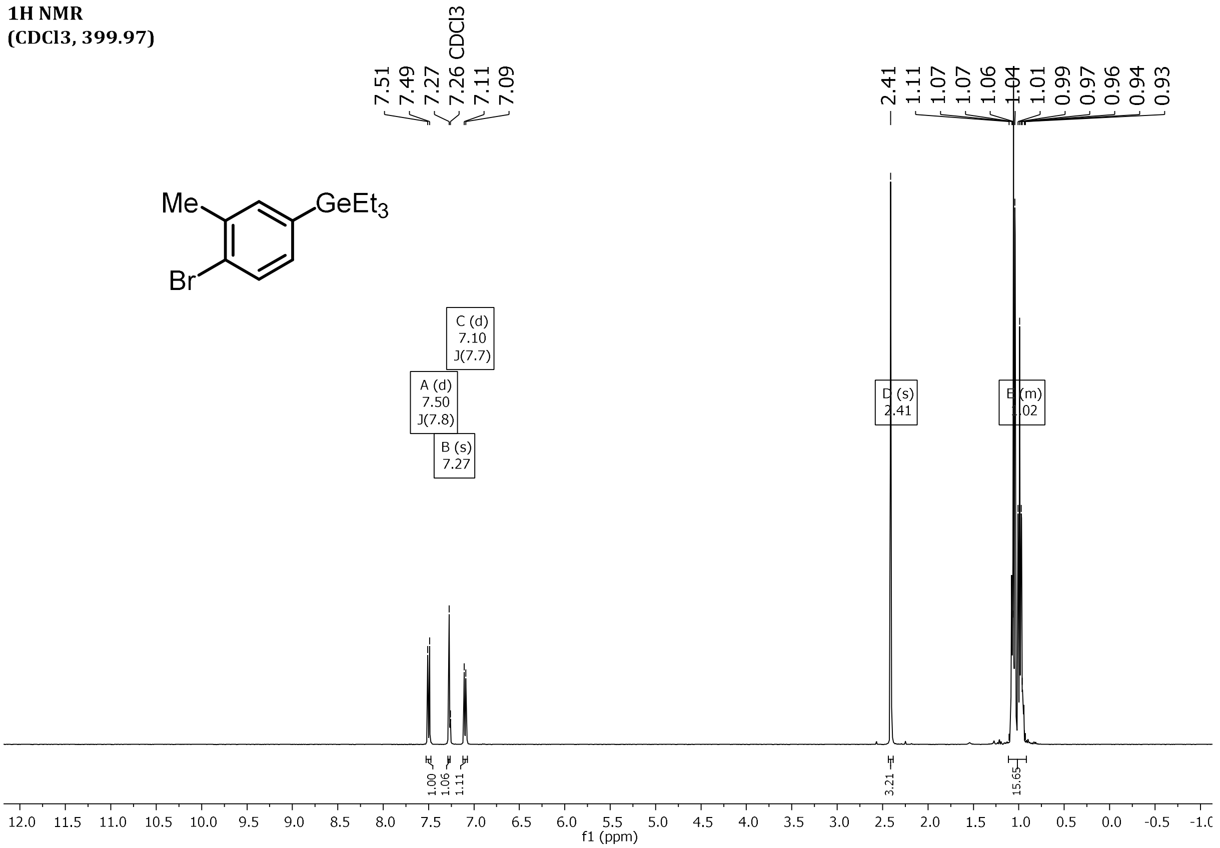

13C NMR
(CDCl3, 100.58 MHz)

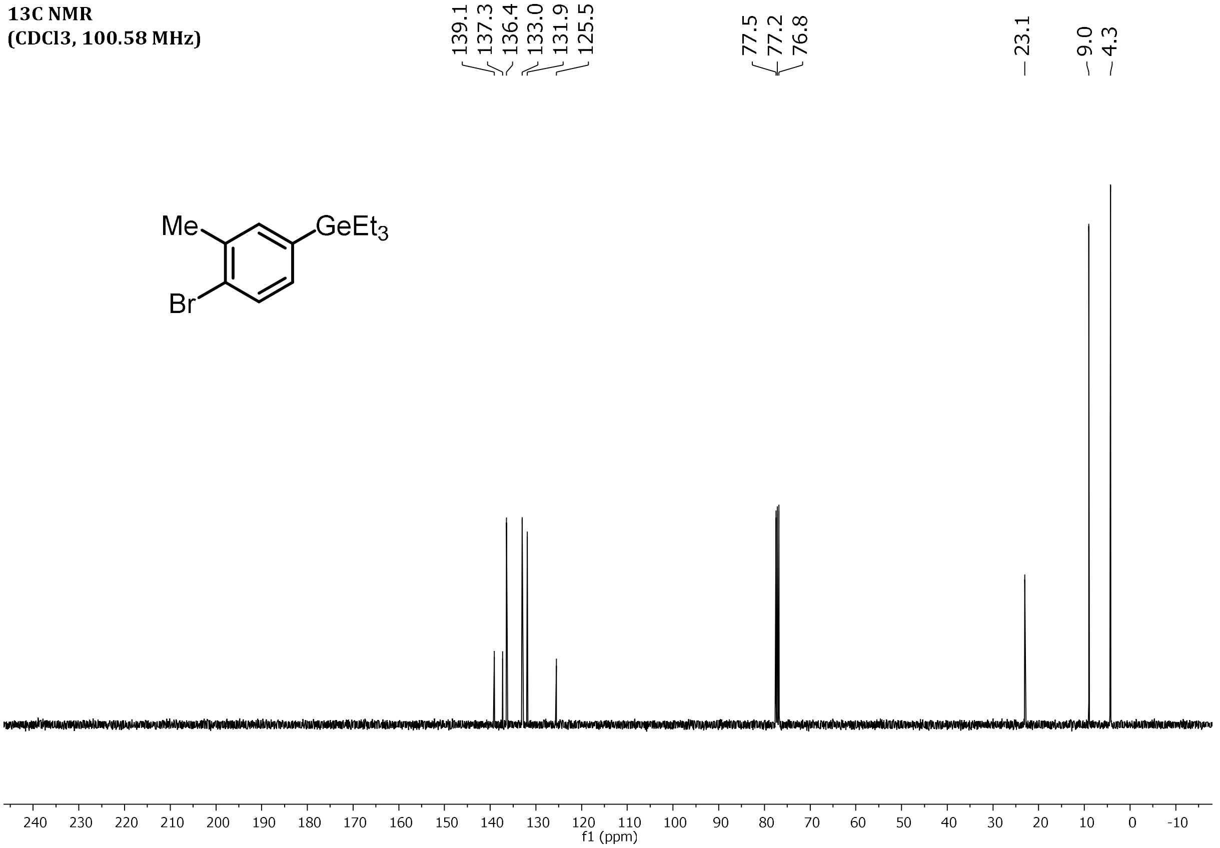

Triethyl(2,4,6-trifluorophenyl)germane

1H NMR
(CDCl3, 599.86 MHz)

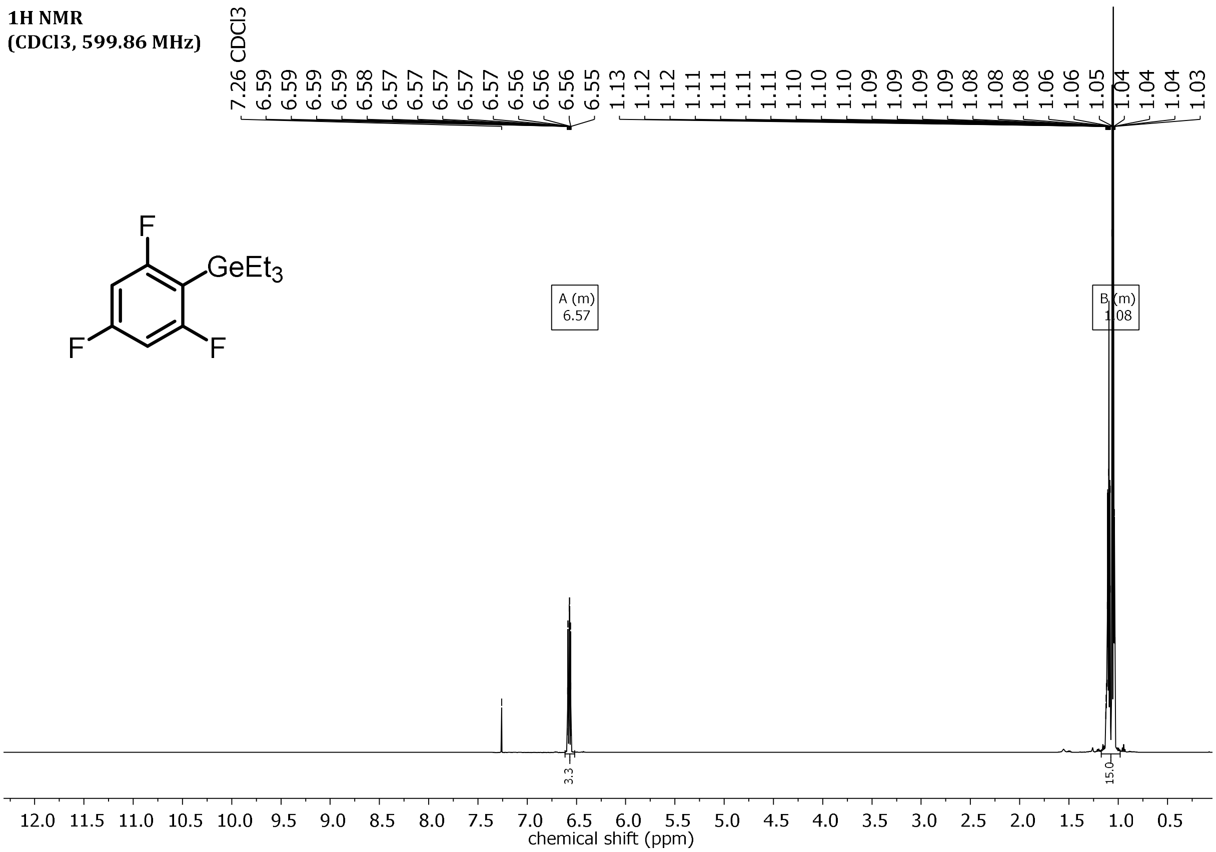

13C NMR
(CDCl3, 150.85 MHz)

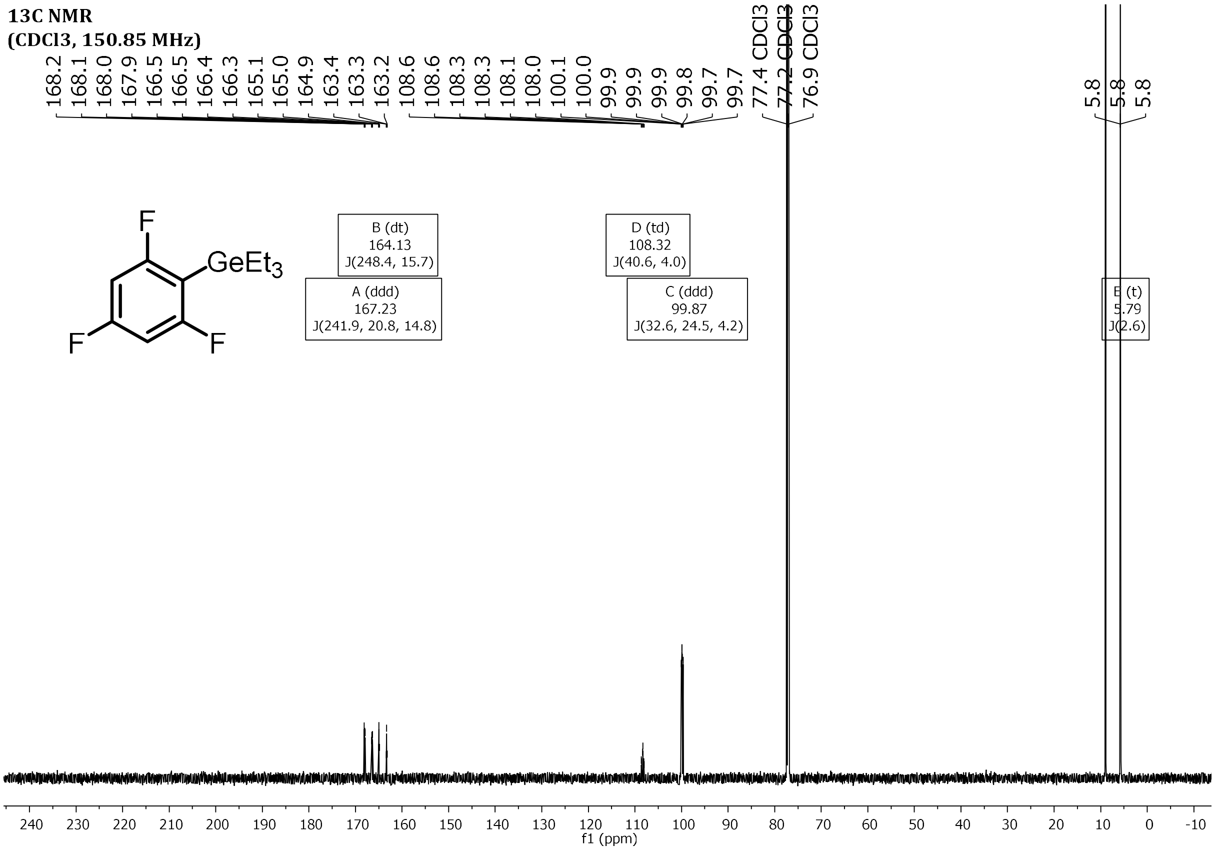

19F NMR
(CDCl3, 564.40 MHz)

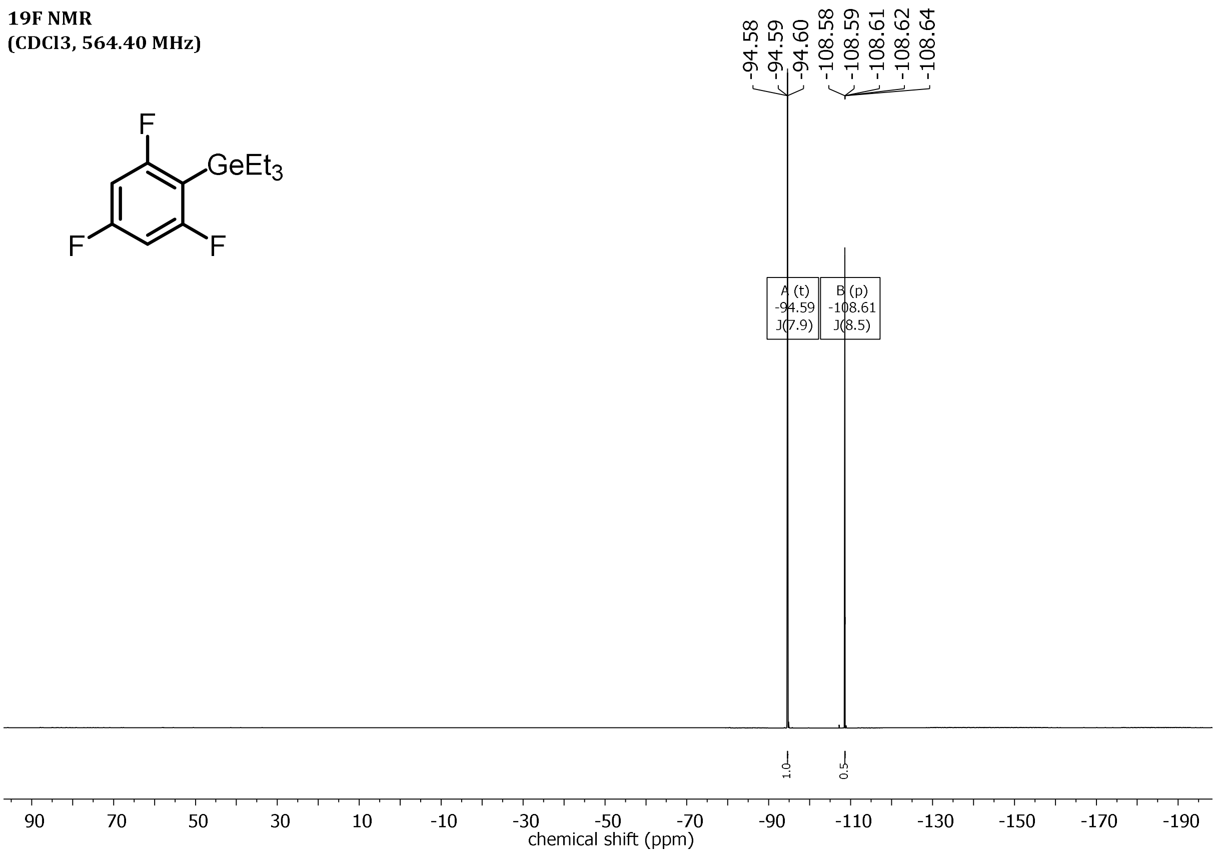

Triethyl(2-isopropylphenyl)germane

1H NMR
(CDCl3, 599.86 MHz)

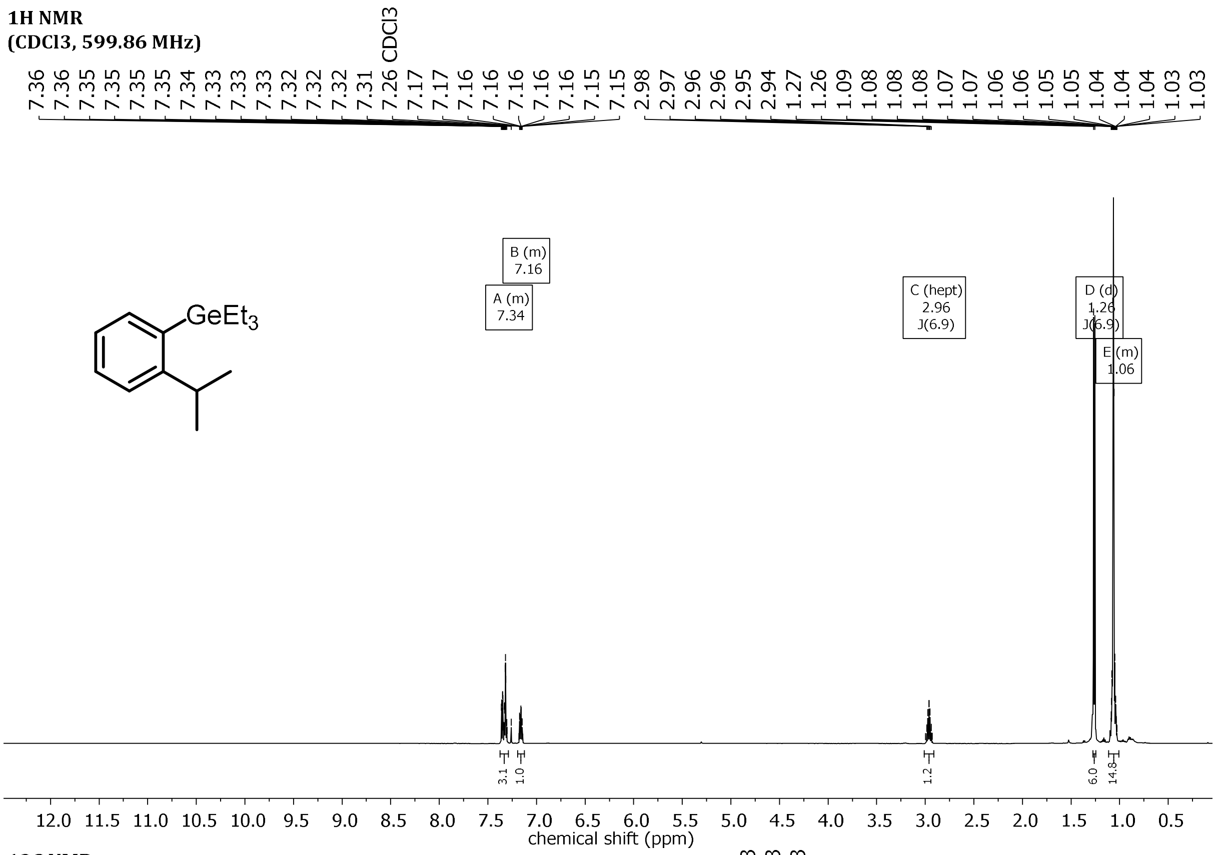

13C NMR
(CDCl3, 150.85 MHz)

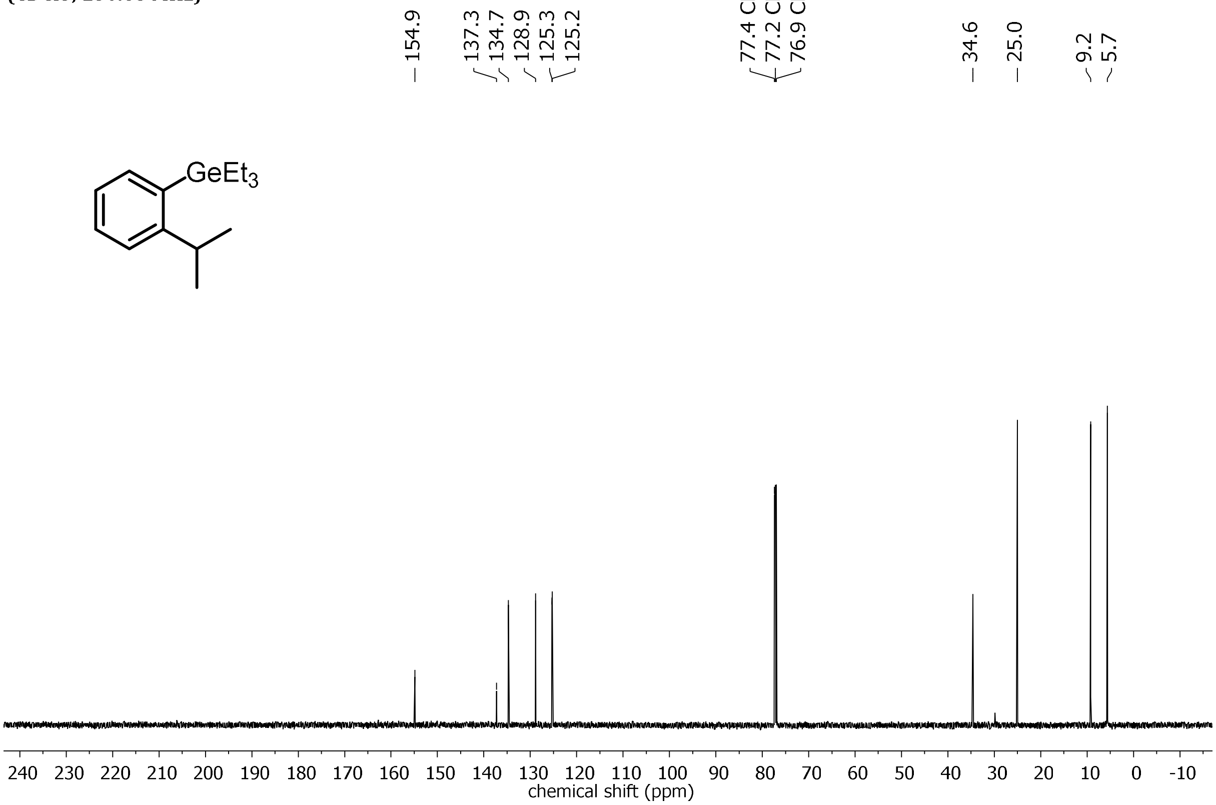

(4-Bromo-2-(trifluoromethoxy)phenyl)triethylgermane

1H NMR
(CDCl3, 599.86)

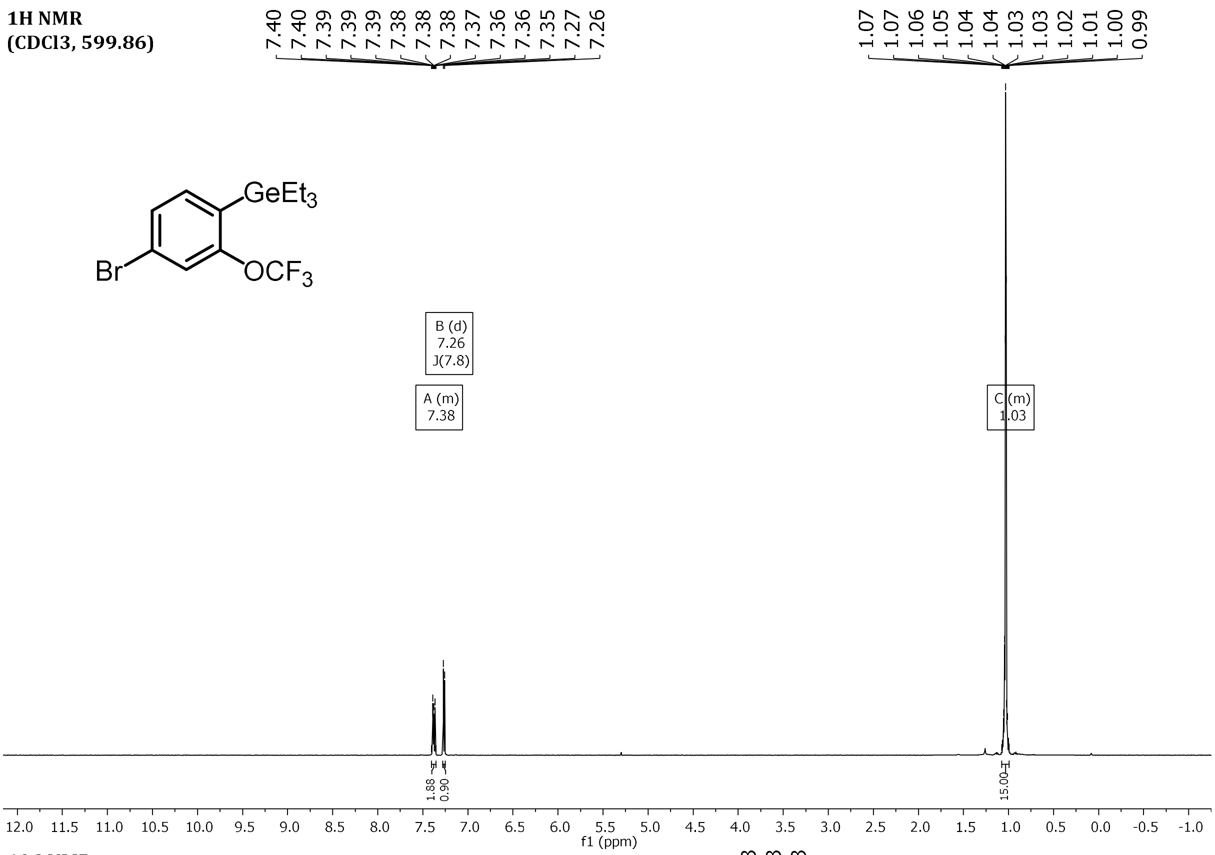

13C NMR
(CDCl3, 150.85 MHz)

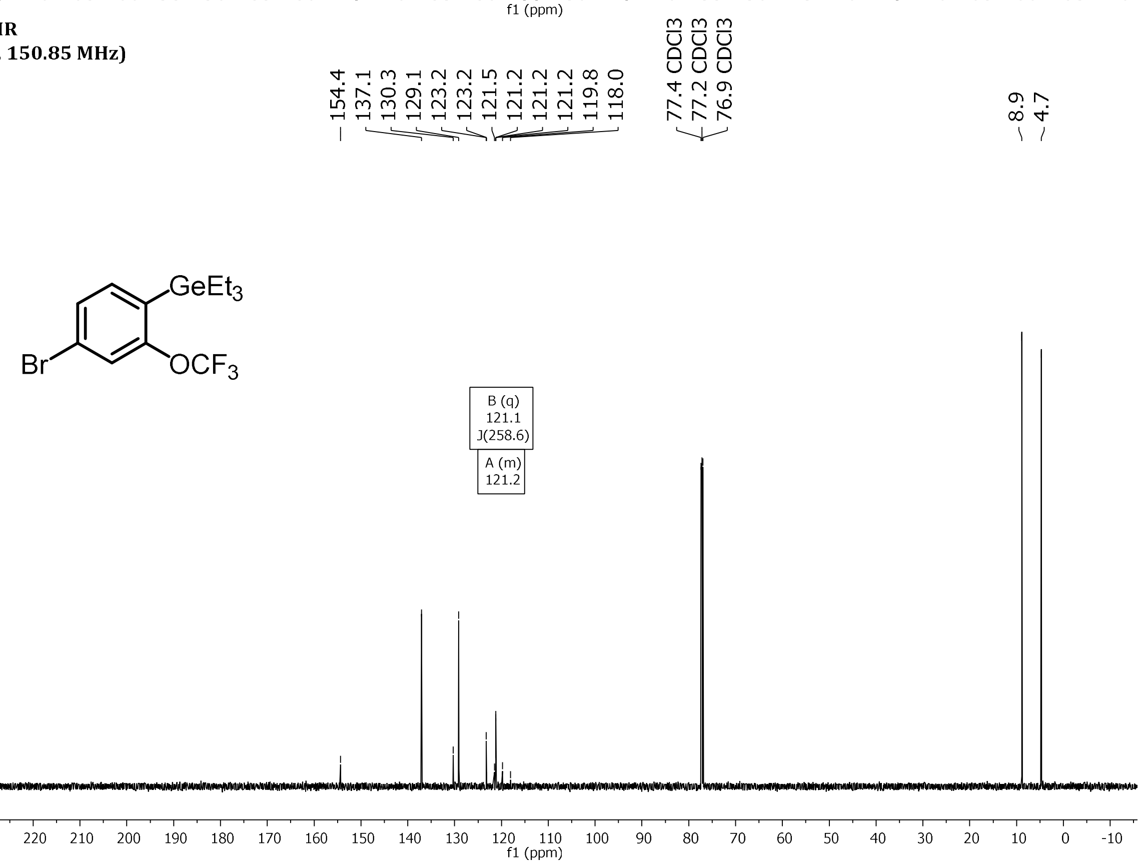

19F NMR
(564.38 MHz, CDCl3)

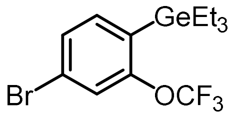

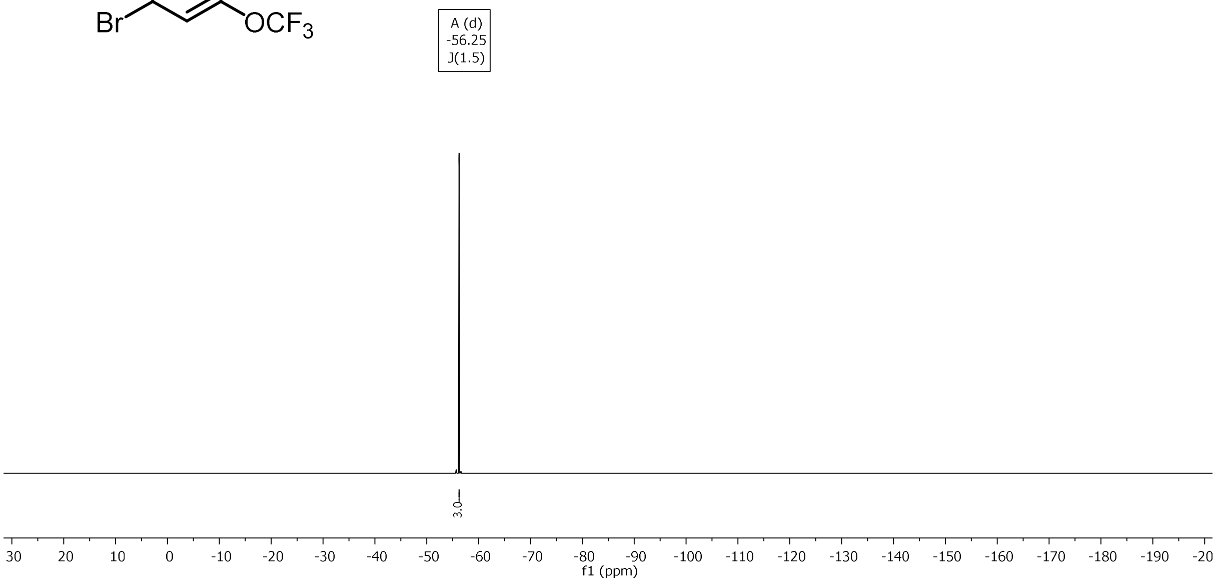

Triethyl(2-methoxyphenyl)germane

1H NMR
(CDCl3, 600.44 MHz)

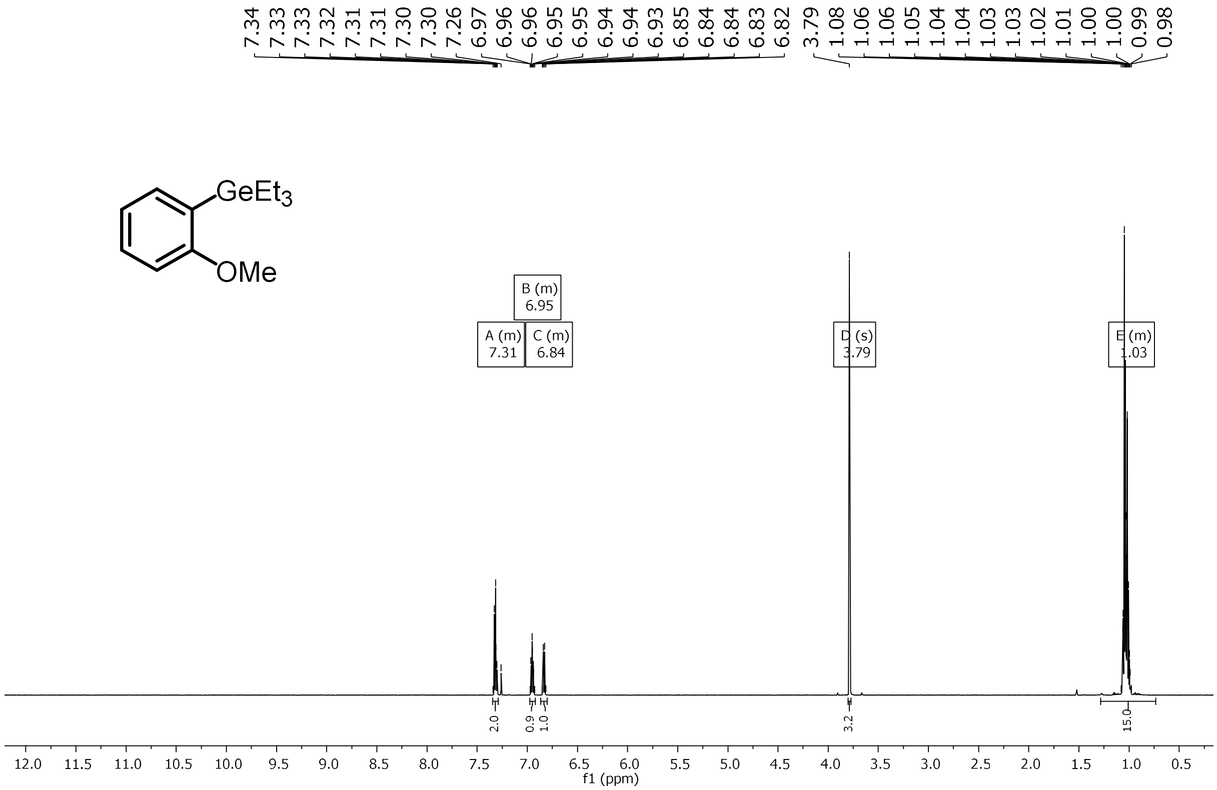

13C NMR
(CDCl3, 151.00 MHz)

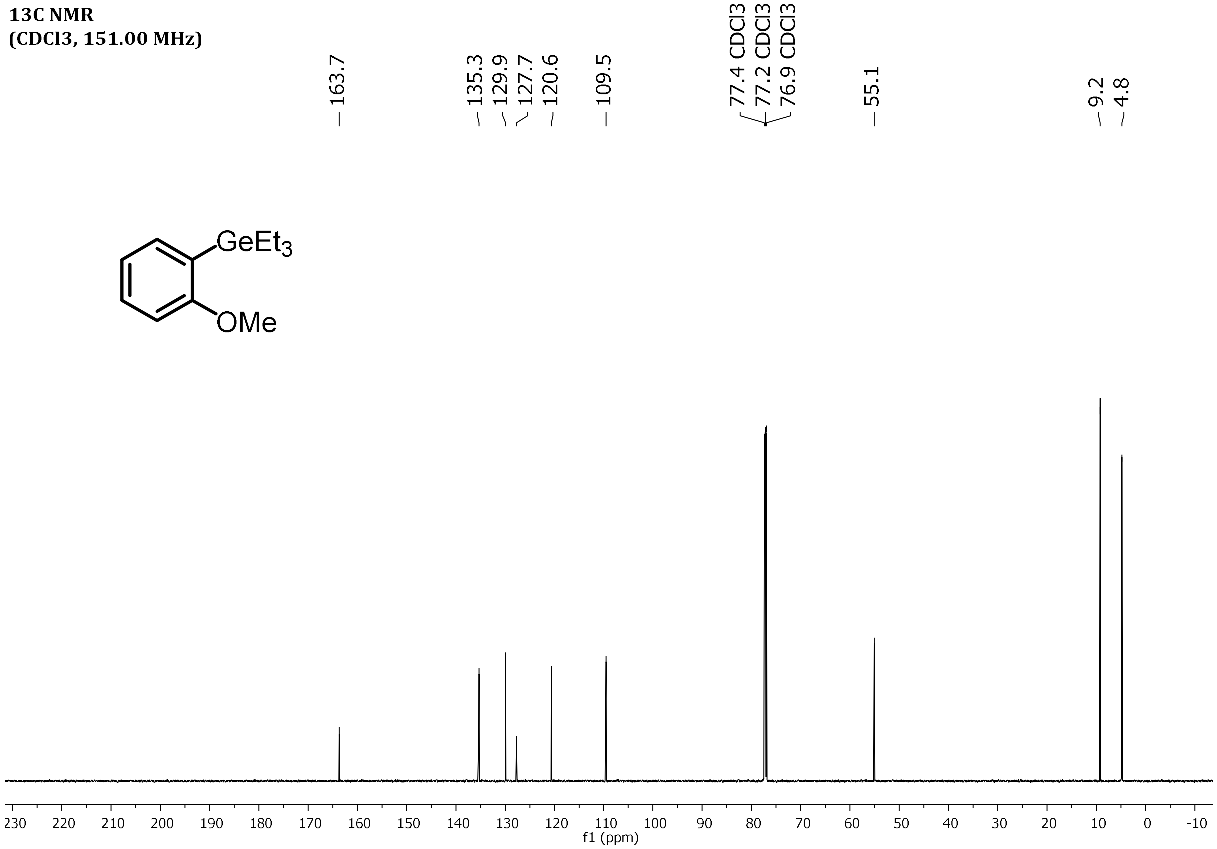

(3-Bromo-5-chlorophenyl)triethylgermane

1H NMR
(CDCl3, 300.03 MHz)

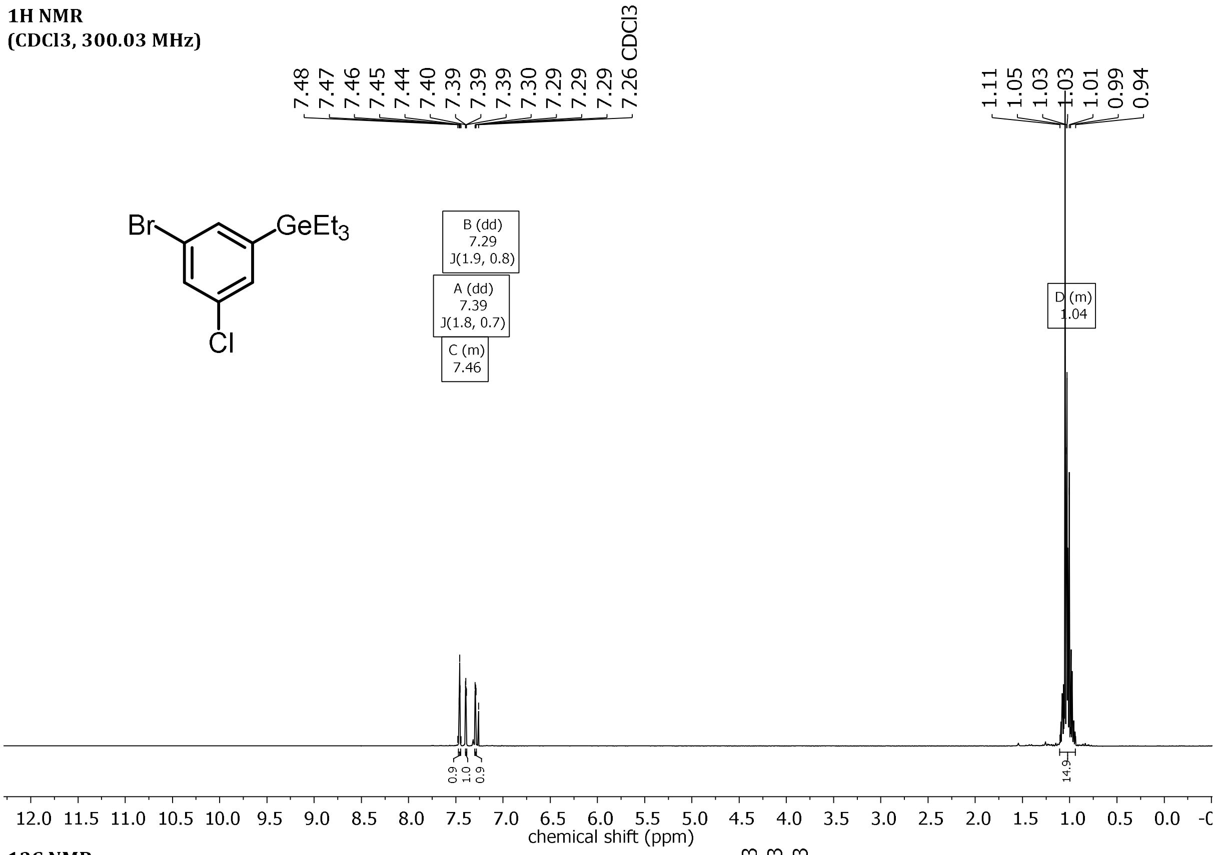

13C NMR
(CDCl3, 75.45 MHz)

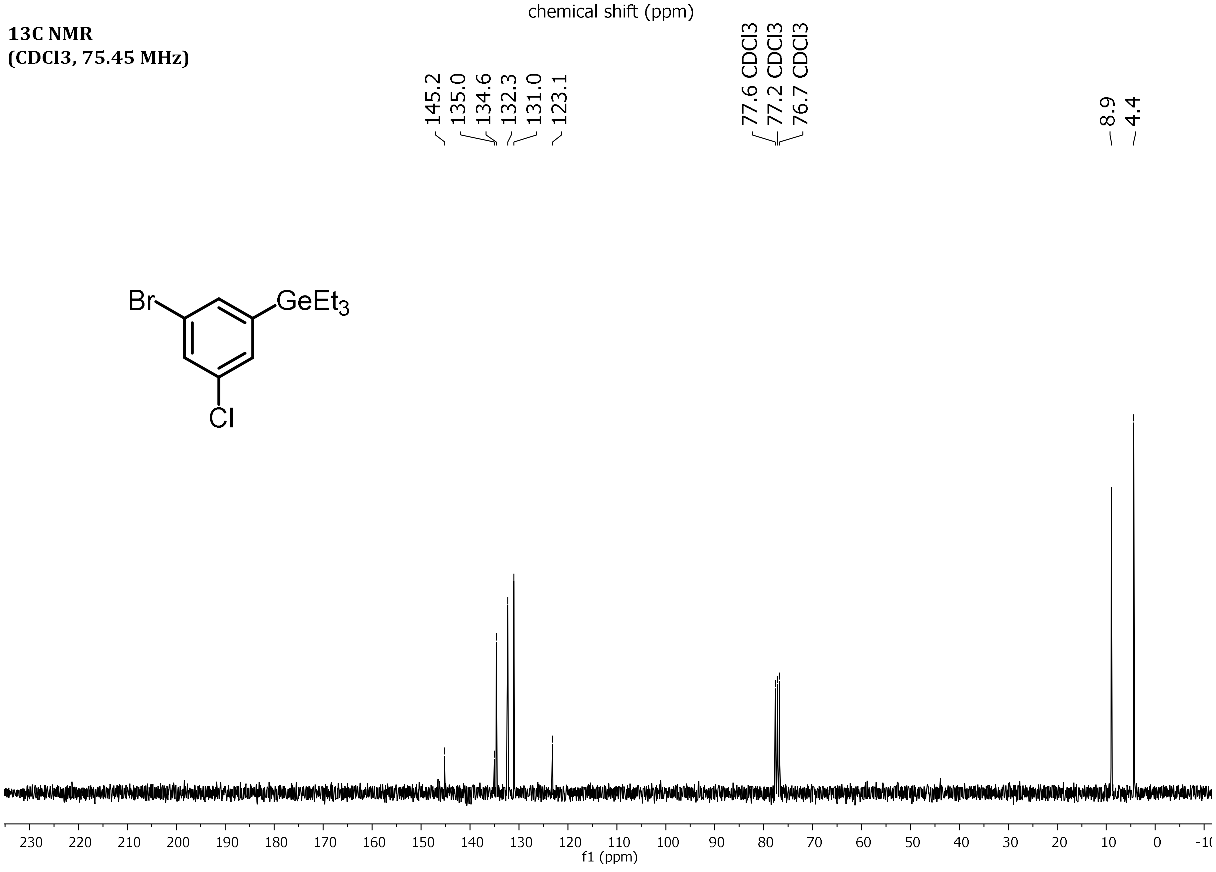

Benzo[*d*][1,3]dioxol-5-yltriethylgermane

1H NMR
(CDCl3, 399.97)

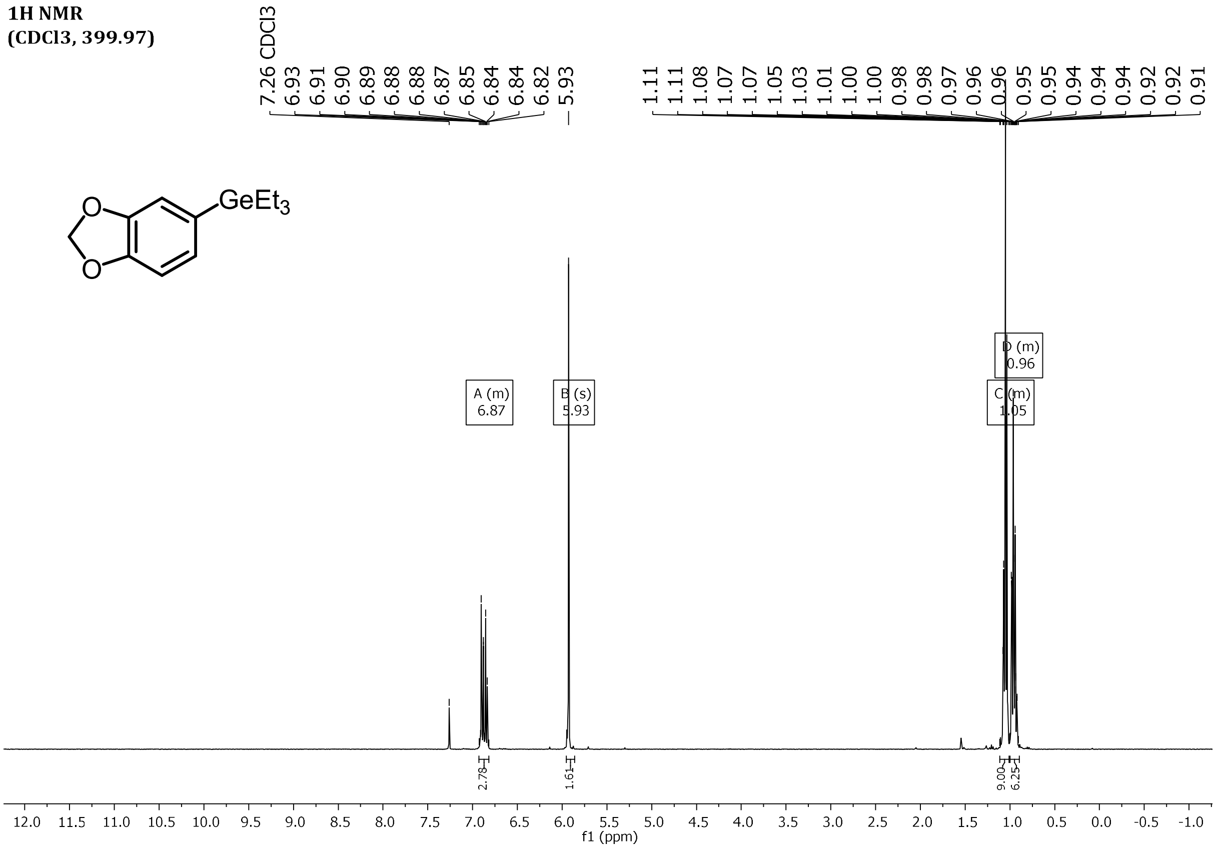

13C NMR
(CDCl3, 100.58 MHz)

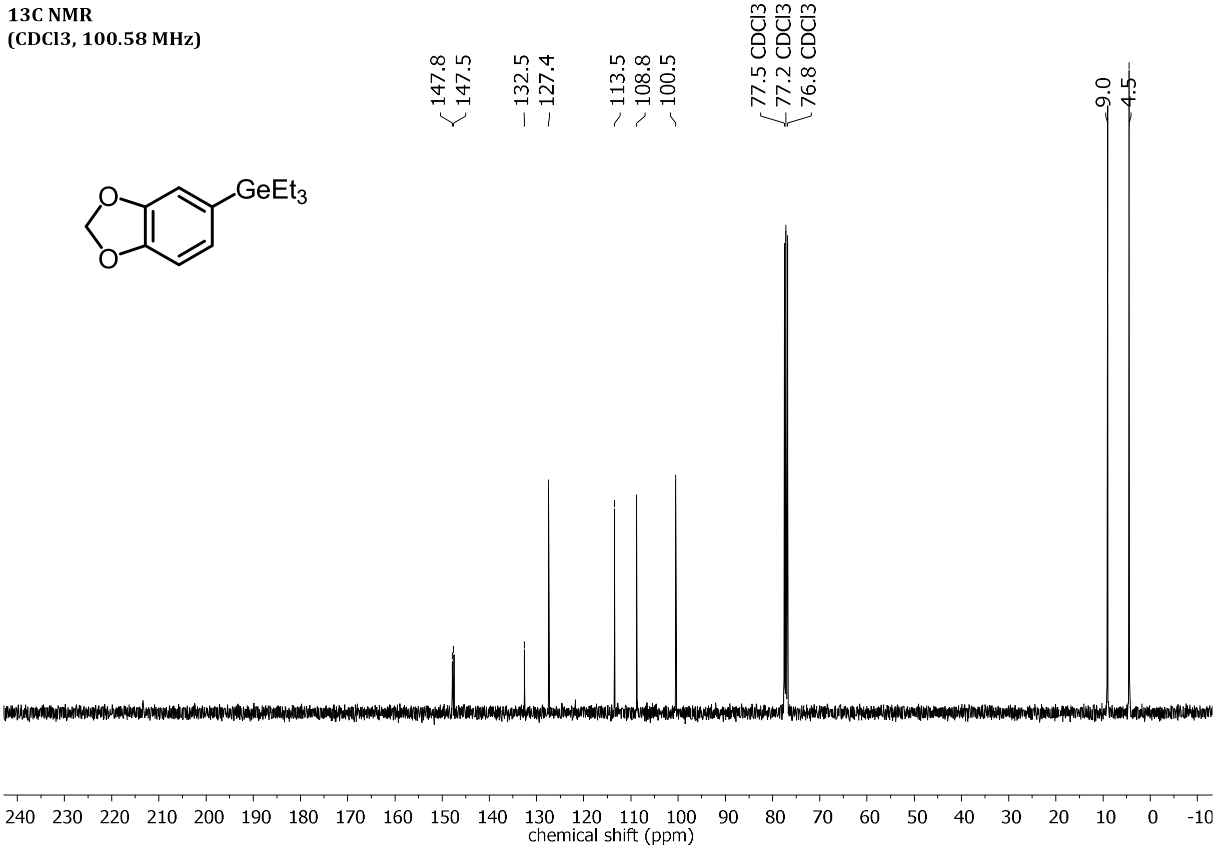

2-Methoxy-5-(triethylgermyl)benzonitrile

1H NMR
(CDCl3, 599.86)

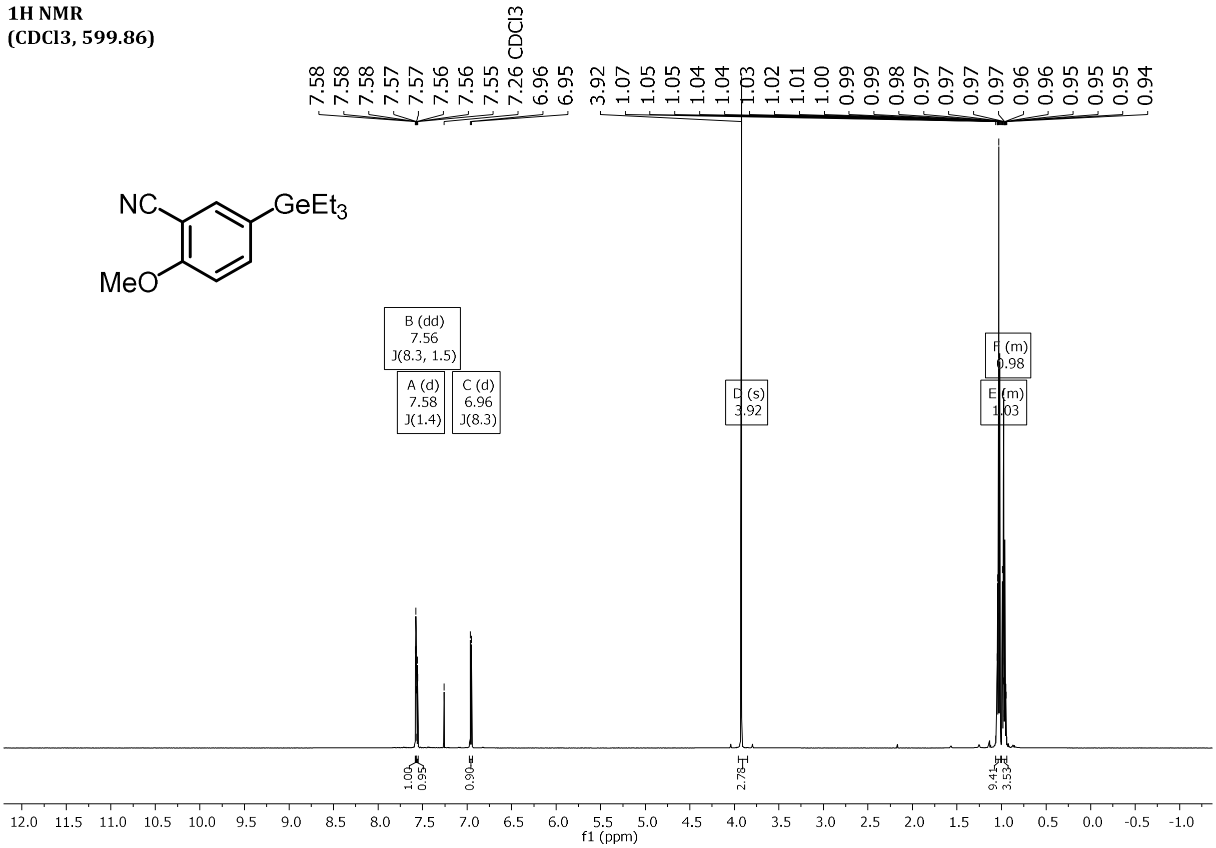

13C NMR
(CDCl3, 150.85 MHz)

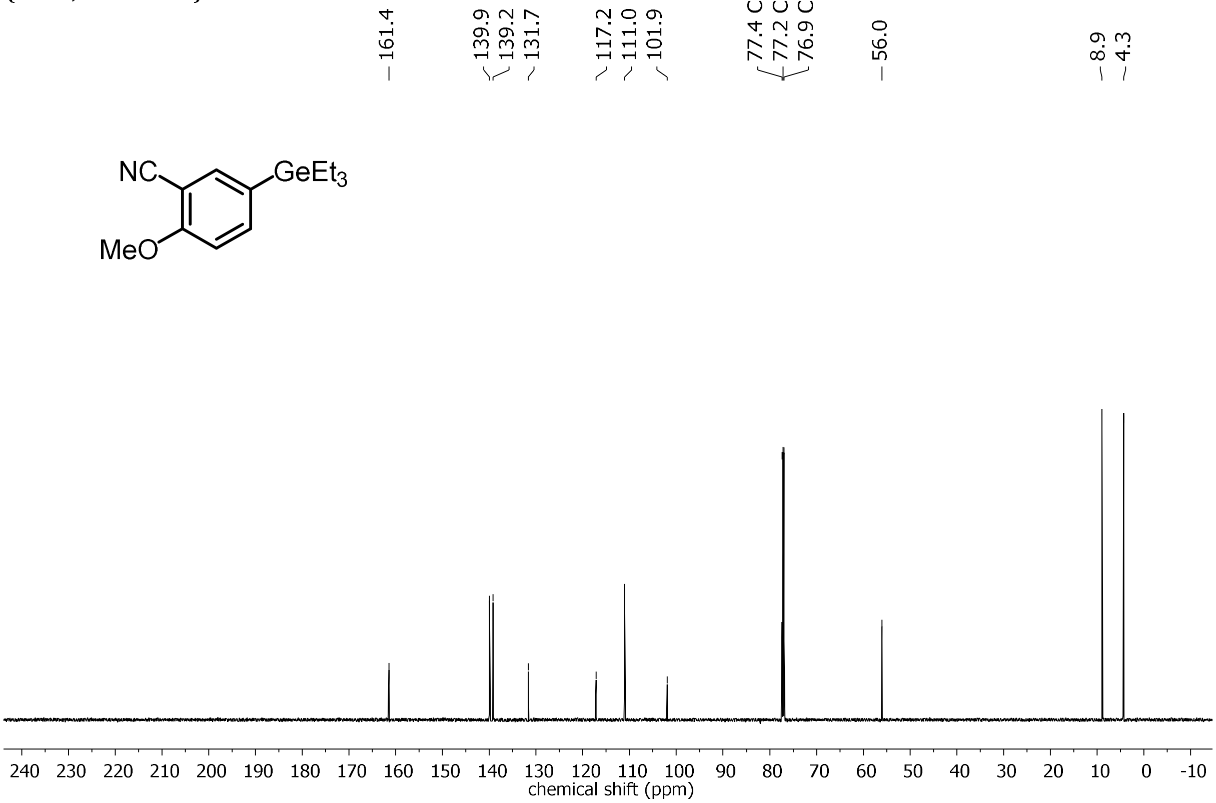

4-(Triethylgermyl)benzonitrile

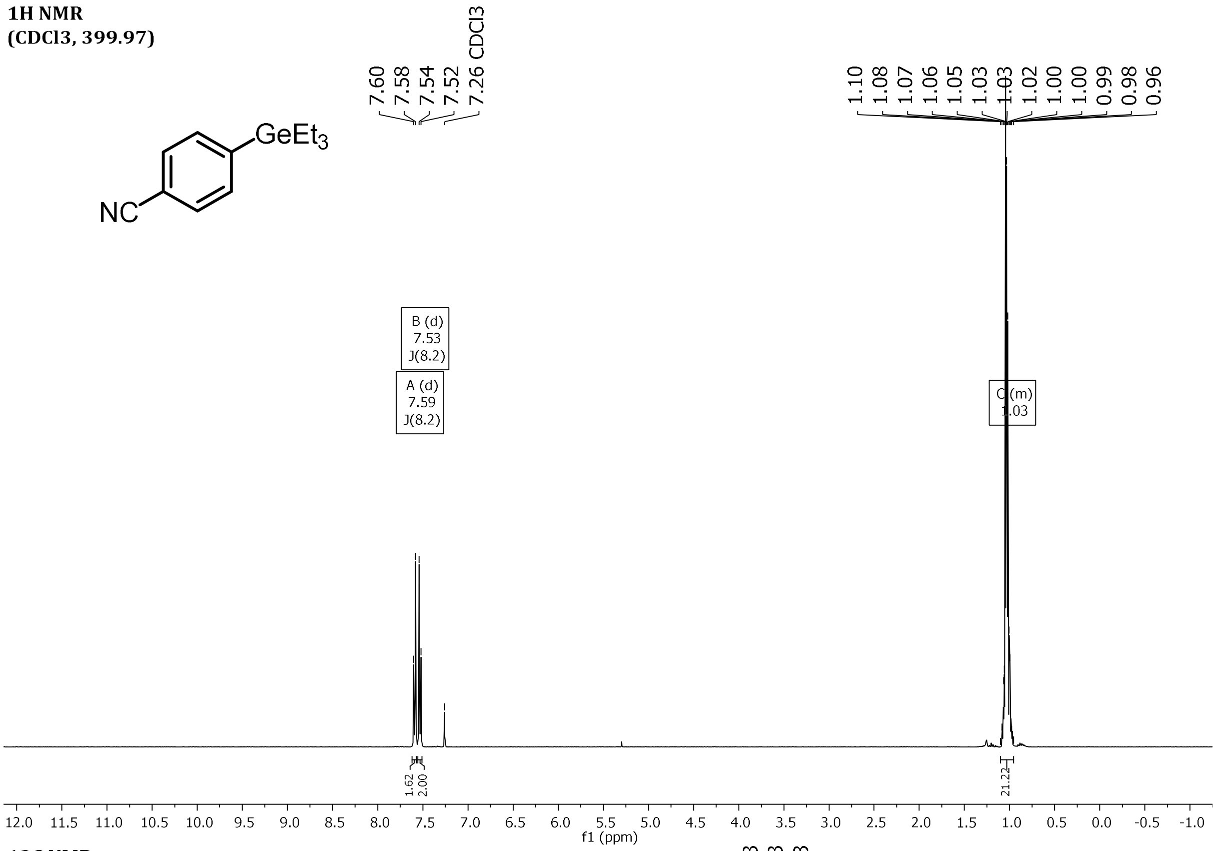

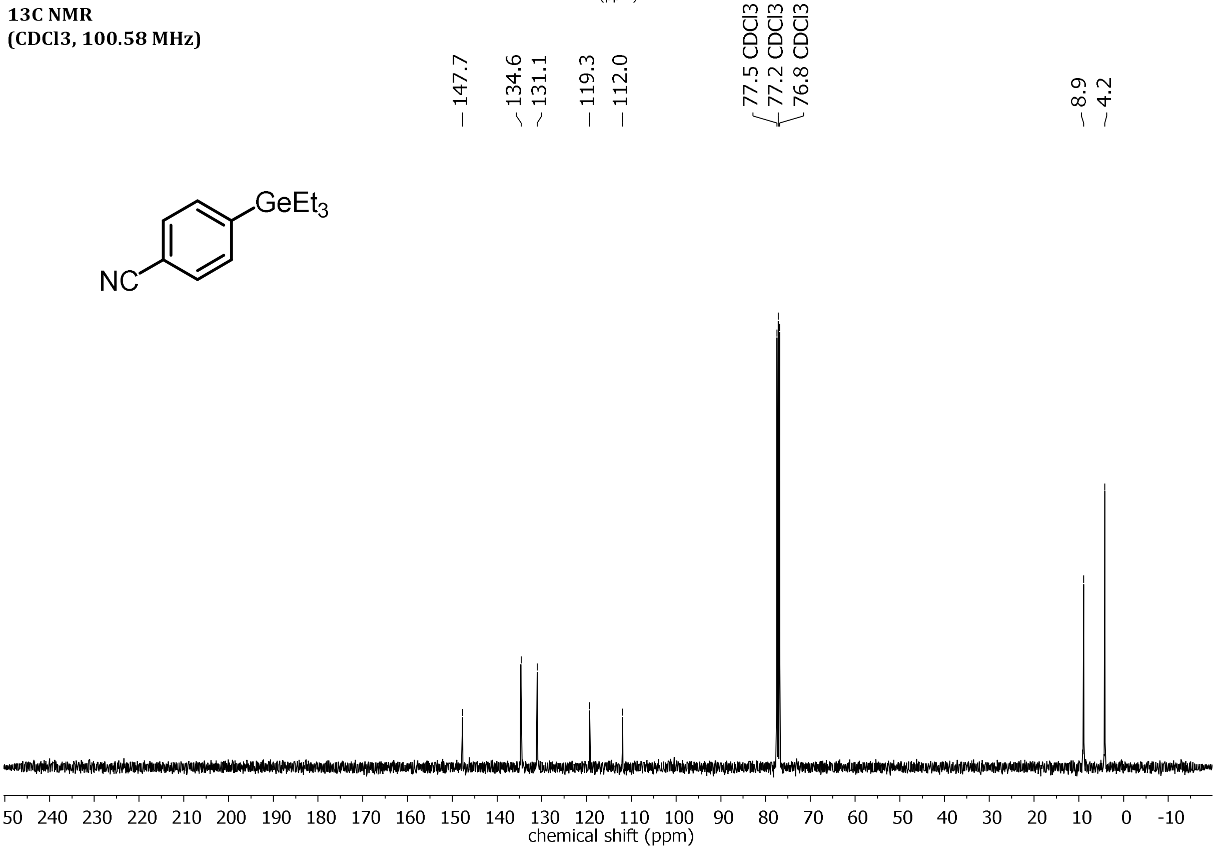

(4-Chloro-3-fluorophenyl)triethylgermane

1H NMR
(CDCl3, 600.44)

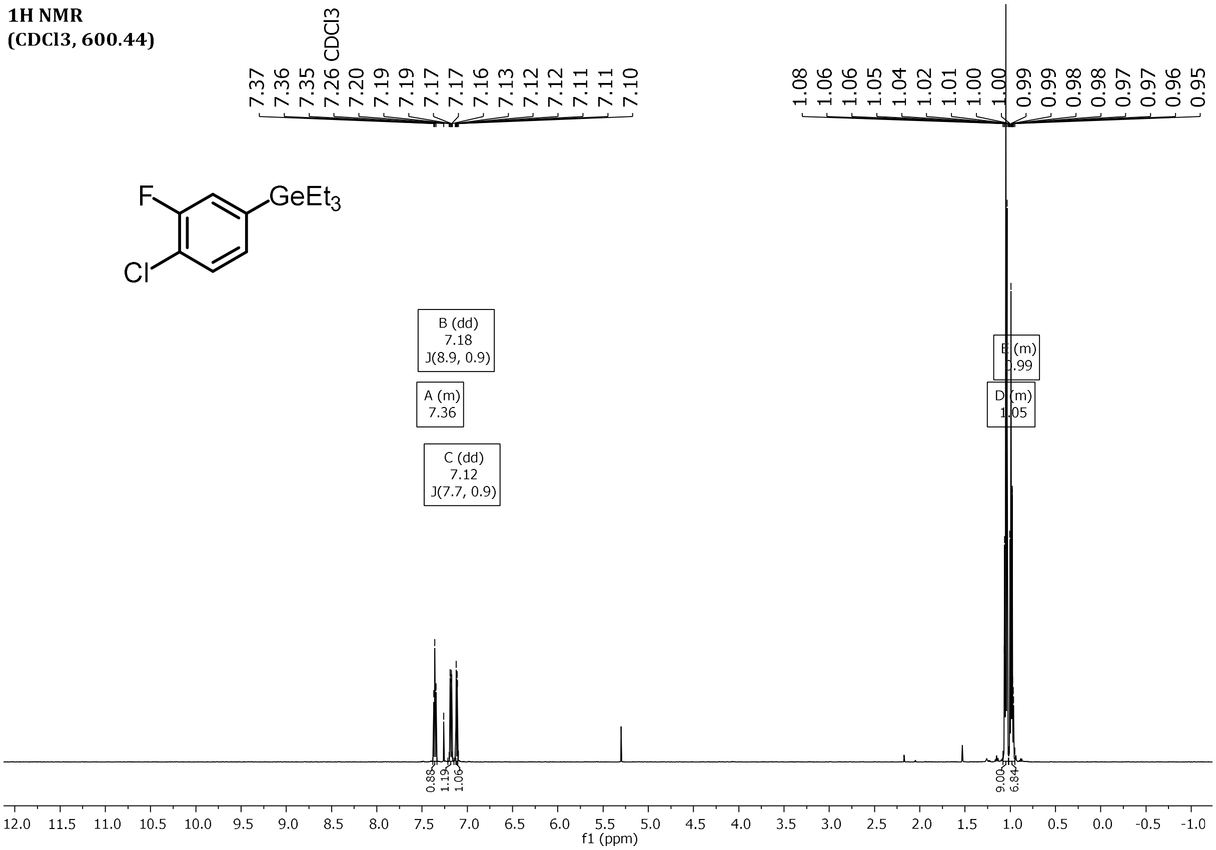

13C NMR
(CDCl3, 151.00 MHz)

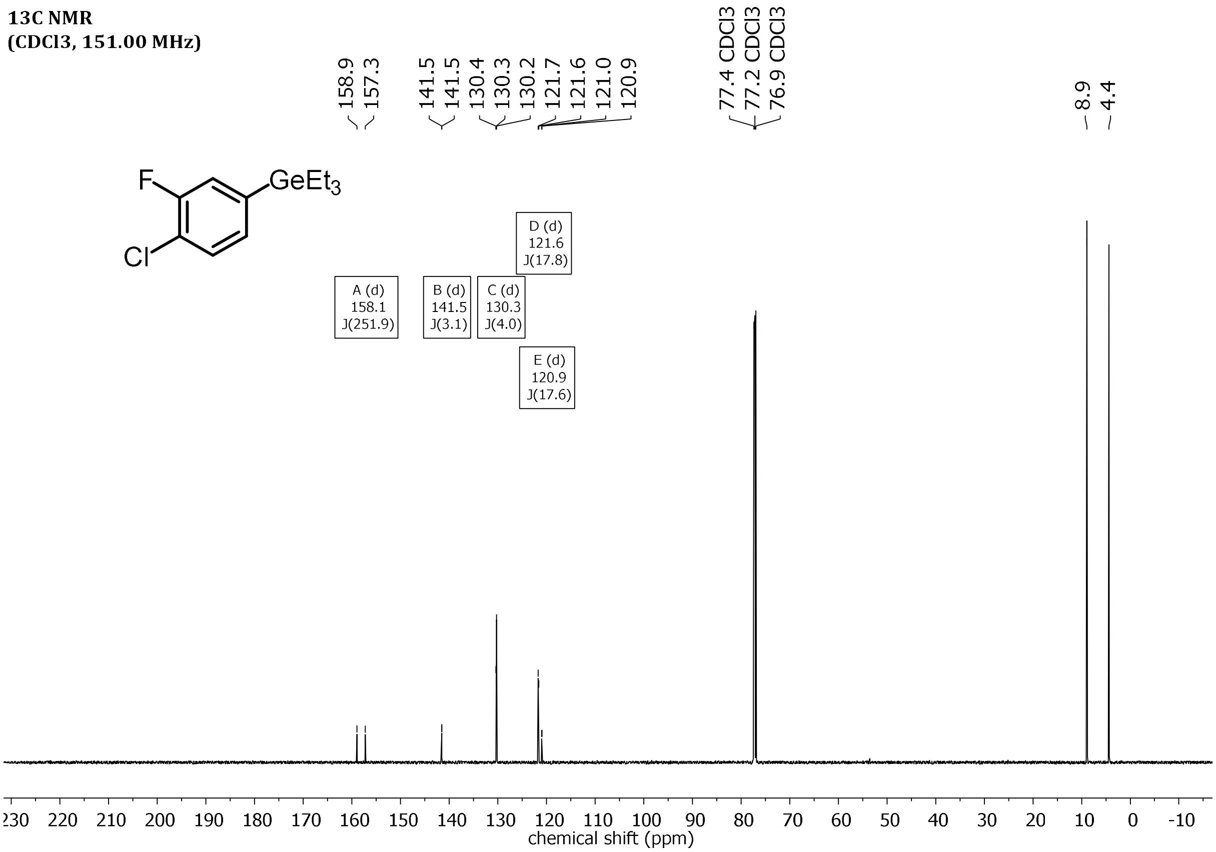

19F NMR
(CDCl3, 564.92)

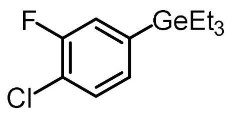

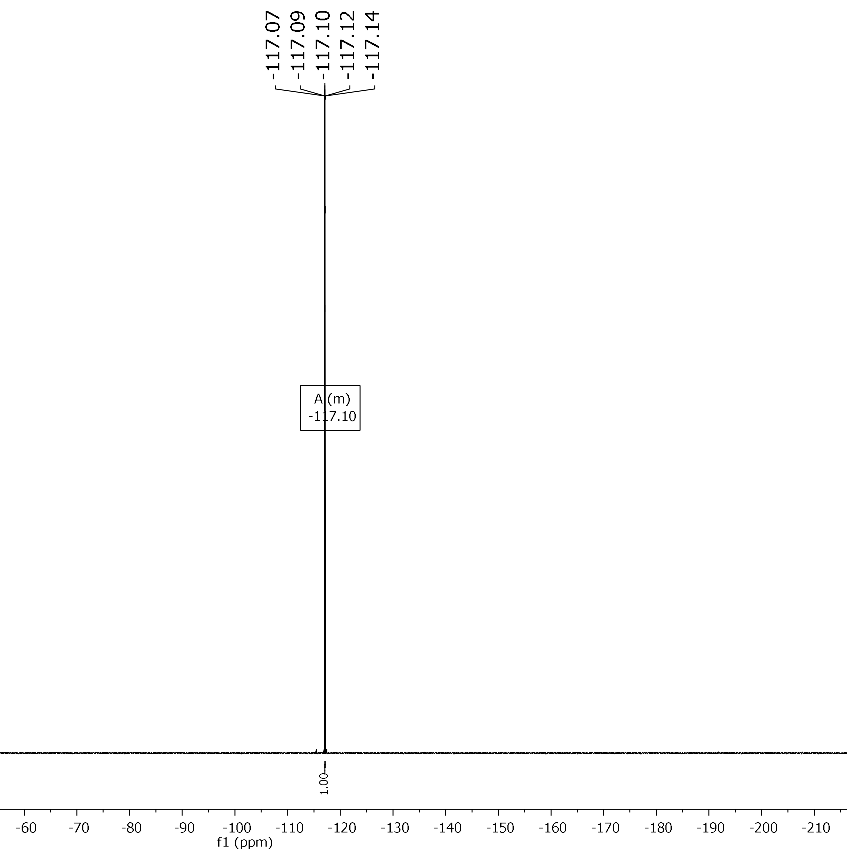

3,5-Dimethyl-4-(triethylgermyl)isoxazole

1H NMR
(CDCl3, 600.44 MHz)

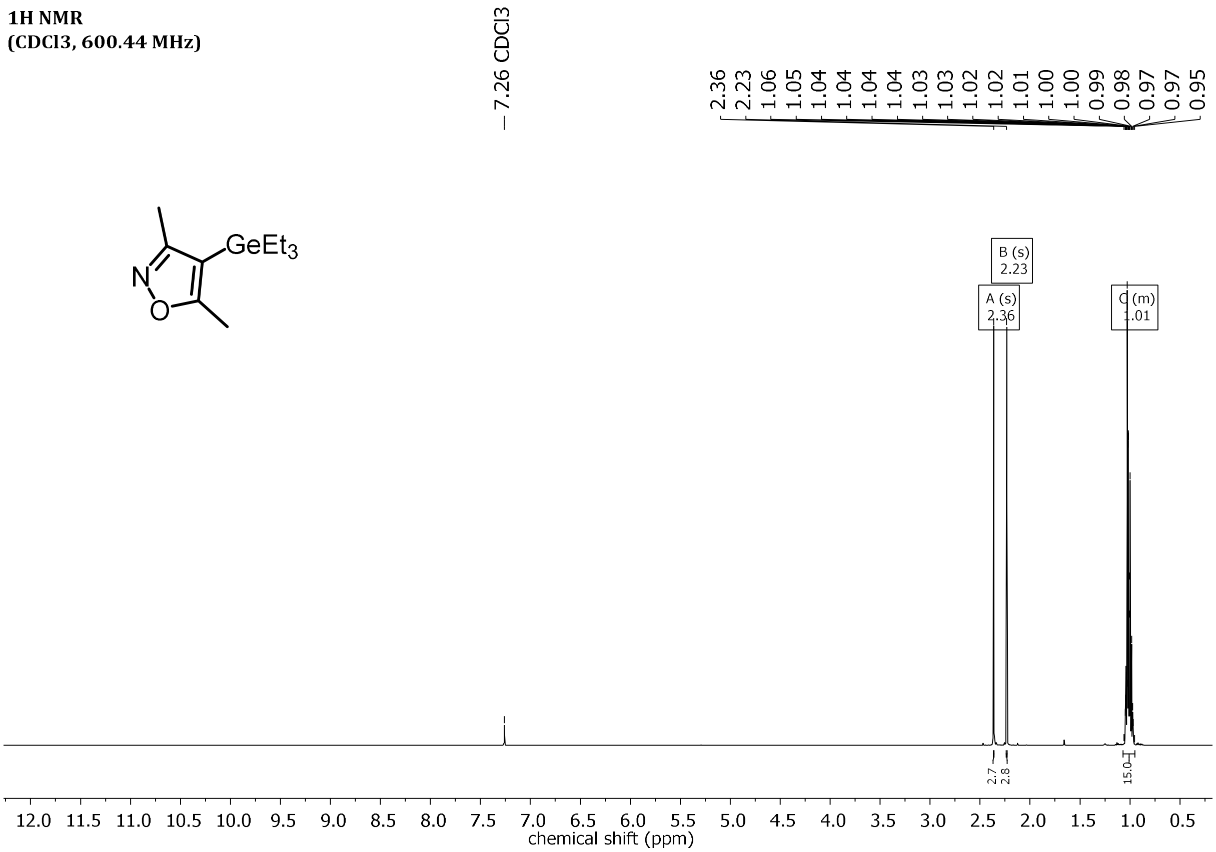

13C NMR
(CDCl3, 151.00 MHz)

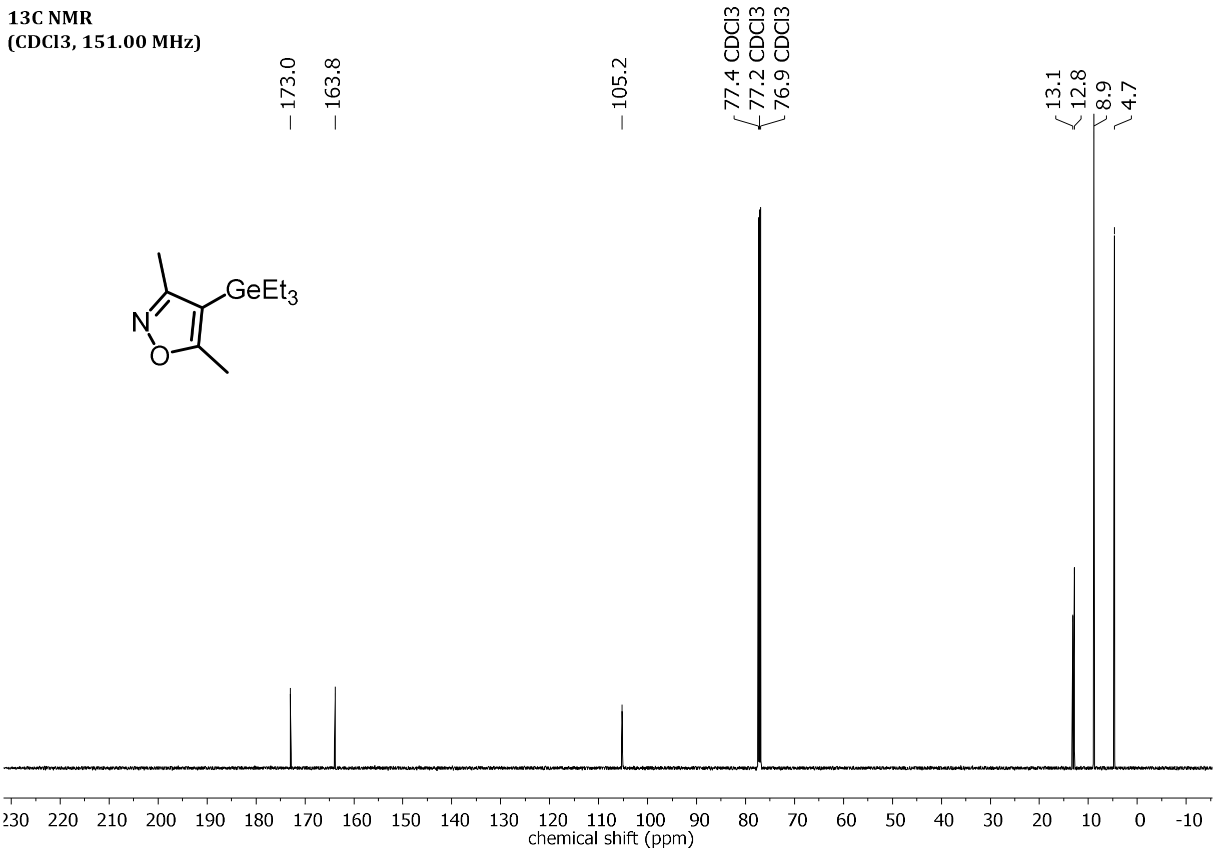

(3-Bromo-5-(triethylgermyl)phenyl)trimethylsilane

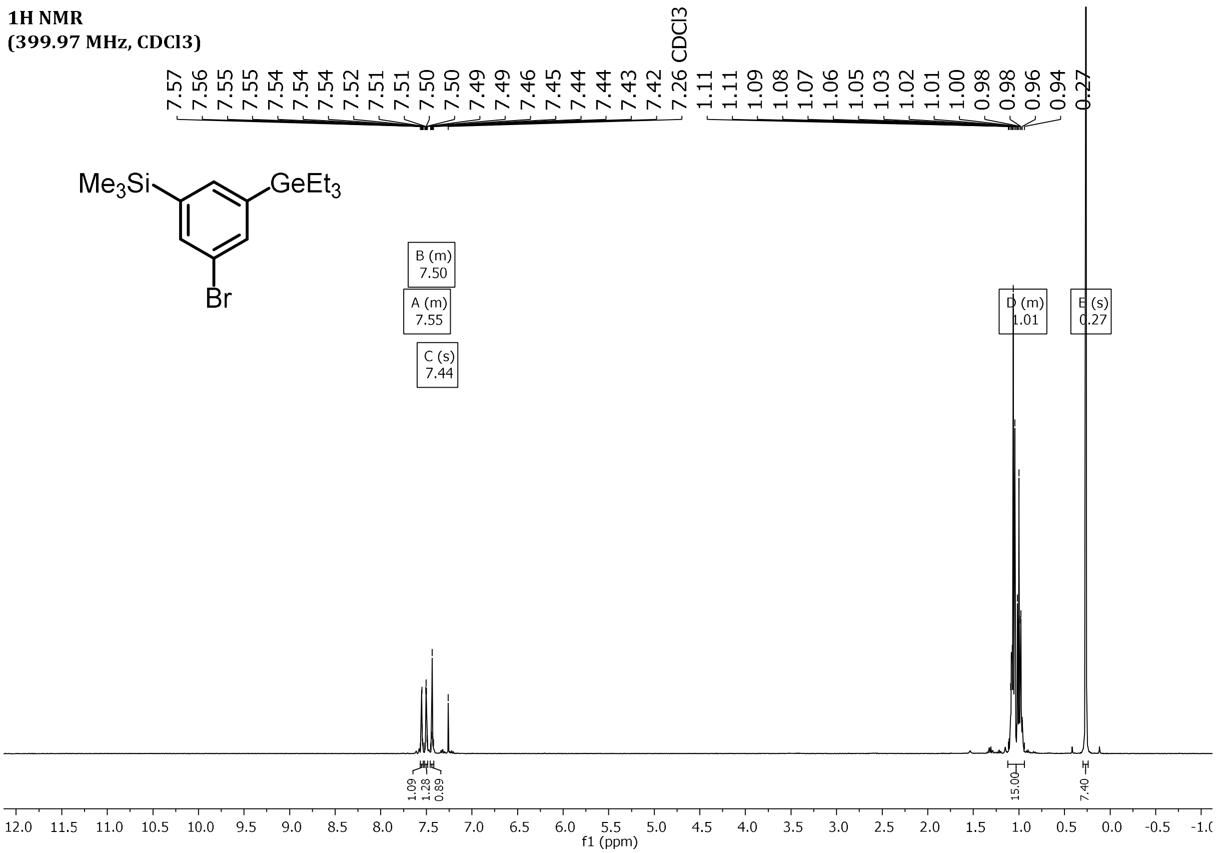

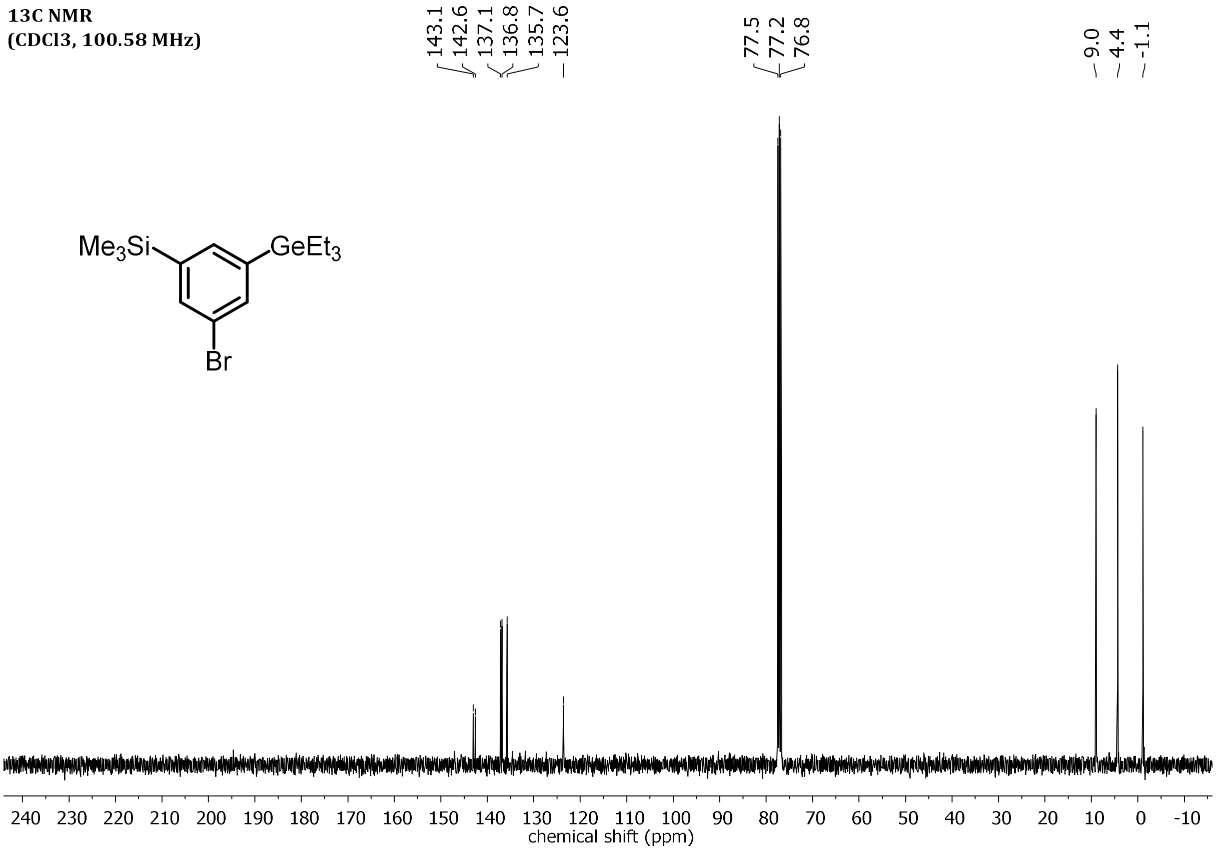

Triethyl(*o*-tolyl)germane

1H NMR
(CDCl3, 599.86 MHz)

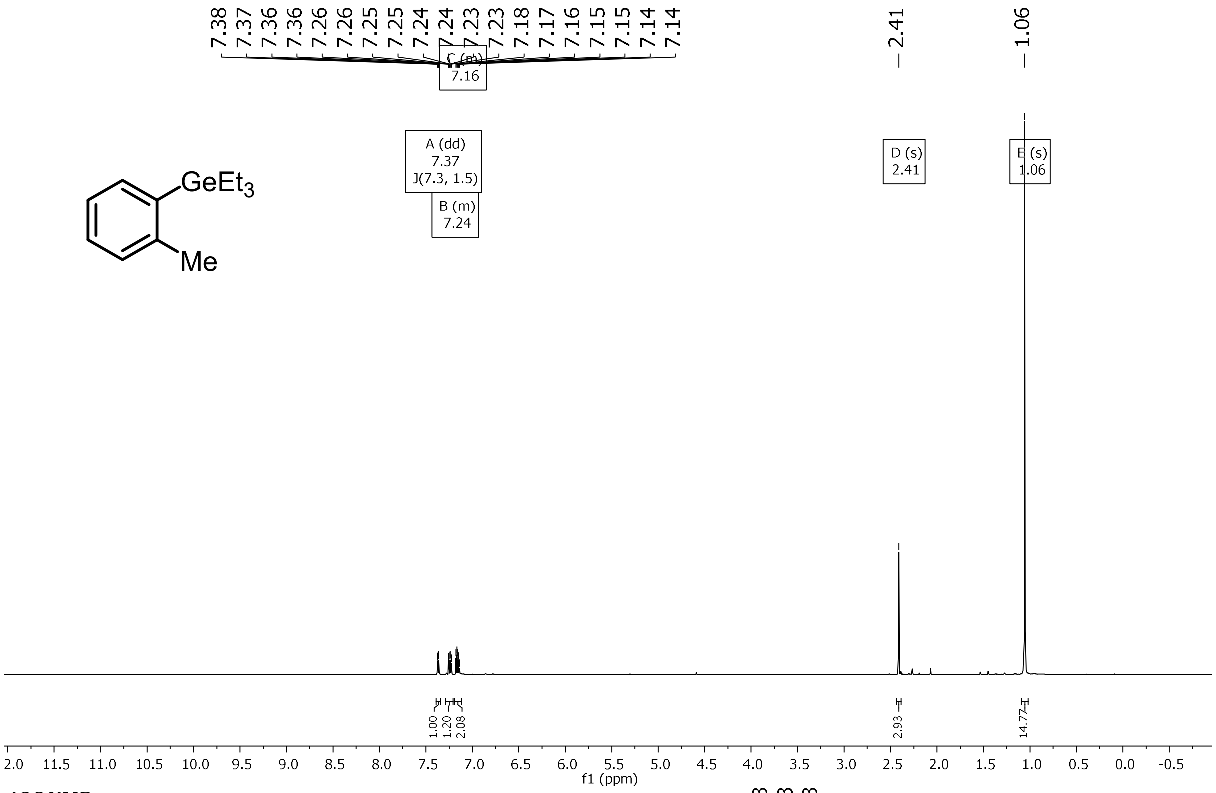

13C NMR
(CDCl3, 150.85 MHz)

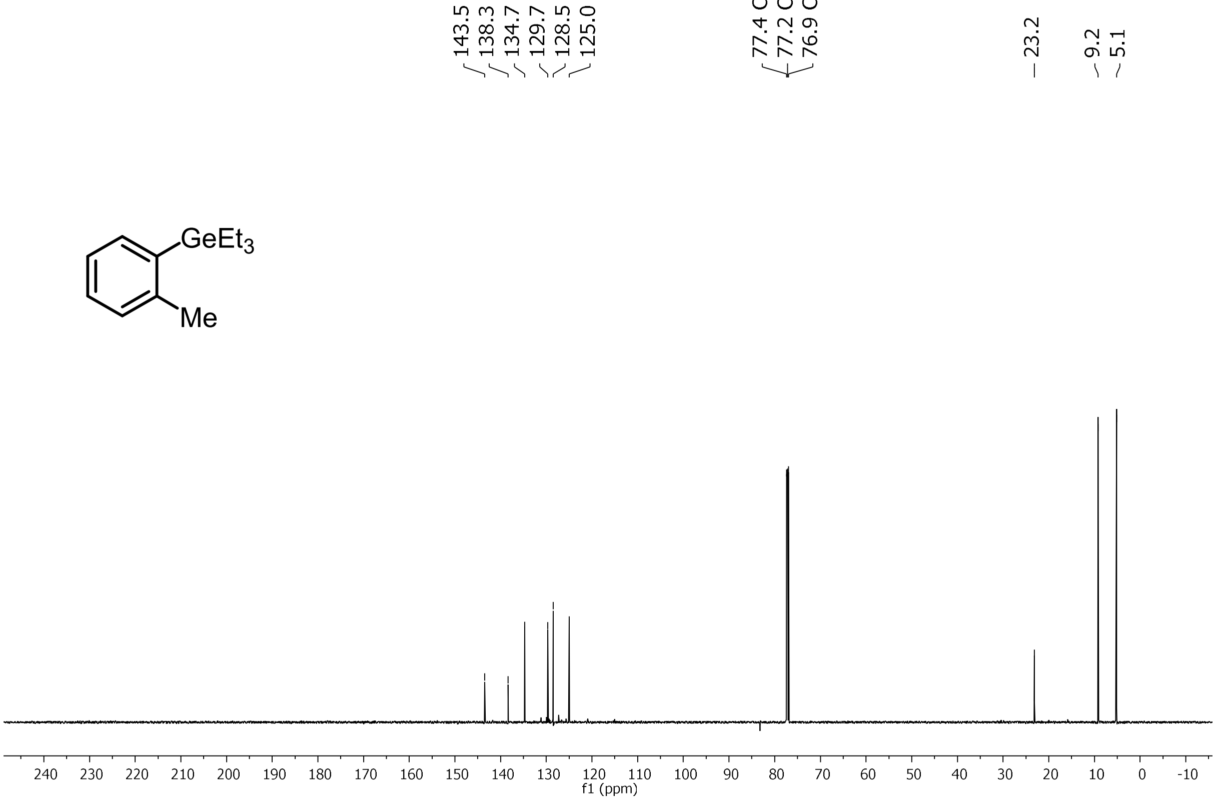

(2-(Allyloxy)phenyl)triethylgermane

1H NMR
(CDCl3, 600.44 MHz)

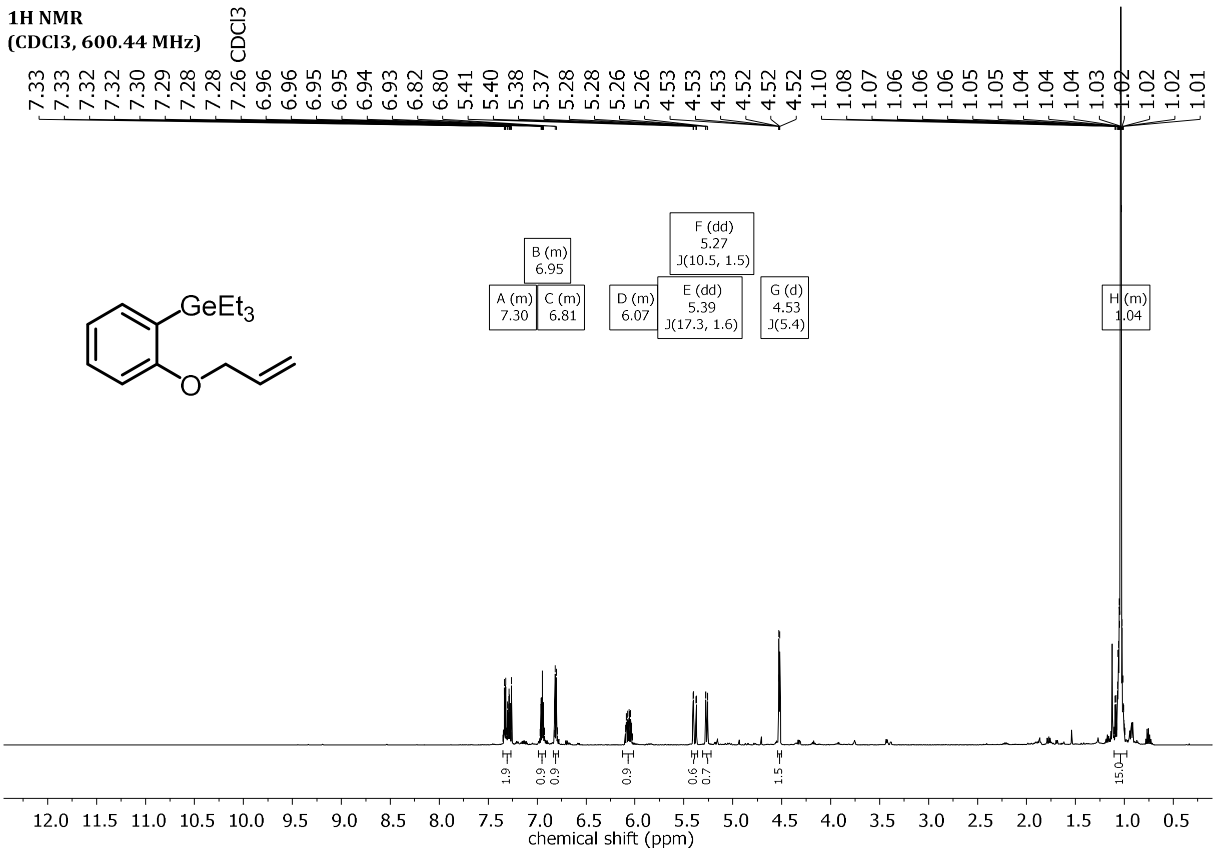

13C NMR
(CDCl3, 151.00 MHz)

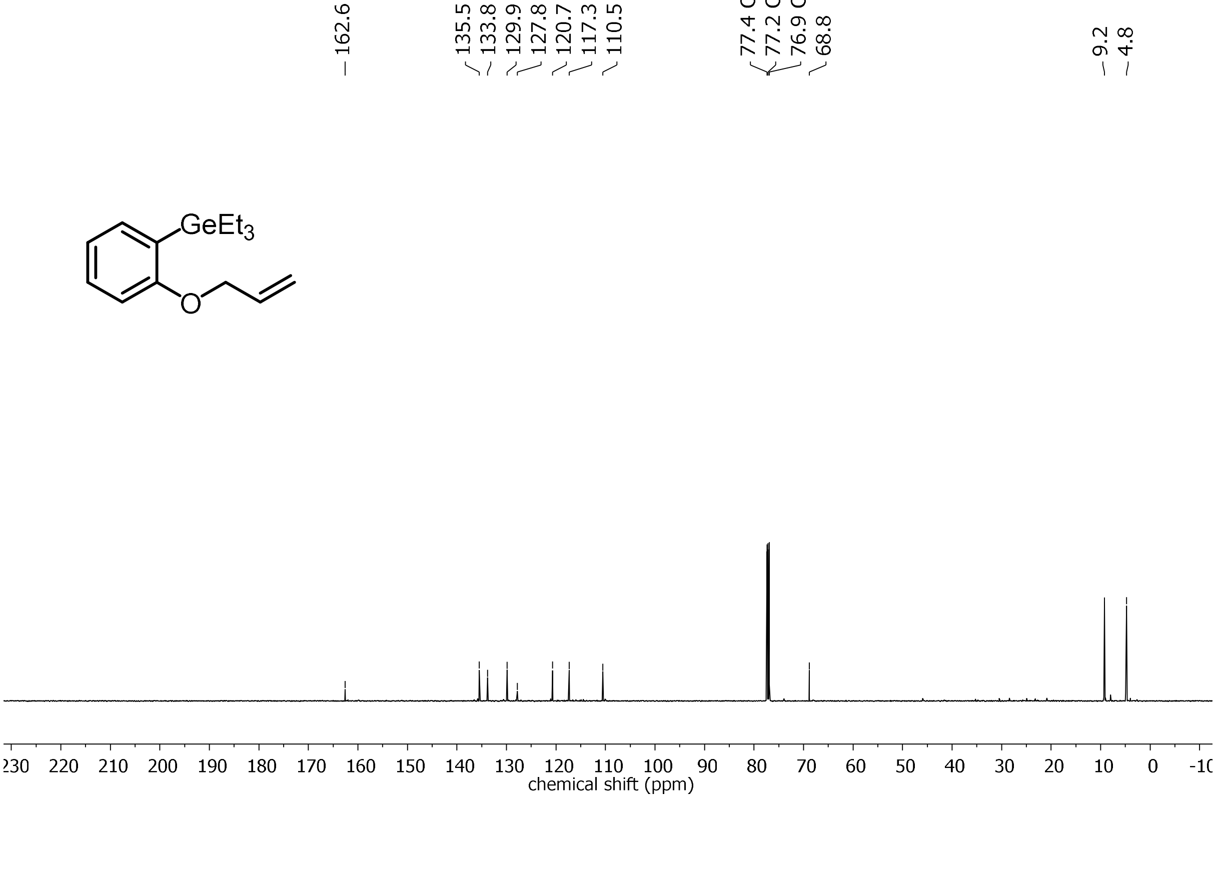

Triethyl(4-(triethylgermyl)phenyl)silane

1H NMR
(599.86 MHz, CDCl3)

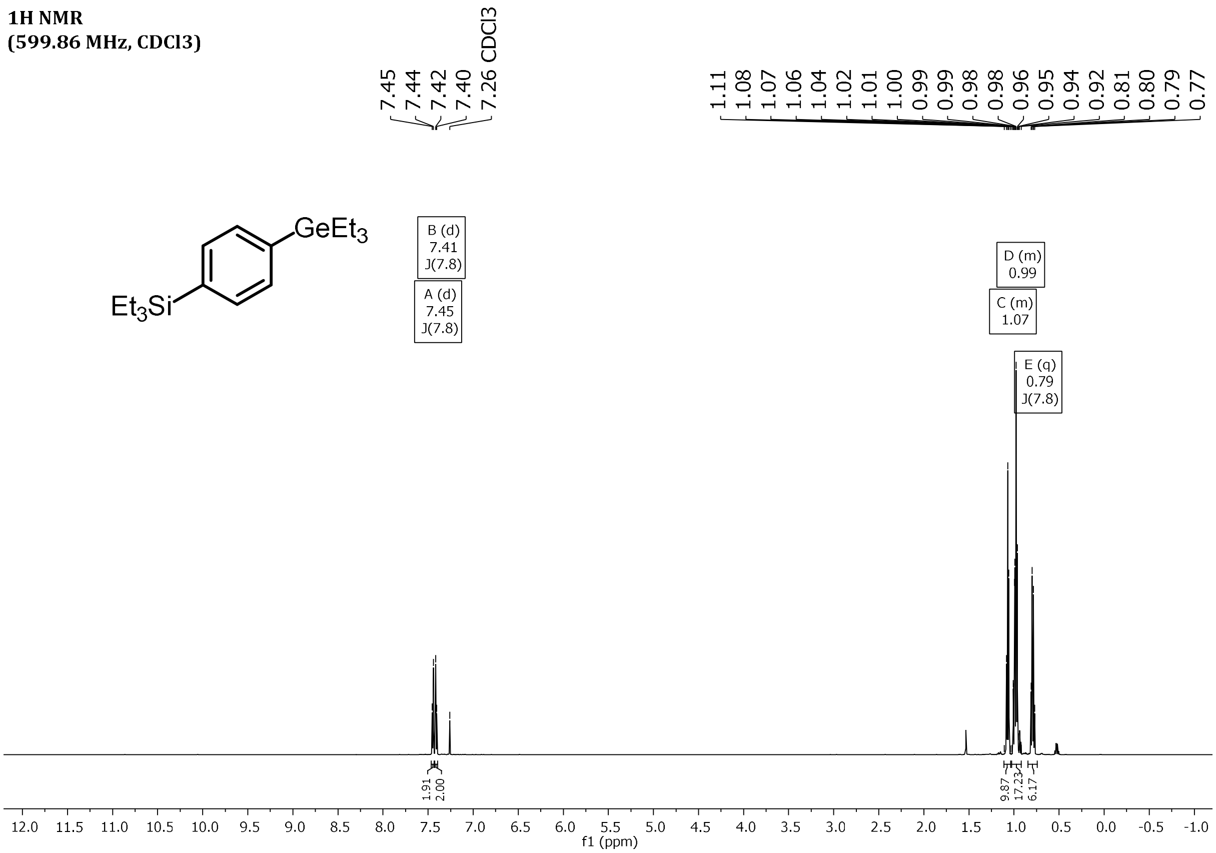

13C NMR
(CDCl3, 150.85 MHz)

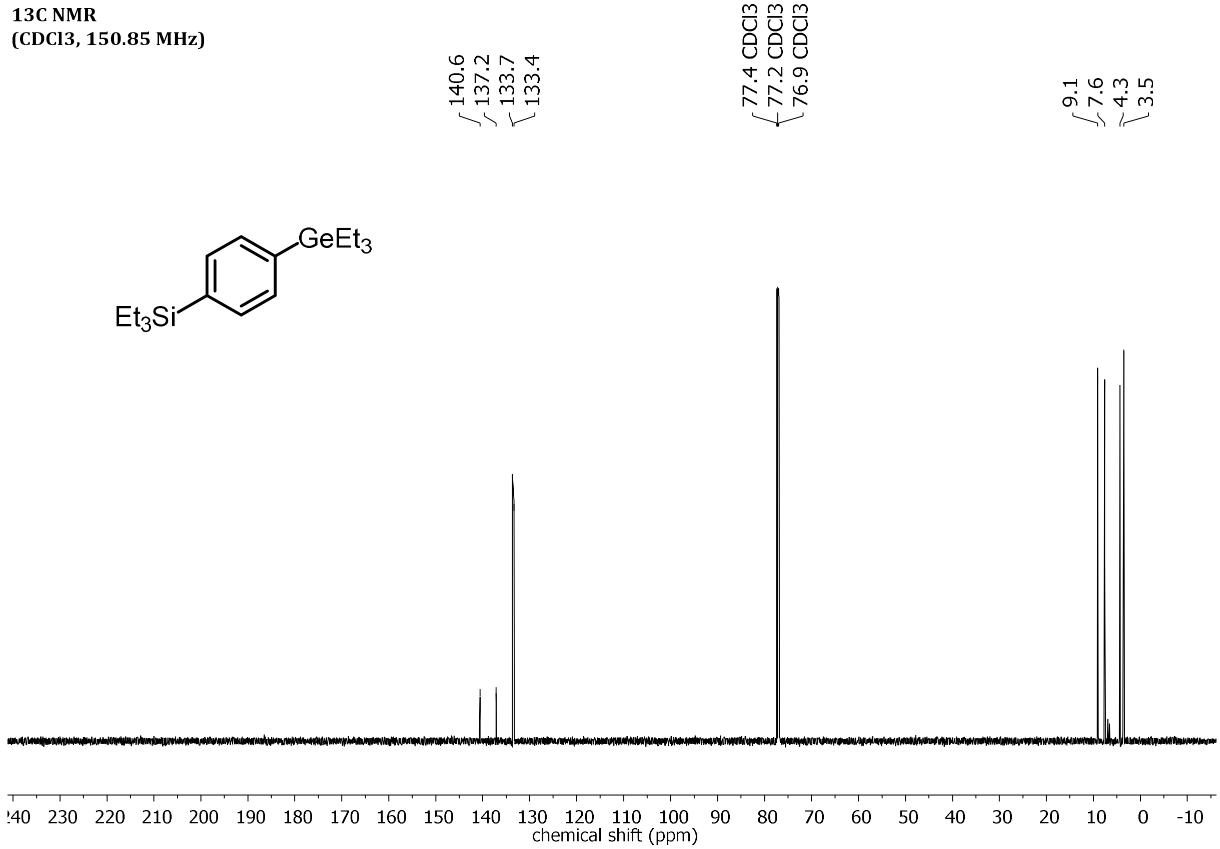

(3-Iodo-5-(triethylgermyl)phenyl)trimethylsilane

1H NMR
(CDCl3, 600.44 MHz)

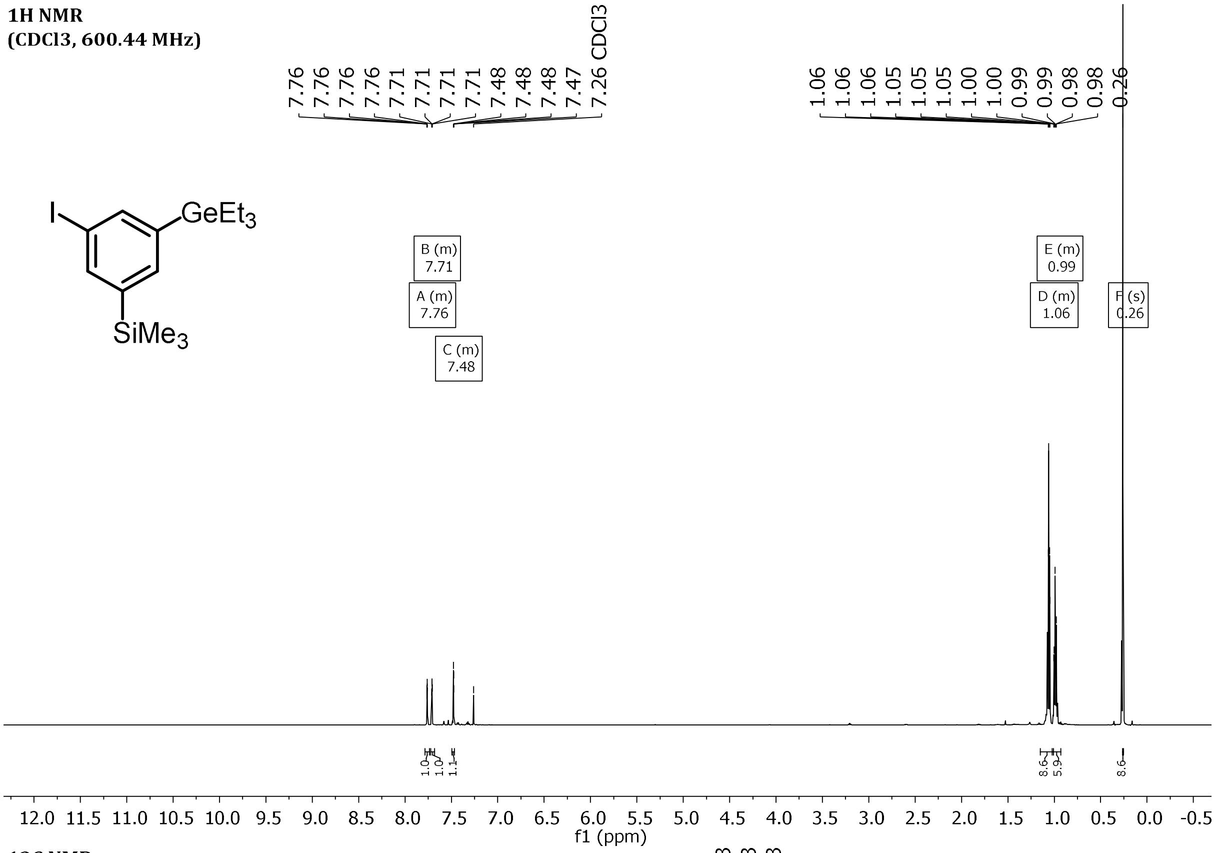

13C NMR
(CDCl3, 151.00 MHz)

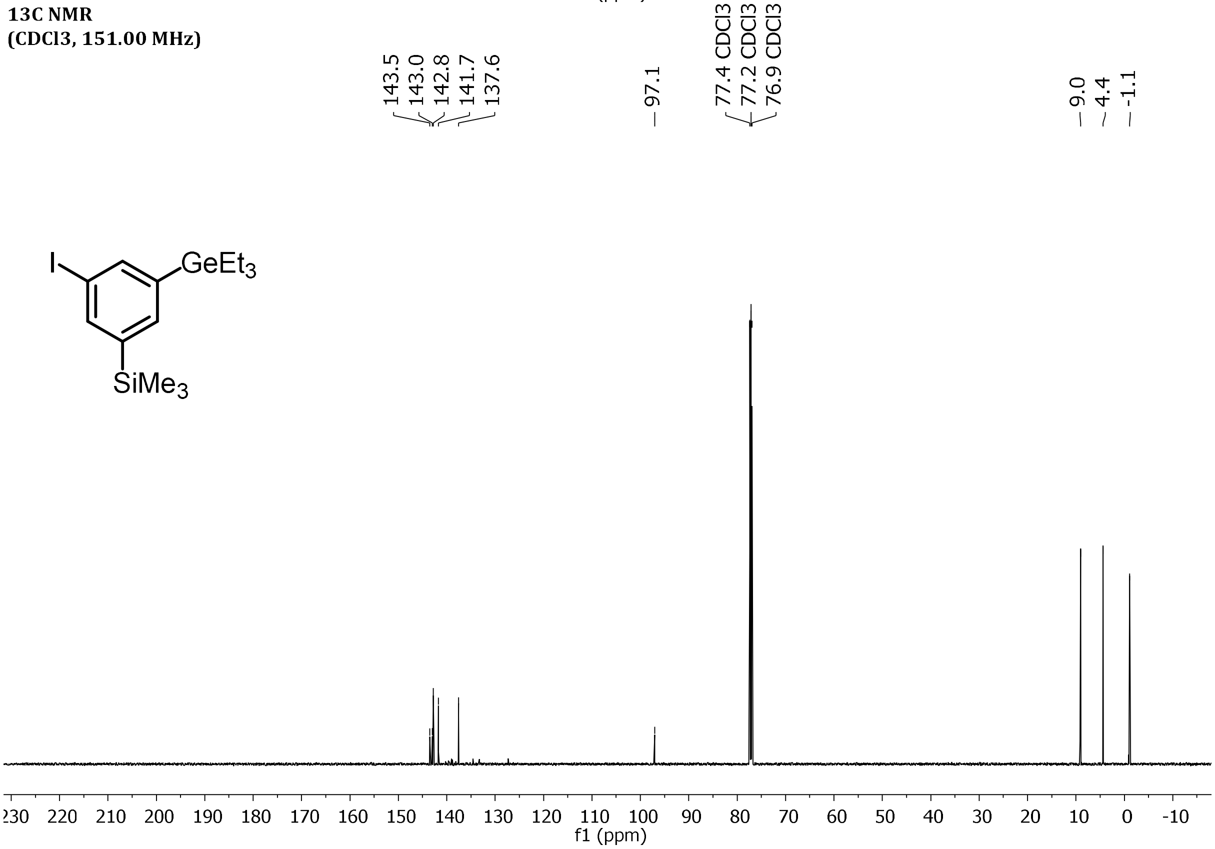

Trimethyl(3-(4,4,5,5-tetramethyl-1,3,2-dioxaborolan-2-yl)-5-(triethylgermyl)phenyl)silane

1H NMR
(599.86 MHz, CDCl3)

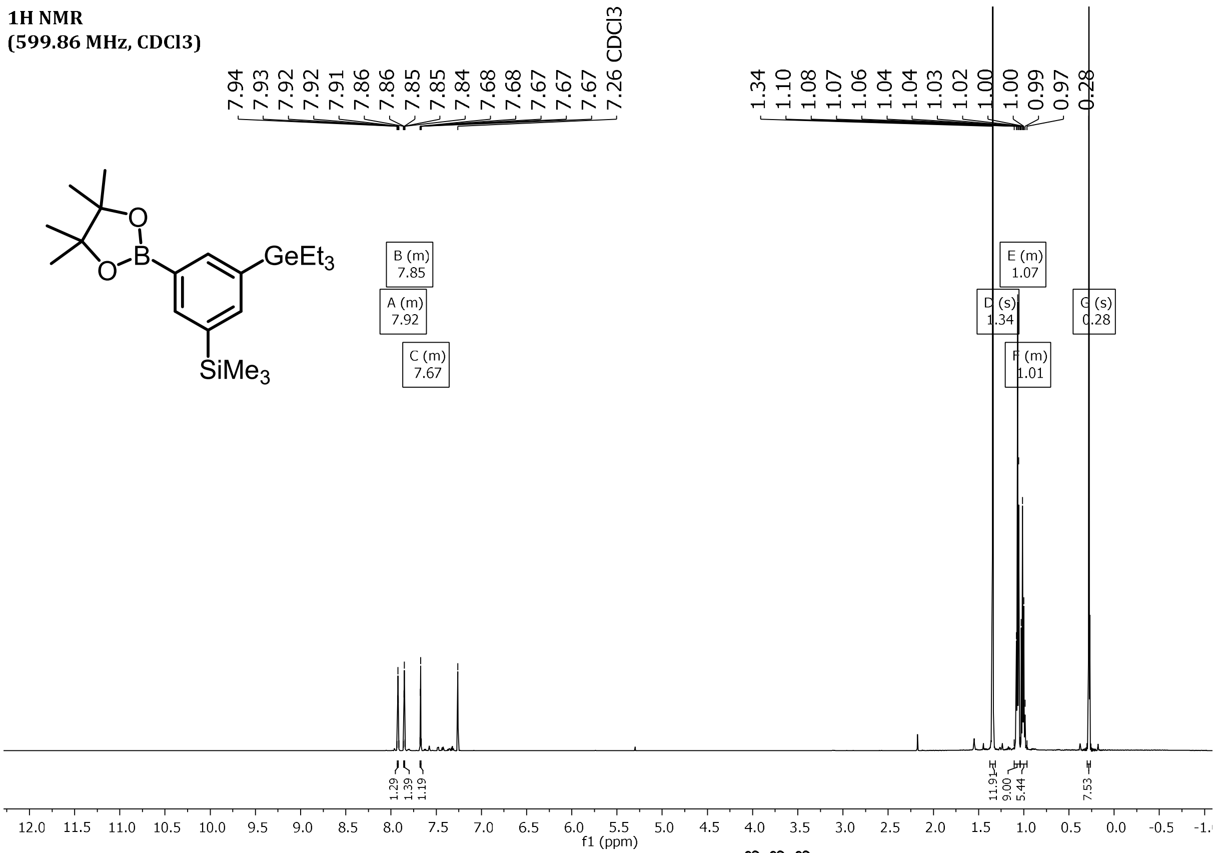

13C NMR
(CDCl3, 150.85 MHz)

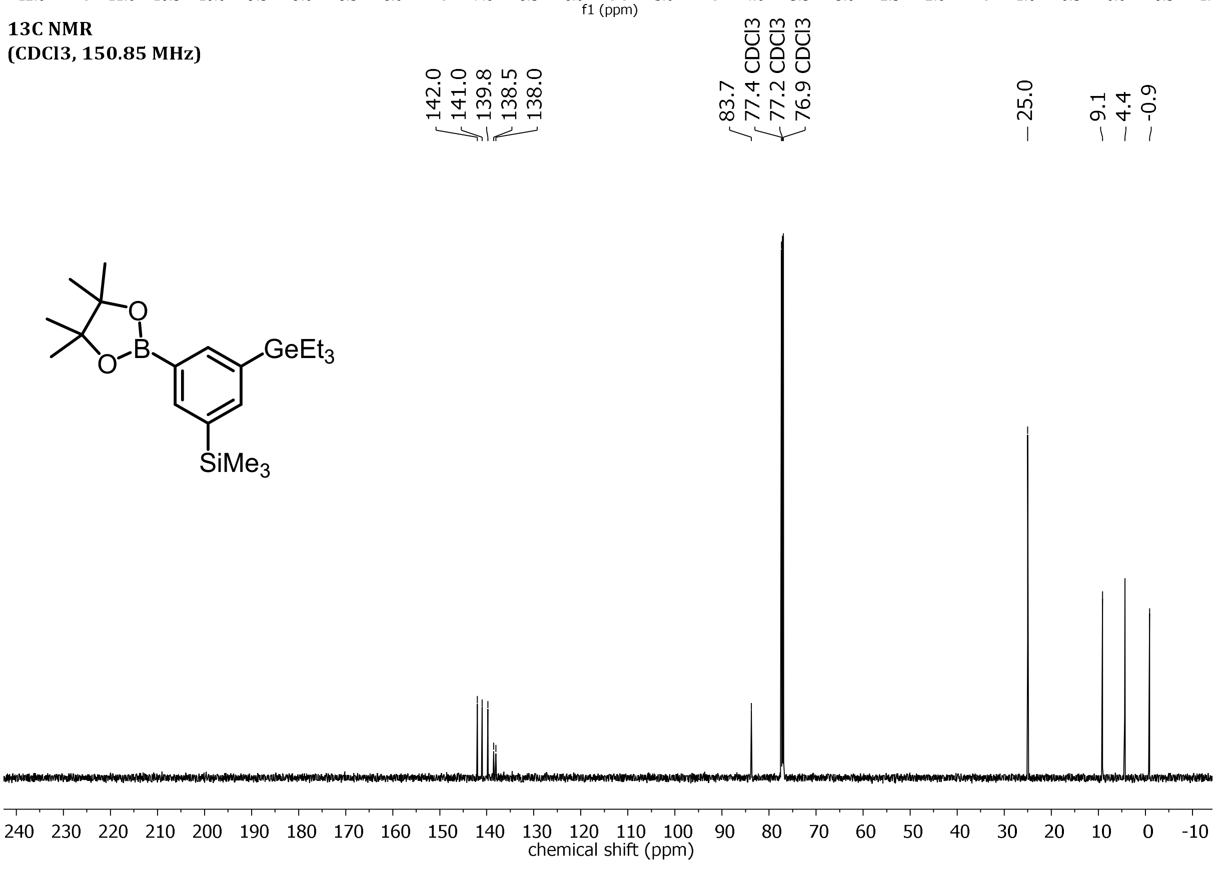

Ethyl 4'-acetoxy-[1,1'-biphenyl]-4-carboxylate

1H NMR
(CDCl3, 600.44 MHz)

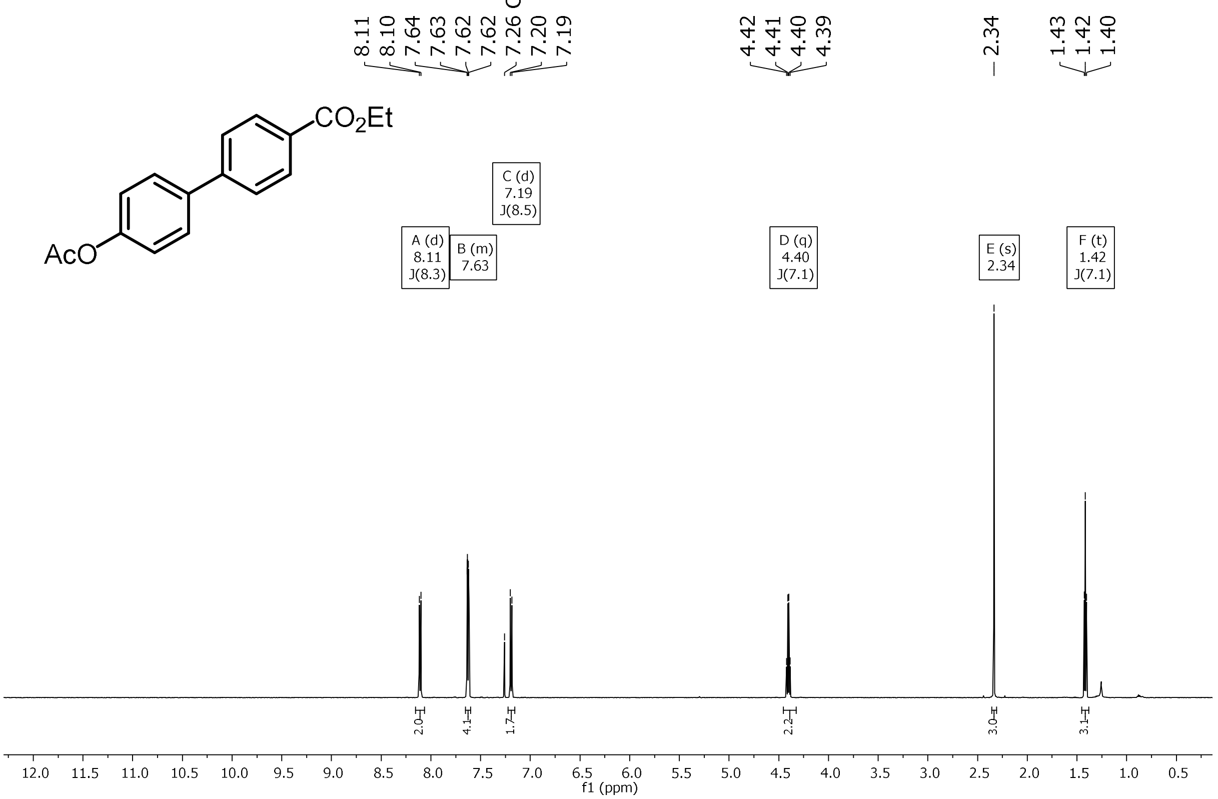

13C NMR
(CDCl3, 151.00 MHz)

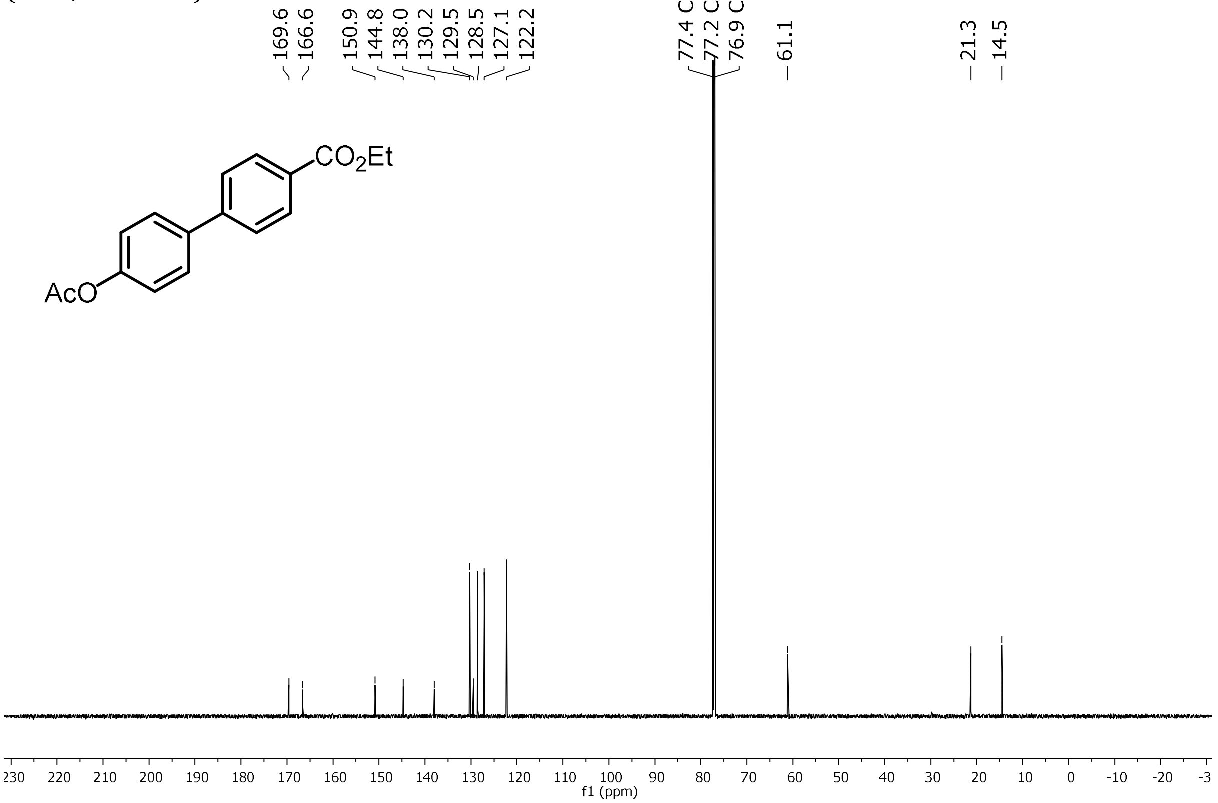

(5-*n*-Butyl-4'-nitro-[1,1'-biphenyl]-3-yl)trimethylsilane

1H NMR
(CDCl3, 600.44 MHz)

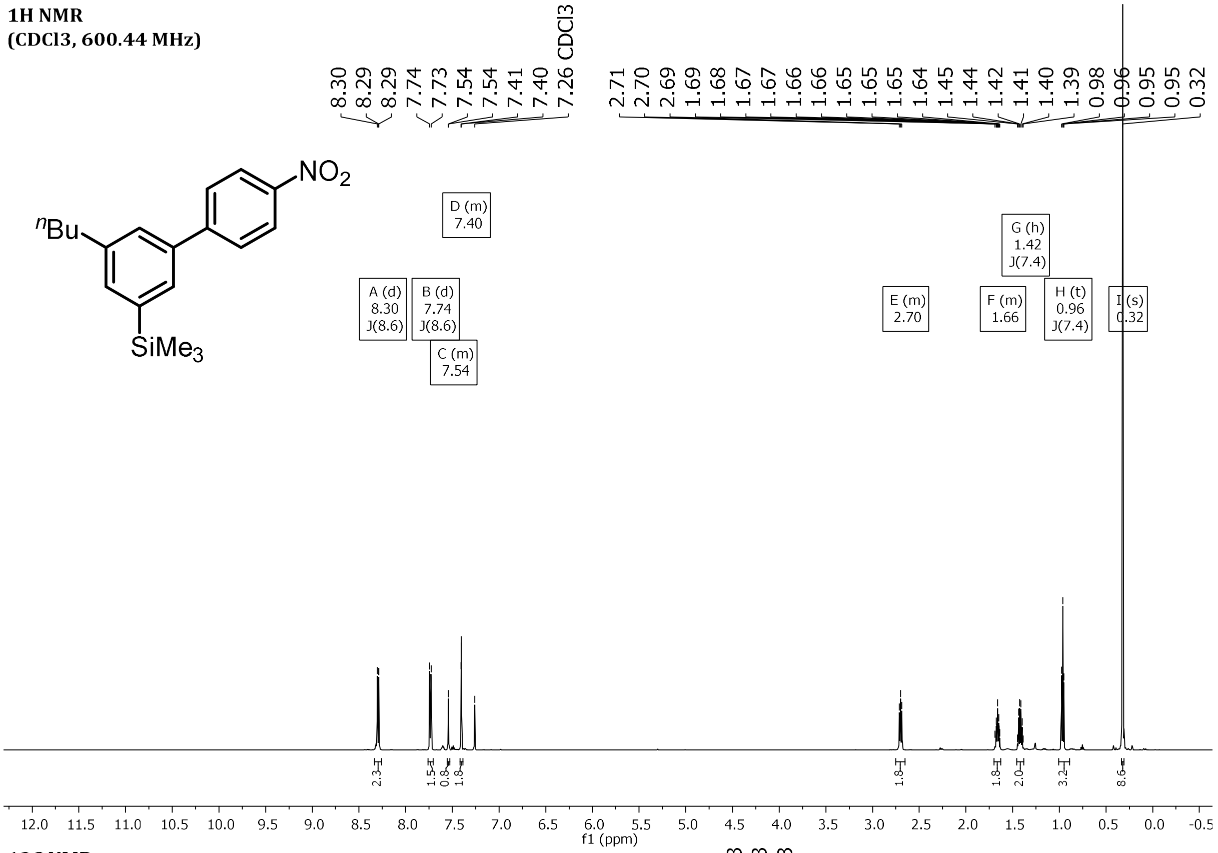

13C NMR
(CDCl3, 151.00 MHz)

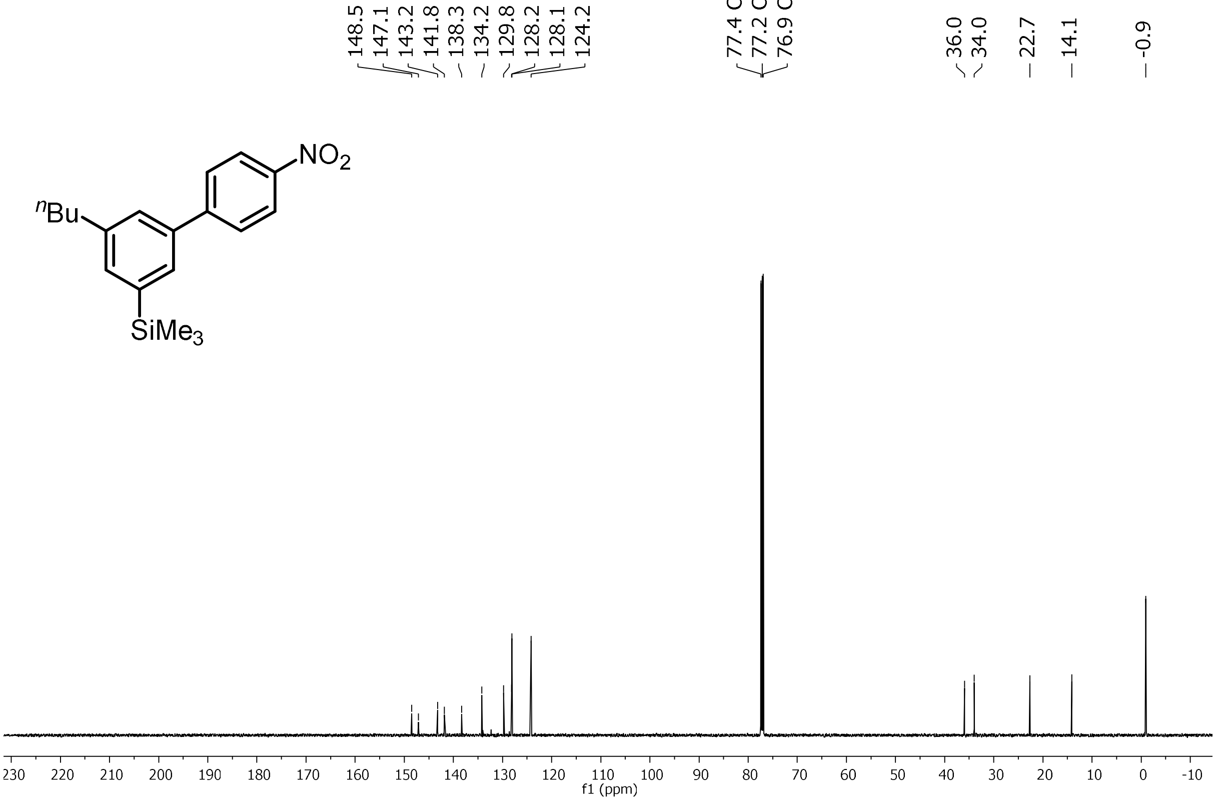

3-*n*-Butyl-5-iodo-4'-nitro-1,1'-biphenyl

1H NMR
(CDCl3, 600.44 MHz)

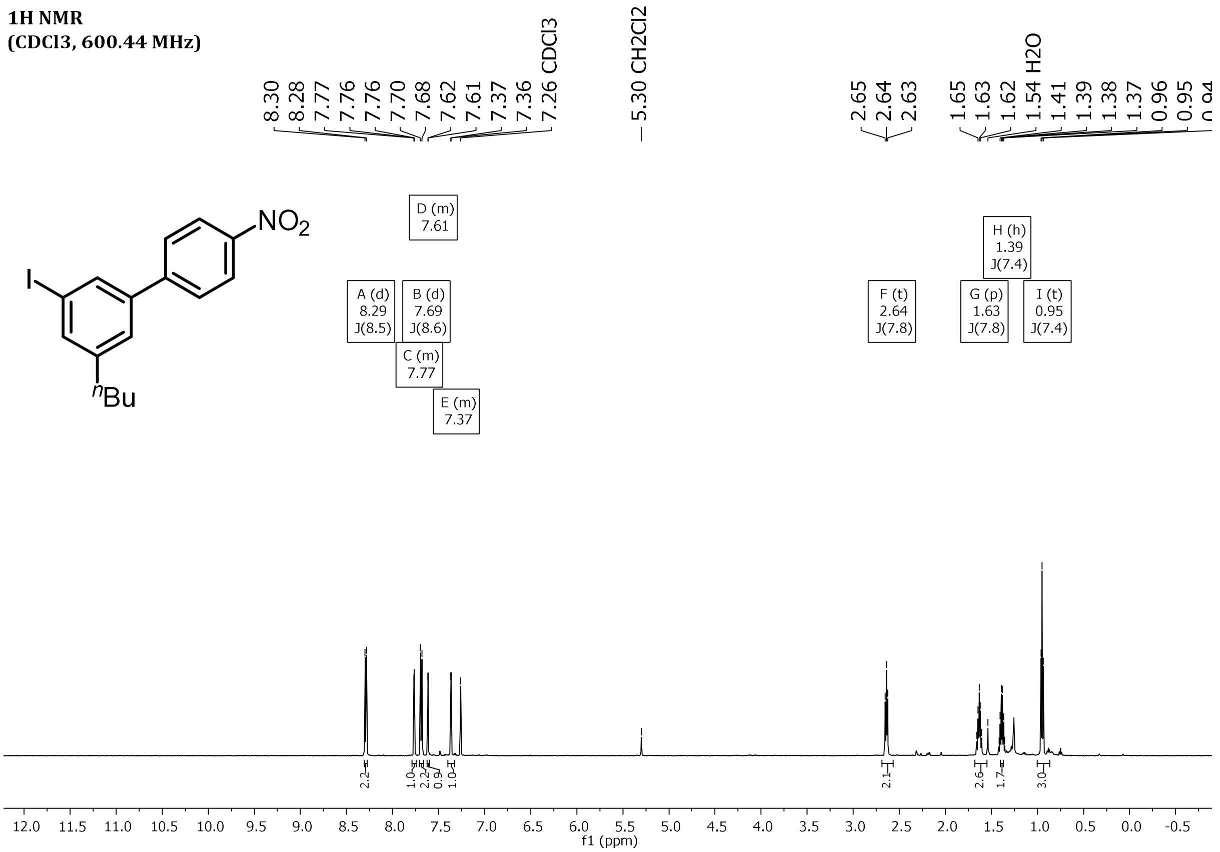

13C NMR
(CDCl3, 151.00 MHz)

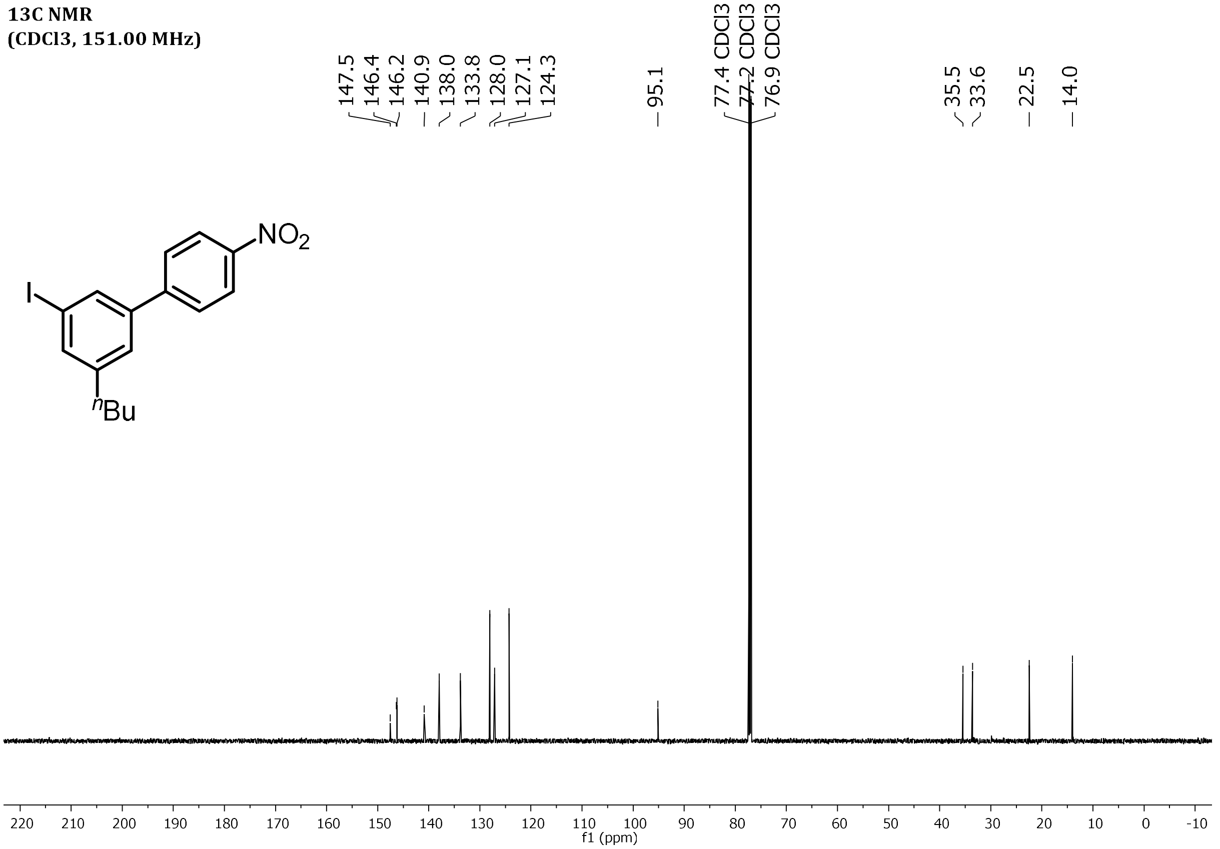

3-(4'-Methoxy-[1,1'-biphenyl]-4-yl)pyridine

1H NMR
(DMSO-d6, 599.86 MHz)

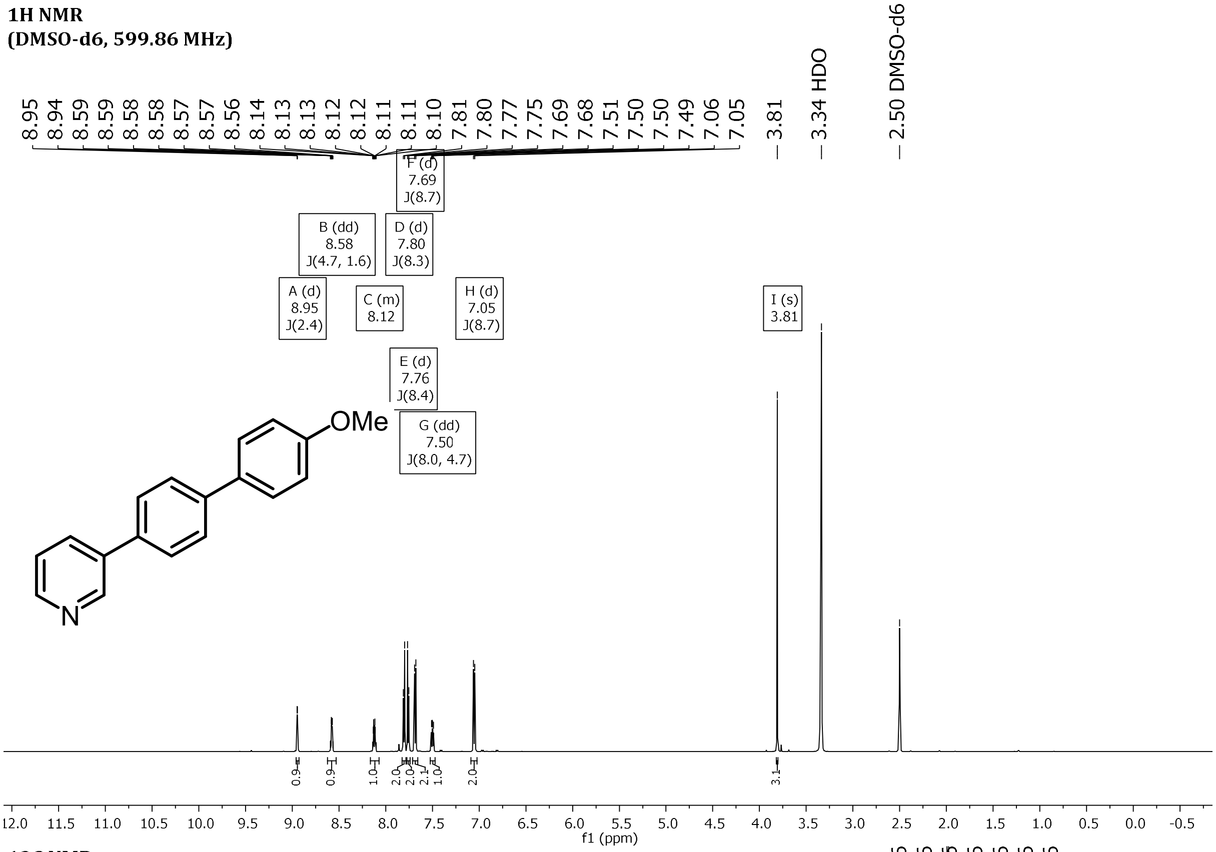

13C NMR
(DMSO-d6, 150.85 MHz)

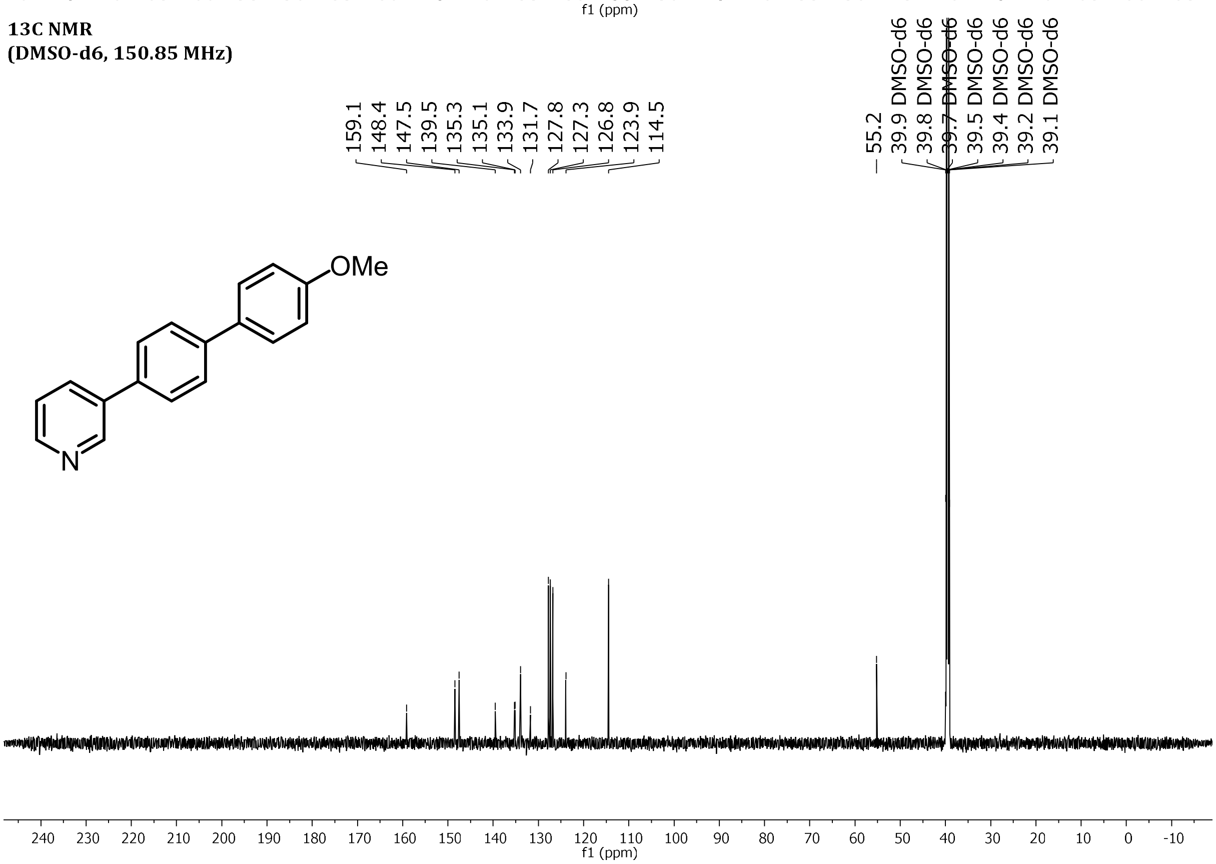

1-(4'-Bromo-5-(trimethylsilyl)-[1,1'-biphenyl]-3-yl)piperidine

1H NMR
(CDCl3, 599.86 MHz)

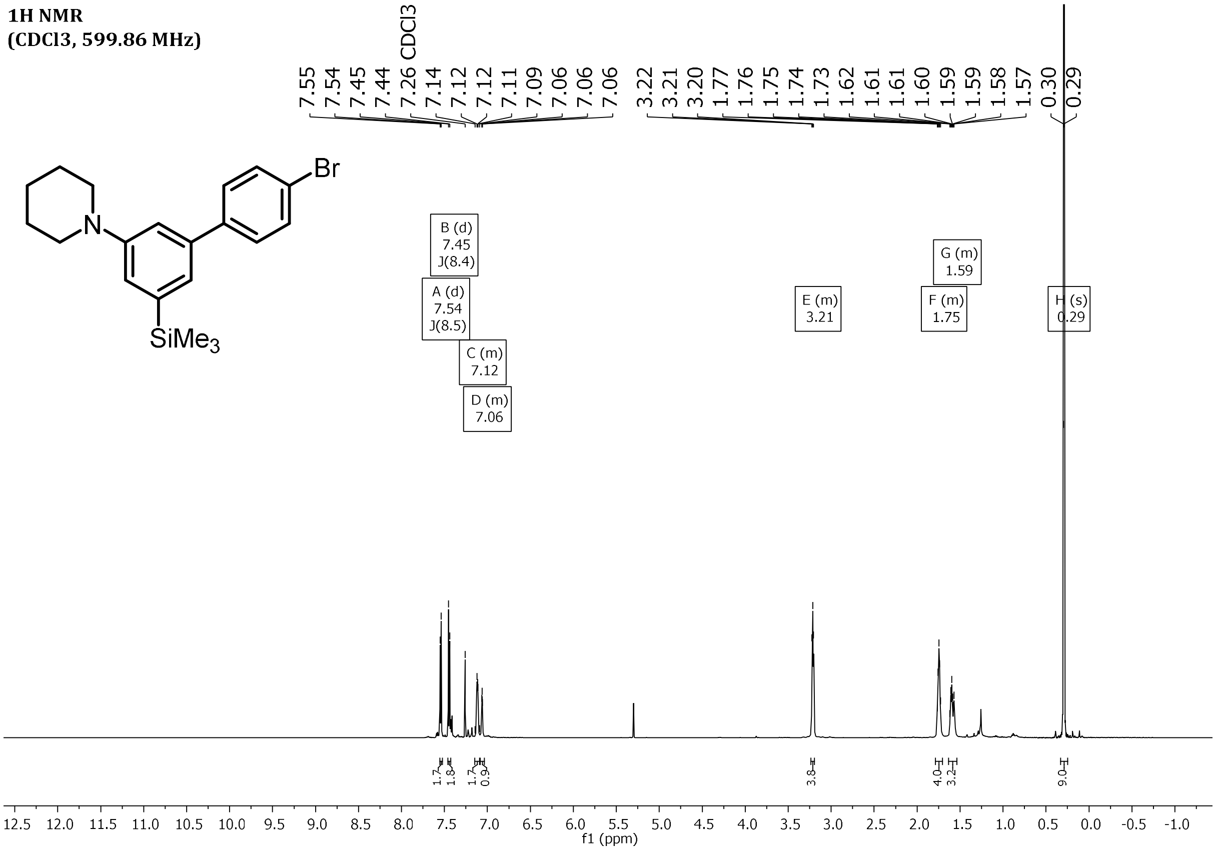

13C NMR
(CDCl3, 150.85 MHz)

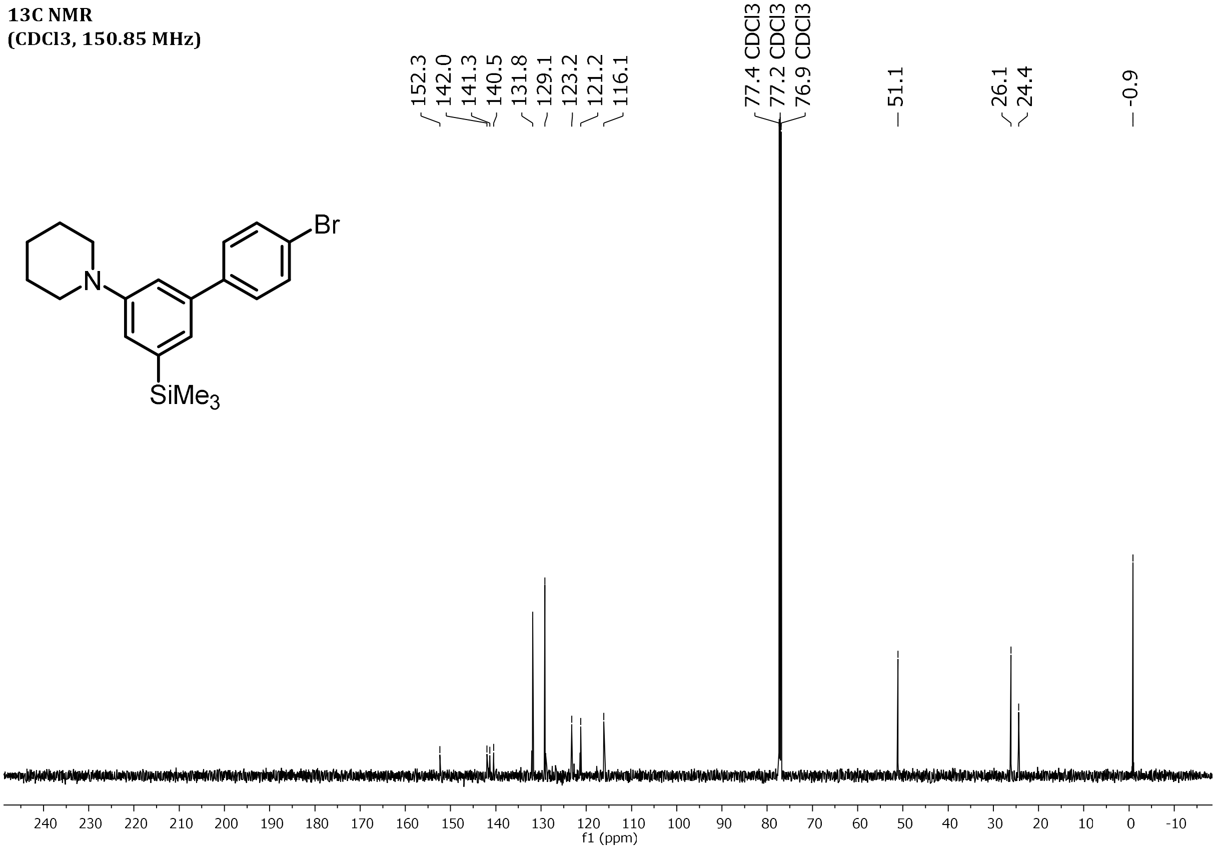

8. References

[1] W.-J. Zhou, K.-H. Wang, J.-X. Wang, D.-F. Huang, *Eur. J. Org. Chem.* **2010**, 416–419.

[2] J. Xie, K. Sekine, S. Witzel, P. Krämer, M. Rudolph, F. Rominger, A. S. K. Hashmi, *Angew. Chem. Int. Ed.* **2018**, *57*, 16648–16653.

[3] S. Chakraborty, J. Ahmed, B. K. Shaw, A. Jose, S. K. Mandal, *Chem. Eur. J.* **2018**, *24*, 17651–17655.

[4] S. Bernhardt, G. Manolikakes, T. Kunz, P. Knochel, *Angew. Chem. Int. Ed.* **2011**, *50*, 9205–9209.

[5] X. Chen, L. Zhou, Y. Li, T. Xie, S. Zhou, *J. Org. Chem.* **2014**, *79*, 230–239.

[6] W.-L. Chen, C.-Y. Chen, Y.-F. Chen, J.-C. Hsieh, *Org. Lett.* **2015**, *17*, 1613–1616.

[7] H. Minami, T. Saito, C. Wang, M. Uchiyama, *Angew. Chem. Int. Ed.* **2015**, *54*, 4665–4668.

[8] M.-H. Hsu, C.-M. Hsu, J.-C. Wang, C.-H. Sun, *Tetrahedron* **2008**, *64*, 4268–4274.

[9] M. Schiek, K. Al-Shamery, A. Lützen, *Synthesis* **2007**, 613–621.

[10] A. Moroda, H. Togo, *Tetrahedron* **2006**, *62*, 12408–12414.

[11] A. E. Brown, B. E. Eichler, *Tetrahedron Letters* **2011**, *52*, 1960–1963.

[12] M. Barbero, S. Dughera, *Tetrahedron* **2018**, *74*, 5758–5769.

[13] E. K. Reeves, J. N. Humke, S. R. Neufeldt, *J. Org. Chem.* **2019**, *84*, 11799–11812.

[14] R. Cai, M. Lu, E. Y. Aguilera, Y. Xi, N. G. Akhmedov, J. L. Petersen, H. Chen, X. Shi, *Angew. Chem. Int. Ed.* **2015**, *54*, 8772–8776.

[15] J. M. Hammann, F. H. Lutter, D. Haas, P. Knochel, *Angew. Chem. Int. Ed.* **2017**, *56*, 1082–1086.

[16] J.-H. Ho, Y.-C. Lin, L.-T. Chou, Y.-Z. Chen, W.-Q. Liu, C.-L. Chuang, *Tetrahedron Letters* **2013**, *54*, 1991–1993.

[17] M. R. Yadav, M. Nagaoka, M. Kashihara, R.-L. Zhong, T. Miyazaki, S. Sakaki, Y. Nakao, *J. Am. Chem. Soc.* **2017**, *139*, 9423–9426.

[18] T. Niwa, H. Ochiai, Y. Watanabe, T. Hosoya, *J. Am. Chem. Soc.* **2015**, *137*, 14313–14318.

[19] D. Wang, M. R. Talipov, M. V. Ivanov, R. Rathore, *J. Am. Chem. Soc.* **2016**, *138*, 16337–16344.

[20] D.-Y. Wang, C. Wang, M. Uchiyama, *J. Am. Chem. Soc.* **2015**, *137*, 10488–10491.

[21] Y.-Y. Chua, H. A. Duong, *Chem. Commun.* **2016**, *52*, 1466–1469.

[22] B. Xing, C. Ni, J. Hu, *Angew. Chem. Int. Ed.* **2018**, *57*, 9896–9900.

[23] X. Ma, S. B. Herzon, *Beilstein J. Org. Chem.* **2018**, *14*, 2259–2265.

[24] P. Nikolaienko, M. Rueping, *Chem. Eur. J.* **2016**, *22*, 2620–2623.

[25] W. Erb, A. Hellal, M. Albini, J. Rouden, J. Blanchet, *Chem. Eur. J.* **2014**, *20*, 6608–6612.

[26] P. S. Gribanov, M. A. Topchiy, Y. D. Golenko, Y. I. Lichtenstein, A. V. Eshtukov, V. E. Terekhov, A. F. Asachenko, M. S. Nechaev, *Green Chem.* **2016**, *18*, 5984–5988.

[27] C. Fricke, G. J. Sherborne, I. Funes-Ardoiz, E. Senol, S. Guven, F. Schoenebeck, *Angew. Chem. Int. Ed.* **2019**, *58*, 17788–17795.

[28] C. Fricke, A. Dahiya, W. B. Reid, F. Schoenebeck, *ACS Catal.* **2019**, *9*, 9231–9236.

[29] M. J. Harper, E. J. Emmett, J. F. Bower, C. A. Russell, *J. Am. Chem. Soc.* **2017**, *139*, 12386–12389.

[30] K. Gondo, J. Oyamada, T. Kitamura, *Org. Lett.* **2015**, *17*, 4778–4781.

[31] I. Kalvet, T. Sperger, T. Scattolin, G. Magnin, F. Schoenebeck, *Angew. Chem. Int. Ed.* **2017**, *56*, 7078–7082.

[32] J. C. Vantourout, R. P. Law, A. Isidro-Llobet, S. J. Atkinson, A. J. B. Watson, *J. Org. Chem.* **2016**, *81*, 3942–3950.

[33] Gaussian 16, Revision C.01, M. J. Frisch, G. W. Trucks, H. B. Schlegel, G. E. Scuseria, M. A. Robb, J. R. Cheeseman, G. Scalmani, V. Barone, G. A. Petersson, H. Nakatsuji, X. Li, M. Caricato, A. V. Marenich, J. Bloino, B. G. Janesko, R. Gomperts, B. Mennucci, H. P. Hratchian, J. V. Ortiz, A. F. Izmaylov, J. L. Sonnenberg, D. Williams-Young, F. Ding, F. Lipparini, F. Egidi, J. Goings, B. Peng, A. Petrone, T. Henderson, D. Ranasinghe, V. G. Zakrzewski, J. Gao, N. Rega, G. Zheng, W. Liang, M. Hada, M. Ehara, K. Toyota, R. Fukuda, J. Hasegawa, M. Ishida, T. Nakajima, Y. Honda, O. Kitao, H. Nakai, T. Vreven, K. Throssell, J. A. Montgomery, Jr., J. E. Peralta, F. Ogliaro, M. J. Bearpark, J. J. Heyd, E. N. Brothers, K. N. Kudin, V. N. Staroverov, T. A. Keith, R. Kobayashi, J. Normand, K. Raghavachari, A. P. Rendell, J. C. Burant, S. S. Iyengar, J. Tomasi, M. Cossi, J. M. Millam, M. Klene, C. Adamo, R. Cammi, J. W. Ochterski, R. L. Martin, K. Morokuma, O. Farkas, J. B. Foresman, and D. J. Fox, Gaussian, Inc., Wallingford CT, 2016.
